# Genome‐wide analysis of European sea bass provides insights into the evolution and functions of single‐exon genes

**DOI:** 10.1002/ece3.7507

**Published:** 2021-04-02

**Authors:** Mbaye Tine, Heiner Kuhl, Peter R. Teske, Richard Reinhardt

**Affiliations:** ^1^ UFR des Sciences Agronomiques, de l'Aquaculture et des Technologies Alimentaires (S2ATA) Université Gaston Berger (UGB) Saint‐Louis Senegal; ^2^ Genome Centre at the Max‐Planck Institute for Plant Breeding Research Köln Germany; ^3^ Department of Ecophysiology and Aquaculture Leibniz‐Institute of Freshwater Ecology and Inland Fisheries (IGB) Berlin Germany; ^4^ Department of Zoology Centre for Ecological Genomics and Wildlife Conservation University of Johannesburg Johannesburg South Africa

**Keywords:** comparative genomics, *Dicentrarchus labrax*, European sea bass, evolution, promoter, single‐exon gene

## Abstract

Several studies have attempted to understand the origin and evolution of single‐exon genes (SEGs) in eukaryotic organisms, including fishes, but few have examined the functional and evolutionary relationships between SEGs and multiple‐exon gene (MEG) paralogs, in particular the conservation of promoter regions. Given that SEGs originate via the reverse transcription of mRNA from a “parental” MEGs, such comparisons may enable identifying evolutionarily‐related SEG/MEG paralogs, which might fulfill equivalent physiological functions. Here, the relationship of SEG proportion with MEG count, gene density, intron count, and chromosome size was assessed for the genome of the European sea bass, *Dicentrarchus labrax*. Then, SEGs with an MEG parent were identified, and promoter sequences of SEG/MEG paralogs were compared, to identify highly conserved functional motifs. The results revealed a total count of 1,585 (8.3% of total genes) SEGs in the European sea bass genome, which was correlated with MEG count but not with gene density. The significant correlation of SEG content with the number of MEGs suggests that SEGs were continuously and independently generated over evolutionary time following species divergence through retrotranscription events, followed by tandem duplications. Functional annotation showed that the majority of SEGs are functional, as is evident from their expression in RNA‐seq data used to support homology‐based genome annotation. Differences in 5′UTR and 3′UTR lengths between SEG/MEG paralogs observed in this study may contribute to gene expression divergence between them and therefore lead to the emergence of new SEG functions. The comparison of nonsynonymous to synonymous changes (Ka/Ks) between SEG/MEG parents showed that 74 of them are under positive selection (Ka/Ks > 1; *p* = .0447). An additional fifteen SEGs with an MEG parent have a common promoter, which implies that they are under the influence of common regulatory networks.

## Graphical abstract

This study investigated the relationship of SEG proportion with MEG count, gene density, intron count, and chromosome size for the genome of sea bass, *Dicentrarchus labrax*. Then, SEGs with an MEG parent were identified, and promoter sequences of SEG/MEG orthologs were compared, to identify highly conserved functional motifs. The results revealed a significant correlation between SEG and MEG counts over the genome and allowed identifying SEG/MEG orthologs that share the same promoter sequence, suggesting that they are under the influence of common regulatory networks.

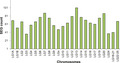

## 1 INTRODUCTION

Early comparative genomic studies on eukaryotes showed that the majority of their genes consist of multiple exons, including coding sequences and untranslated regions, or UTRs. These are the precursors of mRNA and are interrupted by noncoding sequences called introns (Long, 2002; Rogozin et al., 2005; Smith, 1988). These introns are spliced out and exons are concatenated to form the mature mRNA, which is then expressed as a protein product (Rogozin et al., 2005). Recent studies using high‐throughput sequencing technologies have revealed that the genomes of both unicellular and multicellular organisms are composed of a significant proportion of single‐exon genes (SEGs), also called intronless genes (Tay et al., 2009; Tine et al., 2011; Venter, 2001; Yang et al., 2009; Zou et al., 2011). This discovery has raised serious questions about the origin, evolution, and function of this type of genes (Fablet et al., 2009; Savisaar & Hurst, 2016; Shabalina et al., 2010; Zou et al., 2011). Most studies on the origin of SEGs and evolution suggest that they are the result of the reverse transcription of mRNA from a “parental” gene into cDNA and its insertion elsewhere in the genome (Ostertag & Kazazian, 2001). This process, known as retrotransposition, is mediated by long interspersed nuclear element 1 (LINE 1)‐derived enzymes, which encode a reverse transcriptase enzyme that can produce a DNA copy from any RNA molecule in the cell (Cordaux & Batzer, 2008; Doenecke & Albig, 2005; Kaessmann et al., 2009; Sakharkar et al., 2006). There are two possible outcomes concerning the fate of the retro‐transcribed DNA copy. It can either be integrated into a silent location (the most frequent case) where there are no regulatory elements that can promote its transcription (Cooper, 2005; Kaessmann et al., 2009). These sequences, often called retropseudogenes, are under relaxed selection and remain dormant because they lack a regulatory region, and they will most likely eventually be deleted (Cooper, 2005). Alternatively, the retro‐transcribed DNA copy can be integrated near a resident functional promoter that can promote its activation (Xing et al., 2006). This latter case results in active retrogenes that, during the course of evolution, may undergo subfunctionalization and then either share function with the parents, develop a new function through a neofunctionalization process, or completely replace the parental multiple‐exon gene. These retrogenes may then expand in number by duplication and/or recombination events (Altschul et al., 1997; Gentles, 1999).

Most comparative genomic studies on SEGs have focused on their inventory and relative proportions in the genome (Jorquera et al., 2016; Navarro & Galante, 2013; Sakharkar & Kangueane, 2004). Few studies have explored the evolution and functional divergence of SEGs (Grzybowska, 2012; Sakharkar et al., 2006; Shabalina et al., 2010; Zou et al., 2011), and to our knowledge, no study has investigated the adaptive roles that this type of gene may play in living organisms. We have previously demonstrated that a relatively small proportion of claudin SEGs, the largest SEG family in vertebrates, may originally have coexisted with claudin MEGs in the common ancestor of all vertebrate species (Tine et al., 2011). The claudin SEGs were likely inherited from the common ancestor of fishes and other vertebrates. Further tandem duplications may have occurred in teleost fishes, resulting in multiple copies, which may explain the greater number of claudin SEG paralogs in this lineage compared with mammals (Loh et al., 2004). We have also demonstrated that many of newly emerged teleost SEGs may have evolved new functions (Tine et al., 2011), through adaptive functional divergence of encoded proteins, as previously demonstrated in both vertebrates and invertebrates (Emerson et al., 2004; Rosso et al., 2008), where retrogenes have evolved into novel protein‐coding genes with new functions (Marques et al., 2005; Rosso et al., 2008).

The SEGs encoding functional proteins are involved in various biological processes, from early developmental to mature stages and resistance to diverse stressors that organisms must deal with during their life cycle (Kaessmann et al., 2009). The regulation of the expression of genes is crucial for the accomplishment of their biological functions and is under the strict control of various regulatory elements including promoters, enhancers, and repressors (Gordon et al., 2009; Lettice et al., 2003; Vavouri et al., 2006). Promoters contain short motifs where transcription factors bind to regulate the transcription, and the sequences of many of them have been characterized in eukaryotic organisms, including fishes (Molina et al., 2001; Streelman & Kocher, 2002; Tchoudakova et al., 2001; Velan et al., 2015). Although the length and motif content of promoters, as well as their position relative to the 5′UTR, may vary considerably (up to several Kbp upstream of a gene's transcription start site, or TSS) (Placido et al., 2006), some core promoters can occur in short sequences from 100 to 300 bp upstream or downstream of the TSS (Smale & Kadonaga, 2003). Given that genes are organized into common pathways to accomplish their activity (Segal et al., 2003), those that are under the influence of common regulatory networks (i.e., genes that share the same regulatory elements, including promoter motifs) may have similar expression patterns, which implies that they may be involved in the same biological or physiological processes. Genes that arise from retroduplication need to recruit regulatory elements to be transcribed and are therefore more likely to have evolved new functions compared with genes that resulted from segmental DNA duplications (Kaessmann et al., 2009).

In a previous study, 78 SEGs (5.30% of the total gene count) were identified on three different sea bass chromosomes (Tine et al., 2011). Comparative analyses revealed that the fraction of SEGs predicted on these chromosomes is slightly higher than that found in the whole genome of other teleosts (*Takifugu rubripes*, *Tetraodon nigroviridis*, *Oryzias latipes*, *Gasterosteus aculeatus*, and *Danio rerio*). The comparison with stickleback *G. aculeatus* revealed that the count, composition, and order of SEGs varied significantly among corresponding chromosomes. Accordingly, Tine et al. (2011) proposed that these genes have continuously and independently evolved through retrotranscription followed by tandem duplications.

The main objective of the present study was to assess the proportion of SEGs found in the complete European sea bass genome, and to identify features conserved or divergent between SEG and MEG paralogs, which might give new insights into the evolution of SEGs in teleost fishes. Identifying conserved features that may confer specific functions may facilitate a better understanding of the biological functions of SEGs. Using annotations of the sequenced genome of the European sea bass, *Dicentrarchus labrax*, we first identified all SEGs present in the genome and then described their occurrence across chromosomes. We then investigated the relationship between SEGs and other components of the genome such as chromosome size, MEG counts, gene density, and intron counts, to infer their origin. The ratio of nonsynonymous to synonymous changes was compared between SEG and MEG paralogs. More importantly, the promoter sequences of SEG and MEG paralogs were compared to identify SEGs that share the same promoter with their parental MEG. The results revealed a significant correlation between SEG and MEG counts over the genome and allowed identifying SEG/MEG paralogs that share the same promoter sequence, suggesting that they are under the influence of common regulatory networks.

## 2 MATERIALS AND METHODS

### 2.1 SEG inventory

The SEGs present in the *D. labrax* genome were extracted from the sea bass UCSC Genome Browser (http://seabass.mpipz.de/index.html) using the Table browser option. A single‐exon filtering step (ExonCount ≤1 exon) was performed to select genes with only one exon. The nucleotide and encoded protein sequences of these genes were downloaded from the browser, together with the table containing information on exon/intron counts for each gene. Each SEG sequence was blasted against a local database comprising all genes identified as SEGs. The results were filtered to identify duplicates (SEGs with the same nucleotide sequence) and singleton genes (unique genes).

### 2.2 Identification of SEGs and MEG parents

The SEG nucleotide sequences were queried against the European sea bass genome to retrieve paralog MEGs using the BLAT algorithm (Kent, 2002). The BLAT results were carefully checked and manually edited, if necessary. Two SEG loci were considered to be duplicates if the two corresponding sequences matched aligned blocks with an average length of at least 100 bp of nucleotide sequence with ≥40% identity. The same approach was used to identify the number of SEGs with an MEG parent located on the same or a different chromosome. The search for reciprocal best hits (RBH) was performed against the European sea bass genome using both nucleotide and amino acid sequence queries. An MEG was assumed to be a parent of an SEG if they matched on an average length of ≥70% with at least 40% identity. The use of these strict criteria allowed identification of SEGs and their MEG parent loci with high confidence, and to ascertain which SEGs are represented multiple times in the European sea bass genome.

### 2.3 Correlations between SEGs and the other gene entities

The relationships between SEG and MEG counts in the genome were estimated using a Pearson correlation test (Pearson, 1895). The correlation between SEG content and other entities of the genome, such as chromosome size, gene density, and intron count, was also evaluated using the same correlation test. The tests were performed with the R software v.64.3.1.1 (Ihaka & Gentleman, 1996). A probability of less than 5% (*p* < .05) was considered as fiducial level of significance.

### 2.4 Test for natural selection

The ratio of nonsynonymous to synonymous (d_N_/d_S_) substitutions, also called Ka/Ks, which is indicative of the type of natural selection acting on protein sequences, was estimated for all pairs of SEG duplicates using the Nei and Gojobori (NG) method implemented in Ka/Ks_Calculator software (Zhang et al., 2006). The tests were conducted using nucleotide sequences to determine whether singleton genes are under comparable evolutionary constraints, or whether they are subjected to different rates of evolution. Several methods have been incorporated into the Ka/Ks software calculator for the estimation of Ka/Ks ratios, which include NG (Nei & Gojobori, 1986), LPB (Molina et al., 2001; Pamilo & Bianchi, 1993), MLPB (modified LPB) (Tzeng et al., 2004), MLWL (modified LWL) (Tzeng et al., 2004), MYN (modified YN) (Zhang et al., 2006), and YN (Yang et al., 2009). The Ka/Ks_Calculator software uses a maximum‐likelihood framework extended from the method of Zhang et al. (2006), which takes into account transition/transversion rate ratio and nucleotide frequencies, and incorporates these evolutionary features into a codon‐based model. All these methods were tested, and the results were not significantly different between methods. Hence, only the results of the NG method were reported, and Fisher's exact test (Fisher, 1922) was used to test the significance of differences in Ka, Ks, and Ka/Ks ratios between SEG/MEG parents and between SEG/SEG paralogs.

### 2.5 Identification of 3′UTR and 5′UTR, and promoter region

Sequences of the three‐prime untranslated region (3′UTR) and the five‐prime untranslated region (5′UTR) of SEGs and MEG parents were extracted from the BLAT results. Although the length of promoter sequences and the size of motifs can vary considerably, it has been demonstrated that core promoters can be contained in short sequences from ~100 bp upstream and downstream of the start site of the transcription. As we expected that some promoters might be present in the region 1,000 bp upstream of the transcription start site (TSS) of these genes, we extracted 1,000 bp sequences upstream of the potential promoter of SEGs and MEGs from the European sea bass UCSC Genome Browser. The promoter motif characteristics of promoter regions were identified using the PROMOTER PREDICTOR software, version 2.2 (March 1999), implemented on the Berkeley Drosophila Genome Project website (http://www.fruitfly.org/seq_tools/promoter.html). This software allows promoter predictions for eukaryotic sequences and can be used for promoter prediction in teleost fishes. A score cutoff of 0.80 was used as a threshold. When motif characteristics of a promoter region were identified, they were aligned against the existing motifs in public sequences databases, and the corresponding sequences were manually compared between SEG and MEG paralogs to confirm that they are shared.

Given that the aim of these analyses was to specifically identify shared promoters between SEG and MEG paralogs, the promoter region analysis was performed only with SEG and MEG sequences where it was possible to extract the true promoter region with a high level of confidence. A conserved sequence filtering approach using an in‐house script was applied to eliminate SEG and MEG pairs with erroneously identified common regulatory elements. This filtering procedure allowed to select a high‐confidence subset of SEG and MEG parents with conserved promoter regions and to avoid contamination of the dataset with other types of conserved sequence. Then, an all‐against‐all promoter sequence comparison was performed on the first set of SEG/MEG sequences with identified promoter pairs using PROMOTER PREDICTOR, and it was determined how effectively the expected pairs were recovered. The results from the all‐against‐all comparison have the advantage to indicate whether the conserved regions are specific to particular promoters or whether they reflect more general signals that appear in many promoter regions.

### 2.6 Functional annotation

Gene ontology terms assigned to all SEGs identified were downloaded from the European sea bass UCSC genome browser (http://seabass.mpipz.mpg.de/). This functional annotation was performed for all genes that were characterized and annotated in the European sea bass genome, including SEGs. The annotation was done using Blast2GO (https://www.blast2go.com/). Here, only the second level of GO terms (based on cellular component, biological process, and molecular function) is presented.

## 3 RESULTS

### 3.1 SEG counts and correlation analyses

**FIGURE 1:**
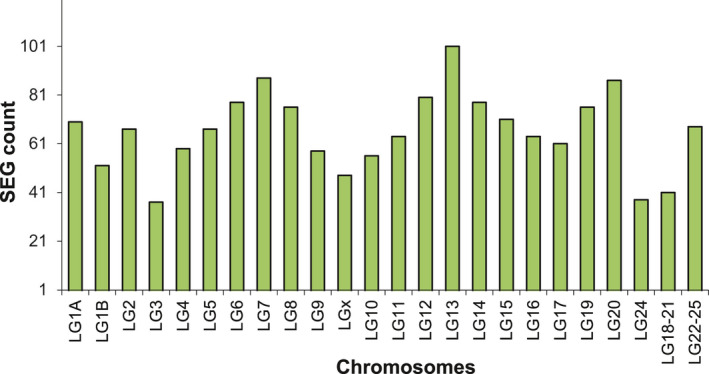
Single‐exon gene counts shown for each chromosome in the genome of European sea bass

The total number of SEGs recorded from the 24 chromosomes of the European sea bass genome was 1,585. The highest counts of SEGs were recorded on chromosomes LG13 (101), LG7 (88), and LG20 (87), followed by LG6 and LG14 (78 each), and followed by LG19 (76), LG1A (70), and LG5 (67), and the lowest numbers were observed on LG3 (37), LG24 (38), LG18‐21 (41), LGx (48), and LG1B (52) (Figure 1).

**FIGURE 2:**
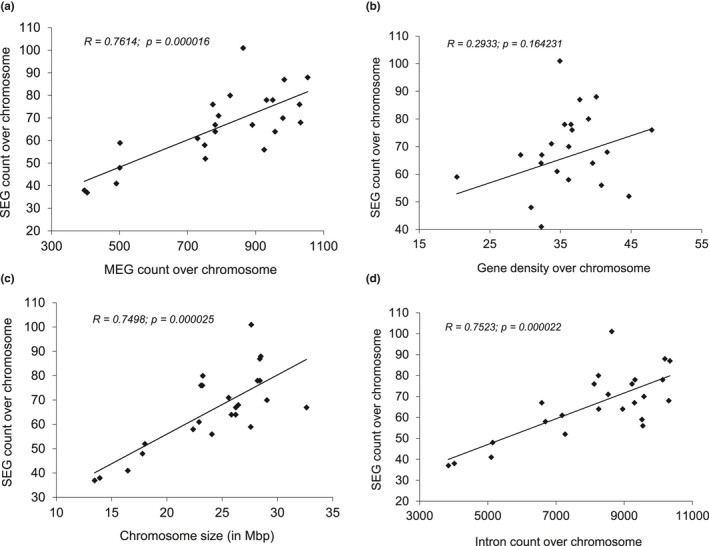
Correlation of single‐exon gene (SEG) count with multiple‐exon gene (MEG) count (a), gene density (b), chromosome size (c), and intron counts over chromosome (d)

The name and chromosomal location of all MEGs and SEGs used in the correlation analyses are indicated in Appendix S1. The number of SEGs on chromosomes was significantly positively correlated with the number of MEGs over chromosome (*R* = 0.7614; *p* = .000016) (Figure 2a), but no significant correlation was found between the proportion of SEGs over chromosome and gene density (*R* = 0.2933; *p* = .164231) (Figure 2b). The SEG count was significantly correlated with chromosome size (*R* = 0.7498; *p* = .000025) (Figure 2c). Likewise, there was a statistically significant positive correlation between the number of SEGs and the proportion of introns over chromosomes (*R* = 0.7523; *p* = .000022) (Figure 2d), which was strongly positively correlated with the number of genes (*R* = 0.8665; *p* < .00001).

### 3.2 SEGs with parental MEGs

Of the 1,585 SEGs identified in the European sea bass genome, 312 have MEG paralogs. Based on their similarity, these MEGs can be considered to be parents of their SEG paralogs. The number of SEGs with an MEG parent located on different chromosomes was higher than the number of SEGs with an MEG parent located on the same chromosome. Chromosome LG7 has more SEGs with parental MEGs (20), whereas chromosome LG1B has fewer (5) SEGs with parental MEGs. The number of SEGs with parent MEGs was not correlated with the number of MEGs or SEGs over chromosomes. Likewise, there was no significant correlation between the number of SEGs with a parental MEG with chromosome size, intron count, or gene density.

### 3.3 UTR conservation between SEGs and MEGs

**FIGURE 3:**
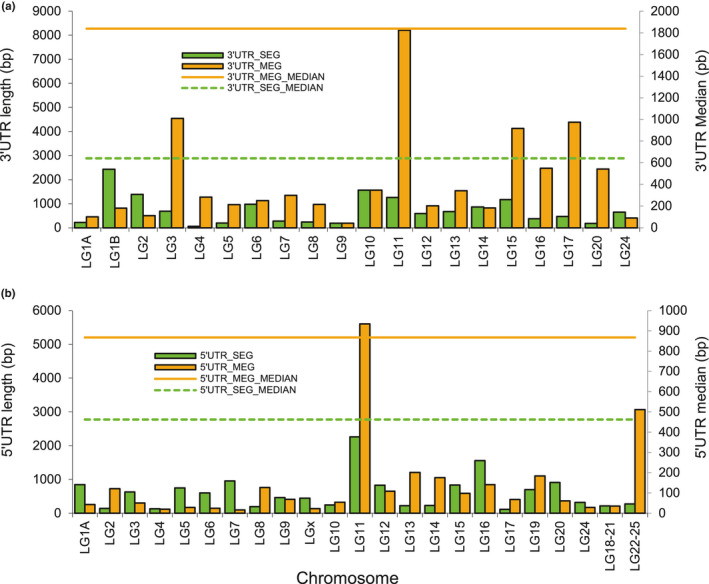
Comparison of the average length of (a) 3′UTR length between single‐exon genes (SEGs) and multiple‐exon genes (MEGs) and (b) 5′UTR length between SEG and MEG paralogs for each chromosome

**FIGURE 4:**
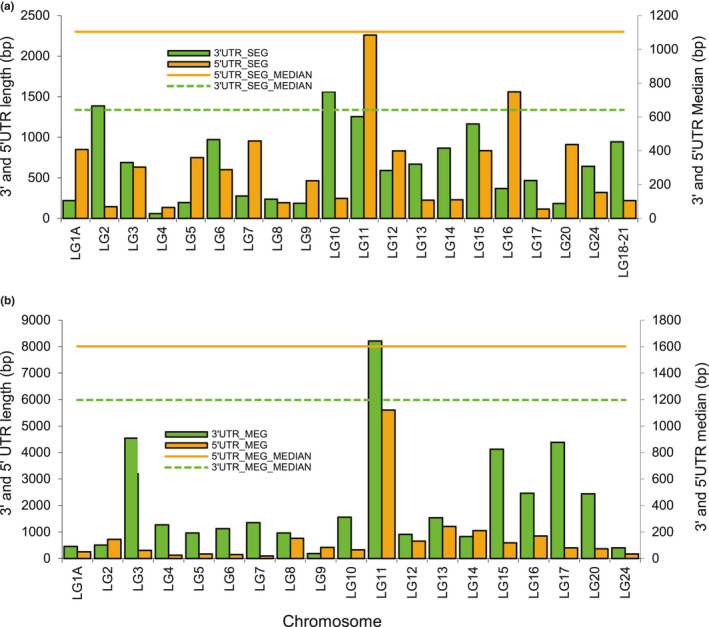
Comparison of the average length of (a) 3′UTR and 5′UTRs between single‐exon genes (SEGs) with multiple‐exon genes (MEG) and (b) 3′UTR and 5′UTR length of SEGs and MEGs over chromosome paralogs

Most of the SEGs identified in the European sea bass genome are without 3′UTR or 5′UTR, or even lacked both ends, which is likely an artifact of using protein sequences from other fish species that did not include UTR sequences for annotation. For most of the chromosomes, the comparison of the average length of the 3′ and 5′ ends between SEG and MEG showed that the latter have the largest 3′UTR (Figure 3a). The median length of the MEGs was also larger than that of the SEGs (Figure 3a). Given that both SEGs and MEGs were annotated using the same procedure, that is, using protein sequences from other teleosts, it is improbable that these results reflect an annotation bias indicative of a more reliable MEG annotation. By contrast, for most of the chromosomes, the average length of 5′UTR of SEGs was larger than that of MEGs, but the median of these latter was larger (Figure 3b). Among all SEGs with an MEG parent analyzed, most had a 3′UTR that was larger than the corresponding 5′UTR (Figure 4a). Likewise, for most of the chromosomes, the average length of the 3′UTR end was greater than that of the 5′UTR end (Figure 4b). However, the median length of 5′UTRs of both SEGs and MEGs was longer than that of 3′UTRs (Figure 4a,b).

### 3.4 Functional categories

The Blast2GO annotation allowed classifying 797 SEGs in functional categories of cellular components. These SEGs are distributed in the following categories as follows: *integral to membrane* (43.22%), *membrane* (16.22%), *extracellular region* (5.87%), *nucleus* (5.03%), *mitochondrion* (23.64%), *intracellular part* (2.52%), *troponin complex* (2.24%), *endoplasmic reticulum* (1.68%), *cell part* (1.68%), *cytoskeleton* (1.12%), and *others* (12.73%). The category *others* comprises 42 different subfunctional categories with one to two SEGs each. The GO annotation also allowed the classification of 295 SEGs in different functional categories of biological processes (FCBPs). These SEGs were assigned to 30 FCBPs, of which the most frequently represented are *regulation of apoptotic process*, *response to chemical stimulus*, *multicellular organismal development*, *termination of G‐protein‐coupled receptor signaling pathway*, *cell–cell signaling,* and *defense response to bacterium* (2%–2.80% each), and *cell redox homeostasis*, *response to stress*, *single‐organism cellular process* (3.15% each), *cell process* (4.90%), *single‐organism process* (4.90%), and *signal transduction* (5.59%). Other, less frequently represented (1%–1.75% each) FCBPs include *autophagy*, *hemopoiesis*, *nervous system development*, *spermatogenesis*, *cell wall macromolecular catabolic process*, *intracellular signal transduction*, *lipid metabolic process*, *phosphorylation,* and *others*. The group *others* includes 82 categories comprising only one SEG each (25.52%). Genes annotated with the GO biological process term for *cell process*, *single‐organism process,* and signal transduction are found predominantly in a subset of 295 SEGs. Finally, 1,524 SEGs could be classified into different functional categories of molecular function. The most important of these are as follows: *protein binding* (60.76%), *GTP binding* (4.82%), *carbohydrate binding* (4.55%), *zinc ion binding* (4.27%), *nucleic acid binding* (2.93%), *ATP binding* (2.93%), *calcium ion binding* (2.58%), *DNA binding* (2.31%), *hydrolase activity* (1.55%), and *others* (51 categories) (11.26%). Genes taking part in protein binding activities are found predominantly in this set of 1,524 SEGs that could be classified into molecular function categories.

The GO annotation of SEGs with an MEG parent revealed that 63.02% of them have a protein name, whereas 31.16% were not annotated (referred as NA in Appendix S1). The remaining SEGs with an MEG parent (5.82%) are annotated as uncharacterized or unnamed proteins. The 63.02% of SEGs with a protein name belong to several gene families, including leucine‐rich repeat protein (3.15%), claudin (2.64%), G‐protein‐coupled receptor (2.54%), e3 ubiquitin–protein ligase (2.13%), forkhead box protein (2.13%), and reverse transcriptase protein (2.13%). Besides these well‐represented gene families, there were other, less common gene families, which include odorant receptor (1.93%), transcription factor (1.83%), transposase (1.83%), transmembrane protein (1.73%), transposable element tc1 and tcb2 transposase (1.73,%), gap junction alpha, beta and gamma (1.62%), tripartite motif‐containing protein (1.62%), and zinc finger protein (1.62%). We also identified other well‐known gene families including histone protein (0.91%), nuclear factor ovary‐like (0.91%), ion channels (0.91%), c‐c chemokine receptor type 11‐like (0.81%), interferon‐induced very large GTPase 1 (0.51%), and fibroblast growth factor‐binding protein 1 (0.41%). Heat shock proteins including heat shock protein 30 and heat shock protein 70 as well as transcriptional activator protein pur‐alpha and pur‐beta are also represented (0.30% each).

### 3.5 Comparison of potential promoters between SEGs and MEGs

Of the 312 SEG and MEG pairs analyzed, 34 have very similar sequence 1,000 bases upstream of the TSS, which might contain the promoter, whereas 278 others did not show similarity in the upstream sequence. The similarity search for sequences characteristic of promoter regions allowed the identification of 15 promoter motifs with a cutoff score of 0.80 (Appendix S2). The average size of the motifs found was 50 bp, including a ~10 bp upper bound of the transcription start binding site (Appendix S2).

### 3.6 Natural selection

The ratios of nonsynonymous to synonymous (Ka/Ks) substitutions could be estimated for 110 SEG/MEG pairs of the 312 SEGs with an MEG parent. The Ka/Ks could not be estimated for the 201 remaining SEG/MEG parents because one of the paralogs lacked the translation start codon required by the Ka/Ks_Calculator program, which might be due to an annotation bias. The average ratio of nonsynonymous to synonymous changes (Ka/Ks) for all SEG/MEG pairs was 1.40 (*p* = .0480), which is indicative of positive selection. The comparison of Ka/Ks between 110 SEG/MEG parents showed that 74 of them are under positive selection (Ka/Ks > 1; *p* = .0447) (Appendix S1), whereas the remaining 36 were not (Ka/Ks < 1). SEG/MEG pairs with a Ka/Ks > 1 include transposase, reverse transcriptase‐like protein, nuclear factor ovary‐like, immunoglobulin light chain precursor, c‐c chemokine receptor type 4‐like, and transposable element/transposase and sry (sex‐determining region y)‐box 4 (Appendix S1). Few of them are not annotated genes or were annotated as uncharacterized protein, which means that the corresponding protein is not present in Gene Ontology repertory.

The average Ka/Ks ratio estimated for 33 SEG/SEG paralogs was 1.01. Of these 33 SEG duplicates for which the Ka/Ks could be estimated, 16 are under positive selection (Ka/Ks > 1; *p* = .0307), whereas the 17 remaining paralog pairs were not (all Ka/Ks < 1).

## 4 DISCUSSION

The main objective of the current study was to identify features conserved or divergent between SEG and MEG paralogs, which may confer a specific function. This may improve our understanding of the biological roles of this type of gene, which has long been considered to be marginal and dysfunctional. The proportion of SEGs in the European sea bass genome was accessed at both chromosomal and genome levels. The results showed significant correlations of SEG count with the proportion of MEGs, chromosome size, and intron count, but no significant correlation with gene density was found. All these correlations indicate that SEGs are evenly spread over the genome. The results also showed that SEG order and composition varied among corresponding chromosomes. The SEG fraction on a particular chromosome is also correlated with the chromosome's total gene content, which suggests that SEGs are distributed across the genome. Functional annotation by gene ontology indicated that SEGs code for a variety of protein families, including leucine‐rich repeat protein, claudins, forkhead box protein, olfactory receptors, histones, and ion channels, all of which are essential for various biological functions. In addition, several ion channels were identified, including potassium voltage‐gated channel and potassium sodium channels, which play important roles in hydromineral balance. Likewise, heat shock proteins, including heat shock protein 30 and heat shock protein 70, which are involved in responses to environmental constraints (Currie et al., 2000; Tine et al., 2010; Oksala et al., 2014), were also identified. The GO functional annotation indicated that a significant number of SEGs belong to different functional categories of cellular component, biological process, and molecular function. The GO results indicated that 295 SEGs are involved in important biological pathways. This finding is supported by the transcriptomic results from the RNA‐seq data used to support the European sea bass genome annotation, which indicates that of the 1,587 SEGs identified, 1,234 are expressed, suggesting that they are functional. However, given that SEGs result from the reverse transcription of mRNA from a “parental” gene into cDNA and its insertion elsewhere in the genome, the transcriptomic data were unsuitable to differentiate between SEGs and their MEG parents. The RNA‐seq data were not specially produced to compare the expression profiles. For that reason, they could not be used to distinguish any SEG that is highly expressed from its parental MEG. Such information could provide strong evidence of SEG functionality compared with their MEG parents. Further transcriptomic analyses using real‐time PCR or RNA‐seq, specially designed to compare expression profiles, are required to identify differentially expressed SEG/MEG parents.

Many features that contribute to the stability of mRNA, as well as its translation and regulation, are located within untranslated regions (UTRs). The 3′UTR that is located downstream from the coding region is not translated into protein but contains several regulatory elements, including polyA adenylation signals and binding sites for micro‐RNAs (Tuller et al., 2009). The 5′UTR at the upstream region also harbors regulatory elements such as sequences functioning as binding sites for regulatory proteins that may affect mRNA regulation and its stability (Lin & Li, 2012; Tuller et al., 2009). Also, the presence of secondary structures, upstream start codon (AUG), and open reading frames (ORFs) in the 5′UTR region affect the overall gene transcription (Mignone et al., 2002; Tuller et al., 2009). The results of this study show that for most of the chromosomes, the average length of the 3′UTR end was overall longer than that of the 5′UTR end for both MEGs and SEGs, in agreement with previous observations that 3′UTRs in metazoans are much longer than 5′UTRs (200–800 nucleotides and 100–200 nucleotides, respectively (Mignone et al., 2002). These results may reflect an important role of 3′UTRs in the regulation of gene expression, especially at the translational level. It has been found that 3′UTR on average is longer and has evolved faster in cichlids compared with other teleosts, which might be due to their meta‐regulation and regulation roles in post‐transcriptional regulation mechanisms (Xiong et al., 2018). The present study also shows that the average length of the 5′UTR end of the SEGs was much longer than that of the MEGs, whereas the latter have longer 3′UTRs. These differences in 5′UTR and 3′UTR lengths might contribute to gene expression divergence between SEGs and their parental MEGs, and therefore lead to sub‐ or neofunctionalization of new SEGs. They may reflect the involvement of 5′UTR in mechanisms governing transcriptional regulation. Indeed, the longer 5′UTRs of SEGs may reflect lower translation rates compared with MEGs, in agreement with previous findings that mRNAs with high translation rates often contain short 5′UTRs (Larsen & Michael, 2014).

The promoter sequences are less conserved than the coding regions (Chiba et al., 2008; Hemberg et al., 2012), which implies that high similarity of promoter regions between SEGs and their MEG parents could be indicative of their involvement in common regulatory networks. In this study, the comparison of 1,000 bp upstream sequence, potentially containing the promoter motifs, indicated that 34 SEG/MEG parents share high similarity. The search for conserved motifs in this region indicated that 15 SEG promoter sequences have an equivalent with strongly conserved motif signals in the same genome. By contrast, the comparison of regions potentially harboring promoter sequences of the 312 MEGs with SEG paralogs failed to identify MEGs that share common promoters with them. These results suggest that promoter sequences evolve in a manner that is closely linked to the genes they control. It also implies that there is little or no identifiable promoter similarity between more distantly related genes. Overall, these results indicate that gene retrotransposition, which is presumably followed by an insertion of a retrotranscript elsewhere in the genome, is likely accompanied by substantial changes in the promoters. This is interesting but not entirely surprising since following retrotransposition, it is more likely that an active retrogene is inserted next to the parental gene with which it can share a common promoter (Fablet et al., 2009). This is consistent with the observation that most SEGs with a common MEG promoter identified in this study are nested genes.

The SEG/MEG pairs that share motif characteristics of functional promoter regions are probably under the influence of common regulatory networks. It has been demonstrated that paralog genes that display high similarities in their promoter regions are likely to be involved in the same physiological pathways (Huning & Kunkel, 2020). The results of the current study thus support previous findings that SEGs in eukaryotic organisms are genes that are just as functional as are MEGs, thus providing strong evidence that they may play crucial roles in the genome. Indeed, depending on their occurrence and frequency, retrogenes may contribute considerably to the diversification of genomes and may therefore be responsible for the emergence of species‐specific features (Kubiak & Makałowska 2017). It can be, therefore, speculated that retrogenes might be involved in specific adaptive processes in many organisms, including teleosts. The alignments produced by the method used, which combined two different approaches, reflect *bona fide* functional sequence rather than background synteny. However, the low number of SEG/MEG parents with common promoter motifs found in this study indicates that few SEGs in the genome are under the influence of the same regulatory networks. This can also be explained by a failure of the approach used to identify promoter motifs. It is possible that the promoters of some SEG/MEG parents are by chance not contained in the 1,000 bp upstream of the coding sequence. It has been demonstrated that some promoters occur several kb upstream of the transcription factor‐binding sites (Khambata‐Ford et al., 2003). By extending the search several kb upstream of the coding sequences, it may be possible to find additional SEG and MEG parents that share common promoter motifs. Although this study allowed identifying SEG and MEG parents that share common promoter motifs, further experimental evidence is needed to confirm that they are under the regulatory control of the same promoter.

A significant number of SEGs identified in the European sea bass genome have MEG paralogs either on the same or on different chromosomes, suggesting that these might originate from LINE1 retrotransposon‐mediated reverse transcription of mRNA from “parental” source genes (Brosius, 1991; Long, 2002). Given that the probability for an inserted retrocopy to meet a functional regulatory element that could promote its transcription is higher in genomes with higher gene density, it can be expected that genomic regions with more coding sequences harbor more potentially functional SEGs. However, the results of this study revealed that the fraction of SEGs is not significantly correlated with gene density at the chromosomal level, in disagreement with the above assumption. This may be explained by the fact that the SEG content in the genome is also dependent on L1 element activity (Seleme et al., 2006), which is related to the chromatin status in which these elements are located in the genome.

The fraction of SEGs found in the European sea bass genome in this study showed significant differences between chromosomes. If retrotransposition is the main mechanism generating SEGs, these results may indicate that differences in the frequency of gene retrotransposition activity may have occurred between chromosomes. The proportion of SEGs in the European sea bass genome was slightly higher than previously reported in other teleost fishes (Tine et al., 2011), which may be explained by the fact that most of SEGs found on different chromosomes in this study were not considered to be duplicates, but of different origin and/or location. The evolutionary analyses based on the estimation of nonsynonymous to synonymous ratios showed that 33 SEG/SEG paralogs have an average Ka/Ks of 1.01 (*p* = .0307), suggesting that they are under positive selection. These SEG/SEG paralogs under positive selection may have different functions, despite not being sufficiently divergent at the nucleotide sequence level. A large proportion of these SEGs of European sea bass are nested genes (i.e., they overlap with the MEG parent), which may explain why they are under positive selection. These nested SEGs have the same gene name as the host MEG paralog (Appendix S2) and may have originated from retroduplication from their host MEGs. It has been demonstrated that duplicated paralogs evolve faster than paralogs with similar levels of divergence and similar function (Kondrashov et al., 2002). The proportion of SEGs (8.3%) found in the European sea bass genome is lower than the fraction reported in other vertebrates, including human (12.3%) and mouse (15.8%) (Sakharkar et al., 2006) and in plants (rice: 19.9%; *Arabidopsis*: 21.7%) (Jain et al., 2008), which may reflect less retrotransposition activity in fish genomes compared with other vertebrates such as human, chimp, dog, cow, rat, and mouse, as previously demonstrated for *Tetraodon nigroviridis* (Yu et al., 2007). About 1% of the genome of this species consists of retrotransposons (Roest et al., 2000), implying a lower frequency of retrotransposition events in its genome. This might be a feature common to teleost fishes, as is evident from the lower fraction of SEGs found in this lineage compared with those previously reported in vertebrates and plants.

## 5 CONCLUSION

This study showed that a large proportion (8.3%) of the European sea bass genes are SEGs. The proportion of SEGs in the European sea bass genome is highly correlated with the number of MEGs, suggesting that SEGs are continually being created by retrotransposition events. This is supported by the significant number of SEGs with a parental MEG in the genome. A significant number of SEGs showed high similarity in the promoter region with their MEG paralogs, which implies that both have the same biological function. The present study is the first to illustrate that some SEGs have conserved features in their promoter regions that are shared with their MEG parents, while others did not conserve motif characteristics of regulatory regions with their MEG parents. The results suggest that certain SEGs have evolved new functions after their genesis by natural selection that has acted on their promoter regions, especially for those with promoter sequences that are dissimilar from the promoter sequences of their potential MEG parents.

## CONFLICT OF INTEREST

Eventual conflicts of interest (including personal communications or additional permissions, related manuscripts), sources of financial support, corporate involvement, and patent holdings are disclosed.

## AUTHOR CONTRIBUTIONS

**Mbaye Tine:** Investigation (lead); writing‐original draft (lead). **Heiner Kuhl:** Formal analysis (supporting); methodology (supporting); supervision (supporting); writing‐review & editing (supporting). **Peter R. Teske:** Formal analysis (supporting); writing‐review & editing (supporting). **Richard Reinhardt:** Funding acquisition (lead); project administration (lead); supervision (equal).

## Supporting information

Appendix S1

Appendix S2

Appendix S3

## Data Availability

All datasets supporting the results of this article are included within the article and its Additional Files. This study was based on European sea bass genome resource data available at the following link: http://seabass.mpipz.de/index.html?org=European+seabass&db=dicLab1&hgsid=1895.
