# Supplementary figures and images for: Genome‐wide analysis of European sea bass provides insights into the evolution and functions of single‐exon genes
Source: Ecol Evol. 2021 Apr 2;11(11):6546–57. doi: 10.1002/ece3.7507 (PMC8207432; doi:10.1002/ece3.7507)

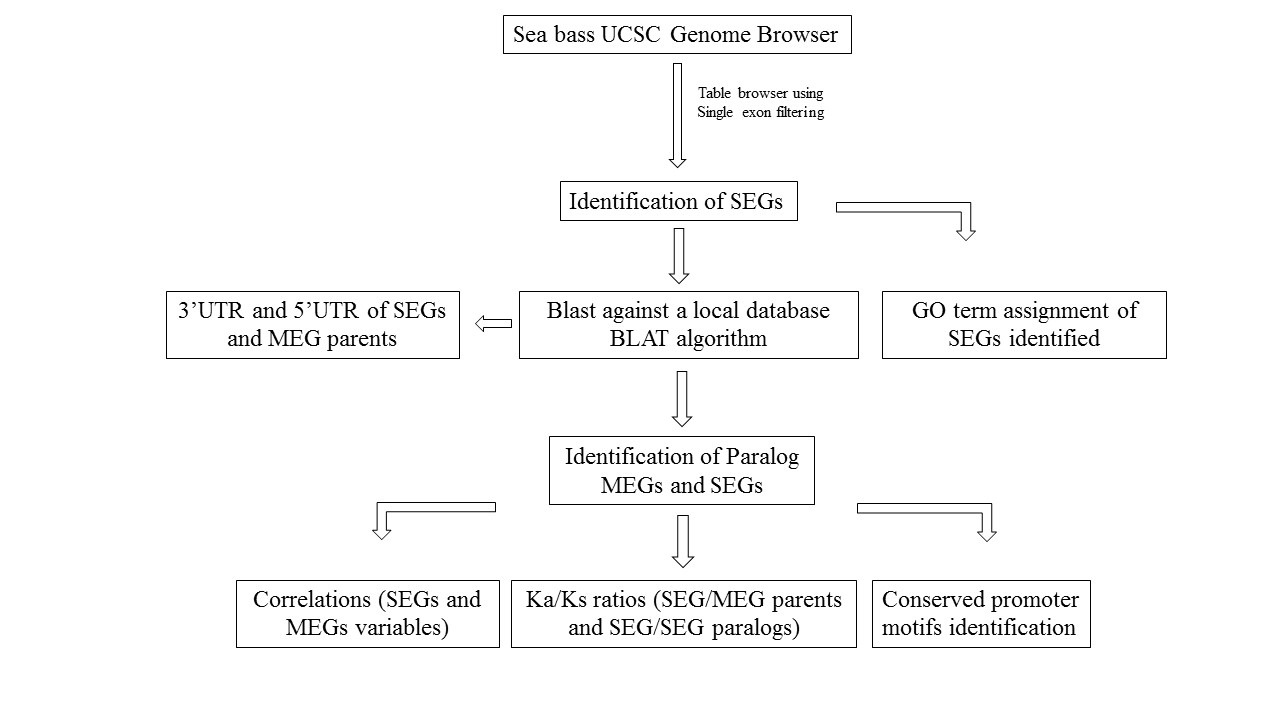


Supplementary File 1: Overview of the pipeline analysis

Supplement: Supplementary file 1 — Appendix S1 [file ECE3-11-6546-s002.docx]
