# Supplementary material for: Genome‐wide analysis of European sea bass provides insights into the evolution and functions of single‐exon genes
Source: Ecol Evol. 2021 Apr 2;11(11):6546–57. doi: 10.1002/ece3.7507 (PMC8207432; doi:10.1002/ece3.7507)
Supplement: Supplementary file 2 — Appendix S2 [file ECE3-11-6546-s003.docx]

Table of mutiple exon genes (MEG) and single exon genes (SEG) that were used for the correlations

| MEG name | Chromosomal location | SEG name | Chromosomal location | KaKs >1 | Protein name |
| --- | --- | --- | --- | --- | --- |
| DLA_LG1A_001660 | LG1A | DLAgn_00087600 | LG1A |  | amphoterin-induced protein 3 |
| DLA_LG1A_000060 | LG1A | DLAgn_00087610 | LG1A | * | transposase |
| DLA_LG1A_000330 | LG1A | DLAgn_00087650 | LG1A |  | low quality protein: zinc finger protein 699 |
| DLA_LG1A_004230 | LG1A | DLAgn_00087670 | LG1A | * | reverse transcriptase-like protein |
| DLA_LG1A_006580 | LG1A | DLAgn_00087700 | LG1A |  | NA |
| DLA_LG1A_006860 | LG1A | DLAgn_00087860 | LG1A |  | NA |
| DLA_LG1A_008900 | LG1A | DLAgn_00088000 | LG1A |  | uncharacterized protein |
| DLA_LG1A_000030 | LG1A | DLAgn_00088210 | LG1A |  | p2y purinoceptor 1-like |
| DLA_LG1A_000090 | LG1A | DLAgn_00088350 | LG1A |  | lipoma hmgic fusion partner-like 4 protein |
| DLA_LG1A_000200 | LG1A | DLAgn_00088860 | LG1A |  | protein phosphatase 1 regulatory subunit 3d-like |
| DLA_LG1A_000220 | LG1A | DLAgn_00089080 | LG1A |  | probable g-protein coupled receptor 173-like |
| DLA_LG1A_000235 | LG1A | DLAgn_00089190 | LG1A |  | leucine-rich repeat neuronal protein 1 |
| DLA_LG1A_000390 | LG1A | DLAgn_00089460 | LG1A |  | transcription factor |
| DLA_LG1A_000460 | LG1A | DLAgn_00089610 | LG1A |  | unnamed protein product [Tetraodon nigroviridis] |
| DLA_LG1A_000490 | LG1A | DLAgn_00089620 | LG1A |  | dna-binding protein inhibitor id-1 |
| DLA_LG1A_000540 | LG1A | DLAgn_00089760 | LG1A |  | NA |
| DLA_LG1A_000630 | LG1A | DLAgn_00090120 | LG1A |  | NA |
| DLA_LG1A_000750 | LG1A | DLAgn_00090140 | LG1A |  | membrane-associated guanylate ww and pdz domain-containing protein 1-like |
| DLA_LG1A_000800 | LG1A | DLAgn_00090150 | LG1A |  | NA |
| DLA_LG1A_000830 | LG1A | DLAgn_00090290 | LG1A |  | d dopamine receptor |
| DLA_LG1A_000970 | LG1A | DLAgn_00090400 | LG1A |  | globoside alpha- -n-acetylgalactosaminyltransferase 1 |
| DLA_LG1A_001030 | LG1A | DLAgn_00090690 | LG1A |  | uncharacterized protein loc101482783 isoform x2 |
| DLA_LG1A_001110 | LG1A | DLAgn_00090870 | LG1A |  | NA |
| DLA_LG1A_001150 | LG1A | DLAgn_00091060 | LG1A |  | twist-related protein 2-like |
| DLA_LG1A_001190 | LG1A | DLAgn_00091290 | LG1A |  | protein-tyrosine kinase 2-beta-like |
| DLA_LG1A_001270 | LG1A | DLAgn_00091410 | LG1A |  | uncharacterized protein loc101169743 |
| DLA_LG1A_001410 | LG1A | DLAgn_00091670 | LG1A |  | leucine-rich repeat neuronal protein 2-like |
| DLA_LG1A_001790 | LG1A | DLAgn_00091700 | LG1A |  | ammonium transporter rh type b-like |
| DLA_LG1A_001890 | LG1A | DLAgn_00091950 | LG1A |  | g-protein coupled receptor 15 |
| DLA_LG1A_002160 | LG1A | DLAgn_00092320 | LG1A |  | leucine-rich repeat neuronal protein 1-like |
| DLA_LG1A_002200 | LG1A | DLAgn_00092570 | LG1A |  | neurogenic differentiation factor 4-like |
| DLA_LG1A_002220 | LG1A | DLAgn_00092590 | LG1A |  | g-protein coupled receptor 84 |
| DLA_LG1A_002430 | LG1A | DLAgn_00093010 | LG1A |  | NA |
| DLA_LG1A_002630 | LG1A | DLAgn_00093020 | LG1A |  | probable g-protein coupled receptor 22-like |
| DLA_LG1A_002690 | LG1A | DLAgn_00093160 | LG1A |  | probable g-protein coupled receptor-like |
| DLA_LG1A_002840 | LG1A | DLAgn_00093340 | LG1A |  | chemokine-like receptor 1-like |
| DLA_LG1A_002870 | LG1A | DLAgn_00093370 | LG1A |  | pi-plc x domain-containing protein 3 |
| DLA_LG1A_002880 | LG1A | DLAgn_00094020 | LG1A |  | protein transport protein sec24b-like |
| DLA_LG1A_003260 | LG1A | DLAgn_00094160 | LG1A |  | short-chain dehydrogenase reductase family 42e member 1-like |
| DLA_LG1A_003310 | LG1A | DLAgn_00094660 | LG1A |  | transcription cofactor hes-6-like |
| DLA_LG1A_003410 | LG1A | DLAgn_00094830 | LG1A |  | a-kinase anchor protein 12 |
| DLA_LG1A_003520 | LG1A | DLAgn_00095010 | LG1A |  | piggybac transposable element-derived protein 4-like |
| DLA_LG1A_003570 | LG1A | DLAgn_00095050 | LG1A |  | polypeptide n-acetylgalactosaminyltransferase 13 |
| DLA_LG1A_003730 | LG1A | DLAgn_00095150 | LG1A |  | zinc binding alcohol dehydrogenase domain containing 2 |
| DLA_LG1A_004350 | LG1A | DLAgn_00095830 | LG1A |  | complement c1q tumor necrosis factor-related protein 3-like |
| DLA_LG1A_004410 | LG1A | DLAgn_00095900 | LG1A |  | hras-like suppressor |
| DLA_LG1A_004495 | LG1A | DLAgn_00095990 | LG1A |  | ethanolamine-phosphate phospho-lyase |
| DLA_LG1A_004495_2 | LG1A | DLAgn_00096130 | LG1A |  | mixed lineage kinase domain-like |
| DLA_LG1A_004830 | LG1A | DLAgn_00096140 | LG1A |  | dna polymerase subunit gamma-1-like |
| DLA_LG1A_004950 | LG1A | DLAgn_00096150 | LG1A |  | myelin gene regulatory factor-like |
| DLA_LG1A_004955 | LG1A | DLAgn_00096180 | LG1A |  | trf1 interacting nuclear factor 2 |
| DLA_LG1A_004970 | LG1A | DLAgn_00096540 | LG1A |  | neurobeachin isoform 2 |
| DLA_LG1A_005130 | LG1A | DLAgn_00096560 | LG1A |  | complement c2-like |
| DLA_LG1A_005240 | LG1A | DLAgn_00096590 | LG1A |  | hypoxia-inducible factor 1-alpha-like |
| DLA_LG1A_005560 | LG1A | DLAgn_00096600 | LG1A |  | serine threonine-protein kinase dclk2-like8 |
| DLA_LG1A_005830 | LG1A | DLAgn_00096790 | LG1A |  | transposable element tcb2 transposase |
| DLA_LG1A_005840 | LG1A | DLAgn_00096920 | LG1A |  | ets translocation variant 4 |
| DLA_LG1A_006010 | LG1A | DLAgn_00097160 | LG1A |  | slow myosin heavy chain 1 |
| DLA_LG1A_006080 | LG1A | DLAgn_00097210 | LG1A |  | calumenin isoform x2 |
| DLA_LG1A_006280 | LG1A | DLAgn_00097340 | LG1A |  | calcium-binding protein 39-like |
| DLA_LG1A_006640 | LG1A | DLAgn_00097380 | LG1A |  | nicotinate phosphoribosyltransferase |
| DLA_LG1A_006670 | LG1A | DLAgn_00097570 | LG1A |  | ww domain-binding protein 11 |
| DLA_LG1A_006760 | LG1A | DLAgn_00097580 | LG1A |  | thioredoxin 2 |
| DLA_LG1A_006810 | LG1A | DLAgn_00097670 | LG1A |  | phenylalanine hydroxylase |
| DLA_LG1A_006830 | LG1A | DLAgn_00097680 | LG1A |  | junctional protein associated with coronary artery disease-like |
| DLA_LG1A_006950 | LG1A | DLAgn_00097690 | LG1A |  | phospholipase delta 1b |
| DLA_LG1A_007140 | LG1A | DLAgn_00097750 | LG1A |  | cytoplasmic dynein 2 light intermediate chain 1 |
| DLA_LG1A_007280 | LG1A | DLAgn_00097900 | LG1A |  | peptidyl-prolyl cis-trans isomerase g |
| DLA_LG1A_007380 | LG1A | DLAgn_00097920 | LG1A |  | disintegrin and metalloproteinase domain-containing protein 22 isoform 1 |
| DLA_LG1A_007470 | LG1A | DLAgn_00098020 | LG1A |  | potassium sodium hyperpolarization-activated cyclic nucleotide-gated channel 1 |
| DLA_LG1A_007600 | LG1A | DLAgn_00098230 | LG1B |  | NA |
| DLA_LG1A_007740 | LG1A | DLAgn_00098370 | LG1B |  | urotensin-2 receptor-like |
| DLA_LG1A_007960 | LG1A | DLAgn_00098460 | LG1B |  | somatostatin receptor type 2-like |
| DLA_LG1A_008030 | LG1A | DLAgn_00098500 | LG1B |  | NA |
| DLA_LG1A_008130 | LG1A | DLAgn_00098660 | LG1B |  | solute carrier family facilitated glucose transporter member 11-like |
| DLA_LG1A_008210 | LG1A | DLAgn_00098670 | LG1B |  | transmembrane protein 100-like |
| DLA_LG1A_008400 | LG1A | DLAgn_00098700 | LG1B |  | uncharacterized protein c10orf71 homolog |
| DLA_LG1A_008500 | LG1A | DLAgn_00099460 | LG1B |  | transmembrane protein 238-like |
| DLA_LG1A_008510 | LG1A | DLAgn_00099570 | LG1B |  | NA |
| DLA_LG1A_008540 | LG1A | DLAgn_00100030 | LG1B |  | NA |
| DLA_LG1A_008710 | LG1A | DLAgn_00100080 | LG1B |  | NA |
| DLA_LG1A_008790 | LG1A | DLAgn_00100090 | LG1B |  | NA |
| DLA_LG1A_008920 | LG1A | DLAgn_00100120 | LG1B |  | leucine-rich repeat-containing protein 3b-like |
| DLA_LG1A_009050 | LG1A | DLAgn_00100150 | LG1B |  | atp-sensitive inward rectifier potassium channel 12-like |
| DLA_LG1A_009075_2 | LG1A | DLAgn_00100290 | LG1B |  | gap junction beta-2 protein |
| DLA_LG1A_009180 | LG1A | DLAgn_00100300 | LG1B |  | zinc finger protein 668 |
| DLA_LG1A_009420 | LG1A | DLAgn_00100370 | LG1B |  | NA |
| DLA_LG1A_009430 | LG1A | DLAgn_00100560 | LG1B |  | histone -like |
| DLA_LG1A_000010 | LG1A | DLAgn_00100640 | LG1B |  | NA |
| DLA_LG1A_000035 | LG1A | DLAgn_00100850 | LG1B |  | noggin 2 |
| DLA_LG1A_000035_2 | LG1A | DLAgn_00100900 | LG1B |  | myeloid-associated differentiation marker homolog |
| DLA_LG1A_000040 | LG1A | DLAgn_00100910 | LG1B |  | myeloid-associated differentiation marker homolog |
| DLA_LG1A_000050 | LG1A | DLAgn_00101550 | LG1B |  | NA |
| DLA_LG1A_000070 | LG1A | DLAgn_00101560 | LG1B |  | NA |
| DLA_LG1A_000080 | LG1A | DLAgn_00101940 | LG1B |  | NA |
| DLA_LG1A_000100 | LG1A | DLAgn_00102150 | LG1B |  | kelch repeat and btb domain-containing protein 13-like |
| DLA_LG1A_000110 | LG1A | DLAgn_00102190 | LG1B |  | beta-1 adrenergic receptor |
| DLA_LG1A_000120 | LG1A | DLAgn_00102550 | LG1B |  | neurogenic differentiation factor 2-like |
| DLA_LG1A_000130 | LG1A | DLAgn_00102620 | LG1B |  | NA |
| DLA_LG1A_000140 | LG1A | DLAgn_00103100 | LG1B |  | carbohydrate sulfotransferase 3-like |
| DLA_LG1A_000150 | LG1A | DLAgn_00103320 | LG1B |  | NA |
| DLA_LG1A_000160 | LG1A | DLAgn_00103630 | LG1B |  | NA |
| DLA_LG1A_000165 | LG1A | DLAgn_00103710 | LG1B |  | gtpase imap family member 8-like |
| DLA_LG1A_000165_2 | LG1A | DLAgn_00103870 | LG1B |  | NA |
| DLA_LG1A_000170 | LG1A | DLAgn_00103970 | LG1B |  | cyclophilin-rna interacting protein |
| DLA_LG1A_000175 | LG1A | DLAgn_00104060 | LG1B |  | g protein-regulated inducer of neurite outgrowth 2-like |
| DLA_LG1A_000180 | LG1A | DLAgn_00104690 | LG1B |  | zinc finger protein 91-like |
| DLA_LG1A_000185 | LG1A | DLAgn_00104750 | LG1B |  | adp-ribosylation factor-like protein 4d |
| DLA_LG1A_000190 | LG1A | DLAgn_00104870 | LG1B |  | cyclin-dependent kinase 5 activator 1 |
| DLA_LG1A_000210 | LG1A | DLAgn_00105280 | LG1B |  | NA |
| DLA_LG1A_000230 | LG1A | DLAgn_00105440 | LG1B |  | c-c chemokine receptor type 7 |
| DLA_LG1A_000250 | LG1A | DLAgn_00105490 | LG1B |  | udp-glucuronosyltransferase 2b31-like |
| DLA_LG1A_000260 | LG1A | DLAgn_00105640 | LG1B |  | inward rectifier potassium channel 16-like |
| DLA_LG1A_000270 | LG1A | DLAgn_00105650 | LG1B |  | inward rectifier potassium channel 2-like |
| DLA_LG1A_000280 | LG1A | DLAgn_00105660 | LG1B |  | NA |
| DLA_LG1A_000290 | LG1A | DLAgn_00105750 | LG1B | * | e3 ubiquitin-protein ligase rnf213- partial |
| DLA_LG1A_000300 | LG1A | DLAgn_00105780 | LG1B |  | NA |
| DLA_LG1A_000310 | LG1A | DLAgn_00105980 | LG1B |  | protein nlrc3-like |
| DLA_LG1A_000320 | LG1A | DLAgn_00106020 | LG1B |  | NA |
| DLA_LG1A_000325 | LG1A | DLAgn_00106040 | LG1B |  | d -like dopamine receptor-like |
| DLA_LG1A_000325_2 | LG1A | DLAgn_00106090 | LG1B |  | monocyte to macrophage differentiation factor |
| DLA_LG1A_000340 | LG1A | DLAgn_00106100 | LG1B |  | transmembrane protein 100-like |
| DLA_LG1A_000350 | LG1A | DLAgn_00108500 | LG2 |  | NA |
| DLA_LG1A_000355 | LG1A | DLAgn_00113200 | LG2 | * | tripartite motif-containing protein 16-like |
| DLA_LG1A_000360 | LG1A | DLAgn_00106150 | LG2 |  | sterile alpha motif domain-containing protein 3-like |
| DLA_LG1A_000370 | LG1A | DLAgn_00106340 | LG2 |  | transposase |
| DLA_LG1A_000380 | LG1A | DLAgn_00106380 | LG2 |  | NA |
| DLA_LG1A_000385 | LG1A | DLAgn_00106390 | LG2 |  | NA |
| DLA_LG1A_000400 | LG1A | DLAgn_00106550 | LG2 |  | uncharacterized protein loc101476819 |
| DLA_LG1A_000420 | LG1A | DLAgn_00106570 | LG2 |  | fatty acid-binding liver-type-like |
| DLA_LG1A_000430 | LG1A | DLAgn_00106730 | LG2 |  | heparan sulfate glucosamine 3-o-sulfotransferase 1-like |
| DLA_LG1A_000440 | LG1A | DLAgn_00107140 | LG2 |  | NA |
| DLA_LG1A_000450 | LG1A | DLAgn_00107550 | LG2 |  | probable g-protein coupled receptor 101 |
| DLA_LG1A_000470 | LG1A | DLAgn_00107610 | LG2 |  | NA |
| DLA_LG1A_000475 | LG1A | DLAgn_00107640 | LG2 |  | NA |
| DLA_LG1A_000480 | LG1A | DLAgn_00107650 | LG2 |  | transcription factor sox-3-like |
| DLA_LG1A_000500 | LG1A | DLAgn_00107870 | LG2 |  | coiled-coil domain-containing protein 85b-like |
| DLA_LG1A_000510 | LG1A | DLAgn_00107900 | LG2 |  | forkhead box protein n3 |
| DLA_LG1A_000530 | LG1A | DLAgn_00107930 | LG2 |  | serine protease 23-like |
| DLA_LG1A_000550 | LG1A | DLAgn_00108210 | LG2 |  | heterogeneous nuclear ribonucleoprotein u-like protein 2 |
| DLA_LG1A_000560 | LG1A | DLAgn_00108510 | LG2 |  | NA |
| DLA_LG1A_000570 | LG1A | DLAgn_00109180 | LG2 |  | PREDICTED: hypothetical protein LOC100089247 [Ornithorhynchus anatinus] |
| DLA_LG1A_000580 | LG1A | DLAgn_00109200 | LG2 |  | NA |
| DLA_LG1A_000590 | LG1A | DLAgn_00109210 | LG2 |  | NA |
| DLA_LG1A_000600 | LG1A | DLAgn_00109240 | LG2 |  | protocadherin gamma-a8-like |
| DLA_LG1A_000610 | LG1A | DLAgn_00109450 | LG2 |  | zinc finger and btb domain containing 5 |
| DLA_LG1A_000620 | LG1A | DLAgn_00109770 | LG2 |  | alpha-2 adrenergic receptor-like |
| DLA_LG1A_000640 | LG1A | DLAgn_00109800 | LG2 |  | a disintegrin and metalloproteinase with thrombospondin motifs 2-like |
| DLA_LG1A_000650 | LG1A | DLAgn_00109820 | LG2 |  | a disintegrin and metalloproteinase with thrombospondin motifs 2 |
| DLA_LG1A_000660 | LG1A | DLAgn_00109830 | LG2 |  | uncharacterized protein loc101478474 |
| DLA_LG1A_000670 | LG1A | DLAgn_00109900 | LG2 |  | transmembrane protein 126a |
| DLA_LG1A_000680 | LG1A | DLAgn_00110030 | LG2 | * | sterile alpha motif domain-containing protein 3-like |
| DLA_LG1A_000690 | LG1A | DLAgn_00110330 | LG2 |  | glucocorticoid receptor |
| DLA_LG1A_000700 | LG1A | DLAgn_00110550 | LG2 |  | mitochondrial carrier triple repeat protein 6-like |
| DLA_LG1A_000720 | LG1A | DLAgn_00110770 | LG2 |  | gap junction alpha-3 |
| DLA_LG1A_000730 | LG1A | DLAgn_00111000 | LG2 |  | histone-lysine n-methyltransferase mll2 |
| DLA_LG1A_000740 | LG1A | DLAgn_00111140 | LG2 |  | leucine-rich repeat transmembrane neuronal protein 2 |
| DLA_LG1A_000760 | LG1A | DLAgn_00111220 | LG2 |  | d dopamine receptor-like |
| DLA_LG1A_000770 | LG1A | DLAgn_00111310 | LG2 |  | probable g-protein coupled receptor 151-like |
| DLA_LG1A_000780 | LG1A | DLAgn_00111420 | LG2 |  | NA |
| DLA_LG1A_000790 | LG1A | DLAgn_00111560 | LG2 |  | NA |
| DLA_LG1A_000810 | LG1A | DLAgn_00111600 | LG2 |  | NA |
| DLA_LG1A_000815 | LG1A | DLAgn_00112070 | LG2 |  | NA |
| DLA_LG1A_000820 | LG1A | DLAgn_00112340 | LG2 |  | transcriptional activator protein pur-alpha-like |
| DLA_LG1A_000840 | LG1A | DLAgn_00112360 | LG2 |  | udp- c:betagal beta- -n-acetylglucosaminyltransferase 2-like |
| DLA_LG1A_000860 | LG1A | DLAgn_00112810 | LG2 |  | slit and ntrk-like protein 4-like |
| DLA_LG1A_000870 | LG1A | DLAgn_00112960 | LG2 |  | NA |
| DLA_LG1A_000880 | LG1A | DLAgn_00112970 | LG2 |  | uncharacterized protein partial |
| DLA_LG1A_000890 | LG1A | DLAgn_00113000 | LG2 |  | uncharacterized protein partial |
| DLA_LG1A_000900 | LG1A | DLAgn_00113050 | LG2 |  | NA |
| DLA_LG1A_000910 | LG1A | DLAgn_00113060 | LG2 |  | protocadherin-1-like isoform x1 |
| DLA_LG1A_000920 | LG1A | DLAgn_00113130 | LG2 | * | nucleolar complex protein 3 partial |
| DLA_LG1A_000930 | LG1A | DLAgn_00113270 | LG2 | * | NA |
| DLA_LG1A_000940 | LG1A | DLAgn_00113300 | LG2 |  | reverse transcriptase |
| DLA_LG1A_000950 | LG1A | DLAgn_00113330 | LG2 |  | protein nlrc3-like |
| DLA_LG1A_000960 | LG1A | DLAgn_00113440 | LG2 |  | NA |
| DLA_LG1A_000980 | LG1A | DLAgn_00113470 | LG2 |  | NA |
| DLA_LG1A_001000 | LG1A | DLAgn_00113510 | LG2 |  | NA |
| DLA_LG1A_001010 | LG1A | DLAgn_00113520 | LG2 |  | NA |
| DLA_LG1A_001020 | LG1A | DLAgn_00113590 | LG2 |  | NA |
| DLA_LG1A_001040 | LG1A | DLAgn_00113670 | LG2 |  | snrna-activating protein complex subunit 1-like |
| DLA_LG1A_001050 | LG1A | DLAgn_00113710 | LG2 |  | NA |
| DLA_LG1A_001055 | LG1A | DLAgn_00113790 | LG2 |  | bardet-biedl syndrome 12 protein |
| DLA_LG1A_001060 | LG1A | DLAgn_00113870 | LG2 |  | nanos homolog 1-like |
| DLA_LG1A_001070 | LG1A | DLAgn_00113960 | LG2 |  | fibroblast growth factor-binding protein 1 |
| DLA_LG1A_001075 | LG1A | DLAgn_00113970 | LG2 |  | fibroblast growth factor-binding protein 2-like |
| DLA_LG1A_001080 | LG1A | DLAgn_00114020 | LG2 |  | NA |
| DLA_LG1A_001090 | LG1A | DLAgn_00114070 | LG2 |  | transposase |
| DLA_LG1A_001100 | LG1A | DLAgn_00114300 | LG2 |  | leucine-rich repeat and immunoglobulin-like domain-containing nogo receptor-interacting protein 2-like |
| DLA_LG1A_001120 | LG1A | DLAgn_00140590 | LG3 |  | transposase |
| DLA_LG1A_001130 | LG1A | DLAgn_00140630 | LG3 | * | transposable element tcb1 transposase |
| DLA_LG1A_001140 | LG1A | DLAgn_00140720 | LG3 |  | NA |
| DLA_LG1A_001160 | LG1A | DLAgn_00140730 | LG3 |  | NA |
| DLA_LG1A_001170 | LG1A | DLAgn_00140750 | LG3 |  | p2y purinoceptor 4-like |
| DLA_LG1A_001180 | LG1A | DLAgn_00140840 | LG3 |  | line-1 type transposase domain-containing protein 1 |
| DLA_LG1A_001200 | LG1A | DLAgn_00140850 | LG3 |  | uncharacterized protein loc100490320 |
| DLA_LG1A_001220 | LG1A | DLAgn_00140900 | LG3 |  | endosialin-like |
| DLA_LG1A_001230 | LG1A | DLAgn_00140960 | LG3 |  | unnamed protein product [Tetraodon nigroviridis] |
| DLA_LG1A_001235 | LG1A | DLAgn_00141030 | LG3 |  | NA |
| DLA_LG1A_001240 | LG1A | DLAgn_00141170 | LG3 |  | NA |
| DLA_LG1A_001250 | LG1A | DLAgn_00141250 | LG3 | * | nuclear factor ovary-like |
| DLA_LG1A_001260 | LG1A | DLAgn_00141300 | LG3 |  | NA |
| DLA_LG1A_001280 | LG1A | DLAgn_00141330 | LG3 | * | reverse transcriptase-like protein |
| DLA_LG1A_001290 | LG1A | DLAgn_00141350 | LG3 |  | progestin and adipoq receptor family member 9 |
| DLA_LG1A_001300 | LG1A | DLAgn_00141380 | LG3 |  | NA |
| DLA_LG1A_001320 | LG1A | DLAgn_00141470 | LG3 |  | reverse transcriptase-like protein |
| DLA_LG1A_001330 | LG1A | DLAgn_00141600 | LG3 |  | upf0696 protein c11orf68 homolog |
| DLA_LG1A_001340 | LG1A | DLAgn_00141630 | LG3 |  | transposable element tcb2 transposase |
| DLA_LG1A_001350 | LG1A | DLAgn_00141830 | LG3 |  | NA |
| DLA_LG1A_001360 | LG1A | DLAgn_00141910 | LG3 |  | prostaglandin d2 receptor 2 |
| DLA_LG1A_001370 | LG1A | DLAgn_00142030 | LG3 |  | NA |
| DLA_LG1A_001375 | LG1A | DLAgn_00142050 | LG3 |  | interferon-induced very large gtpase 1-like |
| DLA_LG1A_001380 | LG1A | DLAgn_00142080 | LG3 |  | ufm1-specific protease 1-like |
| DLA_LG1A_001390 | LG1A | DLAgn_00142160 | LG3 |  | NA |
| DLA_LG1A_001400 | LG1A | DLAgn_00142430 | LG3 |  | NA |
| DLA_LG1A_001420 | LG1A | DLAgn_00142610 | LG3 |  | NA |
| DLA_LG1A_001430 | LG1A | DLAgn_00143210 | LG3 |  | NA |
| DLA_LG1A_001440 | LG1A | DLAgn_00143330 | LG3 |  | alpha-2 adrenergic receptor-like |
| DLA_LG1A_001450 | LG1A | DLAgn_00143490 | LG3 |  | NA |
| DLA_LG1A_001460 | LG1A | DLAgn_00143720 | LG3 |  | probable g-protein coupled receptor 148-like |
| DLA_LG1A_001470 | LG1A | DLAgn_00144160 | LG3 | * | uncharacterized protein loc101155885 |
| DLA_LG1A_001480 | LG1A | DLAgn_00144280 | LG3 |  | NA |
| DLA_LG1A_001500 | LG1A | DLAgn_00144360 | LG3 |  | zinc fingers and homeoboxes protein 3-like |
| DLA_LG1A_001510 | LG1A | DLAgn_00144400 | LG3 | * | e3 ubiquitin isg15 ligase trim25-like |
| DLA_LG1A_001520 | LG1A | DLAgn_00144550 | LG3 |  | NA |
| DLA_LG1A_001530 | LG1A | DLAgn_00144820 | LG3 |  | NA |
| DLA_LG1A_001535 | LG1A | DLAgn_00149430 | LG4 |  | uncharacterized protein loc101483012 |
| DLA_LG1A_001540 | LG1A | DLAgn_00144990 | LG4 |  | NA |
| DLA_LG1A_001550 | LG1A | DLAgn_00145240 | LG4 |  | transcription factor -like |
| DLA_LG1A_001560 | LG1A | DLAgn_00145330 | LG4 |  | NA |
| DLA_LG1A_001570 | LG1A | DLAgn_00145420 | LG4 |  | NA |
| DLA_LG1A_001590 | LG1A | DLAgn_00145560 | LG4 |  | forkhead box protein c1 |
| DLA_LG1A_001620 | LG1A | DLAgn_00145660 | LG4 |  | NA |
| DLA_LG1A_001630 | LG1A | DLAgn_00145920 | LG4 |  | NA |
| DLA_LG1A_001640 | LG1A | DLAgn_00145940 | LG4 |  | transcription factor sox-2-like |
| DLA_LG1A_001650 | LG1A | DLAgn_00146360 | LG4 |  | NA |
| DLA_LG1A_001670 | LG1A | DLAgn_00146560 | LG4 |  | forkhead box d3 |
| DLA_LG1A_001680 | LG1A | DLAgn_00146600 | LG4 |  | NA |
| DLA_LG1A_001690 | LG1A | DLAgn_00146640 | LG4 |  | NA |
| DLA_LG1A_001700 | LG1A | DLAgn_00147230 | LG4 |  | probable g-protein coupled receptor 52 |
| DLA_LG1A_001710 | LG1A | DLAgn_00147360 | LG4 |  | transcription factor jun-d-like |
| DLA_LG1A_001720 | LG1A | DLAgn_00147560 | LG4 | * | immunoglobulin light chain precursor |
| DLA_LG1A_001730 | LG1A | DLAgn_00147690 | LG4 |  | protein nlrc3-like |
| DLA_LG1A_001740 | LG1A | DLAgn_00147700 | LG4 | * | protein nlrc3-like |
| DLA_LG1A_001750 | LG1A | DLAgn_00147830 | LG4 |  | unnamed protein product [Tetraodon nigroviridis] |
| DLA_LG1A_001760 | LG1A | DLAgn_00148070 | LG4 |  | NA |
| DLA_LG1A_001770 | LG1A | DLAgn_00148130 | LG4 |  | NA |
| DLA_LG1A_001780 | LG1A | DLAgn_00148280 | LG4 |  | NA |
| DLA_LG1A_001800 | LG1A | DLAgn_00148820 | LG4 |  | NA |
| DLA_LG1A_001810 | LG1A | DLAgn_00148880 | LG4 |  | NA |
| DLA_LG1A_001820 | LG1A | DLAgn_00149090 | LG4 |  | cortexin-1 isoform x1 |
| DLA_LG1A_001830 | LG1A | DLAgn_00149180 | LG4 |  | NA |
| DLA_LG1A_001840 | LG1A | DLAgn_00149490 | LG4 |  | NA |
| DLA_LG1A_001850 | LG1A | DLAgn_00149790 | LG4 |  | NA |
| DLA_LG1A_001860 | LG1A | DLAgn_00149850 | LG4 |  | fumarate mitochondrial-like |
| DLA_LG1A_001880 | LG1A | DLAgn_00149940 | LG4 |  | NA |
| DLA_LG1A_001900 | LG1A | DLAgn_00150070 | LG4 |  | forkhead box protein e4-like |
| DLA_LG1A_001910 | LG1A | DLAgn_00150080 | LG4 |  | forkhead box d1 |
| DLA_LG1A_001920 | LG1A | DLAgn_00150260 | LG4 |  | NA |
| DLA_LG1A_001930 | LG1A | DLAgn_00150440 | LG4 |  | fucose-1-phosphate guanylyltransferase |
| DLA_LG1A_001940 | LG1A | DLAgn_00150750 | LG4 |  | NA |
| DLA_LG1A_001950 | LG1A | DLAgn_00150840 | LG4 |  | leucine-rich repeat and immunoglobulin-like domain-containing nogo receptor-interacting protein 3-like |
| DLA_LG1A_001960 | LG1A | DLAgn_00150850 | LG4 |  | nuclear factor interleukin-3-regulated protein |
| DLA_LG1A_001970 | LG1A | DLAgn_00150930 | LG4 |  | NA |
| DLA_LG1A_001980 | LG1A | DLAgn_00151010 | LG4 |  | relaxin-3 receptor 1-like |
| DLA_LG1A_001990 | LG1A | DLAgn_00151740 | LG4 |  | NA |
| DLA_LG1A_002000 | LG1A | DLAgn_00151790 | LG4 | * | NA |
| DLA_LG1A_002010 | LG1A | DLAgn_00151910 | LG4 |  | transposable element tcb1 transposase |
| DLA_LG1A_002020 | LG1A | DLAgn_00152070 | LG4 |  | NA |
| DLA_LG1A_002030 | LG1A | DLAgn_00152840 | LG4 |  | proteasome subunit beta type-11-like |
| DLA_LG1A_002040 | LG1A | DLAgn_00152970 | LG4 |  | claudin-22-like |
| DLA_LG1A_002050 | LG1A | DLAgn_00153170 | LG4 |  | fibronectin type iii domain-containing protein 9-like |
| DLA_LG1A_002060 | LG1A | DLAgn_00153290 | LG4 |  | NA |
| DLA_LG1A_002070 | LG1A | DLAgn_00153390 | LG4 |  | c-c chemokine receptor type 4-like |
| DLA_LG1A_002080 | LG1A | DLAgn_00153410 | LG4 | * | c-c chemokine receptor type 4-like |
| DLA_LG1A_002090 | LG1A | DLAgn_00153420 | LG4 | * | junction plakoglobin |
| DLA_LG1A_002100 | LG1A | DLAgn_00153480 | LG4 |  | uracil nucleotide cysteinyl leukotriene receptor |
| DLA_LG1A_002110 | LG1A | DLAgn_00153710 | LG4 |  | transcription factor sox-14-like |
| DLA_LG1A_002120 | LG1A | DLAgn_00153890 | LG4 |  | NA |
| DLA_LG1A_002130 | LG1A | DLAgn_00154000 | LG4 |  | zinc finger protein 648 |
| DLA_LG1A_002150 | LG1A | DLAgn_00154070 | LG4 |  | protein aatf |
| DLA_LG1A_002165 | LG1A | DLAgn_00154190 | LG4 |  | uncharacterized protein loc101466409 isoform x2 |
| DLA_LG1A_002170 | LG1A | DLAgn_00154260 | LG4 |  | piggybac transposable element-derived protein 2-like |
| DLA_LG1A_002180 | LG1A | DLAgn_00154350 | LG4 |  | dentin sialophosphoprotein precursor |
| DLA_LG1A_002190 | LG1A | DLAgn_00154500 | LG4 |  | uncharacterized protein loc101162318 |
| DLA_LG1A_002210 | LG1A | DLAgn_00162820 | LG5 |  | PREDICTED: hypothetical protein LOC100700797 [Oreochromis niloticus] |
| DLA_LG1A_002230 | LG1A | DLAgn_00154690 | LG5 |  | unnamed protein product [Tetraodon nigroviridis] |
| DLA_LG1A_002240 | LG1A | DLAgn_00154740 | LG5 |  | NA |
| DLA_LG1A_002250 | LG1A | DLAgn_00154750 | LG5 |  | neural-cadherin-like |
| DLA_LG1A_002260 | LG1A | DLAgn_00154780 | LG5 |  | NA |
| DLA_LG1A_002270 | LG1A | DLAgn_00154910 | LG5 |  | NA |
| DLA_LG1A_002280 | LG1A | DLAgn_00154980 | LG5 |  | e3 ubiquitin-protein ligase rnf182-like |
| DLA_LG1A_002290 | LG1A | DLAgn_00155050 | LG5 |  | NA |
| DLA_LG1A_002295 | LG1A | DLAgn_00155530 | LG5 |  | NA |
| DLA_LG1A_002300 | LG1A | DLAgn_00155580 | LG5 |  | arylamine n- pineal gland isozyme nat-10-like |
| DLA_LG1A_002310 | LG1A | DLAgn_00155670 | LG5 |  | NA |
| DLA_LG1A_002320 | LG1A | DLAgn_00155970 | LG5 |  | udp-glucuronosyltransferase 2a1-like |
| DLA_LG1A_002330 | LG1A | DLAgn_00156400 | LG5 |  | probable atp-dependent rna helicase ddx28-like |
| DLA_LG1A_002340 | LG1A | DLAgn_00156430 | LG5 |  | leukotriene b4 receptor 1 |
| DLA_LG1A_002360 | LG1A | DLAgn_00156510 | LG5 |  | regulator of g-protein signaling 9-binding |
| DLA_LG1A_002370 | LG1A | DLAgn_00156590 | LG5 |  | NA |
| DLA_LG1A_002380 | LG1A | DLAgn_00156950 | LG5 | * | fh1 fh2 domain-containing protein 3-like isoform 1 |
| DLA_LG1A_002390 | LG1A | DLAgn_00157300 | LG5 |  | NA |
| DLA_LG1A_002400 | LG1A | DLAgn_00157460 | LG5 |  | NA |
| DLA_LG1A_002410 | LG1A | DLAgn_00157510 | LG5 |  | protein phosphatase 1 regulatory subunit 3e |
| DLA_LG1A_002415 | LG1A | DLAgn_00157520 | LG5 |  | NA |
| DLA_LG1A_002415_2 | LG1A | DLAgn_00157610 | LG5 |  | NA |
| DLA_LG1A_002420 | LG1A | DLAgn_00157670 | LG5 |  | melanocortin 1 receptor |
| DLA_LG1A_002425 | LG1A | DLAgn_00157920 | LG5 |  | NA |
| DLA_LG1A_002450 | LG1A | DLAgn_00157930 | LG5 |  | thap domain-containing protein 11 |
| DLA_LG1A_002460 | LG1A | DLAgn_00158590 | LG5 |  | ras association domain-containing protein 10-like |
| DLA_LG1A_002470 | LG1A | DLAgn_00158890 | LG5 |  | calcitonin gene-related peptide precursor |
| DLA_LG1A_002480 | LG1A | DLAgn_00158900 | LG5 |  | uncharacterized protein partial |
| DLA_LG1A_002490 | LG1A | DLAgn_00159110 | LG5 |  | NA |
| DLA_LG1A_002500 | LG1A | DLAgn_00159470 | LG5 |  | unnamed protein product [Tetraodon nigroviridis] |
| DLA_LG1A_002510 | LG1A | DLAgn_00159660 | LG5 |  | btb poz domain-containing protein kctd12-like |
| DLA_LG1A_002520 | LG1A | DLAgn_00159750 | LG5 |  | mitochondrial ribonuclease p protein 1-like |
| DLA_LG1A_002530 | LG1A | DLAgn_00159990 | LG5 |  | protein diaphanous homolog 3 |
| DLA_LG1A_002540 | LG1A | DLAgn_00160710 | LG5 |  | e3 ubiquitin-protein ligase rnf128-like |
| DLA_LG1A_002550 | LG1A | DLAgn_00160740 | LG5 |  | cortexin-2 isoform x1 |
| DLA_LG1A_002560 | LG1A | DLAgn_00160750 | LG5 |  | NA |
| DLA_LG1A_002570 | LG1A | DLAgn_00161020 | LG5 |  | relaxin-3 receptor 1-like |
| DLA_LG1A_002580 | LG1A | DLAgn_00161120 | LG5 |  | relaxin-3 receptor 1-like |
| DLA_LG1A_002590 | LG1A | DLAgn_00161250 | LG5 |  | NA |
| DLA_LG1A_002600 | LG1A | DLAgn_00161280 | LG5 |  | NA |
| DLA_LG1A_002605 | LG1A | DLAgn_00161430 | LG5 |  | NA |
| DLA_LG1A_002610 | LG1A | DLAgn_00161440 | LG5 |  | potassium voltage-gated channel subfamily a member 4-like |
| DLA_LG1A_002640 | LG1A | DLAgn_00161500 | LG5 |  | fin bud initiation factor homolog |
| DLA_LG1A_002650 | LG1A | DLAgn_00161530 | LG5 |  | leucine-rich repeat and immunoglobulin-like domain-containing nogo receptor-interacting protein 1-b-like |
| DLA_LG1A_002660 | LG1A | DLAgn_00161600 | LG5 |  | leucine-rich repeat-containing protein 4c-like |
| DLA_LG1A_002670 | LG1A | DLAgn_00161650 | LG5 |  | carbohydrate sulfotransferase 1 |
| DLA_LG1A_002680 | LG1A | DLAgn_00161660 | LG5 |  | NA |
| DLA_LG1A_002700 | LG1A | DLAgn_00161830 | LG5 |  | NA |
| DLA_LG1A_002710 | LG1A | DLAgn_00161850 | LG5 |  | NA |
| DLA_LG1A_002730 | LG1A | DLAgn_00161930 | LG5 |  | suppressor of cytokine signaling 4 |
| DLA_LG1A_002740 | LG1A | DLAgn_00162060 | LG5 |  | mucin-5ac-like isoform x1 |
| DLA_LG1A_002750 | LG1A | DLAgn_00162810 | LG5 |  | interferon-induced very large gtpase 1-like |
| DLA_LG1A_002760 | LG1A | DLAgn_00162920 | LG5 |  | NA |
| DLA_LG1A_002780 | LG1A | DLAgn_00163020 | LG5 |  | forkhead box protein b1-like |
| DLA_LG1A_002790 | LG1A | DLAgn_00163060 | LG5 | * | reverse transcriptase-like protein |
| DLA_LG1A_002800 | LG1A | DLAgn_00163180 | LG5 |  | stonustoxin subunit alpha-like |
| DLA_LG1A_002810 | LG1A | DLAgn_00163260 | LG5 |  | ankyrin repeat and sterile alpha motif domain-containing protein 1b-like |
| DLA_LG1A_002820 | LG1A | DLAgn_00163320 | LG5 |  | kelch repeat and btb domain-containing protein 13-like |
| DLA_LG1A_002825 | LG1A | DLAgn_00163340 | LG5 |  | ras-like protein family member 12-like |
| DLA_LG1A_002830 | LG1A | DLAgn_00163400 | LG5 |  | meprin a subunit beta-like |
| DLA_LG1A_002850 | LG1A | DLAgn_00163600 | LG5 |  | transposable element tcb1 transposase |
| DLA_LG1A_002860 | LG1A | DLAgn_00163650 | LG5 |  | ac transposable element derived 3 |
| DLA_LG1A_002890 | LG1A | DLAgn_00163670 | LG5 |  | transposase |
| DLA_LG1A_002910 | LG1A | DLAgn_00163730 | LG5 |  | gap junction delta-2 |
| DLA_LG1A_002930 | LG1A | DLAgn_00163780 | LG5 |  | general transcription factor ii-i repeat domain-containing protein 2-like |
| DLA_LG1A_002940 | LG1A | DLAgn_00163870 | LG5 |  | mesoderm development candidate 1 |
| DLA_LG1A_002950 | LG1A | DLAgn_00170510 | LG6 |  | NA |
| DLA_LG1A_002960 | LG1A | DLAgn_00164140 | LG6 |  | relaxin-3 receptor 1-like |
| DLA_LG1A_002970 | LG1A | DLAgn_00164180 | LG6 |  | e3 ubiquitin-protein ligase trim21-like |
| DLA_LG1A_002980 | LG1A | DLAgn_00164200 | LG6 |  | vacuolar protein sorting 52 |
| DLA_LG1A_002990 | LG1A | DLAgn_00164250 | LG6 |  | leucine-rich repeat neuronal protein 3-like |
| DLA_LG1A_003000 | LG1A | DLAgn_00164310 | LG6 |  | e3 ubiquitin-protein ligase trim21-like |
| DLA_LG1A_003010 | LG1A | DLAgn_00164320 | LG6 | * | e3 ubiquitin-protein ligase trim21-like |
| DLA_LG1A_003020 | LG1A | DLAgn_00164380 | LG6 |  | NA |
| DLA_LG1A_003040 | LG1A | DLAgn_00164400 | LG6 |  | mixed lineage kinase domain-like |
| DLA_LG1A_003050 | LG1A | DLAgn_00164540 | LG6 |  | NA |
| DLA_LG1A_003060 | LG1A | DLAgn_00164560 | LG6 |  | uncharacterized protein c11orf89 |
| DLA_LG1A_003080 | LG1A | DLAgn_00164720 | LG6 |  | chk1 checkpoint-like protein |
| DLA_LG1A_003090 | LG1A | DLAgn_00164730 | LG6 |  | NA |
| DLA_LG1A_003100 | LG1A | DLAgn_00164760 | LG6 |  | sterile alpha motif domain-containing protein 3-like |
| DLA_LG1A_003110 | LG1A | DLAgn_00164890 | LG6 |  | upf0428 protein cxorf56 homolog |
| DLA_LG1A_003120 | LG1A | DLAgn_00165040 | LG6 |  | NA |
| DLA_LG1A_003130 | LG1A | DLAgn_00165470 | LG6 |  | NA |
| DLA_LG1A_003140 | LG1A | DLAgn_00165630 | LG6 |  | immunoglobulin kappa chain variable region |
| DLA_LG1A_003150 | LG1A | DLAgn_00165700 | LG6 |  | leucine-rich repeat-containing protein 10 |
| DLA_LG1A_003160 | LG1A | DLAgn_00165710 | LG6 |  | isoform cra_b |
| DLA_LG1A_003170 | LG1A | DLAgn_00165830 | LG6 |  | atp-sensitive inward rectifier potassium channel 11 |
| DLA_LG1A_003180 | LG1A | DLAgn_00165840 | LG6 |  | rCG22762 [Rattus norvegicus] |
| DLA_LG1A_003190 | LG1A | DLAgn_00165860 | LG6 |  | NA |
| DLA_LG1A_003200 | LG1A | DLAgn_00165870 | LG6 |  | NA |
| DLA_LG1A_003215 | LG1A | DLAgn_00165910 | LG6 |  | reverse transcriptase-like protein |
| DLA_LG1A_003220 | LG1A | DLAgn_00166160 | LG6 |  | achaete-scute homolog 1 |
| DLA_LG1A_003230 | LG1A | DLAgn_00166190 | LG6 |  | four jointed box 1 |
| DLA_LG1A_003250 | LG1A | DLAgn_00166250 | LG6 |  | leucine-rich repeat-containing protein 4c |
| DLA_LG1A_003290 | LG1A | DLAgn_00166260 | LG6 |  | recombination activating protein 2 |
| DLA_LG1A_003320 | LG1A | DLAgn_00166360 | LG6 |  | gdp-d-glucose phosphorylase 1-like |
| DLA_LG1A_003330 | LG1A | DLAgn_00166530 | LG6 |  | complement c1q tumor necrosis factor-related protein 4-like |
| DLA_LG1A_003340 | LG1A | DLAgn_00166840 | LG6 |  | transmembrane protein 60 |
| DLA_LG1A_003350 | LG1A | DLAgn_00166890 | LG6 | * | f-box lrr-repeat protein 14-like |
| DLA_LG1A_003360 | LG1A | DLAgn_00167290 | LG6 |  | leucine-rich repeat-containing protein 4 |
| DLA_LG1A_003370 | LG1A | DLAgn_00167920 | LG6 |  | ankyrin repeat domain-containing protein 34c-like |
| DLA_LG1A_003380 | LG1A | DLAgn_00168040 | LG6 |  | cholesterol 25-hydroxylase-like protein member 2-like |
| DLA_LG1A_003390 | LG1A | DLAgn_00168160 | LG6 |  | unnamed protein product [Tetraodon nigroviridis] |
| DLA_LG1A_003400 | LG1A | DLAgn_00168180 | LG6 |  | NA |
| DLA_LG1A_003420 | LG1A | DLAgn_00168210 | LG6 |  | forkhead box protein b1-like |
| DLA_LG1A_003430 | LG1A | DLAgn_00168230 | LG6 |  | beta- -galactosyl-o-glycosyl-glycoprotein beta- -n-acetylglucosaminyltransferase 3 |
| DLA_LG1A_003440 | LG1A | DLAgn_00168270 | LG6 |  | unnamed protein product [Tetraodon nigroviridis] |
| DLA_LG1A_003450 | LG1A | DLAgn_00168490 | LG6 |  | NA |
| DLA_LG1A_003460 | LG1A | DLAgn_00168630 | LG6 |  | NA |
| DLA_LG1A_003470 | LG1A | DLAgn_00168930 | LG6 |  | leucine-rich repeat-containing protein 10b |
| DLA_LG1A_003480 | LG1A | DLAgn_00169000 | LG6 |  | NA |
| DLA_LG1A_003500 | LG1A | DLAgn_00169190 | LG6 |  | p2y purinoceptor 4-like |
| DLA_LG1A_003510 | LG1A | DLAgn_00169260 | LG6 |  | fin bud initiation factor-like |
| DLA_LG1A_003530 | LG1A | DLAgn_00169280 | LG6 |  | NA |
| DLA_LG1A_003540 | LG1A | DLAgn_00169570 | LG6 |  | NA |
| DLA_LG1A_003550 | LG1A | DLAgn_00169930 | LG6 |  | probable g-protein coupled receptor 22-like |
| DLA_LG1A_003580 | LG1A | DLAgn_00170260 | LG6 |  | shaker-related potassium channel tsha2-like |
| DLA_LG1A_003590 | LG1A | DLAgn_00170270 | LG6 |  | potassium voltage-gated channel subfamily a member 1-like |
| DLA_LG1A_003595 | LG1A | DLAgn_00170480 | LG6 |  | t-cell surface antigen cd2 precursor |
| DLA_LG1A_003595_2 | LG1A | DLAgn_00170550 | LG6 |  | p2y purinoceptor 1 |
| DLA_LG1A_003600 | LG1A | DLAgn_00170640 | LG6 |  | NA |
| DLA_LG1A_003610 | LG1A | DLAgn_00170820 | LG6 |  | NA |
| DLA_LG1A_003620 | LG1A | DLAgn_00170830 | LG6 |  | unnamed protein product [Tetraodon nigroviridis] |
| DLA_LG1A_003630 | LG1A | DLAgn_00170910 | LG6 |  | toll-like receptor 2 type-1 |
| DLA_LG1A_003660 | LG1A | DLAgn_00170940 | LG6 |  | forkhead box d4 |
| DLA_LG1A_003680 | LG1A | DLAgn_00170950 | LG6 |  | protein phosphatase 1 regulatory subunit 3a |
| DLA_LG1A_003700 | LG1A | DLAgn_00170980 | LG6 |  | NA |
| DLA_LG1A_003710 | LG1A | DLAgn_00171120 | LG6 |  | leucine-rich repeat and immunoglobulin-like domain-containing nogo receptor-interacting protein 1-like |
| DLA_LG1A_003720 | LG1A | DLAgn_00171270 | LG6 |  | NA |
| DLA_LG1A_003740 | LG1A | DLAgn_00171480 | LG6 |  | uncharacterized protein c11orf96 homolog |
| DLA_LG1A_003750 | LG1A | DLAgn_00171950 | LG6 |  | alpha- -mannosyl-glycoprotein 4-beta-n-acetylglucosaminyltransferase c-like |
| DLA_LG1A_003760 | LG1A | DLAgn_00172110 | LG6 |  | NA |
| DLA_LG1A_003770 | LG1A | DLAgn_00172310 | LG6 |  | retrotransposable element tf2 155 kda protein type 1-like |
| DLA_LG1A_003775 | LG1A | DLAgn_00172330 | LG6 |  | NA |
| DLA_LG1A_003780 | LG1A | DLAgn_00172370 | LG6 |  | kelch repeat and btb domain-containing protein 13-like |
| DLA_LG1A_003790 | LG1A | DLAgn_00172830 | LG6 |  | phosphatidylinositol n-acetylglucosaminyltransferase subunit y |
| DLA_LG1A_003800 | LG1A | DLAgn_00172960 | LG6 |  | ras association domain-containing protein 10-like |
| DLA_LG1A_003805 | LG1A | DLAgn_00173000 | LG6 |  | novel protein |
| DLA_LG1A_003820 | LG1A | DLAgn_00173240 | LG6 |  | NA |
| DLA_LG1A_003830 | LG1A | DLAgn_00173400 | LG6 | * | uncharacterized protein loc101470281 |
| DLA_LG1A_003840 | LG1A | DLAgn_00173680 | LG6 |  | solute carrier family member 3 |
| DLA_LG1A_003850 | LG1A | DLAgn_00173850 | LG6 |  | forkhead box l1 |
| DLA_LG1A_003860 | LG1A | DLAgn_00174210 | LG6 |  | zinc finger bed domain-containing protein 4-like |
| DLA_LG1A_003870 | LG1A | DLAgn_00174230 | LG6 |  | reverse transcriptase-like protein |
| DLA_LG1A_003880 | LG1A | DLAgn_00180450 | LG7 |  | alpha-1-acid glycoprotein 1 |
| DLA_LG1A_003890 | LG1A | DLAgn_00181770 | LG7 |  | NA |
| DLA_LG1A_003900 | LG1A | DLAgn_00174440 | LG7 |  | zinc finger bed domain-containing protein 1-like |
| DLA_LG1A_003910 | LG1A | DLAgn_00174490 | LG7 |  | gtpase imap family member 4-like |
| DLA_LG1A_003930 | LG1A | DLAgn_00174500 | LG7 |  | NA |
| DLA_LG1A_003940 | LG1A | DLAgn_00174690 | LG7 |  | NA |
| DLA_LG1A_003950 | LG1A | DLAgn_00174890 | LG7 |  | NA |
| DLA_LG1A_003960 | LG1A | DLAgn_00175000 | LG7 |  | NA |
| DLA_LG1A_003970 | LG1A | DLAgn_00175050 | LG7 |  | NA |
| DLA_LG1A_003980 | LG1A | DLAgn_00175120 | LG7 |  | uncharacterized protein loc101471451 isoform x1 |
| DLA_LG1A_004000 | LG1A | DLAgn_00175200 | LG7 |  | NA |
| DLA_LG1A_004010 | LG1A | DLAgn_00175330 | LG7 |  | NA |
| DLA_LG1A_004020 | LG1A | DLAgn_00175460 | LG7 |  | zinc finger protein 572 |
| DLA_LG1A_004030 | LG1A | DLAgn_00175480 | LG7 |  | zinc finger protein 214 |
| DLA_LG1A_004040 | LG1A | DLAgn_00175490 | LG7 |  | oocyte zinc finger protein 6-like |
| DLA_LG1A_004050 | LG1A | DLAgn_00175510 | LG7 |  | zinc finger protein 235-like |
| DLA_LG1A_004060 | LG1A | DLAgn_00175520 | LG7 |  | zinc finger protein 235-like |
| DLA_LG1A_004080 | LG1A | DLAgn_00175730 | LG7 |  | junction plakoglobin |
| DLA_LG1A_004090 | LG1A | DLAgn_00175840 | LG7 |  | NA |
| DLA_LG1A_004100 | LG1A | DLAgn_00176170 | LG7 |  | transcription factor sp6-like |
| DLA_LG1A_004110 | LG1A | DLAgn_00176490 | LG7 |  | myeloid-associated differentiation marker-like |
| DLA_LG1A_004120 | LG1A | DLAgn_00176500 | LG7 |  | perforin-1-like |
| DLA_LG1A_004130 | LG1A | DLAgn_00176820 | LG7 |  | prostaglandin d2 receptor 2 |
| DLA_LG1A_004140 | LG1A | DLAgn_00177050 | LG7 |  | alpha-2 adrenergic receptor-like |
| DLA_LG1A_004150 | LG1A | DLAgn_00177120 | LG7 |  | noggin 4 precursor |
| DLA_LG1A_004155 | LG1A | DLAgn_00177150 | LG7 |  | transcription factor jun-b-like |
| DLA_LG1A_004160 | LG1A | DLAgn_00177250 | LG7 |  | NA |
| DLA_LG1A_004170 | LG1A | DLAgn_00177290 | LG7 |  | nuclear factor interleukin-3-regulated |
| DLA_LG1A_004180 | LG1A | DLAgn_00177540 | LG7 |  | NA |
| DLA_LG1A_004190 | LG1A | DLAgn_00177630 | LG7 |  | inositol -trisphosphate receptor-interacting |
| DLA_LG1A_004200 | LG1A | DLAgn_00178170 | LG7 |  | nanos homolog 3 |
| DLA_LG1A_004210 | LG1A | DLAgn_00178330 | LG7 |  | reverse transcriptase-like protein |
| DLA_LG1A_004220 | LG1A | DLAgn_00178570 | LG7 |  | tpa_exp: claudin 31 |
| DLA_LG1A_004250 | LG1A | DLAgn_00178620 | LG7 |  | PREDICTED: hypothetical protein LOC100692456 [Oreochromis niloticus] |
| DLA_LG1A_004260 | LG1A | DLAgn_00178640 | LG7 |  | NA |
| DLA_LG1A_004280 | LG1A | DLAgn_00178650 | LG7 |  | NA |
| DLA_LG1A_004285 | LG1A | DLAgn_00179070 | LG7 |  | NA |
| DLA_LG1A_004290 | LG1A | DLAgn_00179220 | LG7 |  | trace amine-associated receptor 4-like |
| DLA_LG1A_004310 | LG1A | DLAgn_00179490 | LG7 |  | ecto-adp-ribosyltransferase 5 precursor |
| DLA_LG1A_004320 | LG1A | DLAgn_00179560 | LG7 |  | histone partial |
| DLA_LG1A_004330 | LG1A | DLAgn_00179730 | LG7 |  | histone partial |
| DLA_LG1A_004340 | LG1A | DLAgn_00179820 | LG7 |  | NA |
| DLA_LG1A_004370 | LG1A | DLAgn_00180010 | LG7 |  | secretory phospholipase a2 receptor-like |
| DLA_LG1A_004380 | LG1A | DLAgn_00180030 | LG7 |  | reverse transcriptase-like protein |
| DLA_LG1A_004390 | LG1A | DLAgn_00180060 | LG7 |  | gtp-binding protein rhes-like |
| DLA_LG1A_004400 | LG1A | DLAgn_00180370 | LG7 |  | PREDICTED: hypothetical protein LOC100701921 [Oreochromis niloticus] |
| DLA_LG1A_004420 | LG1A | DLAgn_00180380 | LG7 |  | NA |
| DLA_LG1A_004430 | LG1A | DLAgn_00180390 | LG7 |  | uncharacterized protein loc100894812 |
| DLA_LG1A_004450 | LG1A | DLAgn_00180400 | LG7 |  | NA |
| DLA_LG1A_004470 | LG1A | DLAgn_00180420 | LG7 |  | NA |
| DLA_LG1A_004480 | LG1A | DLAgn_00180460 | LG7 |  | tbt-binding partial |
| DLA_LG1A_004490 | LG1A | DLAgn_00180580 | LG7 |  | NA |
| DLA_LG1A_004500 | LG1A | DLAgn_00180680 | LG7 |  | forkhead box protein d1-like |
| DLA_LG1A_004510 | LG1A | DLAgn_00180700 | LG7 |  | sphingosine 1-phosphate receptor 1-like |
| DLA_LG1A_004520 | LG1A | DLAgn_00181060 | LG7 |  | rhomboid domain-containing protein 2 |
| DLA_LG1A_004530 | LG1A | DLAgn_00181230 | LG7 |  | small integral membrane protein 18 |
| DLA_LG1A_004550 | LG1A | DLAgn_00181370 | LG7 |  | low quality protein: ankyrin-2-like |
| DLA_LG1A_004570 | LG1A | DLAgn_00181470 | LG7 |  | protein nlrc3-like |
| DLA_LG1A_004580 | LG1A | DLAgn_00181560 | LG7 |  | NA |
| DLA_LG1A_004585 | LG1A | DLAgn_00181730 | LG7 |  | d dopamine receptor-like |
| DLA_LG1A_004590 | LG1A | DLAgn_00181890 | LG7 |  | proline-rich protein 21 |
| DLA_LG1A_004600 | LG1A | DLAgn_00182040 | LG7 |  | NA |
| DLA_LG1A_004610 | LG1A | DLAgn_00182110 | LG7 |  | 5-hydroxytryptamine receptor 4-like |
| DLA_LG1A_004620 | LG1A | DLAgn_00182140 | LG7 |  | leucine-rich repeat transmembrane neuronal protein 1-like |
| DLA_LG1A_004630 | LG1A | DLAgn_00182350 | LG7 |  | uncharacterized protein loc285556 |
| DLA_LG1A_004640 | LG1A | DLAgn_00182380 | LG7 |  | extracellular superoxide dismutase |
| DLA_LG1A_004650 | LG1A | DLAgn_00183020 | LG7 |  | forkhead box protein e1 |
| DLA_LG1A_004660 | LG1A | DLAgn_00183160 | LG7 |  | g protein-regulated inducer of neurite outgrowth 3 |
| DLA_LG1A_004670 | LG1A | DLAgn_00183200 | LG7 |  | NA |
| DLA_LG1A_004680 | LG1A | DLAgn_00183240 | LG7 |  | acyl-coenzyme a thioesterase 4-like isoform x1 |
| DLA_LG1A_004690 | LG1A | DLAgn_00183250 | LG7 |  | uncharacterized protein loc101482234 |
| DLA_LG1A_004700 | LG1A | DLAgn_00183470 | LG7 |  | protein mab-21-like 2 |
| DLA_LG1A_004710 | LG1A | DLAgn_00183520 | LG7 |  | dctp pyrophosphatase 1-like |
| DLA_LG1A_004720 | LG1A | DLAgn_00183620 | LG7 |  | fibroblast growth factor-binding protein 1-like |
| DLA_LG1A_004730 | LG1A | DLAgn_00183630 | LG7 |  | fibroblast growth factor-binding protein 2-like |
| DLA_LG1A_004740 | LG1A | DLAgn_00183940 | LG7 |  | tnfaip3-interacting protein 2-like |
| DLA_LG1A_004750 | LG1A | DLAgn_00184450 | LG7 |  | perforin-1-like |
| DLA_LG1A_004760 | LG1A | DLAgn_00184580 | LG7 |  | gtp-binding protein rhes-like |
| DLA_LG1A_004770 | LG1A | DLAgn_00184620 | LG7 |  | NA |
| DLA_LG1A_004780 | LG1A | DLAgn_00184990 | LG7 |  | NA |
| DLA_LG1A_004790 | LG1A | DLAgn_00185040 | LG7 |  | NA |
| DLA_LG1A_004810 | LG1A | DLAgn_00185210 | LG7 |  | dynein heavy chain domain-containing protein 1 |
| DLA_LG1A_004820 | LG1A | DLAgn_00185360 | LG7 |  | NA |
| DLA_LG1A_004840 | LG1A | DLAgn_00185540 | LG7 |  | urotensin-2 receptor-like |
| DLA_LG1A_004850 | LG1A | DLAgn_00185630 | LG7 |  | NA |
| DLA_LG1A_004860 | LG1A | DLAgn_00185640 | LG7 |  | NA |
| DLA_LG1A_004870 | LG1A | DLAgn_00185660 | LG7 |  | immediate early response gene 2 protein |
| DLA_LG1A_004875 | LG1A | DLAgn_00185720 | LG7 |  | transcription factor iiia |
| DLA_LG1A_004880 | LG1A | DLAgn_00186410 | LG8 |  | NA |
| DLA_LG1A_004890 | LG1A | DLAgn_00186420 | LG8 |  | NA |
| DLA_LG1A_004900 | LG1A | DLAgn_00186510 | LG8 | * | atp-sensitive inward rectifier potassium channel 12-like |
| DLA_LG1A_004910 | LG1A | DLAgn_00186550 | LG8 |  | NA |
| DLA_LG1A_004920 | LG1A | DLAgn_00186770 | LG8 | * | cerebellar degeneration-related protein 2-like |
| DLA_LG1A_004930 | LG1A | DLAgn_00186870 | LG8 |  | gtp-binding protein rhes-like |
| DLA_LG1A_004960 | LG1A | DLAgn_00186980 | LG8 | * | uncharacterized protein loc101466409 isoform x1 |
| DLA_LG1A_004980 | LG1A | DLAgn_00187050 | LG8 |  | dexamethasone-induced protein homolog |
| DLA_LG1A_004990 | LG1A | DLAgn_00187070 | LG8 |  | suppressor of cytokine signaling 1 |
| DLA_LG1A_005000 | LG1A | DLAgn_00187140 | LG8 |  | NA |
| DLA_LG1A_005010 | LG1A | DLAgn_00187170 | LG8 |  | NA |
| DLA_LG1A_005020 | LG1A | DLAgn_00187950 | LG8 |  | NA |
| DLA_LG1A_005030 | LG1A | DLAgn_00187960 | LG8 |  | NA |
| DLA_LG1A_005040 | LG1A | DLAgn_00188070 | LG8 |  | uncharacterized protein loc101469015 isoform x1 |
| DLA_LG1A_005050 | LG1A | DLAgn_00188440 | LG8 |  | forkhead box protein d3-a-like |
| DLA_LG1A_005060 | LG1A | DLAgn_00188540 | LG8 |  | major facilitator superfamily domain-containing protein 6-like |
| DLA_LG1A_005075 | LG1A | DLAgn_00188570 | LG8 |  | immediate early response gene 2 protein |
| DLA_LG1A_005080 | LG1A | DLAgn_00188690 | LG8 |  | NA |
| DLA_LG1A_005090 | LG1A | DLAgn_00188820 | LG8 |  | myeloid-associated differentiation marker homolog |
| DLA_LG1A_005100 | LG1A | DLAgn_00188850 | LG8 |  | NA |
| DLA_LG1A_005110 | LG1A | DLAgn_00189020 | LG8 |  | sterile alpha motif domain-containing protein 9-like |
| DLA_LG1A_005120 | LG1A | DLAgn_00189330 | LG8 |  | zinc finger mym-type protein 1-like |
| DLA_LG1A_005140 | LG1A | DLAgn_00189480 | LG8 |  | NA |
| DLA_LG1A_005150 | LG1A | DLAgn_00189500 | LG8 |  | fumarylacetoacetate hydrolase domain-containing protein 1 |
| DLA_LG1A_005160 | LG1A | DLAgn_00189640 | LG8 |  | sterile alpha motif domain-containing protein 9-like |
| DLA_LG1A_005170 | LG1A | DLAgn_00190230 | LG8 |  | somatostatin receptor type 2-like |
| DLA_LG1A_005180 | LG1A | DLAgn_00190240 | LG8 |  | NA |
| DLA_LG1A_005190 | LG1A | DLAgn_00190330 | LG8 |  | NA |
| DLA_LG1A_005195 | LG1A | DLAgn_00190360 | LG8 |  | myeloid-associated differentiation marker-like protein 2-like |
| DLA_LG1A_005200 | LG1A | DLAgn_00190570 | LG8 |  | protein amnionless-like |
| DLA_LG1A_005210 | LG1A | DLAgn_00190630 | LG8 |  | inward rectifier potassium channel 2-like |
| DLA_LG1A_005230 | LG1A | DLAgn_00190720 | LG8 |  | transposon tx1 uncharacterized 149 kda |
| DLA_LG1A_005270 | LG1A | DLAgn_00190850 | LG8 |  | ring finger protein 222 |
| DLA_LG1A_005300 | LG1A | DLAgn_00190970 | LG8 |  | gtpase imap family member 7-like |
| DLA_LG1A_005310 | LG1A | DLAgn_00190990 | LG8 |  | tpa_exp: claudin 31 |
| DLA_LG1A_005320 | LG1A | DLAgn_00191050 | LG8 |  | g-protein coupled receptor 183-like |
| DLA_LG1A_005330 | LG1A | DLAgn_00191130 | LG8 |  | NA |
| DLA_LG1A_005340 | LG1A | DLAgn_00191960 | LG8 |  | g-protein coupled estrogen receptor 1-like |
| DLA_LG1A_005350 | LG1A | DLAgn_00192190 | LG8 |  | extracellular leucine-rich repeat and fibronectin type-iii domain-containing protein 1-like |
| DLA_LG1A_005360 | LG1A | DLAgn_00192480 | LG8 | * | frizzled homolog 2 |
| DLA_LG1A_005370 | LG1A | DLAgn_00192640 | LG8 |  | uncharacterized protein loc100505836 isoform 2 |
| DLA_LG1A_005380 | LG1A | DLAgn_00193320 | LG8 | * | zwilling a |
| DLA_LG1A_005390 | LG1A | DLAgn_00193330 | LG8 |  | zwilling a |
| DLA_LG1A_005400 | LG1A | DLAgn_00193480 | LG8 |  | NA |
| DLA_LG1A_005410 | LG1A | DLAgn_00193600 | LG8 |  | protein hexim1 |
| DLA_LG1A_005420 | LG1A | DLAgn_00193610 | LG8 |  | rho gtpase-activating protein 23-like |
| DLA_LG1A_005430 | LG1A | DLAgn_00193640 | LG8 |  | NA |
| DLA_LG1A_005440 | LG1A | DLAgn_00193710 | LG8 |  | gap junction gamma-1 |
| DLA_LG1A_005450 | LG1A | DLAgn_00193840 | LG8 |  | reprimo-like protein |
| DLA_LG1A_005460 | LG1A | DLAgn_00193890 | LG8 |  | neurogenic differentiation 2 |
| DLA_LG1A_005465 | LG1A | DLAgn_00193970 | LG8 |  | somatostatin receptor type 5-like |
| DLA_LG1A_005470 | LG1A | DLAgn_00194180 | LG8 |  | phosphoinositide-interacting protein |
| DLA_LG1A_005490 | LG1A | DLAgn_00194190 | LG8 |  | isoform cra_b |
| DLA_LG1A_005500 | LG1A | DLAgn_00194220 | LG8 |  | NA |
| DLA_LG1A_005510 | LG1A | DLAgn_00194240 | LG8 |  | monocyte to macrophage differentiation factor |
| DLA_LG1A_005520 | LG1A | DLAgn_00194270 | LG8 |  | noggin 3 |
| DLA_LG1A_005530 | LG1A | DLAgn_00194580 | LG8 |  | gig2-like protein |
| DLA_LG1A_005540 | LG1A | DLAgn_00194620 | LG8 | * | gig2-like protein |
| DLA_LG1A_005550 | LG1A | DLAgn_00194730 | LG8 | * | nuclear factor ovary-like |
| DLA_LG1A_005570 | LG1A | DLAgn_00194740 | LG8 |  | upf0722 protein c11orf88 homolog |
| DLA_LG1A_005580 | LG1A | DLAgn_00194780 | LG8 | * | nuclear factor ovary-like |
| DLA_LG1A_005590 | LG1A | DLAgn_00194800 | LG8 |  | unnamed protein product [Tetraodon nigroviridis] |
| DLA_LG1A_005600 | LG1A | DLAgn_00194820 | LG8 |  | NA |
| DLA_LG1A_005610 | LG1A | DLAgn_00194870 | LG8 | * | heat shock protein 70 |
| DLA_LG1A_005620 | LG1A | DLAgn_00194880 | LG8 |  | reverse transcriptase-like protein |
| DLA_LG1A_005630 | LG1A | DLAgn_00194920 | LG8 |  | transposon tx1 uncharacterized 149 kda |
| DLA_LG1A_005640 | LG1A | DLAgn_00194930 | LG8 |  | transposon tx1 uncharacterized 149 kda |
| DLA_LG1A_005660 | LG1A | DLAgn_00194950 | LG8 |  | NA |
| DLA_LG1A_005670 | LG1A | DLAgn_00195310 | LG8 |  | NA |
| DLA_LG1A_005680 | LG1A | DLAgn_00195350 | LG8 |  | NA |
| DLA_LG1A_005690 | LG1A | DLAgn_00195400 | LG8 |  | NA |
| DLA_LG1A_005700 | LG1A | DLAgn_00195450 | LG8 | * | NA |
| DLA_LG1A_005710 | LG1A | DLAgn_00195720 | LG8 |  | NA |
| DLA_LG1A_005715 | LG1A | DLAgn_00195980 | LG8 |  | NA |
| DLA_LG1A_005720 | LG1A | DLAgn_00196090 | LG8 |  | t-cell receptor beta chain |
| DLA_LG1A_005730 | LG1A | DLAgn_00196620 | LG8 |  | NA |
| DLA_LG1A_005740 | LG1A | DLAgn_00196810 | LG8 | * | uncharacterized protein partial |
| DLA_LG1A_005750 | LG1A | DLAgn_00197980 | LG9 |  | NA |
| DLA_LG1A_005760 | LG1A | DLAgn_00197510 | LG9 |  | NA |
| DLA_LG1A_005770 | LG1A | DLAgn_00197650 | LG9 |  | transposable element tcb1 transposase |
| DLA_LG1A_005780 | LG1A | DLAgn_00197790 | LG9 |  | NA |
| DLA_LG1A_005800 | LG1A | DLAgn_00197800 | LG9 |  | NA |
| DLA_LG1A_005810 | LG1A | DLAgn_00197880 | LG9 |  | NA |
| DLA_LG1A_005835 | LG1A | DLAgn_00197910 | LG9 |  | NA |
| DLA_LG1A_005835_2 | LG1A | DLAgn_00197920 | LG9 |  | leucine-rich repeat-containing protein 3b-like |
| DLA_LG1A_005850 | LG1A | DLAgn_00198000 | LG9 |  | NA |
| DLA_LG1A_005870 | LG1A | DLAgn_00198110 | LG9 |  | NA |
| DLA_LG1A_005880 | LG1A | DLAgn_00198210 | LG9 |  | NA |
| DLA_LG1A_005890 | LG1A | DLAgn_00198220 | LG9 |  | transcription factor hivep3 |
| DLA_LG1A_005900 | LG1A | DLAgn_00198390 | LG9 |  | NA |
| DLA_LG1A_005910 | LG1A | DLAgn_00198400 | LG9 |  | protocadherin alpha-3-like |
| DLA_LG1A_005920 | LG1A | DLAgn_00198500 | LG9 |  | transposase |
| DLA_LG1A_005930 | LG1A | DLAgn_00198540 | LG9 |  | NA |
| DLA_LG1A_005935 | LG1A | DLAgn_00198610 | LG9 |  | gap junction beta-3 |
| DLA_LG1A_005940 | LG1A | DLAgn_00198780 | LG9 | * | sry (sex determining region y)-box 4 |
| DLA_LG1A_005960 | LG1A | DLAgn_00198810 | LG9 |  | 39s ribosomal protein mitochondrial |
| DLA_LG1A_005970 | LG1A | DLAgn_00198870 | LG9 |  | PREDICTED: hypothetical protein LOC100330047 [Danio rerio] |
| DLA_LG1A_005980 | LG1A | DLAgn_00199100 | LG9 |  | NA |
| DLA_LG1A_005990 | LG1A | DLAgn_00199120 | LG9 |  | soss complex subunit c-like |
| DLA_LG1A_006000 | LG1A | DLAgn_00199230 | LG9 |  | uncharacterized protein loc101472750 |
| DLA_LG1A_006020 | LG1A | DLAgn_00199240 | LG9 |  | uncharacterized protein loc101472750 |
| DLA_LG1A_006030 | LG1A | DLAgn_00199360 | LG9 |  | upf0767 protein c1orf212 homolog |
| DLA_LG1A_006040 | LG1A | DLAgn_00199390 | LG9 |  | retrovirus polyprotein |
| DLA_LG1A_006050 | LG1A | DLAgn_00199550 | LG9 |  | tlr4 interactor with leucine rich repeats |
| DLA_LG1A_006060 | LG1A | DLAgn_00199780 | LG9 |  | proline-rich protein 15-like |
| DLA_LG1A_006070 | LG1A | DLAgn_00199870 | LG9 |  | piggybac transposable element-derived protein 3-like |
| DLA_LG1A_006090 | LG1A | DLAgn_00200020 | LG9 |  | NA |
| DLA_LG1A_006100 | LG1A | DLAgn_00200060 | LG9 |  | NA |
| DLA_LG1A_006110 | LG1A | DLAgn_00200260 | LG9 |  | fer3-like protein |
| DLA_LG1A_006120 | LG1A | DLAgn_00200290 | LG9 | * | alpha-( )-fucosyltransferase-like |
| DLA_LG1A_006125 | LG1A | DLAgn_00200330 | LG9 |  | lactose-binding lectin l-2-like |
| DLA_LG1A_006130 | LG1A | DLAgn_00200360 | LG9 |  | lactose-binding lectin l-2-like |
| DLA_LG1A_006140 | LG1A | DLAgn_00200370 | LG9 |  | lactose-binding lectin l-2-like |
| DLA_LG1A_006150 | LG1A | DLAgn_00200710 | LG9 |  | oocyte zinc finger protein 6-like |
| DLA_LG1A_006160 | LG1A | DLAgn_00200730 | LG9 |  | protein fam84b-like |
| DLA_LG1A_006165 | LG1A | DLAgn_00201200 | LG9 |  | protein fam84b-like |
| DLA_LG1A_006170 | LG1A | DLAgn_00201690 | LG9 |  | NA |
| DLA_LG1A_006180 | LG1A | DLAgn_00201990 | LG9 |  | leucine-rich repeat and immunoglobulin-like domain-containing nogo receptor-interacting protein 1-like |
| DLA_LG1A_006190 | LG1A | DLAgn_00202030 | LG9 |  | zinc finger protein 516-like |
| DLA_LG1A_006195 | LG1A | DLAgn_00202070 | LG9 |  | unnamed protein product [Tetraodon nigroviridis] |
| DLA_LG1A_006200 | LG1A | DLAgn_00202540 | LG9 |  | NA |
| DLA_LG1A_006210 | LG1A | DLAgn_00202710 | LG9 |  | dolichol-phosphate mannosyltransferase subunit 3 |
| DLA_LG1A_006220 | LG1A | DLAgn_00202880 | LG9 |  | NA |
| DLA_LG1A_006230 | LG1A | DLAgn_00203120 | LG9 |  | NA |
| DLA_LG1A_006240 | LG1A | DLAgn_00203610 | LG9 |  | tpa_exp: claudin 30 |
| DLA_LG1A_006250 | LG1A | DLAgn_00203650 | LG9 |  | sororin |
| DLA_LG1A_006260 | LG1A | DLAgn_00203870 | LG9 |  | cyclin-dependent kinase 5 activator 1-like |
| DLA_LG1A_006270 | LG1A | DLAgn_00203910 | LG9 |  | NA |
| DLA_LG1A_006290 | LG1A | DLAgn_00204090 | LG9 | * | free fatty acid receptor 2-like |
| DLA_LG1A_006300 | LG1A | DLAgn_00204280 | LG9 |  | NA |
| DLA_LG1A_006310 | LG1A | DLAgn_00204310 | LG9 |  | transmembrane protein 74-like |
| DLA_LG1A_006320 | LG1A | DLAgn_00204470 | LG9 |  | gap junction alpha-9 |
| DLA_LG1A_006330 | LG1A | DLAgn_00204650 | LG9 |  | transposase |
| DLA_LG1A_006340 | LG1A | DLAgn_00204770 | LG9 |  | NA |
| DLA_LG1A_006350 | LG1A | DLAgn_00204810 | LG9 |  | poliovirus receptor-related protein 2-like |
| DLA_LG1A_006360 | LG1A | DLAgn_00207580 | LGx |  | leucine-rich repeat neuronal protein 3-like |
| DLA_LG1A_006370 | LG1A | DLAgn_00204970 | LGx |  | cat eye syndrome critical region protein 6 |
| DLA_LG1A_006380 | LG1A | DLAgn_00205000 | LGx |  | novel protein |
| DLA_LG1A_006390 | LG1A | DLAgn_00205030 | LGx |  | transposable element tcb1 transposase |
| DLA_LG1A_006400 | LG1A | DLAgn_00205050 | LGx |  | uncharacterized protein loc101486585 |
| DLA_LG1A_006410 | LG1A | DLAgn_00205060 | LGx | * | NA |
| DLA_LG1A_006420 | LG1A | DLAgn_00205190 | LGx |  | reverse transcriptase-like protein |
| DLA_LG1A_006430 | LG1A | DLAgn_00205210 | LGx |  | NA |
| DLA_LG1A_006440 | LG1A | DLAgn_00205300 | LGx |  | atp-dependent dna helicase q1-like |
| DLA_LG1A_006450 | LG1A | DLAgn_00205320 | LGx |  | nuclear factor ovary-like |
| DLA_LG1A_006460 | LG1A | DLAgn_00205400 | LGx |  | e3 ubiquitin-protein ligase trim21-like |
| DLA_LG1A_006470 | LG1A | DLAgn_00205450 | LGx |  | e3 ubiquitin-protein ligase trim21-like |
| DLA_LG1A_006480 | LG1A | DLAgn_00205460 | LGx |  | reverse transcriptase |
| DLA_LG1A_006485 | LG1A | DLAgn_00205470 | LGx |  | reverse transcriptase |
| DLA_LG1A_006485_2 | LG1A | DLAgn_00205600 | LGx |  | NA |
| DLA_LG1A_006490 | LG1A | DLAgn_00205720 | LGx |  | NA |
| DLA_LG1A_006500 | LG1A | DLAgn_00205740 | LGx |  | NA |
| DLA_LG1A_006510 | LG1A | DLAgn_00205750 | LGx |  | NA |
| DLA_LG1A_006530 | LG1A | DLAgn_00205760 | LGx |  | NA |
| DLA_LG1A_006540 | LG1A | DLAgn_00205790 | LGx |  | NA |
| DLA_LG1A_006545 | LG1A | DLAgn_00205850 | LGx |  | e3 ubiquitin-protein ligase trim21-like |
| DLA_LG1A_006550 | LG1A | DLAgn_00206070 | LGx |  | pleckstrin homology-like domain family a member 1 |
| DLA_LG1A_006560 | LG1A | DLAgn_00206090 | LGx |  | e3 ubiquitin-protein ligase trim39-like |
| DLA_LG1A_006570 | LG1A | DLAgn_00206150 | LGx |  | neuron navigator 3 isoform 1 |
| DLA_LG1A_006590 | LG1A | DLAgn_00206290 | LGx |  | amphoterin-induced protein 2 |
| DLA_LG1A_006600 | LG1A | DLAgn_00206480 | LGx |  | neurotrophin-3 isoform 1 |
| DLA_LG1A_006610 | LG1A | DLAgn_00206500 | LGx |  | NA |
| DLA_LG1A_006620 | LG1A | DLAgn_00206510 | LGx |  | mam and ldl-receptor class a domain-containing protein c10orf112-like |
| DLA_LG1A_006630 | LG1A | DLAgn_00206730 | LGx |  | plexin-c1-like isoform x1 |
| DLA_LG1A_006650 | LG1A | DLAgn_00207110 | LGx |  | NA |
| DLA_LG1A_006660 | LG1A | DLAgn_00207170 | LGx |  | NA |
| DLA_LG1A_006680 | LG1A | DLAgn_00207220 | LGx |  | reverse transcriptase-like protein |
| DLA_LG1A_006690 | LG1A | DLAgn_00207290 | LGx |  | ras-related protein rab-8b isoform x5 |
| DLA_LG1A_006700 | LG1A | DLAgn_00207620 | LGx |  | probable g protein-coupled receptor 85-like |
| DLA_LG1A_006710 | LG1A | DLAgn_00207660 | LGx |  | NA |
| DLA_LG1A_006720 | LG1A | DLAgn_00207920 | LGx |  | NA |
| DLA_LG1A_006730 | LG1A | DLAgn_00207960 | LGx |  | tripartite motif-containing protein 39-like |
| DLA_LG1A_006740 | LG1A | DLAgn_00208160 | LGx |  | NA |
| DLA_LG1A_006750 | LG1A | DLAgn_00208230 | LGx |  | urocortin 3 precursor |
| DLA_LG1A_006770 | LG1A | DLAgn_00208260 | LGx |  | NA |
| DLA_LG1A_006780 | LG1A | DLAgn_00208490 | LGx |  | leucine-rich repeat-containing protein 4-like |
| DLA_LG1A_006790 | LG1A | DLAgn_00208550 | LGx |  | inosine-5 -monophosphate dehydrogenase 1b-like isoform x1 |
| DLA_LG1A_006840 | LG1A | DLAgn_00208800 | LGx |  | tetraspanin-7 |
| DLA_LG1A_006850 | LG1A | DLAgn_00208870 | LGx |  | transcription factor spi-c |
| DLA_LG1A_006880 | LG1A | DLAgn_00209360 | LGx |  | NA |
| DLA_LG1A_006890 | LG1A | DLAgn_00209410 | LGx |  | troponin cardiac muscle isoforms-like |
| DLA_LG1A_006900 | LG1A | DLAgn_00209510 | LGx |  | NA |
| DLA_LG1A_006910 | LG1A | DLAgn_00210190 | LGx |  | nuclear factor ovary-like |
| DLA_LG1A_006920 | LG1A | DLAgn_00008270 | LG10 |  | leucine-rich repeat-containing protein 30-like |
| DLA_LG1A_006930 | LG1A | DLAgn_00000050 | LG10 |  | unnamed protein product [Tetraodon nigroviridis] |
| DLA_LG1A_006940 | LG1A | DLAgn_00000200 | LG10 |  | zinc finger bed domain-containing protein 5 isoform x1 |
| DLA_LG1A_006980 | LG1A | DLAgn_00000260 | LG10 |  | NA |
| DLA_LG1A_006990 | LG1A | DLAgn_00000340 | LG10 |  | uncharacterized |
| DLA_LG1A_007000 | LG1A | DLAgn_00000360 | LG10 |  | transposable element tcb1 transposase |
| DLA_LG1A_007010 | LG1A | DLAgn_00000890 | LG10 |  | taste type member |
| DLA_LG1A_007020 | LG1A | DLAgn_00000940 | LG10 |  | protein-tyrosine kinase 2-beta-like |
| DLA_LG1A_007030 | LG1A | DLAgn_00000950 | LG10 |  | protein-tyrosine kinase 2-beta-like |
| DLA_LG1A_007040 | LG1A | DLAgn_00000970 | LG10 |  | NA |
| DLA_LG1A_007050 | LG1A | DLAgn_00002290 | LG10 |  | novel sal-like protein |
| DLA_LG1A_007060 | LG1A | DLAgn_00002340 | LG10 |  | leucine rich adaptor protein 1-like |
| DLA_LG1A_007070 | LG1A | DLAgn_00002460 | LG10 |  | transcription factor ap-1-like |
| DLA_LG1A_007080 | LG1A | DLAgn_00002600 | LG10 |  | transmembrane protein 125-like |
| DLA_LG1A_007090 | LG1A | DLAgn_00002900 | LG10 |  | beta- -galactosyltransferase 2 |
| DLA_LG1A_007100 | LG1A | DLAgn_00002910 | LG10 |  | uncharacterized protein loc101486585 |
| DLA_LG1A_007110 | LG1A | DLAgn_00003100 | LG10 |  | uncharacterized protein c5orf35-like |
| DLA_LG1A_007120 | LG1A | DLAgn_00003450 | LG10 |  | transcription factor sox-14-like |
| DLA_LG1A_007130 | LG1A | DLAgn_00004000 | LG10 |  | transcription factor jun-d |
| DLA_LG1A_007135 | LG1A | DLAgn_00004050 | LG10 |  | cortexin-1 isoform x1 |
| DLA_LG1A_007150 | LG1A | DLAgn_00004100 | LG10 |  | NA |
| DLA_LG1A_007160 | LG1A | DLAgn_00004380 | LG10 |  | protein fem-1 homolog a-like |
| DLA_LG1A_007170 | LG1A | DLAgn_00004420 | LG10 |  | g-protein coupled receptor 35-like |
| DLA_LG1A_007180 | LG1A | DLAgn_00004480 | LG10 |  | novel protein containing immunoglobulin domains |
| DLA_LG1A_007190 | LG1A | DLAgn_00005120 | LG10 |  | NA |
| DLA_LG1A_007200 | LG1A | DLAgn_00005160 | LG10 |  | ras-related protein rap-2b-like |
| DLA_LG1A_007210 | LG1A | DLAgn_00005450 | LG10 |  | adp-ribosylation factor-like protein 14 |
| DLA_LG1A_007220 | LG1A | DLAgn_00005740 | LG10 |  | NA |
| DLA_LG1A_007230 | LG1A | DLAgn_00005870 | LG10 |  | uncharacterized protein loc76237 |
| DLA_LG1A_007240 | LG1A | DLAgn_00005970 | LG10 |  | NA |
| DLA_LG1A_007260 | LG1A | DLAgn_00006110 | LG10 |  | zinc finger bed domain-containing protein 1 |
| DLA_LG1A_007270 | LG1A | DLAgn_00006320 | LG10 |  | NA |
| DLA_LG1A_007290 | LG1A | DLAgn_00006330 | LG10 |  | forkhead box protein q1-like |
| DLA_LG1A_007300 | LG1A | DLAgn_00006350 | LG10 |  | uncharacterized protein loc101169091 |
| DLA_LG1A_007320 | LG1A | DLAgn_00006390 | LG10 |  | uncharacterized protein loc101169091 |
| DLA_LG1A_007330 | LG1A | DLAgn_00006500 | LG10 |  | zinc finger protein 830-like |
| DLA_LG1A_007340 | LG1A | DLAgn_00006550 | LG10 |  | voltage-dependent r-type calcium channel subunit alpha-1e |
| DLA_LG1A_007350 | LG1A | DLAgn_00006610 | LG10 |  | ccaat enhancer-binding protein delta |
| DLA_LG1A_007360 | LG1A | DLAgn_00006870 | LG10 |  | 7-alpha-hydroxycholest-4-en-3-one 12-alpha-hydroxylase-like |
| DLA_LG1A_007370 | LG1A | DLAgn_00007140 | LG10 |  | protein fuzzy homolog |
| DLA_LG1A_007390 | LG1A | DLAgn_00008070 | LG10 |  | NA |
| DLA_LG1A_007395 | LG1A | DLAgn_00008130 | LG10 |  | disks large homolog 5 |
| DLA_LG1A_007400 | LG1A | DLAgn_00008380 | LG10 |  | NA |
| DLA_LG1A_007410 | LG1A | DLAgn_00009010 | LG10 |  | NA |
| DLA_LG1A_007420 | LG1A | DLAgn_00009170 | LG10 |  | cxorf33 precursor |
| DLA_LG1A_007430 | LG1A | DLAgn_00009200 | LG10 |  | NA |
| DLA_LG1A_007440 | LG1A | DLAgn_00009390 | LG10 |  | frizzled-8-like |
| DLA_LG1A_007450 | LG1A | DLAgn_00009460 | LG10 | * | tripartite motif-containing protein 16-like |
| DLA_LG1A_007460 | LG1A | DLAgn_00009470 | LG10 |  | tripartite motif-containing protein 16-like |
| DLA_LG1A_007480 | LG1A | DLAgn_00009540 | LG10 |  | NA |
| DLA_LG1A_007490 | LG1A | DLAgn_00009560 | LG10 |  | tripartite motif-containing protein 16-like |
| DLA_LG1A_007500 | LG1A | DLAgn_00009590 | LG10 | * | tripartite motif-containing protein 16-like |
| DLA_LG1A_007510 | LG1A | DLAgn_00009650 | LG10 |  | tripartite motif-containing protein 16-like |
| DLA_LG1A_007520 | LG1A | DLAgn_00009660 | LG10 |  | tripartite motif-containing protein 16-like |
| DLA_LG1A_007530 | LG1A | DLAgn_00009700 | LG10 |  | e3 ubiquitin-protein ligase rnf182-like |
| DLA_LG1A_007540 | LG1A | DLAgn_00009720 | LG10 |  | NA |
| DLA_LG1A_007550 | LG1A | DLAgn_00009850 | LG11 | * | NA |
| DLA_LG1A_007560 | LG1A | DLAgn_00009860 | LG11 |  | NA |
| DLA_LG1A_007570 | LG1A | DLAgn_00009870 | LG11 |  | hydroxycarboxylic acid receptor 2-like |
| DLA_LG1A_007580 | LG1A | DLAgn_00010390 | LG11 |  | heparan sulfate glucosamine 3-o-sulfotransferase 1-like |
| DLA_LG1A_007585 | LG1A | DLAgn_00010430 | LG11 |  | zinc finger protein 518a |
| DLA_LG1A_007590 | LG1A | DLAgn_00010610 | LG11 |  | protein atonal homolog 7-like |
| DLA_LG1A_007610 | LG1A | DLAgn_00011230 | LG11 |  | transmembrane protein 121-like |
| DLA_LG1A_007620 | LG1A | DLAgn_00011290 | LG11 |  | uncharacterized protein kiaa1383 homolog |
| DLA_LG1A_007630 | LG1A | DLAgn_00011570 | LG11 |  | protein slx4ip-like |
| DLA_LG1A_007640 | LG1A | DLAgn_00011620 | LG11 |  | transposase |
| DLA_LG1A_007650 | LG1A | DLAgn_00011660 | LG11 |  | leucine-rich repeat transmembrane protein flrt3 |
| DLA_LG1A_007660 | LG1A | DLAgn_00011830 | LG11 |  | coiled-coil domain-containing protein 177 |
| DLA_LG1A_007670 | LG1A | DLAgn_00011850 | LG11 |  | transforming acidic coiled-coil-containing protein 2-like isoform x1 |
| DLA_LG1A_007680 | LG1A | DLAgn_00011860 | LG11 |  | transforming acidic coiled-coil-containing protein 2-like isoform x1 |
| DLA_LG1A_007690 | LG1A | DLAgn_00011870 | LG11 |  | transforming acidic coiled-coil-containing protein 2-like isoform x1 |
| DLA_LG1A_007700 | LG1A | DLAgn_00012020 | LG11 |  | proline-rich protein 18 |
| DLA_LG1A_007710 | LG1A | DLAgn_00012090 | LG11 |  | zinc finger ccch domain-containing protein 6 |
| DLA_LG1A_007720 | LG1A | DLAgn_00012220 | LG11 |  | probable g-protein coupled receptor 75-like |
| DLA_LG1A_007735 | LG1A | DLAgn_00012380 | LG11 |  | brain-specific angiogenesis inhibitor 3-like |
| DLA_LG1A_007750 | LG1A | DLAgn_00012410 | LG11 |  | protein eyes shut homolog |
| DLA_LG1A_007760 | LG1A | DLAgn_00012500 | LG11 |  | dystonin-like isoform 2 |
| DLA_LG1A_007770 | LG1A | DLAgn_00012520 | LG11 |  | low quality protein: dystonin-like |
| DLA_LG1A_007780 | LG1A | DLAgn_00012730 | LG11 |  | neuropeptide y receptor type 1-like |
| DLA_LG1A_007785 | LG1A | DLAgn_00012920 | LG11 |  | shadow of prion |
| DLA_LG1A_007790 | LG1A | DLAgn_00013060 | LG11 |  | leucine-rich repeat-containing protein 18-like |
| DLA_LG1A_007800 | LG1A | DLAgn_00013250 | LG11 |  | hermansky-pudlak syndrome 6 protein |
| DLA_LG1A_007810 | LG1A | DLAgn_00013270 | LG11 |  | NA |
| DLA_LG1A_007820 | LG1A | DLAgn_00013340 | LG11 |  | NA |
| DLA_LG1A_007830 | LG1A | DLAgn_00013360 | LG11 |  | ligand-dependent corepressor |
| DLA_LG1A_007840 | LG1A | DLAgn_00013560 | LG11 |  | marvel domain-containing protein 1 |
| DLA_LG1A_007850 | LG1A | DLAgn_00013920 | LG11 |  | probable vesicular acetylcholine transporter-a-like |
| DLA_LG1A_007860 | LG1A | DLAgn_00013990 | LG11 |  | unnamed protein product [Tetraodon nigroviridis] |
| DLA_LG1A_007870 | LG1A | DLAgn_00014410 | LG11 |  | cgmp-dependent protein kinase 1-like isoform 1 |
| DLA_LG1A_007920 | LG1A | DLAgn_00014700 | LG11 |  | unnamed protein product [Tetraodon nigroviridis] |
| DLA_LG1A_007930 | LG1A | DLAgn_00014870 | LG11 |  | NA |
| DLA_LG1A_007940 | LG1A | DLAgn_00015100 | LG11 |  | gremlin-2 precursor |
| DLA_LG1A_007950 | LG1A | DLAgn_00015120 | LG11 |  | NA |
| DLA_LG1A_007970 | LG1A | DLAgn_00015300 | LG11 |  | splicing factor 3b subunit 5 |
| DLA_LG1A_007980 | LG1A | DLAgn_00015380 | LG11 |  | uncharacterized protein loc101480770 |
| DLA_LG1A_007990 | LG1A | DLAgn_00015390 | LG11 |  | oligodendrocyte transcription factor 3 |
| DLA_LG1A_008000 | LG1A | DLAgn_00015450 | LG11 |  | NA |
| DLA_LG1A_008010 | LG1A | DLAgn_00015470 | LG11 |  | NA |
| DLA_LG1A_008020 | LG1A | DLAgn_00015640 | LG11 |  | NA |
| DLA_LG1A_008040 | LG1A | DLAgn_00015650 | LG11 |  | NA |
| DLA_LG1A_008050 | LG1A | DLAgn_00016210 | LG11 |  | zinc finger protein 850-like |
| DLA_LG1A_008055 | LG1A | DLAgn_00016230 | LG11 | * | zinc finger protein 182 |
| DLA_LG1A_008060 | LG1A | DLAgn_00016450 | LG11 |  | homeobox protein 11aa |
| DLA_LG1A_008070 | LG1A | DLAgn_00016610 | LG11 |  | zinc finger protein 804b-like |
| DLA_LG1A_008080 | LG1A | DLAgn_00016820 | LG11 |  | NA |
| DLA_LG1A_008090 | LG1A | DLAgn_00016830 | LG11 |  | pro-neuregulin- membrane-bound isoform-like |
| DLA_LG1A_008100 | LG1A | DLAgn_00016840 | LG11 |  | NA |
| DLA_LG1A_008110 | LG1A | DLAgn_00016850 | LG11 |  | NA |
| DLA_LG1A_008140 | LG1A | DLAgn_00016860 | LG11 |  | NA |
| DLA_LG1A_008150 | LG1A | DLAgn_00017030 | LG11 |  | NA |
| DLA_LG1A_008160 | LG1A | DLAgn_00017040 | LG11 |  | protein nlrc3-like |
| DLA_LG1A_008170 | LG1A | DLAgn_00017090 | LG11 |  | forkhead box protein g1-like |
| DLA_LG1A_008180 | LG1A | DLAgn_00017130 | LG11 |  | 2-aminoethanethiol dioxygenase |
| DLA_LG1A_008190 | LG1A | DLAgn_00017170 | LG11 |  | cd48 antigen precursor |
| DLA_LG1A_008200 | LG1A | DLAgn_00017210 | LG11 |  | d -like dopamine receptor-like |
| DLA_LG1A_008220 | LG1A | DLAgn_00017540 | LG11 |  | g protein-regulated inducer of neurite outgrowth 2-like |
| DLA_LG1A_008225 | LG1A | DLAgn_00017660 | LG11 |  | transposable element tcb2 transposase |
| DLA_LG1A_008230 | LG1A | DLAgn_00017830 | LG11 |  | NA |
| DLA_LG1A_008240 | LG1A | DLAgn_00017950 | LG11 |  | reverse transcriptase-like protein |
| DLA_LG1A_008250 | LG1A | DLAgn_00018240 | LG11 | * | scan domain-containing protein 3-like |
| DLA_LG1A_008260 | LG1A | DLAgn_00025420 | LG12 |  | NA |
| DLA_LG1A_008280 | LG1A | DLAgn_00018290 | LG12 | * | general transcription factor ii-i repeat domain-containing protein 2-like |
| DLA_LG1A_008290 | LG1A | DLAgn_00018340 | LG12 |  | gtpase imap family member 7 |
| DLA_LG1A_008320 | LG1A | DLAgn_00018360 | LG12 |  | leucine-rich repeat transmembrane protein flrt2-like |
| DLA_LG1A_008330 | LG1A | DLAgn_00018370 | LG12 |  | general transcription factor ii-i repeat domain-containing protein 2-like |
| DLA_LG1A_008340 | LG1A | DLAgn_00018410 | LG12 |  | reverse transcriptase-like protein |
| DLA_LG1A_008350 | LG1A | DLAgn_00018420 | LG12 | * | reverse transcriptase-like protein |
| DLA_LG1A_008360 | LG1A | DLAgn_00018450 | LG12 |  | NA |
| DLA_LG1A_008370 | LG1A | DLAgn_00018510 | LG12 |  | gap junction alpha-10 |
| DLA_LG1A_008380 | LG1A | DLAgn_00018750 | LG12 |  | uncharacterized protein loc101171476 |
| DLA_LG1A_008390 | LG1A | DLAgn_00018770 | LG12 |  | zinc-binding protein a33-like |
| DLA_LG1A_008410 | LG1A | DLAgn_00019080 | LG12 |  | NA |
| DLA_LG1A_008420 | LG1A | DLAgn_00019150 | LG12 |  | potassium voltage-gated channel subfamily f member 1 |
| DLA_LG1A_008430 | LG1A | DLAgn_00019350 | LG12 |  | alk tyrosine kinase receptor |
| DLA_LG1A_008440 | LG1A | DLAgn_00019530 | LG12 |  | heat shock protein 30 |
| DLA_LG1A_008450 | LG1A | DLAgn_00019630 | LG12 |  | insulinoma-associated protein 2 |
| DLA_LG1A_008460 | LG1A | DLAgn_00019650 | LG12 |  | ral gtpase-activating protein subunit alpha-1 |
| DLA_LG1A_008470 | LG1A | DLAgn_00019720 | LG12 |  | PREDICTED: hypothetical protein LOC798447 [Danio rerio] |
| DLA_LG1A_008480 | LG1A | DLAgn_00019770 | LG12 |  | pol protein |
| DLA_LG1A_008490 | LG1A | DLAgn_00019810 | LG12 |  | leucine-rich repeat and immunoglobulin-like domain-containing nogo receptor-interacting protein 2-like |
| DLA_LG1A_008520 | LG1A | DLAgn_00019870 | LG12 |  | complement component c1q receptor |
| DLA_LG1A_008530 | LG1A | DLAgn_00019890 | LG12 |  | serta domain-containing protein 4-like |
| DLA_LG1A_008560 | LG1A | DLAgn_00019970 | LG12 |  | oligodendrocyte transcription factor 3-like |
| DLA_LG1A_008570 | LG1A | DLAgn_00020100 | LG12 |  | NA |
| DLA_LG1A_008580 | LG1A | DLAgn_00020260 | LG12 |  | insulinoma-associated protein 1-like |
| DLA_LG1A_008590 | LG1A | DLAgn_00020290 | LG12 |  | NA |
| DLA_LG1A_008600 | LG1A | DLAgn_00020430 | LG12 |  | claudin-20-like |
| DLA_LG1A_008605 | LG1A | DLAgn_00020450 | LG12 |  | NA |
| DLA_LG1A_008610 | LG1A | DLAgn_00020610 | LG12 |  | NA |
| DLA_LG1A_008620 | LG1A | DLAgn_00020770 | LG12 |  | uncharacterized protein loc101476285 |
| DLA_LG1A_008630 | LG1A | DLAgn_00020960 | LG12 |  | cytochrome p450 1b1-like |
| DLA_LG1A_008640 | LG1A | DLAgn_00020970 | LG12 |  | cytochrome p450 1b1-like |
| DLA_LG1A_008650 | LG1A | DLAgn_00021110 | LG12 |  | muscarinic acetylcholine receptor m5-like |
| DLA_LG1A_008660 | LG1A | DLAgn_00021850 | LG12 |  | NA |
| DLA_LG1A_008680 | LG1A | DLAgn_00021880 | LG12 |  | NA |
| DLA_LG1A_008690 | LG1A | DLAgn_00021900 | LG12 |  | NA |
| DLA_LG1A_008700 | LG1A | DLAgn_00022080 | LG12 |  | protein fam181a-like |
| DLA_LG1A_008725 | LG1A | DLAgn_00022450 | LG12 |  | NA |
| DLA_LG1A_008730 | LG1A | DLAgn_00022670 | LG12 |  | dnaj homolog subfamily c member 30-like |
| DLA_LG1A_008740 | LG1A | DLAgn_00022750 | LG12 |  | NA |
| DLA_LG1A_008750 | LG1A | DLAgn_00022800 | LG12 |  | sry-box containing gene 11a |
| DLA_LG1A_008770 | LG1A | DLAgn_00022850 | LG12 |  | cannabinoid receptor type 1b-like |
| DLA_LG1A_008780 | LG1A | DLAgn_00022930 | LG12 |  | cannabinoid receptor type 1b-like |
| DLA_LG1A_008800 | LG1A | DLAgn_00023340 | LG12 |  | platelet-activating factor receptor-like |
| DLA_LG1A_008810 | LG1A | DLAgn_00023490 | LG12 |  | b2 bradykinin receptor |
| DLA_LG1A_008820 | LG1A | DLAgn_00023510 | LG12 |  | psychosine receptor |
| DLA_LG1A_008830 | LG1A | DLAgn_00023550 | LG12 |  | protein nlrc3-like |
| DLA_LG1A_008840 | LG1A | DLAgn_00023600 | LG12 |  | NA |
| DLA_LG1A_008850 | LG1A | DLAgn_00023650 | LG12 |  | intron-binding protein partial |
| DLA_LG1A_008855 | LG1A | DLAgn_00023700 | LG12 |  | NA |
| DLA_LG1A_008860 | LG1A | DLAgn_00023790 | LG12 |  | alpha-( )-fucosyltransferase |
| DLA_LG1A_008870 | LG1A | DLAgn_00024020 | LG12 |  | transposase |
| DLA_LG1A_008875 | LG1A | DLAgn_00024440 | LG12 |  | immunoglobulin light chain |
| DLA_LG1A_008880 | LG1A | DLAgn_00024520 | LG12 |  | NA |
| DLA_LG1A_008885 | LG1A | DLAgn_00024580 | LG12 |  | solute carrier family 35 member g2-like |
| DLA_LG1A_008890 | LG1A | DLAgn_00024910 | LG12 |  | transmembrane protein 229b-like |
| DLA_LG1A_008905 | LG1A | DLAgn_00024950 | LG12 |  | NA |
| DLA_LG1A_008905_2 | LG1A | DLAgn_00025150 | LG12 |  | NA |
| DLA_LG1A_008905_4 | LG1A | DLAgn_00025210 | LG12 |  | syntaxin-11-like |
| DLA_LG1A_008910 | LG1A | DLAgn_00025320 | LG12 |  | forkhead box protein partial |
| DLA_LG1A_008930 | LG1A | DLAgn_00025520 | LG12 |  | interferon-induced very large gtpase 1-like |
| DLA_LG1A_008960 | LG1A | DLAgn_00025700 | LG12 |  | uncharacterized protein loc101466565 isoform x1 |
| DLA_LG1A_008970 | LG1A | DLAgn_00026080 | LG12 |  | coiled-coil domain-containing protein 177 |
| DLA_LG1A_008980 | LG1A | DLAgn_00026150 | LG12 |  | NA |
| DLA_LG1A_008990 | LG1A | DLAgn_00026170 | LG12 |  | NA |
| DLA_LG1A_009000 | LG1A | DLAgn_00026230 | LG12 |  | NA |
| DLA_LG1A_009010 | LG1A | DLAgn_00026260 | LG12 |  | uncharacterized protein loc101175210 |
| DLA_LG1A_009020 | LG1A | DLAgn_00026310 | LG12 |  | NA |
| DLA_LG1A_009030 | LG1A | DLAgn_00026540 | LG12 |  | nanos homolog 1-like |
| DLA_LG1A_009040 | LG1A | DLAgn_00026600 | LG12 |  | probable g-protein coupled receptor 151 |
| DLA_LG1A_009070 | LG1A | DLAgn_00026730 | LG12 |  | NA |
| DLA_LG1A_009075 | LG1A | DLAgn_00026750 | LG12 |  | NA |
| DLA_LG1A_009090 | LG1A | DLAgn_00026930 | LG12 |  | protein ahnak2-like |
| DLA_LG1A_009100 | LG1A | DLAgn_00027040 | LG12 |  | bag family molecular chaperone regulator 5 |
| DLA_LG1A_009120 | LG1A | DLAgn_00027070 | LG12 |  | e3 ubiquitin isg15 ligase trim25-like |
| DLA_LG1A_009130 | LG1A | DLAgn_00027080 | LG12 |  | tripartite motif-containing protein 16-like |
| DLA_LG1A_009140 | LG1A | DLAgn_00027100 | LG12 |  | protein nlrc3-like |
| DLA_LG1A_009150 | LG1A | DLAgn_00027110 | LG12 |  | protein nlrc3-like |
| DLA_LG1A_009160 | LG1A | DLAgn_00027140 | LG12 |  | NA |
| DLA_LG1A_009165 | LG1A | DLAgn_00027150 | LG12 |  | e3 ubiquitin-protein ligase trim21-like |
| DLA_LG1A_009190 | LG1A | DLAgn_00027540 | LG13 |  | NA |
| DLA_LG1A_009200 | LG1A | DLAgn_00027600 | LG13 |  | p2y purinoceptor 14 |
| DLA_LG1A_009210 | LG1A | DLAgn_00027620 | LG13 |  | zinc finger protein 729-like |
| DLA_LG1A_009220 | LG1A | DLAgn_00027760 | LG13 |  | NA |
| DLA_LG1A_009230 | LG1A | DLAgn_00027770 | LG13 |  | NA |
| DLA_LG1A_009240 | LG1A | DLAgn_00027870 | LG13 |  | olfactory receptor 52k1-like |
| DLA_LG1A_009250 | LG1A | DLAgn_00027880 | LG13 |  | olfactory receptor 52k1-like |
| DLA_LG1A_009260 | LG1A | DLAgn_00027890 | LG13 |  | odorant receptor |
| DLA_LG1A_009265 | LG1A | DLAgn_00027970 | LG13 |  | NA |
| DLA_LG1A_009270 | LG1A | DLAgn_00027980 | LG13 |  | NA |
| DLA_LG1A_009280 | LG1A | DLAgn_00028000 | LG13 |  | NA |
| DLA_LG1A_009290 | LG1A | DLAgn_00028020 | LG13 |  | NA |
| DLA_LG1A_009300 | LG1A | DLAgn_00028030 | LG13 |  | gap junction gamma-1 |
| DLA_LG1A_009310 | LG1A | DLAgn_00028050 | LG13 |  | NA |
| DLA_LG1A_009315 | LG1A | DLAgn_00028100 | LG13 |  | trace amine-associated receptor 1-like |
| DLA_LG1A_009320 | LG1A | DLAgn_00028160 | LG13 |  | phospholipid scramblase 1 |
| DLA_LG1A_009330 | LG1A | DLAgn_00028190 | LG13 |  | unconventional myosin-xviiia-like isoform x1 |
| DLA_LG1A_009340 | LG1A | DLAgn_00028340 | LG13 |  | protein phosphatase 1d-like |
| DLA_LG1A_009370 | LG1A | DLAgn_00028600 | LG13 |  | uncharacterized protein loc101468230 isoform x1 |
| DLA_LG1A_009380 | LG1A | DLAgn_00028610 | LG13 |  | pou domain class 2-associating factor 1-like |
| DLA_LG1A_009390 | LG1A | DLAgn_00028830 | LG13 |  | NA |
| DLA_LG1A_009400 | LG1A | DLAgn_00028990 | LG13 |  | NA |
| DLA_LG1A_009410 | LG1A | DLAgn_00029150 | LG13 |  | e3 ubiquitin-protein ligase rnf182-like |
| DLA_LG1A_009425 | LG1A | DLAgn_00029220 | LG13 |  | lysophosphatidic acid receptor 6-like |
| DLA_LG1A_009440 | LG1A | DLAgn_00029260 | LG13 |  | forkhead box l2 |
| DLA_LG1A_009460 | LG1A | DLAgn_00029280 | LG13 |  | NA |
| DLA_LG1A_009470 | LG1A | DLAgn_00029350 | LG13 |  | lysophosphatidic acid receptor 6-like |
| DLA_LG1A_009480 | LG1A | DLAgn_00029380 | LG13 |  | NA |
| DLA_LG1A_009490 | LG1A | DLAgn_00029730 | LG13 |  | NA |
| DLA_LG1A_009495 | LG1A | DLAgn_00030290 | LG13 |  | atp-sensitive inward rectifier potassium channel 1-like |
| DLA_LG1B_003810 | LG1B | DLAgn_00030420 | LG13 |  | forkhead box g1 |
| DLA_LG1B_000290 | LG1B | DLAgn_00030890 | LG13 |  | slit and ntrk-like protein 3-like |
| DLA_LG1B_002230 | LG1B | DLAgn_00030910 | LG13 | ** | uncharacterized protein loc101466409 isoform x1 |
| DLA_LG1B_002750 | LG1B | DLAgn_00031140 | LG13 |  | frizzled-like protein 9 |
| DLA_LG1B_003580 | LG1B | DLAgn_00031260 | LG13 |  | NA |
| DLA_LG1B_004290 | LG1B | DLAgn_00031500 | LG13 |  | calcium voltage- p q alpha 1a isoform cra_a |
| DLA_LG1B_005090 | LG1B | DLAgn_00031540 | LG13 |  | unnamed protein product [Tetraodon nigroviridis] |
| DLA_LG1B_005720 | LG1B | DLAgn_00031630 | LG13 |  | NA |
| DLA_LG1B_006300 | LG1B | DLAgn_00031680 | LG13 |  | NA |
| DLA_LG1B_006780 | LG1B | DLAgn_00031810 | LG13 |  | NA |
| DLA_LG1B_000160 | LG1B | DLAgn_00031890 | LG13 |  | protein unc-119 homolog a-like |
| DLA_LG1B_000310 | LG1B | DLAgn_00032190 | LG13 |  | ring finger protein 26 |
| DLA_LG1B_000665 | LG1B | DLAgn_00032570 | LG13 |  | NA |
| DLA_LG1B_000910 | LG1B | DLAgn_00032750 | LG13 |  | NA |
| DLA_LG1B_001230 | LG1B | DLAgn_00032850 | LG13 |  | histone h2a type 2-b-like |
| DLA_LG1B_001325 | LG1B | DLAgn_00032970 | LG13 |  | NA |
| DLA_LG1B_001590 | LG1B | DLAgn_00033150 | LG13 |  | alpha-( )-fucosyltransferase-like |
| DLA_LG1B_001930 | LG1B | DLAgn_00033190 | LG13 |  | dnaj homolog subfamily c member 30-like |
| DLA_LG1B_002290 | LG1B | DLAgn_00033280 | LG13 |  | serine palmitoyltransferase small subunit b |
| DLA_LG1B_002350 | LG1B | DLAgn_00033350 | LG13 |  | uncharacterized membrane protein c3orf80 homolog |
| DLA_LG1B_002420 | LG1B | DLAgn_00033380 | LG13 |  | p2y purinoceptor 2-like |
| DLA_LG1B_002900 | LG1B | DLAgn_00033480 | LG13 |  | ras-related protein rap-2b-like |
| DLA_LG1B_003150 | LG1B | DLAgn_00033590 | LG13 |  | extracellular calcium-sensing receptor-like |
| DLA_LG1B_003630 | LG1B | DLAgn_00033650 | LG13 |  | extracellular calcium-sensing receptor-like |
| DLA_LG1B_003640 | LG1B | DLAgn_00034080 | LG13 |  | suppressor of cytokine signaling 9 |
| DLA_LG1B_003880 | LG1B | DLAgn_00034420 | LG13 |  | NA |
| DLA_LG1B_003950 | LG1B | DLAgn_00034560 | LG13 |  | odorant receptor |
| DLA_LG1B_004050 | LG1B | DLAgn_00034570 | LG13 |  | odorant receptor |
| DLA_LG1B_004080 | LG1B | DLAgn_00034600 | LG13 |  | odorant receptor |
| DLA_LG1B_004150 | LG1B | DLAgn_00034690 | LG13 |  | trace amine-associated receptor 1-like |
| DLA_LG1B_004200 | LG1B | DLAgn_00034700 | LG13 |  | e3 ubiquitin isg15 ligase trim25-like |
| DLA_LG1B_004210 | LG1B | DLAgn_00034850 | LG13 |  | olfactory receptor family f subfamily 600 member 1 |
| DLA_LG1B_004420 | LG1B | DLAgn_00034860 | LG13 |  | odorant receptor |
| DLA_LG1B_004480 | LG1B | DLAgn_00034880 | LG13 |  | odorant receptor |
| DLA_LG1B_004490 | LG1B | DLAgn_00034890 | LG13 |  | odorant receptor |
| DLA_LG1B_004530 | LG1B | DLAgn_00034910 | LG13 |  | odorant receptor |
| DLA_LG1B_005010 | LG1B | DLAgn_00034920 | LG13 |  | odorant receptor |
| DLA_LG1B_005020 | LG1B | DLAgn_00034930 | LG13 |  | odorant receptor |
| DLA_LG1B_005040 | LG1B | DLAgn_00034940 | LG13 |  | odorant receptor |
| DLA_LG1B_005140 | LG1B | DLAgn_00035050 | LG13 |  | piggybac transposable element-derived protein 4-like |
| DLA_LG1B_005230 | LG1B | DLAgn_00035070 | LG13 |  | protein mab-21-like 1-like |
| DLA_LG1B_005500 | LG1B | DLAgn_00035180 | LG13 |  | nuclease harbi1-like |
| DLA_LG1B_005630 | LG1B | DLAgn_00035220 | LG13 |  | claudin-8-like |
| DLA_LG1B_005760 | LG1B | DLAgn_00035230 | LG13 |  | claudin-8-like |
| DLA_LG1B_005840 | LG1B | DLAgn_00035240 | LG13 |  | claudin-8-like |
| DLA_LG1B_005940 | LG1B | DLAgn_00035310 | LG13 |  | general transcription factor ii-i repeat domain-containing protein 2-like |
| DLA_LG1B_005970 | LG1B | DLAgn_00035400 | LG13 |  | lysophosphatidic acid receptor 6-like |
| DLA_LG1B_006090 | LG1B | DLAgn_00035470 | LG13 |  | mid1-interacting protein 1-b-like |
| DLA_LG1B_006380 | LG1B | DLAgn_00035500 | LG13 |  | protein fam181b |
| DLA_LG1B_006530 | LG1B | DLAgn_00035770 | LG13 |  | nuclear pore complex protein nup88 |
| DLA_LG1B_006650 | LG1B | DLAgn_00035810 | LG13 |  | udp- c:betagal beta- -n-acetylglucosaminyltransferase 2-like |
| DLA_LG1B_006680 | LG1B | DLAgn_00036010 | LG13 |  | claudin 3 |
| DLA_LG1B_006860 | LG1B | DLAgn_00036020 | LG13 |  | claudin-3-like |
| DLA_LG1B_006930 | LG1B | DLAgn_00036030 | LG13 |  | claudin 9 |
| DLA_LG1B_007050 | LG1B | DLAgn_00036040 | LG13 |  | claudin-like protein zf-a89 |
| DLA_LG1B_007160 | LG1B | DLAgn_00036050 | LG13 |  | claudin 4 |
| DLA_LG1B_007230 | LG1B | DLAgn_00036060 | LG13 |  | claudin-like protein zf-a89 |
| DLA_LG1B_007360 | LG1B | DLAgn_00036070 | LG13 |  | claudin-like protein zf-a89-like |
| DLA_LG1B_000005 | LG1B | DLAgn_00036080 | LG13 |  | claudin 4 |
| DLA_LG1B_000005_2 | LG1B | DLAgn_00036090 | LG13 |  | claudin 4 |
| DLA_LG1B_000010 | LG1B | DLAgn_00036110 | LG13 |  | tpa_exp: claudin 13 |
| DLA_LG1B_000020 | LG1B | DLAgn_00036210 | LG13 |  | NA |
| DLA_LG1B_000060 | LG1B | DLAgn_00036400 | LG13 |  | NA |
| DLA_LG1B_000070 | LG1B | DLAgn_00036490 | LG13 |  | NA |
| DLA_LG1B_000080 | LG1B | DLAgn_00036550 | LG13 |  | NA |
| DLA_LG1B_000090 | LG1B | DLAgn_00036560 | LG13 |  | NA |
| DLA_LG1B_000100 | LG1B | DLAgn_00036610 | LG13 |  | NA |
| DLA_LG1B_000110 | LG1B | DLAgn_00036640 | LG13 |  | loc569167 protein |
| DLA_LG1B_000120 | LG1B | DLAgn_00036790 | LG13 |  | uncharacterized protein loc101469446 |
| DLA_LG1B_000130 | LG1B | DLAgn_00036920 | LG13 |  | NA |
| DLA_LG1B_000145 | LG1B | DLAgn_00036950 | LG13 |  | NA |
| DLA_LG1B_000150 | LG1B | DLAgn_00046680 | LG14 |  | NA |
| DLA_LG1B_000170 | LG1B | DLAgn_00037160 | LG14 |  | NA |
| DLA_LG1B_000180 | LG1B | DLAgn_00037210 | LG14 |  | zinc finger protein 638 |
| DLA_LG1B_000200 | LG1B | DLAgn_00037230 | LG14 |  | map4 protein |
| DLA_LG1B_000210 | LG1B | DLAgn_00037430 | LG14 |  | NA |
| DLA_LG1B_000220 | LG1B | DLAgn_00037490 | LG14 |  | NA |
| DLA_LG1B_000230 | LG1B | DLAgn_00037620 | LG14 |  | NA |
| DLA_LG1B_000240 | LG1B | DLAgn_00037690 | LG14 |  | g-protein coupled receptor 12-like |
| DLA_LG1B_000250 | LG1B | DLAgn_00037730 | LG14 |  | NA |
| DLA_LG1B_000260 | LG1B | DLAgn_00037990 | LG14 |  | uncharacterized protein loc101158970 |
| DLA_LG1B_000270 | LG1B | DLAgn_00038460 | LG14 |  | early endosome antigen 1 |
| DLA_LG1B_000275 | LG1B | DLAgn_00038490 | LG14 |  | acidic leucine-rich nuclear phosphoprotein 32 family member b |
| DLA_LG1B_000280 | LG1B | DLAgn_00038500 | LG14 |  | transposase |
| DLA_LG1B_000300 | LG1B | DLAgn_00038600 | LG14 |  | histone h2b 1 2-like |
| DLA_LG1B_000330 | LG1B | DLAgn_00038610 | LG14 |  | histone h2a-like |
| DLA_LG1B_000340 | LG1B | DLAgn_00038650 | LG14 |  | nad-specific glutamate dehydrogenase-like isoform 1 |
| DLA_LG1B_000350 | LG1B | DLAgn_00038720 | LG14 |  | opioid-binding protein cell adhesion molecule-like |
| DLA_LG1B_000360 | LG1B | DLAgn_00039420 | LG14 |  | autism susceptibility gene 2 |
| DLA_LG1B_000370 | LG1B | DLAgn_00039480 | LG14 |  | NA |
| DLA_LG1B_000380 | LG1B | DLAgn_00039490 | LG14 |  | uncharacterized protein loc101160452 |
| DLA_LG1B_000390 | LG1B | DLAgn_00039640 | LG14 |  | NA |
| DLA_LG1B_000400 | LG1B | DLAgn_00039860 | LG14 |  | transmembrane protein 93 |
| DLA_LG1B_000410 | LG1B | DLAgn_00039920 | LG14 |  | NA |
| DLA_LG1B_000420 | LG1B | DLAgn_00040070 | LG14 |  | leucine-rich repeat transmembrane protein flrt1 |
| DLA_LG1B_000430 | LG1B | DLAgn_00040080 | LG14 |  | transposable element tcb1 transposase |
| DLA_LG1B_000435 | LG1B | DLAgn_00040350 | LG14 |  | transmembrane protein 88-like |
| DLA_LG1B_000440 | LG1B | DLAgn_00040560 | LG14 |  | gap junction gamma-1 |
| DLA_LG1B_000470 | LG1B | DLAgn_00040710 | LG14 |  | gap junction beta-1 |
| DLA_LG1B_000480 | LG1B | DLAgn_00040720 | LG14 |  | gap junction alpha-3 |
| DLA_LG1B_000490 | LG1B | DLAgn_00040900 | LG14 |  | odorant receptor |
| DLA_LG1B_000500 | LG1B | DLAgn_00041190 | LG14 |  | p2y purinoceptor 10-like |
| DLA_LG1B_000510 | LG1B | DLAgn_00041200 | LG14 |  | lysophosphatidic acid receptor 6-like |
| DLA_LG1B_000520 | LG1B | DLAgn_00041260 | LG14 |  | olfactory receptor 11a1-like |
| DLA_LG1B_000530 | LG1B | DLAgn_00041640 | LG14 |  | histone partial |
| DLA_LG1B_000550 | LG1B | DLAgn_00041840 | LG14 |  | NA |
| DLA_LG1B_000570 | LG1B | DLAgn_00041940 | LG14 |  | voltage-gated potassium channel subunit beta-3-like |
| DLA_LG1B_000580 | LG1B | DLAgn_00042000 | LG14 |  | hypothetical protein [Epinephelus bruneus] |
| DLA_LG1B_000590 | LG1B | DLAgn_00042400 | LG14 |  | NA |
| DLA_LG1B_000600 | LG1B | DLAgn_00042410 | LG14 |  | transcriptional activator protein pur-alpha-like |
| DLA_LG1B_000610 | LG1B | DLAgn_00042620 | LG14 |  | uncharacterized loc338667 |
| DLA_LG1B_000620 | LG1B | DLAgn_00042660 | LG14 |  | u11 u12 snrnp 35k |
| DLA_LG1B_000630 | LG1B | DLAgn_00042670 | LG14 |  | NA |
| DLA_LG1B_000640 | LG1B | DLAgn_00042720 | LG14 |  | NA |
| DLA_LG1B_000650 | LG1B | DLAgn_00042880 | LG14 |  | neurosecretory protein vgf |
| DLA_LG1B_000660 | LG1B | DLAgn_00043530 | LG14 |  | serine-rich and transmembrane domain-containing protein 1-like |
| DLA_LG1B_000670 | LG1B | DLAgn_00043600 | LG14 |  | NA |
| DLA_LG1B_000680 | LG1B | DLAgn_00043700 | LG14 |  | olfactory receptor 1c1-like |
| DLA_LG1B_000690 | LG1B | DLAgn_00043710 | LG14 |  | olfactory receptor 1c1-like |
| DLA_LG1B_000700 | LG1B | DLAgn_00043720 | LG14 |  | olfactory receptor 1c1-like |
| DLA_LG1B_000710 | LG1B | DLAgn_00043730 | LG14 |  | olfactory receptor 1c1-like |
| DLA_LG1B_000720 | LG1B | DLAgn_00043740 | LG14 |  | main olfactory receptor |
| DLA_LG1B_000730 | LG1B | DLAgn_00043750 | LG14 |  | odorant receptor |
| DLA_LG1B_000740 | LG1B | DLAgn_00043760 | LG14 |  | odorant receptor |
| DLA_LG1B_000750 | LG1B | DLAgn_00043770 | LG14 |  | odorant receptor |
| DLA_LG1B_000760 | LG1B | DLAgn_00043790 | LG14 |  | trophoblast glyco |
| DLA_LG1B_000770 | LG1B | DLAgn_00044050 | LG14 |  | claudin b |
| DLA_LG1B_000790 | LG1B | DLAgn_00044060 | LG14 |  | claudin-like protein zf-a89-like |
| DLA_LG1B_000800 | LG1B | DLAgn_00044080 | LG14 |  | claudin-like protein zf-a89-like |
| DLA_LG1B_000810 | LG1B | DLAgn_00044090 | LG14 |  | claudin 4 |
| DLA_LG1B_000815 | LG1B | DLAgn_00044100 | LG14 |  | claudin-like protein zf-a89-like |
| DLA_LG1B_000820 | LG1B | DLAgn_00044110 | LG14 |  | claudin 4 |
| DLA_LG1B_000830 | LG1B | DLAgn_00044120 | LG14 |  | claudin b |
| DLA_LG1B_000840 | LG1B | DLAgn_00044130 | LG14 |  | claudin-3-like |
| DLA_LG1B_000850 | LG1B | DLAgn_00044140 | LG14 |  | claudin 3 |
| DLA_LG1B_000870 | LG1B | DLAgn_00044910 | LG14 |  | d -like dopamine receptor-like |
| DLA_LG1B_000880 | LG1B | DLAgn_00044970 | LG14 |  | 7-alpha-hydroxycholest-4-en-3-one 12-alpha-hydroxylase-like |
| DLA_LG1B_000890 | LG1B | DLAgn_00045020 | LG14 |  | NA |
| DLA_LG1B_000900 | LG1B | DLAgn_00045130 | LG14 |  | ankyrin repeat domain-containing protein sowaha |
| DLA_LG1B_000920 | LG1B | DLAgn_00045260 | LG14 |  | NA |
| DLA_LG1B_000930 | LG1B | DLAgn_00045300 | LG14 |  | ankyrin repeat and sterile alpha motif domain-containing protein 1b-like |
| DLA_LG1B_000940 | LG1B | DLAgn_00045310 | LG14 |  | mhc class i antigen |
| DLA_LG1B_000960 | LG1B | DLAgn_00045580 | LG14 |  | upf0461 protein c5orf24 homolog |
| DLA_LG1B_000970 | LG1B | DLAgn_00045590 | LG14 |  | tripartite motif-containing protein 16-like |
| DLA_LG1B_000980 | LG1B | DLAgn_00046010 | LG14 |  | NA |
| DLA_LG1B_000990 | LG1B | DLAgn_00046260 | LG14 |  | atp-sensitive inward rectifier potassium channel 1-like |
| DLA_LG1B_001000 | LG1B | DLAgn_00046360 | LG14 |  | olfactory marker protein |
| DLA_LG1B_001010 | LG1B | DLAgn_00046970 | LG14 |  | NA |
| DLA_LG1B_001020 | LG1B | DLAgn_00048600 | LG15 |  | nuclear factor ovary-like |
| DLA_LG1B_001030 | LG1B | DLAgn_00047180 | LG15 |  | u3 small nucleolar rna-associated protein 14 homolog a-like |
| DLA_LG1B_001040 | LG1B | DLAgn_00047700 | LG15 |  | muscle ring finger 1 |
| DLA_LG1B_001045 | LG1B | DLAgn_00047740 | LG15 |  | NA |
| DLA_LG1B_001050 | LG1B | DLAgn_00047860 | LG15 | * | pogo transposable element with krab domain-like |
| DLA_LG1B_001060 | LG1B | DLAgn_00047940 | LG15 |  | upf0733 protein c2orf88 homolog |
| DLA_LG1B_001070 | LG1B | DLAgn_00048350 | LG15 |  | gap junction gamma-1 |
| DLA_LG1B_001080 | LG1B | DLAgn_00048400 | LG15 |  | twist-related protein 2 |
| DLA_LG1B_001100 | LG1B | DLAgn_00048460 | LG15 |  | olfactory receptor 52d1-like |
| DLA_LG1B_001110 | LG1B | DLAgn_00048480 | LG15 |  | olfactory receptor 52d1-like |
| DLA_LG1B_001120 | LG1B | DLAgn_00048490 | LG15 |  | olfactory receptor 52d1-like |
| DLA_LG1B_001130 | LG1B | DLAgn_00048670 | LG15 |  | cd59 glyco |
| DLA_LG1B_001140 | LG1B | DLAgn_00048780 | LG15 |  | cyclin-dependent kinase regulatory subunit 2 |
| DLA_LG1B_001150 | LG1B | DLAgn_00049100 | LG15 |  | NA |
| DLA_LG1B_001160 | LG1B | DLAgn_00049120 | LG15 |  | odorant receptor |
| DLA_LG1B_001170 | LG1B | DLAgn_00049130 | LG15 |  | olfactory receptor 2g6-like |
| DLA_LG1B_001180 | LG1B | DLAgn_00049140 | LG15 |  | odorant receptor |
| DLA_LG1B_001190 | LG1B | DLAgn_00049160 | LG15 |  | odorant receptor |
| DLA_LG1B_001200 | LG1B | DLAgn_00049170 | LG15 |  | odorant receptor |
| DLA_LG1B_001210 | LG1B | DLAgn_00049180 | LG15 |  | frizzled homolog 7a |
| DLA_LG1B_001220 | LG1B | DLAgn_00049230 | LG15 |  | btb poz domain-containing protein kctd4 |
| DLA_LG1B_001250 | LG1B | DLAgn_00049410 | LG15 |  | 5-hydroxytryptamine receptor 1f |
| DLA_LG1B_001260 | LG1B | DLAgn_00050150 | LG15 |  | protein reprimo a-like |
| DLA_LG1B_001270 | LG1B | DLAgn_00050310 | LG15 |  | NA |
| DLA_LG1B_001280 | LG1B | DLAgn_00050520 | LG15 |  | beta- -galactosyltransferase 1-like |
| DLA_LG1B_001290 | LG1B | DLAgn_00050570 | LG15 |  | glucose-6-phosphatase partial |
| DLA_LG1B_001300 | LG1B | DLAgn_00050960 | LG15 |  | transcription factor sp9 |
| DLA_LG1B_001310 | LG1B | DLAgn_00051170 | LG15 |  | NA |
| DLA_LG1B_001320 | LG1B | DLAgn_00051190 | LG15 |  | caspase 10 |
| DLA_LG1B_001330 | LG1B | DLAgn_00051220 | LG15 |  | NA |
| DLA_LG1B_001340 | LG1B | DLAgn_00051330 | LG15 |  | NA |
| DLA_LG1B_001350 | LG1B | DLAgn_00051420 | LG15 |  | sry-box containing gene 21 |
| DLA_LG1B_001360 | LG1B | DLAgn_00051460 | LG15 |  | slit and ntrk-like protein 5-like |
| DLA_LG1B_001370 | LG1B | DLAgn_00051480 | LG15 |  | protocadherin-17-like |
| DLA_LG1B_001380 | LG1B | DLAgn_00051710 | LG15 |  | NA |
| DLA_LG1B_001390 | LG1B | DLAgn_00051800 | LG15 |  | helix-loop-helix protein 2 |
| DLA_LG1B_001400 | LG1B | DLAgn_00051930 | LG15 |  | gap junction alpha-8 |
| DLA_LG1B_001410 | LG1B | DLAgn_00051990 | LG15 |  | e3 ubiquitin-protein ligase trim21-like |
| DLA_LG1B_001420 | LG1B | DLAgn_00052010 | LG15 |  | probable g-protein coupled receptor 156-like |
| DLA_LG1B_001430 | LG1B | DLAgn_00052150 | LG15 |  | NA |
| DLA_LG1B_001450 | LG1B | DLAgn_00052170 | LG15 |  | NA |
| DLA_LG1B_001460 | LG1B | DLAgn_00052370 | LG15 |  | transposase |
| DLA_LG1B_001470 | LG1B | DLAgn_00052400 | LG15 |  | NA |
| DLA_LG1B_001480 | LG1B | DLAgn_00052710 | LG15 |  | high-affinity lysophosphatidic acid receptor |
| DLA_LG1B_001490 | LG1B | DLAgn_00052720 | LG15 |  | pou domain protein |
| DLA_LG1B_001500 | LG1B | DLAgn_00052900 | LG15 |  | sry-box containing gene 1a |
| DLA_LG1B_001510 | LG1B | DLAgn_00053030 | LG15 |  | NA |
| DLA_LG1B_001520 | LG1B | DLAgn_00053170 | LG15 |  | NA |
| DLA_LG1B_001530 | LG1B | DLAgn_00053220 | LG15 |  | kelch repeat and btb domain-containing protein 7 |
| DLA_LG1B_001540 | LG1B | DLAgn_00053310 | LG15 |  | potassium channel tetramerisation domain containing |
| DLA_LG1B_001550 | LG1B | DLAgn_00053380 | LG15 |  | NA |
| DLA_LG1B_001560 | LG1B | DLAgn_00053490 | LG15 |  | NA |
| DLA_LG1B_001570 | LG1B | DLAgn_00053590 | LG15 |  | serine threonine-protein phosphatase 2a regulatory subunit b subunit beta isoform x2 |
| DLA_LG1B_001580 | LG1B | DLAgn_00053760 | LG15 |  | NA |
| DLA_LG1B_001595 | LG1B | DLAgn_00053770 | LG15 |  | frizzled homolog 8c |
| DLA_LG1B_001600 | LG1B | DLAgn_00054000 | LG15 |  | NA |
| DLA_LG1B_001610 | LG1B | DLAgn_00054040 | LG15 |  | adenylate cyclase type 5 |
| DLA_LG1B_001620 | LG1B | DLAgn_00054120 | LG15 |  | transposable element tcb2 transposase |
| DLA_LG1B_001630 | LG1B | DLAgn_00054160 | LG15 |  | NA |
| DLA_LG1B_001650 | LG1B | DLAgn_00054330 | LG15 |  | NA |
| DLA_LG1B_001660 | LG1B | DLAgn_00054340 | LG15 |  | NA |
| DLA_LG1B_001670 | LG1B | DLAgn_00054350 | LG15 |  | programmed cell death protein 5 |
| DLA_LG1B_001680 | LG1B | DLAgn_00054370 | LG15 | * | PREDICTED: tetraspanin-5-like [Oreochromis niloticus] |
| DLA_LG1B_001700 | LG1B | DLAgn_00054430 | LG15 |  | adp-ribosylation factor-like protein 4c |
| DLA_LG1B_001710 | LG1B | DLAgn_00054580 | LG15 |  | tripartite motif-containing protein 16-like |
| DLA_LG1B_001715 | LG1B | DLAgn_00054880 | LG15 |  | NA |
| DLA_LG1B_001720 | LG1B | DLAgn_00054980 | LG15 |  | transposase |
| DLA_LG1B_001730 | LG1B | DLAgn_00055330 | LG15 |  | NA |
| DLA_LG1B_001740 | LG1B | DLAgn_00055340 | LG15 |  | protein fam46c |
| DLA_LG1B_001750 | LG1B | DLAgn_00055370 | LG15 |  | zona pellucida-like domain-containing protein 1-like |
| DLA_LG1B_001760 | LG1B | DLAgn_00055570 | LG15 |  | gap junction alpha-3 |
| DLA_LG1B_001780 | LG1B | DLAgn_00055720 | LG16 |  | NA |
| DLA_LG1B_001790 | LG1B | DLAgn_00055770 | LG16 |  | leucine-rich repeat and immunoglobulin-like domain-containing nogo receptor-interacting protein 1-like |
| DLA_LG1B_001800 | LG1B | DLAgn_00055900 | LG16 |  | cortexin-2-like isoform 1 |
| DLA_LG1B_001810 | LG1B | DLAgn_00056240 | LG16 |  | NA |
| DLA_LG1B_001830 | LG1B | DLAgn_00056340 | LG16 |  | tetraspanin-8 isoform 1 |
| DLA_LG1B_001850 | LG1B | DLAgn_00056640 | LG16 |  | uncharacterized protein loc101470778 |
| DLA_LG1B_001870 | LG1B | DLAgn_00056820 | LG16 |  | uncharacterized protein loc101156905 |
| DLA_LG1B_001880 | LG1B | DLAgn_00056840 | LG16 |  | glutaredoxin 3 |
| DLA_LG1B_001890 | LG1B | DLAgn_00056910 | LG16 |  | ring finger protein 183-like |
| DLA_LG1B_001900 | LG1B | DLAgn_00057010 | LG16 |  | apoptosis-associated speck-like protein containing a card |
| DLA_LG1B_001910 | LG1B | DLAgn_00057020 | LG16 |  | potassium voltage-gated channel subfamily e member 1 |
| DLA_LG1B_001920 | LG1B | DLAgn_00057170 | LG16 |  | interferon-inducible gtpase 5-like |
| DLA_LG1B_001940 | LG1B | DLAgn_00057340 | LG16 |  | free fatty acid receptor 2-like |
| DLA_LG1B_001950 | LG1B | DLAgn_00057420 | LG16 |  | protein fam110c-like |
| DLA_LG1B_001960 | LG1B | DLAgn_00057500 | LG16 |  | cannabinoid receptor 2 |
| DLA_LG1B_001980 | LG1B | DLAgn_00057530 | LG16 |  | gap junction alpha-9 protein |
| DLA_LG1B_001990 | LG1B | DLAgn_00057560 | LG16 |  | unnamed protein product [Tetraodon nigroviridis] |
| DLA_LG1B_002010 | LG1B | DLAgn_00058010 | LG16 |  | NA |
| DLA_LG1B_002030 | LG1B | DLAgn_00058390 | LG16 |  | NA |
| DLA_LG1B_002040 | LG1B | DLAgn_00058400 | LG16 |  | NA |
| DLA_LG1B_002050 | LG1B | DLAgn_00058450 | LG16 |  | NA |
| DLA_LG1B_002060 | LG1B | DLAgn_00058460 | LG16 |  | NA |
| DLA_LG1B_002065 | LG1B | DLAgn_00059350 | LG16 |  | uncharacterized protein loc101166494 |
| DLA_LG1B_002065_3 | LG1B | DLAgn_00059370 | LG16 |  | transmembrane protein 238-like |
| DLA_LG1B_002070 | LG1B | DLAgn_00059450 | LG16 |  | NA |
| DLA_LG1B_002090 | LG1B | DLAgn_00059470 | LG16 |  | NA |
| DLA_LG1B_002100 | LG1B | DLAgn_00060280 | LG16 |  | NA |
| DLA_LG1B_002110 | LG1B | DLAgn_00060290 | LG16 |  | NA |
| DLA_LG1B_002130 | LG1B | DLAgn_00061120 | LG16 |  | NA |
| DLA_LG1B_002140 | LG1B | DLAgn_00061450 | LG16 |  | e3 ubiquitin-protein ligase nhlrc1 |
| DLA_LG1B_002150 | LG1B | DLAgn_00061570 | LG16 |  | NA |
| DLA_LG1B_002170 | LG1B | DLAgn_00061770 | LG16 |  | tpa_inf: twist1a |
| DLA_LG1B_002180 | LG1B | DLAgn_00062320 | LG16 | * | NA |
| DLA_LG1B_002190 | LG1B | DLAgn_00062440 | LG16 |  | NA |
| DLA_LG1B_002200 | LG1B | DLAgn_00062530 | LG16 |  | zinc finger protein 34 |
| DLA_LG1B_002210 | LG1B | DLAgn_00062600 | LG16 |  | membrane progestin receptor alpha |
| DLA_LG1B_002220 | LG1B | DLAgn_00062870 | LG16 |  | transmembrane protein 158-like |
| DLA_LG1B_002250 | LG1B | DLAgn_00062890 | LG16 |  | NA |
| DLA_LG1B_002260 | LG1B | DLAgn_00062900 | LG16 |  | NA |
| DLA_LG1B_002270 | LG1B | DLAgn_00063100 | LG16 |  | tripartite motif-containing protein 39-like |
| DLA_LG1B_002280 | LG1B | DLAgn_00063110 | LG16 |  | tripartite motif-containing protein 39-like |
| DLA_LG1B_002295 | LG1B | DLAgn_00063240 | LG16 |  | sry (sex determining region y)-box 4 |
| DLA_LG1B_002300 | LG1B | DLAgn_00063450 | LG16 |  | c-c chemokine receptor type 4-like |
| DLA_LG1B_002310 | LG1B | DLAgn_00063520 | LG16 |  | NA |
| DLA_LG1B_002320 | LG1B | DLAgn_00063600 | LG16 |  | v-set domain-containing t-cell activation inhibitor 1-like |
| DLA_LG1B_002330 | LG1B | DLAgn_00063750 | LG16 |  | malate mitochondrial-like |
| DLA_LG1B_002340 | LG1B | DLAgn_00063780 | LG16 |  | actin-binding rho-activating |
| DLA_LG1B_002360 | LG1B | DLAgn_00064340 | LG16 |  | transmembrane protein 74 |
| DLA_LG1B_002370 | LG1B | DLAgn_00064440 | LG16 |  | trophoblast glyco |
| DLA_LG1B_002380 | LG1B | DLAgn_00064600 | LG16 |  | pou class transcription factor 2-like |
| DLA_LG1B_002390 | LG1B | DLAgn_00064730 | LG16 |  | NA |
| DLA_LG1B_002400 | LG1B | DLAgn_00065000 | LG16 |  | NA |
| DLA_LG1B_002410 | LG1B | DLAgn_00065030 | LG16 | *** | transposase |
| DLA_LG1B_002430 | LG1B | DLAgn_00065320 | LG16 |  | NA |
| DLA_LG1B_002440 | LG1B | DLAgn_00065330 | LG16 |  | frizzled-1 precursor |
| DLA_LG1B_002450 | LG1B | DLAgn_00065440 | LG16 |  | uncharacterized |
| DLA_LG1B_002460 | LG1B | DLAgn_00065450 | LG16 |  | general transcription factor ii-i repeat domain-containing protein 2-like |
| DLA_LG1B_002470 | LG1B | DLAgn_00065490 | LG16 |  | transposase |
| DLA_LG1B_002480 | LG1B | DLAgn_00065510 | LG16 |  | uncharacterized protein loc101466409 isoform x1 |
| DLA_LG1B_002490 | LG1B | DLAgn_00065540 | LG16 |  | uncharacterized protein loc101165865 |
| DLA_LG1B_002500 | LG1B | DLAgn_00065650 | LG16 |  | transposase |
| DLA_LG1B_002510 | LG1B | DLAgn_00065720 | LG16 |  | quinone oxidoreductase pig3-like |
| DLA_LG1B_002530 | LG1B | DLAgn_00065830 | LG16 |  | retrotransposable element tf2 155 kda protein type 1-like |
| DLA_LG1B_002540 | LG1B | DLAgn_00065840 | LG16 |  | uncharacterized protein loc101467069 |
| DLA_LG1B_002550 | LG1B | DLAgn_00065930 | LG17 |  | 5-hydroxytryptamine receptor 1e |
| DLA_LG1B_002560 | LG1B | DLAgn_00065950 | LG17 |  | gap junction beta-7 |
| DLA_LG1B_002580 | LG1B | DLAgn_00066030 | LG17 |  | NA |
| DLA_LG1B_002590 | LG1B | DLAgn_00066180 | LG17 |  | NA |
| DLA_LG1B_002600 | LG1B | DLAgn_00066300 | LG17 | * | gamma-crystallin m3-like |
| DLA_LG1B_002610 | LG1B | DLAgn_00066310 | LG17 | * | gamma-crystallin m3-like |
| DLA_LG1B_002620 | LG1B | DLAgn_00066320 | LG17 |  | daple_xenla ame: full=daple-like protein ame: full=coiled-coil domain-containing protein 88c-like ame: full=dvl-associating protein with a high frequency of leucine residues-like short=xdal |
| DLA_LG1B_002630 | LG1B | DLAgn_00066430 | LG17 |  | zinc finger bed domain-containing protein 1-like |
| DLA_LG1B_002650 | LG1B | DLAgn_00066450 | LG17 |  | NA |
| DLA_LG1B_002660 | LG1B | DLAgn_00066490 | LG17 |  | NA |
| DLA_LG1B_002665 | LG1B | DLAgn_00066530 | LG17 |  | uncharacterized protein loc101162800 |
| DLA_LG1B_002670 | LG1B | DLAgn_00066760 | LG17 |  | alpha- -mannosyl-glycoprotein 2-beta-n-acetylglucosaminyltransferase |
| DLA_LG1B_002680 | LG1B | DLAgn_00066780 | LG17 |  | thrombomodulin precursor |
| DLA_LG1B_002690 | LG1B | DLAgn_00066940 | LG17 |  | synaptosomal-associated protein 25-like isoform 2 |
| DLA_LG1B_002700 | LG1B | DLAgn_00067000 | LG17 |  | NA |
| DLA_LG1B_002710 | LG1B | DLAgn_00067040 | LG17 | * | gtpase imap family member 7-like |
| DLA_LG1B_002720 | LG1B | DLAgn_00067120 | LG17 |  | prkr-interacting protein 1 homolog |
| DLA_LG1B_002730 | LG1B | DLAgn_00067400 | LG17 | * | cytosolic carboxypeptidase-like protein 5-like |
| DLA_LG1B_002740 | LG1B | DLAgn_00067420 | LG17 |  | dolichyl-diphosphooligosaccharide--protein glycosyltransferase subunit 4 |
| DLA_LG1B_002760 | LG1B | DLAgn_00067790 | LG17 |  | vomeronasal type 1 receptor 6 |
| DLA_LG1B_002770 | LG1B | DLAgn_00068090 | LG17 |  | type iii iodothyronine deiodinase |
| DLA_LG1B_002780 | LG1B | DLAgn_00068340 | LG17 |  | band -like protein 2-like |
| DLA_LG1B_002790 | LG1B | DLAgn_00068430 | LG17 |  | NA |
| DLA_LG1B_002800 | LG1B | DLAgn_00068570 | LG17 |  | c-c chemokine receptor type 4-like |
| DLA_LG1B_002810 | LG1B | DLAgn_00068640 | LG17 |  | pou class transcription factor 2-like |
| DLA_LG1B_002820 | LG1B | DLAgn_00068840 | LG17 |  | NA |
| DLA_LG1B_002830 | LG1B | DLAgn_00068860 | LG17 |  | uncharacterized protein encoded by linc00116-like |
| DLA_LG1B_002860 | LG1B | DLAgn_00069040 | LG17 |  | complement c1q tumor necrosis factor-related protein 3-like |
| DLA_LG1B_002880 | LG1B | DLAgn_00069550 | LG17 |  | unnamed protein product [Tetraodon nigroviridis] |
| DLA_LG1B_002890 | LG1B | DLAgn_00069680 | LG17 |  | unnamed protein product [Tetraodon nigroviridis] |
| DLA_LG1B_002910 | LG1B | DLAgn_00069710 | LG17 |  | NA |
| DLA_LG1B_002920 | LG1B | DLAgn_00069850 | LG17 |  | zinc finger mym-type protein 1 |
| DLA_LG1B_002930 | LG1B | DLAgn_00069870 | LG17 |  | zinc finger bed domain-containing protein 4-like |
| DLA_LG1B_002940 | LG1B | DLAgn_00070180 | LG17 |  | gremlin-1-like |
| DLA_LG1B_002950 | LG1B | DLAgn_00070230 | LG17 |  | muscarinic acetylcholine receptor m5 |
| DLA_LG1B_002960 | LG1B | DLAgn_00070350 | LG17 |  | transposable element tcb2 transposase |
| DLA_LG1B_002980 | LG1B | DLAgn_00070650 | LG17 |  | transmembrane protein 121-like |
| DLA_LG1B_002990 | LG1B | DLAgn_00070860 | LG17 |  | NA |
| DLA_LG1B_003000 | LG1B | DLAgn_00070940 | LG17 |  | NA |
| DLA_LG1B_003010 | LG1B | DLAgn_00071100 | LG17 |  | potassium voltage-gated channel subfamily s member 3-like |
| DLA_LG1B_003020 | LG1B | DLAgn_00071180 | LG17 |  | rho-related gtp-binding protein |
| DLA_LG1B_003030 | LG1B | DLAgn_00071240 | LG17 |  | potassium voltage-gated channel subfamily f member 1 |
| DLA_LG1B_003040 | LG1B | DLAgn_00071260 | LG17 |  | leucine-rich alpha-2-glycoprotein |
| DLA_LG1B_003050 | LG1B | DLAgn_00071690 | LG17 |  | mesogenin 1 |
| DLA_LG1B_003060 | LG1B | DLAgn_00071760 | LG17 |  | loc569167 protein |
| DLA_LG1B_003070 | LG1B | DLAgn_00071850 | LG17 |  | syntaxin-11-like isoform x1 |
| DLA_LG1B_003090 | LG1B | DLAgn_00071860 | LG17 |  | syntaxin-11-like isoform x1 |
| DLA_LG1B_003100 | LG1B | DLAgn_00072060 | LG17 |  | g-protein coupled receptor 6-like |
| DLA_LG1B_003110 | LG1B | DLAgn_00072160 | LG17 |  | protein fam110c-like |
| DLA_LG1B_003120 | LG1B | DLAgn_00072230 | LG17 |  | insulinoma-associated protein 1-like |
| DLA_LG1B_003130 | LG1B | DLAgn_00072290 | LG17 |  | nuclear factor ovary-like |
| DLA_LG1B_003140 | LG1B | DLAgn_00072420 | LG17 |  | NA |
| DLA_LG1B_003145 | LG1B | DLAgn_00072540 | LG17 |  | NA |
| DLA_LG1B_003160 | LG1B | DLAgn_00072800 | LG17 |  | reverse transcriptase-like protein |
| DLA_LG1B_003170 | LG1B | DLAgn_00073270 | LG17 |  | urotensin i precursor |
| DLA_LG1B_003180 | LG1B | DLAgn_00073330 | LG17 |  | prostaglandin e2 receptor ep2 subtype-like |
| DLA_LG1B_003190 | LG1B | DLAgn_00073460 | LG17 |  | gag-like protein |
| DLA_LG1B_003200 | LG1B | DLAgn_00073470 | LG17 |  | transposon tx1 uncharacterized 149 kda |
| DLA_LG1B_003210 | LG1B | DLAgn_00073500 | LG17 |  | 5-hydroxytryptamine receptor 1b-like |
| DLA_LG1B_003220 | LG1B | DLAgn_00073680 | LG17 |  | claudin-20-like |
| DLA_LG1B_003230 | LG1B | DLAgn_00073750 | LG17 |  | NA |
| DLA_LG1B_003240 | LG1B | DLAgn_00079090 | LG19 |  | tripartite motif-containing protein 16-like |
| DLA_LG1B_003250 | LG1B | DLAgn_00079100 | LG19 |  | tripartite motif-containing protein 16-like |
| DLA_LG1B_003260 | LG1B | DLAgn_00079120 | LG19 |  | uncharacterized protein loc101733467 |
| DLA_LG1B_003270 | LG1B | DLAgn_00079140 | LG19 |  | tripartite motif-containing protein 16-like |
| DLA_LG1B_003280 | LG1B | DLAgn_00079150 | LG19 |  | e3 ubiquitin isg15 ligase trim25-like |
| DLA_LG1B_003290 | LG1B | DLAgn_00079180 | LG19 |  | NA |
| DLA_LG1B_003300 | LG1B | DLAgn_00079360 | LG19 | * | gtpase imap family member 7-like |
| DLA_LG1B_003310 | LG1B | DLAgn_00079390 | LG19 |  | NA |
| DLA_LG1B_003320 | LG1B | DLAgn_00079520 | LG19 |  | transcription factor jun-d |
| DLA_LG1B_003330 | LG1B | DLAgn_00079640 | LG19 |  | transposase |
| DLA_LG1B_003350 | LG1B | DLAgn_00079650 | LG19 |  | NA |
| DLA_LG1B_003355 | LG1B | DLAgn_00079780 | LG19 |  | NA |
| DLA_LG1B_003360 | LG1B | DLAgn_00079830 | LG19 |  | NA |
| DLA_LG1B_003370 | LG1B | DLAgn_00079990 | LG19 |  | NA |
| DLA_LG1B_003380 | LG1B | DLAgn_00080010 | LG19 |  | NA |
| DLA_LG1B_003390 | LG1B | DLAgn_00080100 | LG19 |  | uncharacterized protein c9orf172 homolog |
| DLA_LG1B_003400 | LG1B | DLAgn_00080140 | LG19 |  | PREDICTED: hypothetical protein LOC798447 [Danio rerio] |
| DLA_LG1B_003410 | LG1B | DLAgn_00080150 | LG19 |  | teashirt homolog 3-like |
| DLA_LG1B_003420 | LG1B | DLAgn_00080340 | LG19 |  | uncharacterized protein |
| DLA_LG1B_003430 | LG1B | DLAgn_00080420 | LG19 |  | purine-rich element-binding protein gamma-like |
| DLA_LG1B_003440 | LG1B | DLAgn_00080630 | LG19 |  | alpha-2b adrenergic receptor-like |
| DLA_LG1B_003450 | LG1B | DLAgn_00080670 | LG19 |  | piggybac transposable element-derived protein 4-like |
| DLA_LG1B_003455 | LG1B | DLAgn_00080720 | LG19 |  | NA |
| DLA_LG1B_003460 | LG1B | DLAgn_00080780 | LG19 | * | tpa: os05g0242100-like |
| DLA_LG1B_003470 | LG1B | DLAgn_00080790 | LG19 |  | tpa: os05g0242100-like |
| DLA_LG1B_003480 | LG1B | DLAgn_00081010 | LG19 |  | NA |
| DLA_LG1B_003490 | LG1B | DLAgn_00081120 | LG19 |  | ankyrin repeat domain-containing protein 34b-like |
| DLA_LG1B_003500 | LG1B | DLAgn_00081170 | LG19 |  | NA |
| DLA_LG1B_003510 | LG1B | DLAgn_00081210 | LG19 |  | reverse transcriptase-like protein |
| DLA_LG1B_003520 | LG1B | DLAgn_00081250 | LG19 |  | NA |
| DLA_LG1B_003530 | LG1B | DLAgn_00081350 | LG19 |  | hydroxycarboxylic acid receptor 2-like |
| DLA_LG1B_003550 | LG1B | DLAgn_00081500 | LG19 |  | NA |
| DLA_LG1B_003560 | LG1B | DLAgn_00081510 | LG19 |  | NA |
| DLA_LG1B_003570 | LG1B | DLAgn_00081520 | LG19 |  | NA |
| DLA_LG1B_003590 | LG1B | DLAgn_00081640 | LG19 |  | unnamed protein product [Tetraodon nigroviridis] |
| DLA_LG1B_003610 | LG1B | DLAgn_00081750 | LG19 |  | cholesterol 25-hydroxylase-like protein member 2-like |
| DLA_LG1B_003620 | LG1B | DLAgn_00081830 | LG19 |  | NA |
| DLA_LG1B_003650 | LG1B | DLAgn_00081850 | LG19 |  | NA |
| DLA_LG1B_003660 | LG1B | DLAgn_00082160 | LG19 |  | growth arrest-specific protein 1-like |
| DLA_LG1B_003670 | LG1B | DLAgn_00082260 | LG19 |  | forkhead box protein d1-like |
| DLA_LG1B_003680 | LG1B | DLAgn_00082550 | LG19 |  | NA |
| DLA_LG1B_003690 | LG1B | DLAgn_00082600 | LG19 |  | NA |
| DLA_LG1B_003700 | LG1B | DLAgn_00083060 | LG19 |  | glycoprotein gp2-like isoform x1 |
| DLA_LG1B_003705 | LG1B | DLAgn_00083070 | LG19 |  | NA |
| DLA_LG1B_003705_2 | LG1B | DLAgn_00083250 | LG19 |  | relaxin-3 receptor 1 |
| DLA_LG1B_003720 | LG1B | DLAgn_00083500 | LG19 |  | uro-adherence factor a-like |
| DLA_LG1B_003730 | LG1B | DLAgn_00083530 | LG19 |  | e3 ubiquitin-protein ligase nedd4-like isoform x1 |
| DLA_LG1B_003740 | LG1B | DLAgn_00083640 | LG19 |  | NA |
| DLA_LG1B_003760 | LG1B | DLAgn_00083700 | LG19 |  | NA |
| DLA_LG1B_003770 | LG1B | DLAgn_00083810 | LG19 |  | uncharacterized protein |
| DLA_LG1B_003780 | LG1B | DLAgn_00083930 | LG19 |  | myosin light chain 4-like |
| DLA_LG1B_003800 | LG1B | DLAgn_00084000 | LG19 |  | unnamed protein product [Tetraodon nigroviridis] |
| DLA_LG1B_003820 | LG1B | DLAgn_00084190 | LG19 |  | NA |
| DLA_LG1B_003830 | LG1B | DLAgn_00084200 | LG19 |  | proteinase-activated receptor 4-like |
| DLA_LG1B_003840 | LG1B | DLAgn_00084460 | LG19 |  | vomeronasal type 1 receptor 5 |
| DLA_LG1B_003850 | LG1B | DLAgn_00085090 | LG19 |  | tpa: shadow 2 precursor |
| DLA_LG1B_003860 | LG1B | DLAgn_00085120 | LG19 |  | NA |
| DLA_LG1B_003870 | LG1B | DLAgn_00085140 | LG19 |  | apelin receptor a |
| DLA_LG1B_003890 | LG1B | DLAgn_00085380 | LG19 |  | NA |
| DLA_LG1B_003900 | LG1B | DLAgn_00085670 | LG19 |  | NA |
| DLA_LG1B_003920 | LG1B | DLAgn_00085880 | LG19 |  | alpha- -fucosyltransferase |
| DLA_LG1B_003930 | LG1B | DLAgn_00085960 | LG19 |  | NA |
| DLA_LG1B_003940 | LG1B | DLAgn_00086250 | LG19 |  | claudin-23-like |
| DLA_LG1B_003975 | LG1B | DLAgn_00086440 | LG19 |  | udp- c:betagal beta- -n-acetylglucosaminyltransferase 2-like |
| DLA_LG1B_003980 | LG1B | DLAgn_00086590 | LG19 |  | heat shock protein 30 |
| DLA_LG1B_003990 | LG1B | DLAgn_00086720 | LG19 |  | NA |
| DLA_LG1B_004000 | LG1B | DLAgn_00086780 | LG19 |  | NA |
| DLA_LG1B_004010 | LG1B | DLAgn_00086880 | LG19 |  | unnamed protein product [Tetraodon nigroviridis] |
| DLA_LG1B_004020 | LG1B | DLAgn_00086970 | LG19 |  | e3 ubiquitin-protein ligase rnf182-like |
| DLA_LG1B_004030 | LG1B | DLAgn_00087190 | LG19 |  | NA |
| DLA_LG1B_004040 | LG1B | DLAgn_00087210 | LG19 |  | prepromelanin concentrating hormone |
| DLA_LG1B_004060 | LG1B | DLAgn_00087420 | LG19 |  | 5-hydroxytryptamine receptor 1a-beta-like |
| DLA_LG1B_004065 | LG1B | DLAgn_00087430 | LG19 |  | inverted formin-2-like |
| DLA_LG1B_004070 | LG1B | DLAgn_00087510 | LG19 |  | tripartite motif-containing protein 16-like |
| DLA_LG1B_004090 | LG1B | DLAgn_00087520 | LG19 |  | tripartite motif-containing protein 16-like |
| DLA_LG1B_004100 | LG1B | DLAgn_00087540 | LG19 |  | tripartite motif-containing protein 16-like |
| DLA_LG1B_004110 | LG1B | DLAgn_00115260 | LG20 |  | NA |
| DLA_LG1B_004120 | LG1B | DLAgn_00114730 | LG20 |  | NA |
| DLA_LG1B_004130 | LG1B | DLAgn_00114800 | LG20 |  | leucine-rich repeat transmembrane neuronal protein 4-like |
| DLA_LG1B_004160 | LG1B | DLAgn_00114810 | LG20 |  | leucine-rich repeat transmembrane neuronal protein 4-like |
| DLA_LG1B_004170 | LG1B | DLAgn_00115060 | LG20 |  | frizzled homolog 10 |
| DLA_LG1B_004180 | LG1B | DLAgn_00115120 | LG20 |  | immediate early response gene 5-like |
| DLA_LG1B_004190 | LG1B | DLAgn_00115290 | LG20 |  | unnamed protein product [Tetraodon nigroviridis] |
| DLA_LG1B_004220 | LG1B | DLAgn_00115320 | LG20 |  | uncharacterized protein c9orf172 homolog |
| DLA_LG1B_004230 | LG1B | DLAgn_00115430 | LG20 |  | apelin receptor a |
| DLA_LG1B_004240 | LG1B | DLAgn_00115530 | LG20 |  | non-pou domain-containing octamer-binding |
| DLA_LG1B_004245 | LG1B | DLAgn_00115780 | LG20 |  | NA |
| DLA_LG1B_004260 | LG1B | DLAgn_00115980 | LG20 |  | unnamed protein product [Tetraodon nigroviridis] |
| DLA_LG1B_004270 | LG1B | DLAgn_00115990 | LG20 |  | dual specificity protein phosphatase 18 |
| DLA_LG1B_004280 | LG1B | DLAgn_00116190 | LG20 |  | transmembrane protein 119 |
| DLA_LG1B_004310 | LG1B | DLAgn_00116250 | LG20 |  | interferon-induced 17 kda protein precursor |
| DLA_LG1B_004320 | LG1B | DLAgn_00117180 | LG20 |  | piggybac transposase uribo2 |
| DLA_LG1B_004330 | LG1B | DLAgn_00117460 | LG20 |  | NA |
| DLA_LG1B_004340 | LG1B | DLAgn_00117710 | LG20 |  | NA |
| DLA_LG1B_004350 | LG1B | DLAgn_00117770 | LG20 |  | NA |
| DLA_LG1B_004360 | LG1B | DLAgn_00117850 | LG20 |  | NA |
| DLA_LG1B_004370 | LG1B | DLAgn_00118060 | LG20 |  | beta-4c adrenergic receptor-like |
| DLA_LG1B_004380 | LG1B | DLAgn_00118140 | LG20 |  | NA |
| DLA_LG1B_004390 | LG1B | DLAgn_00118470 | LG20 |  | NA |
| DLA_LG1B_004400 | LG1B | DLAgn_00118530 | LG20 |  | hydroxycarboxylic acid receptor 2 |
| DLA_LG1B_004410 | LG1B | DLAgn_00118540 | LG20 |  | hydroxycarboxylic acid receptor 2-like |
| DLA_LG1B_004430 | LG1B | DLAgn_00118560 | LG20 |  | hydroxycarboxylic acid receptor 3 |
| DLA_LG1B_004440 | LG1B | DLAgn_00118760 | LG20 |  | forkhead box protein b2 |
| DLA_LG1B_004460 | LG1B | DLAgn_00118770 | LG20 |  | NA |
| DLA_LG1B_004470 | LG1B | DLAgn_00118780 | LG20 |  | c2 calcium-dependent domain-containing protein 4c-like |
| DLA_LG1B_004500 | LG1B | DLAgn_00119260 | LG20 |  | histone h1c-like isoform x1 |
| DLA_LG1B_004505 | LG1B | DLAgn_00119400 | LG20 | * | transposase |
| DLA_LG1B_004510 | LG1B | DLAgn_00119420 | LG20 |  | NA |
| DLA_LG1B_004520 | LG1B | DLAgn_00119430 | LG20 |  | upf0542 protein c5orf43 homolog |
| DLA_LG1B_004540 | LG1B | DLAgn_00119470 | LG20 |  | forkhead box protein d5-a-like |
| DLA_LG1B_004550 | LG1B | DLAgn_00119560 | LG20 |  | 5-hydroxytryptamine receptor 1a-beta-like |
| DLA_LG1B_004560 | LG1B | DLAgn_00119980 | LG20 |  | NA |
| DLA_LG1B_004570 | LG1B | DLAgn_00120070 | LG20 |  | centrosomal protein of 78 kda-like isoform x1 |
| DLA_LG1B_004580 | LG1B | DLAgn_00120240 | LG20 |  | uncharacterized protein loc101478033 |
| DLA_LG1B_004590 | LG1B | DLAgn_00120370 | LG20 |  | NA |
| DLA_LG1B_004600 | LG1B | DLAgn_00120390 | LG20 |  | prion protein |
| DLA_LG1B_004610 | LG1B | DLAgn_00120420 | LG20 |  | NA |
| DLA_LG1B_004620 | LG1B | DLAgn_00120450 | LG20 |  | claudin-5-like |
| DLA_LG1B_004630 | LG1B | DLAgn_00120480 | LG20 |  | platelet glycoprotein ib beta chain-like |
| DLA_LG1B_004640 | LG1B | DLAgn_00120680 | LG20 |  | hypermethylated in cancer 2 |
| DLA_LG1B_004650 | LG1B | DLAgn_00120700 | LG20 |  | melanocortin-2 receptor |
| DLA_LG1B_004660 | LG1B | DLAgn_00121130 | LG20 |  | growth arrest-specific protein 1-like |
| DLA_LG1B_004670 | LG1B | DLAgn_00121520 | LG20 | * | udp-glucuronosyltransferase 2a2-like |
| DLA_LG1B_004680 | LG1B | DLAgn_00121540 | LG20 |  | t-cell acute lymphocytic leukemia protein 2 |
| DLA_LG1B_004690 | LG1B | DLAgn_00121580 | LG20 |  | PREDICTED: titin-like [Maylandia zebra] |
| DLA_LG1B_004700 | LG1B | DLAgn_00121830 | LG20 |  | NA |
| DLA_LG1B_004710 | LG1B | DLAgn_00122140 | LG20 |  | zinc-binding protein a33-like |
| DLA_LG1B_004720 | LG1B | DLAgn_00122170 | LG20 |  | transcriptional activator protein pur-beta-like |
| DLA_LG1B_004730 | LG1B | DLAgn_00122340 | LG20 | * | transposable element tc1 transposase |
| DLA_LG1B_004740 | LG1B | DLAgn_00122350 | LG20 |  | major histocompatibility complex class i-related gene |
| DLA_LG1B_004760 | LG1B | DLAgn_00122410 | LG20 |  | zinc-binding protein a33-like |
| DLA_LG1B_004770 | LG1B | DLAgn_00122420 | LG20 |  | NA |
| DLA_LG1B_004780 | LG1B | DLAgn_00122600 | LG20 |  | NA |
| DLA_LG1B_004790 | LG1B | DLAgn_00122660 | LG20 |  | NA |
| DLA_LG1B_004800 | LG1B | DLAgn_00122920 | LG20 |  | probable g-protein coupled receptor 21 |
| DLA_LG1B_004810 | LG1B | DLAgn_00122950 | LG20 |  | NA |
| DLA_LG1B_004820 | LG1B | DLAgn_00122960 | LG20 |  | udp-glucuronosyltransferase 2a2-like |
| DLA_LG1B_004830 | LG1B | DLAgn_00122970 | LG20 |  | udp-glucuronosyltransferase 2a2-like |
| DLA_LG1B_004840 | LG1B | DLAgn_00123020 | LG20 |  | natterin-3-like |
| DLA_LG1B_004850 | LG1B | DLAgn_00123030 | LG20 |  | natterin-3-like |
| DLA_LG1B_004860 | LG1B | DLAgn_00123040 | LG20 |  | natterin-3-like |
| DLA_LG1B_004870 | LG1B | DLAgn_00123240 | LG20 |  | radiation-inducible immediate-early gene iex-1 |
| DLA_LG1B_004880 | LG1B | DLAgn_00123330 | LG20 |  | serine-rich adhesin for platelets-like isoform x1 |
| DLA_LG1B_004885 | LG1B | DLAgn_00123590 | LG20 |  | NA |
| DLA_LG1B_004890 | LG1B | DLAgn_00123610 | LG20 |  | NA |
| DLA_LG1B_004900 | LG1B | DLAgn_00123740 | LG20 |  | zinc finger and btb domain-containing protein 26-like |
| DLA_LG1B_004910 | LG1B | DLAgn_00123910 | LG20 |  | NA |
| DLA_LG1B_004920 | LG1B | DLAgn_00124080 | LG20 |  | uncharacterized protein c8orf4 homolog |
| DLA_LG1B_004930 | LG1B | DLAgn_00124100 | LG20 |  | NA |
| DLA_LG1B_004940 | LG1B | DLAgn_00124250 | LG20 |  | NA |
| DLA_LG1B_004960 | LG1B | DLAgn_00124260 | LG20 |  | NA |
| DLA_LG1B_004970 | LG1B | DLAgn_00124500 | LG20 |  | alpha-( )-fucosyltransferase-like |
| DLA_LG1B_004980 | LG1B | DLAgn_00124620 | LG20 |  | p2y purinoceptor 2 |
| DLA_LG1B_004990 | LG1B | DLAgn_00124650 | LG20 |  | NA |
| DLA_LG1B_005000 | LG1B | DLAgn_00124780 | LG20 |  | NA |
| DLA_LG1B_005005 | LG1B | DLAgn_00124800 | LG20 |  | zgc:161969 protein |
| DLA_LG1B_005030 | LG1B | DLAgn_00124850 | LG20 |  | NA |
| DLA_LG1B_005050 | LG1B | DLAgn_00124890 | LG20 |  | transposable element tcb1 transposase |
| DLA_LG1B_005060 | LG1B | DLAgn_00124980 | LG20 |  | reverse transcriptase-like protein |
| DLA_LG1B_005070 | LG1B | DLAgn_00124990 | LG20 |  | uncharacterized protein loc101066806 |
| DLA_LG1B_005080 | LG1B | DLAgn_00125010 | LG20 |  | beta- -galactosyltransferase 2-like |
| DLA_LG1B_005100 | LG1B | DLAgn_00125080 | LG20 |  | NA |
| DLA_LG1B_005110 | LG1B | DLAgn_00125140 | LG20 |  | junction plakoglobin |
| DLA_LG1B_005120 | LG1B | DLAgn_00136170 | LG24 |  | inhibin beta b chain-like |
| DLA_LG1B_005130 | LG1B | DLAgn_00136180 | LG24 |  | NA |
| DLA_LG1B_005150 | LG1B | DLAgn_00136460 | LG24 | * | adp-ribosylation factor-like protein 4c-like |
| DLA_LG1B_005160 | LG1B | DLAgn_00136520 | LG24 |  | NA |
| DLA_LG1B_005170 | LG1B | DLAgn_00136530 | LG24 |  | insulin-like growth factor-binding protein complex acid labile subunit-like |
| DLA_LG1B_005180 | LG1B | DLAgn_00136610 | LG24 |  | udp- c:betagal beta- -n-acetylglucosaminyltransferase 3-like |
| DLA_LG1B_005190 | LG1B | DLAgn_00136740 | LG24 |  | NA |
| DLA_LG1B_005200 | LG1B | DLAgn_00136950 | LG24 |  | NA |
| DLA_LG1B_005210 | LG1B | DLAgn_00136980 | LG24 |  | leucine-rich repeat-containing protein 3-like |
| DLA_LG1B_005220 | LG1B | DLAgn_00137540 | LG24 |  | NA |
| DLA_LG1B_005240 | LG1B | DLAgn_00137670 | LG24 |  | trace amine-associated receptor 6-like |
| DLA_LG1B_005250 | LG1B | DLAgn_00137830 | LG24 |  | transcription elongation factor a n-terminal and central domain-containing protein isoform 1 |
| DLA_LG1B_005260 | LG1B | DLAgn_00137840 | LG24 |  | thymosin beta-12 |
| DLA_LG1B_005270 | LG1B | DLAgn_00137950 | LG24 | * | transposable element tc1 transposase |
| DLA_LG1B_005280 | LG1B | DLAgn_00138090 | LG24 |  | nuclear fragile x mental retardation-interacting protein 1-like |
| DLA_LG1B_005300 | LG1B | DLAgn_00138100 | LG24 |  | NA |
| DLA_LG1B_005310 | LG1B | DLAgn_00138130 | LG24 |  | NA |
| DLA_LG1B_005320 | LG1B | DLAgn_00138140 | LG24 |  | NA |
| DLA_LG1B_005330 | LG1B | DLAgn_00138180 | LG24 |  | NA |
| DLA_LG1B_005340 | LG1B | DLAgn_00138200 | LG24 |  | dedicator of cytokinesis protein 9-like isoform x1 |
| DLA_LG1B_005350 | LG1B | DLAgn_00138300 | LG24 |  | fidgetin-like |
| DLA_LG1B_005360 | LG1B | DLAgn_00138350 | LG24 |  | beta- -galactosyltransferase 1-like |
| DLA_LG1B_005370 | LG1B | DLAgn_00138540 | LG24 |  | isoform cra_a |
| DLA_LG1B_005390 | LG1B | DLAgn_00138770 | LG24 |  | general transcription factor ii-i repeat domain-containing protein 2-like |
| DLA_LG1B_005410 | LG1B | DLAgn_00138780 | LG24 |  | NA |
| DLA_LG1B_005420 | LG1B | DLAgn_00139090 | LG24 |  | protein sprouty homolog 2 |
| DLA_LG1B_005430 | LG1B | DLAgn_00139100 | LG24 |  | slit and ntrk-like protein 1-like |
| DLA_LG1B_005450 | LG1B | DLAgn_00139120 | LG24 |  | slit and ntrk-like protein 5-like |
| DLA_LG1B_005460 | LG1B | DLAgn_00139170 | LG24 |  | rho gtpase-activating protein 6-like |
| DLA_LG1B_005470 | LG1B | DLAgn_00139320 | LG24 |  | NA |
| DLA_LG1B_005480 | LG1B | DLAgn_00139420 | LG24 |  | NA |
| DLA_LG1B_005490 | LG1B | DLAgn_00139700 | LG24 |  | transcription factor sox-1a-like |
| DLA_LG1B_005510 | LG1B | DLAgn_00139950 | LG24 |  | NA |
| DLA_LG1B_005520 | LG1B | DLAgn_00139960 | LG24 |  | cardiac phospholamban-like |
| DLA_LG1B_005525 | LG1B | DLAgn_00139990 | LG24 |  | NA |
| DLA_LG1B_005530 | LG1B | DLAgn_00140210 | LG24 | * | immunoglobulin light chain precursor |
| DLA_LG1B_005550 | LG1B | DLAgn_00140230 | LG24 | * | trace amine-associated receptor 1-like |
| DLA_LG1B_005560 | LG1B | DLAgn_00140240 | LG24 |  | trace amine-associated receptor 1-like |
| DLA_LG1B_005570 | LG1B | DLAgn_00077920 | LG18-21 |  | dermatan sulfate epimerase-like |
| DLA_LG1B_005580 | LG1B | DLAgn_00073820 | LG18-21 |  | uncharacterized protein loc101163881 |
| DLA_LG1B_005590 | LG1B | DLAgn_00073940 | LG18-21 |  | transposase |
| DLA_LG1B_005600 | LG1B | DLAgn_00074030 | LG18-21 |  | frizzled-8-like |
| DLA_LG1B_005610 | LG1B | DLAgn_00074050 | LG18-21 | * | NA |
| DLA_LG1B_005620 | LG1B | DLAgn_00074400 | LG18-21 |  | protein arginine n-methyltransferase 6 |
| DLA_LG1B_005640 | LG1B | DLAgn_00074520 | LG18-21 |  | c-c chemokine receptor type 4-like |
| DLA_LG1B_005650 | LG1B | DLAgn_00074640 | LG18-21 |  | NA |
| DLA_LG1B_005660 | LG1B | DLAgn_00074780 | LG18-21 |  | NA |
| DLA_LG1B_005670 | LG1B | DLAgn_00074860 | LG18-21 |  | unnamed protein product [Tetraodon nigroviridis] |
| DLA_LG1B_005680 | LG1B | DLAgn_00075050 | LG18-21 |  | apc membrane recruitment protein 2-like |
| DLA_LG1B_005690 | LG1B | DLAgn_00075220 | LG18-21 |  | butyrophilin subfamily 1 member a1-like |
| DLA_LG1B_005700 | LG1B | DLAgn_00075330 | LG18-21 |  | protein dln-1-like |
| DLA_LG1B_005710 | LG1B | DLAgn_00075550 | LG18-21 |  | arylamine n- pineal gland isozyme nat-10-like |
| DLA_LG1B_005725 | LG1B | DLAgn_00076030 | LG18-21 |  | ribosome biogenesis regulatory protein homolog |
| DLA_LG1B_005740 | LG1B | DLAgn_00076040 | LG18-21 |  | corticotropin-releasing hormone precursor |
| DLA_LG1B_005750 | LG1B | DLAgn_00076100 | LG18-21 |  | class e basic helix-loop-helix protein 22 |
| DLA_LG1B_005770 | LG1B | DLAgn_00076270 | LG18-21 |  | g-protein coupled receptor 12-like |
| DLA_LG1B_005780 | LG1B | DLAgn_00076480 | LG18-21 |  | nanos homolog 2 |
| DLA_LG1B_005800 | LG1B | DLAgn_00076700 | LG18-21 |  | xin actin-binding repeat-containing protein 1-like |
| DLA_LG1B_005810 | LG1B | DLAgn_00076710 | LG18-21 |  | xin actin-binding repeat-containing protein 1-like |
| DLA_LG1B_005820 | LG1B | DLAgn_00076750 | LG18-21 |  | transmembrane protein 22 |
| DLA_LG1B_005830 | LG1B | DLAgn_00076870 | LG18-21 |  | carbohydrate sulfotransferase 2 |
| DLA_LG1B_005845 | LG1B | DLAgn_00076920 | LG18-21 |  | c-c chemokine receptor type 11-like |
| DLA_LG1B_005850 | LG1B | DLAgn_00076950 | LG18-21 |  | uracil nucleotide cysteinyl leukotriene receptor-like |
| DLA_LG1B_005860 | LG1B | DLAgn_00076970 | LG18-21 |  | otu domain-containing protein 1 |
| DLA_LG1B_005870 | LG1B | DLAgn_00077070 | LG18-21 |  | NA |
| DLA_LG1B_005880 | LG1B | DLAgn_00077290 | LG18-21 |  | NA |
| DLA_LG1B_005890 | LG1B | DLAgn_00077310 | LG18-21 | * | nuclear factor ovary-like |
| DLA_LG1B_005900 | LG1B | DLAgn_00077320 | LG18-21 |  | nuclear factor ovary-like |
| DLA_LG1B_005910 | LG1B | DLAgn_00077330 | LG18-21 |  | NA |
| DLA_LG1B_005915 | LG1B | DLAgn_00078390 | LG18-21 |  | kelch-like protein 34 |
| DLA_LG1B_005920 | LG1B | DLAgn_00078500 | LG18-21 |  | NA |
| DLA_LG1B_005930 | LG1B | DLAgn_00078550 | LG18-21 |  | NA |
| DLA_LG1B_005950 | LG1B | DLAgn_00078610 | LG18-21 |  | NA |
| DLA_LG1B_005955 | LG1B | DLAgn_00078670 | LG18-21 |  | melanocortin 4 receptor |
| DLA_LG1B_005960 | LG1B | DLAgn_00078730 | LG18-21 |  | NA |
| DLA_LG1B_005980 | LG1B | DLAgn_00078960 | LG18-21 |  | gtpase imap family member 8-like |
| DLA_LG1B_005990 | LG1B | DLAgn_00078980 | LG18-21 |  | NA |
| DLA_LG1B_006000 | LG1B | DLAgn_00078990 | LG18-21 |  | uncharacterized protein loc101486971 |
| DLA_LG1B_006010 | LG1B | DLAgn_00079020 | LG18-21 |  | epithelial-stromal interaction protein 1-like |
| DLA_LG1B_006020 | LG1B | DLAgn_00125170 | LG22-25 |  | general transcription factor ii-i repeat domain-containing protein 2-like |
| DLA_LG1B_006030 | LG1B | DLAgn_00125290 | LG22-25 |  | NA |
| DLA_LG1B_006040 | LG1B | DLAgn_00125380 | LG22-25 |  | g-protein coupled receptor 182 |
| DLA_LG1B_006050 | LG1B | DLAgn_00125530 | LG22-25 |  | NA |
| DLA_LG1B_006055 | LG1B | DLAgn_00125600 | LG22-25 |  | transposable element tc1 transposase |
| DLA_LG1B_006060 | LG1B | DLAgn_00125700 | LG22-25 |  | NA |
| DLA_LG1B_006070 | LG1B | DLAgn_00125940 | LG22-25 |  | p2y purinoceptor 1-like |
| DLA_LG1B_006075 | LG1B | DLAgn_00125980 | LG22-25 |  | p2y purinoceptor 1-like |
| DLA_LG1B_006075_2 | LG1B | DLAgn_00126000 | LG22-25 |  | udp-glucuronosyltransferase 2b31-like |
| DLA_LG1B_006075_3 | LG1B | DLAgn_00126290 | LG22-25 | * | NA |
| DLA_LG1B_006075_4 | LG1B | DLAgn_00126300 | LG22-25 | * | NA |
| DLA_LG1B_006080 | LG1B | DLAgn_00126430 | LG22-25 |  | ubx domain-containing protein 10-like |
| DLA_LG1B_006100 | LG1B | DLAgn_00126470 | LG22-25 |  | protein fam43b-like |
| DLA_LG1B_006110 | LG1B | DLAgn_00126480 | LG22-25 |  | NA |
| DLA_LG1B_006120 | LG1B | DLAgn_00126500 | LG22-25 |  | NA |
| DLA_LG1B_006130 | LG1B | DLAgn_00126670 | LG22-25 |  | NA |
| DLA_LG1B_006140 | LG1B | DLAgn_00126890 | LG22-25 |  | NA |
| DLA_LG1B_006150 | LG1B | DLAgn_00127120 | LG22-25 |  | NA |
| DLA_LG1B_006160 | LG1B | DLAgn_00127140 | LG22-25 |  | ccaat enhancer-binding protein beta |
| DLA_LG1B_006170 | LG1B | DLAgn_00127590 | LG22-25 |  | probable g-protein coupled receptor 173-like |
| DLA_LG1B_006180 | LG1B | DLAgn_00128060 | LG22-25 |  | NA |
| DLA_LG1B_006200 | LG1B | DLAgn_00128170 | LG22-25 |  | NA |
| DLA_LG1B_006210 | LG1B | DLAgn_00128390 | LG22-25 |  | NA |
| DLA_LG1B_006220 | LG1B | DLAgn_00128450 | LG22-25 |  | NA |
| DLA_LG1B_006230 | LG1B | DLAgn_00128510 | LG22-25 |  | NA |
| DLA_LG1B_006240 | LG1B | DLAgn_00128660 | LG22-25 |  | NA |
| DLA_LG1B_006260 | LG1B | DLAgn_00128770 | LG22-25 |  | platelet glycoprotein ix-like |
| DLA_LG1B_006270 | LG1B | DLAgn_00128790 | LG22-25 |  | iq motif and sec7 domain-containing protein 1-like isoform x2 |
| DLA_LG1B_006280 | LG1B | DLAgn_00129060 | LG22-25 | * | transposable element tc1 transposase |
| DLA_LG1B_006290 | LG1B | DLAgn_00129180 | LG22-25 |  | rod opsin |
| DLA_LG1B_006310 | LG1B | DLAgn_00129270 | LG22-25 |  | ammonium transporter rh type b-like |
| DLA_LG1B_006320 | LG1B | DLAgn_00129500 | LG22-25 |  | amphoterin-induced protein 3 |
| DLA_LG1B_006330 | LG1B | DLAgn_00129560 | LG22-25 |  | unnamed protein product [Tetraodon nigroviridis] |
| DLA_LG1B_006340 | LG1B | DLAgn_00129570 | LG22-25 |  | class e basic helix-loop-helix protein 23-like |
| DLA_LG1B_006350 | LG1B | DLAgn_00129750 | LG22-25 |  | transcription factor -like |
| DLA_LG1B_006360 | LG1B | DLAgn_00130240 | LG22-25 |  | potassium voltage-gated channel subfamily a member 2-like |
| DLA_LG1B_006390 | LG1B | DLAgn_00130410 | LG22-25 |  | l _3 |
| DLA_LG1B_006400 | LG1B | DLAgn_00130620 | LG22-25 |  | NA |
| DLA_LG1B_006410 | LG1B | DLAgn_00130790 | LG22-25 |  | galanin receptor type 1-like |
| DLA_LG1B_006420 | LG1B | DLAgn_00131030 | LG22-25 |  | c-c chemokine receptor type 4-like |
| DLA_LG1B_006430 | LG1B | DLAgn_00131040 | LG22-25 |  | visual pigment-like receptor peropsin-like |
| DLA_LG1B_006440 | LG1B | DLAgn_00131060 | LG22-25 |  | neurogenic differentiation factor 4-like |
| DLA_LG1B_006450 | LG1B | DLAgn_00131340 | LG22-25 |  | fidgetin-like protein 2 |
| DLA_LG1B_006480 | LG1B | DLAgn_00131350 | LG22-25 |  | NA |
| DLA_LG1B_006490 | LG1B | DLAgn_00131560 | LG22-25 |  | NA |
| DLA_LG1B_006510 | LG1B | DLAgn_00131680 | LG22-25 |  | rna-binding protein 15-like |
| DLA_LG1B_006520 | LG1B | DLAgn_00131790 | LG22-25 |  | amphoterin-induced protein 1-like |
| DLA_LG1B_006535 | LG1B | DLAgn_00131800 | LG22-25 |  | probable g-protein coupled receptor 61-like |
| DLA_LG1B_006540 | LG1B | DLAgn_00131940 | LG22-25 |  | NA |
| DLA_LG1B_006550 | LG1B | DLAgn_00132020 | LG22-25 |  | NA |
| DLA_LG1B_006560 | LG1B | DLAgn_00132040 | LG22-25 |  | NA |
| DLA_LG1B_006570 | LG1B | DLAgn_00132100 | LG22-25 |  | forkhead box l2 |
| DLA_LG1B_006580 | LG1B | DLAgn_00132200 | LG22-25 |  | kinesin-like protein kif1c isoform 2 |
| DLA_LG1B_006590 | LG1B | DLAgn_00133020 | LG22-25 |  | neurexophilin-4-like |
| DLA_LG1B_006600 | LG1B | DLAgn_00134000 | LG22-25 |  | NA |
| DLA_LG1B_006610 | LG1B | DLAgn_00134220 | LG22-25 |  | agrin |
| DLA_LG1B_006620 | LG1B | DLAgn_00134500 | LG22-25 |  | twist-related protein 2-like |
| DLA_LG1B_006640 | LG1B | DLAgn_00134620 | LG22-25 |  | NA |
| DLA_LG1B_006660 | LG1B | DLAgn_00135230 | LG22-25 |  | NA |
| DLA_LG1B_006670 | LG1B | DLAgn_00135260 | LG22-25 |  | reverse transcriptase-like protein |
| DLA_LG1B_006675 | LG1B | DLAgn_00135410 | LG22-25 |  | 39s ribosomal protein mitochondrial-like |
| DLA_LG1B_006690 | LG1B | DLAgn_00135430 | LG22-25 |  | protein las1 homolog |
| DLA_LG1B_006700 | LG1B | DLAgn_00135670 | LG22-25 |  | gag-pol fusion polyprotein |
| DLA_LG1B_006710 | LG1B | DLAgn_00135710 | LG22-25 |  | line-1 type transposase domain-containing protein 1-like |
| DLA_LG1B_006720 | LG1B | DLAgn_00135780 | LG22-25 |  | NA |
| DLA_LG1B_006740 | LG1B | DLAgn_00135850 | LG22-25 |  | probable g-protein coupled receptor 173 |
| DLA_LG1B_006750 | LG1B | DLAgn_00136060 | LG22-25 |  | zinc finger bed domain-containing protein 4 |
| DLA_LG1B_006760 | LG1B | DLAgn_00136070 | LG22-25 |  | zinc finger bed domain-containing protein 1-like |
| DLA_LG1B_006770 | LG1B |  |  |  |  |
| DLA_LG1B_006790 | LG1B |  |  |  |  |
| DLA_LG1B_006800 | LG1B |  |  |  |  |
| DLA_LG1B_006810 | LG1B |  |  |  |  |
| DLA_LG1B_006820 | LG1B |  |  |  |  |
| DLA_LG1B_006830 | LG1B |  |  |  |  |
| DLA_LG1B_006840 | LG1B |  |  |  |  |
| DLA_LG1B_006850 | LG1B |  |  |  |  |
| DLA_LG1B_006870 | LG1B |  |  |  |  |
| DLA_LG1B_006880 | LG1B |  |  |  |  |
| DLA_LG1B_006890 | LG1B |  |  |  |  |
| DLA_LG1B_006900 | LG1B |  |  |  |  |
| DLA_LG1B_006910 | LG1B |  |  |  |  |
| DLA_LG1B_006920 | LG1B |  |  |  |  |
| DLA_LG1B_006945 | LG1B |  |  |  |  |
| DLA_LG1B_006955 | LG1B |  |  |  |  |
| DLA_LG1B_006970 | LG1B |  |  |  |  |
| DLA_LG1B_006980 | LG1B |  |  |  |  |
| DLA_LG1B_006990 | LG1B |  |  |  |  |
| DLA_LG1B_007000 | LG1B |  |  |  |  |
| DLA_LG1B_007010 | LG1B |  |  |  |  |
| DLA_LG1B_007020 | LG1B |  |  |  |  |
| DLA_LG1B_007030 | LG1B |  |  |  |  |
| DLA_LG1B_007040 | LG1B |  |  |  |  |
| DLA_LG1B_007060 | LG1B |  |  |  |  |
| DLA_LG1B_007070 | LG1B |  |  |  |  |
| DLA_LG1B_007080 | LG1B |  |  |  |  |
| DLA_LG1B_007085 | LG1B |  |  |  |  |
| DLA_LG1B_007110 | LG1B |  |  |  |  |
| DLA_LG1B_007120 | LG1B |  |  |  |  |
| DLA_LG1B_007130 | LG1B |  |  |  |  |
| DLA_LG1B_007140 | LG1B |  |  |  |  |
| DLA_LG1B_007145 | LG1B |  |  |  |  |
| DLA_LG1B_007150 | LG1B |  |  |  |  |
| DLA_LG1B_007165 | LG1B |  |  |  |  |
| DLA_LG1B_007170 | LG1B |  |  |  |  |
| DLA_LG1B_007180 | LG1B |  |  |  |  |
| DLA_LG1B_007190 | LG1B |  |  |  |  |
| DLA_LG1B_007195 | LG1B |  |  |  |  |
| DLA_LG1B_007195_2 | LG1B |  |  |  |  |
| DLA_LG1B_007200 | LG1B |  |  |  |  |
| DLA_LG1B_007210 | LG1B |  |  |  |  |
| DLA_LG1B_007220 | LG1B |  |  |  |  |
| DLA_LG1B_007240 | LG1B |  |  |  |  |
| DLA_LG1B_007260 | LG1B |  |  |  |  |
| DLA_LG1B_007270 | LG1B |  |  |  |  |
| DLA_LG1B_007280 | LG1B |  |  |  |  |
| DLA_LG1B_007285 | LG1B |  |  |  |  |
| DLA_LG1B_007290 | LG1B |  |  |  |  |
| DLA_LG1B_007295 | LG1B |  |  |  |  |
| DLA_LG1B_007300 | LG1B |  |  |  |  |
| DLA_LG1B_007310 | LG1B |  |  |  |  |
| DLA_LG1B_007330 | LG1B |  |  |  |  |
| DLA_LG1B_007335 | LG1B |  |  |  |  |
| DLA_LG1B_007375 | LG1B |  |  |  |  |
| DLA_LG1B_007380 | LG1B |  |  |  |  |
| DLA_LG1B_007390 | LG1B |  |  |  |  |
| DLA_LG1B_007400 | LG1B |  |  |  |  |
| DLA_LG1B_007420 | LG1B |  |  |  |  |
| DLA_LG1B_007430 | LG1B |  |  |  |  |
| DLA_LG2_002660 | LG2 |  |  |  |  |
| DLA_LG2_000460 | LG2 |  |  |  |  |
| DLA_LG2_000700 | LG2 |  |  |  |  |
| DLA_LG2_000910 | LG2 |  |  |  |  |
| DLA_LG2_001960 | LG2 |  |  |  |  |
| DLA_LG2_003620 | LG2 |  |  |  |  |
| DLA_LG2_004060 | LG2 |  |  |  |  |
| DLA_LG2_004400 | LG2 |  |  |  |  |
| DLA_LG2_005120 | LG2 |  |  |  |  |
| DLA_LG2_005650 | LG2 |  |  |  |  |
| DLA_LG2_005860 | LG2 |  |  |  |  |
| DLA_LG2_006080 | LG2 |  |  |  |  |
| DLA_LG2_006810 | LG2 |  |  |  |  |
| DLA_LG2_000020 | LG2 |  |  |  |  |
| DLA_LG2_000090 | LG2 |  |  |  |  |
| DLA_LG2_000150 | LG2 |  |  |  |  |
| DLA_LG2_000330 | LG2 |  |  |  |  |
| DLA_LG2_000560 | LG2 |  |  |  |  |
| DLA_LG2_000590 | LG2 |  |  |  |  |
| DLA_LG2_000610 | LG2 |  |  |  |  |
| DLA_LG2_000710 | LG2 |  |  |  |  |
| DLA_LG2_000720 | LG2 |  |  |  |  |
| DLA_LG2_000730 | LG2 |  |  |  |  |
| DLA_LG2_000760 | LG2 |  |  |  |  |
| DLA_LG2_000940 | LG2 |  |  |  |  |
| DLA_LG2_001370 | LG2 |  |  |  |  |
| DLA_LG2_001420 | LG2 |  |  |  |  |
| DLA_LG2_001610 | LG2 |  |  |  |  |
| DLA_LG2_001860 | LG2 |  |  |  |  |
| DLA_LG2_002090 | LG2 |  |  |  |  |
| DLA_LG2_002100 | LG2 |  |  |  |  |
| DLA_LG2_002340 | LG2 |  |  |  |  |
| DLA_LG2_002525 | LG2 |  |  |  |  |
| DLA_LG2_002620 | LG2 |  |  |  |  |
| DLA_LG2_002690 | LG2 |  |  |  |  |
| DLA_LG2_002890 | LG2 |  |  |  |  |
| DLA_LG2_002960 | LG2 |  |  |  |  |
| DLA_LG2_003060 | LG2 |  |  |  |  |
| DLA_LG2_003180 | LG2 |  |  |  |  |
| DLA_LG2_003200 | LG2 |  |  |  |  |
| DLA_LG2_003530 | LG2 |  |  |  |  |
| DLA_LG2_003770 | LG2 |  |  |  |  |
| DLA_LG2_003930 | LG2 |  |  |  |  |
| DLA_LG2_003950 | LG2 |  |  |  |  |
| DLA_LG2_004180 | LG2 |  |  |  |  |
| DLA_LG2_004250 | LG2 |  |  |  |  |
| DLA_LG2_004610 | LG2 |  |  |  |  |
| DLA_LG2_004675 | LG2 |  |  |  |  |
| DLA_LG2_004680 | LG2 |  |  |  |  |
| DLA_LG2_004760 | LG2 |  |  |  |  |
| DLA_LG2_004820 | LG2 |  |  |  |  |
| DLA_LG2_004860 | LG2 |  |  |  |  |
| DLA_LG2_004900 | LG2 |  |  |  |  |
| DLA_LG2_004960 | LG2 |  |  |  |  |
| DLA_LG2_005350 | LG2 |  |  |  |  |
| DLA_LG2_005700 | LG2 |  |  |  |  |
| DLA_LG2_005730 | LG2 |  |  |  |  |
| DLA_LG2_005800 | LG2 |  |  |  |  |
| DLA_LG2_005910 | LG2 |  |  |  |  |
| DLA_LG2_005990 | LG2 |  |  |  |  |
| DLA_LG2_005995 | LG2 |  |  |  |  |
| DLA_LG2_006140 | LG2 |  |  |  |  |
| DLA_LG2_006145 | LG2 |  |  |  |  |
| DLA_LG2_006250 | LG2 |  |  |  |  |
| DLA_LG2_006510 | LG2 |  |  |  |  |
| DLA_LG2_006520 | LG2 |  |  |  |  |
| DLA_LG2_006540 | LG2 |  |  |  |  |
| DLA_LG2_006665 | LG2 |  |  |  |  |
| DLA_LG2_006730 | LG2 |  |  |  |  |
| DLA_LG2_006750 | LG2 |  |  |  |  |
| DLA_LG2_006770 | LG2 |  |  |  |  |
| DLA_LG2_006780 | LG2 |  |  |  |  |
| DLA_LG2_006850 | LG2 |  |  |  |  |
| DLA_LG2_006890 | LG2 |  |  |  |  |
| DLA_LG2_007110 | LG2 |  |  |  |  |
| DLA_LG2_000010 | LG2 |  |  |  |  |
| DLA_LG2_000030 | LG2 |  |  |  |  |
| DLA_LG2_000040 | LG2 |  |  |  |  |
| DLA_LG2_000050 | LG2 |  |  |  |  |
| DLA_LG2_000070 | LG2 |  |  |  |  |
| DLA_LG2_000075 | LG2 |  |  |  |  |
| DLA_LG2_000075_2 | LG2 |  |  |  |  |
| DLA_LG2_000080 | LG2 |  |  |  |  |
| DLA_LG2_000100 | LG2 |  |  |  |  |
| DLA_LG2_000110 | LG2 |  |  |  |  |
| DLA_LG2_000120 | LG2 |  |  |  |  |
| DLA_LG2_000130 | LG2 |  |  |  |  |
| DLA_LG2_000140 | LG2 |  |  |  |  |
| DLA_LG2_000160 | LG2 |  |  |  |  |
| DLA_LG2_000180 | LG2 |  |  |  |  |
| DLA_LG2_000200 | LG2 |  |  |  |  |
| DLA_LG2_000210 | LG2 |  |  |  |  |
| DLA_LG2_000215 | LG2 |  |  |  |  |
| DLA_LG2_000220 | LG2 |  |  |  |  |
| DLA_LG2_000230 | LG2 |  |  |  |  |
| DLA_LG2_000240 | LG2 |  |  |  |  |
| DLA_LG2_000250 | LG2 |  |  |  |  |
| DLA_LG2_000260 | LG2 |  |  |  |  |
| DLA_LG2_000270 | LG2 |  |  |  |  |
| DLA_LG2_000280 | LG2 |  |  |  |  |
| DLA_LG2_000285 | LG2 |  |  |  |  |
| DLA_LG2_000290 | LG2 |  |  |  |  |
| DLA_LG2_000300 | LG2 |  |  |  |  |
| DLA_LG2_000310 | LG2 |  |  |  |  |
| DLA_LG2_000320 | LG2 |  |  |  |  |
| DLA_LG2_000335 | LG2 |  |  |  |  |
| DLA_LG2_000340 | LG2 |  |  |  |  |
| DLA_LG2_000360 | LG2 |  |  |  |  |
| DLA_LG2_000370 | LG2 |  |  |  |  |
| DLA_LG2_000380 | LG2 |  |  |  |  |
| DLA_LG2_000390 | LG2 |  |  |  |  |
| DLA_LG2_000400 | LG2 |  |  |  |  |
| DLA_LG2_000410 | LG2 |  |  |  |  |
| DLA_LG2_000420 | LG2 |  |  |  |  |
| DLA_LG2_000430 | LG2 |  |  |  |  |
| DLA_LG2_000440 | LG2 |  |  |  |  |
| DLA_LG2_000450 | LG2 |  |  |  |  |
| DLA_LG2_000480 | LG2 |  |  |  |  |
| DLA_LG2_000490 | LG2 |  |  |  |  |
| DLA_LG2_000500 | LG2 |  |  |  |  |
| DLA_LG2_000510 | LG2 |  |  |  |  |
| DLA_LG2_000520 | LG2 |  |  |  |  |
| DLA_LG2_000530 | LG2 |  |  |  |  |
| DLA_LG2_000540 | LG2 |  |  |  |  |
| DLA_LG2_000550 | LG2 |  |  |  |  |
| DLA_LG2_000570 | LG2 |  |  |  |  |
| DLA_LG2_000580 | LG2 |  |  |  |  |
| DLA_LG2_000600 | LG2 |  |  |  |  |
| DLA_LG2_000620 | LG2 |  |  |  |  |
| DLA_LG2_000630 | LG2 |  |  |  |  |
| DLA_LG2_000640 | LG2 |  |  |  |  |
| DLA_LG2_000650 | LG2 |  |  |  |  |
| DLA_LG2_000670 | LG2 |  |  |  |  |
| DLA_LG2_000680 | LG2 |  |  |  |  |
| DLA_LG2_000690 | LG2 |  |  |  |  |
| DLA_LG2_000740 | LG2 |  |  |  |  |
| DLA_LG2_000750 | LG2 |  |  |  |  |
| DLA_LG2_000755 | LG2 |  |  |  |  |
| DLA_LG2_000770 | LG2 |  |  |  |  |
| DLA_LG2_000780 | LG2 |  |  |  |  |
| DLA_LG2_000790 | LG2 |  |  |  |  |
| DLA_LG2_000800 | LG2 |  |  |  |  |
| DLA_LG2_000810 | LG2 |  |  |  |  |
| DLA_LG2_000830 | LG2 |  |  |  |  |
| DLA_LG2_000840 | LG2 |  |  |  |  |
| DLA_LG2_000850 | LG2 |  |  |  |  |
| DLA_LG2_000860 | LG2 |  |  |  |  |
| DLA_LG2_000870 | LG2 |  |  |  |  |
| DLA_LG2_000890 | LG2 |  |  |  |  |
| DLA_LG2_000900 | LG2 |  |  |  |  |
| DLA_LG2_000915 | LG2 |  |  |  |  |
| DLA_LG2_000920 | LG2 |  |  |  |  |
| DLA_LG2_000930 | LG2 |  |  |  |  |
| DLA_LG2_000950 | LG2 |  |  |  |  |
| DLA_LG2_000960 | LG2 |  |  |  |  |
| DLA_LG2_000970 | LG2 |  |  |  |  |
| DLA_LG2_000980 | LG2 |  |  |  |  |
| DLA_LG2_000990 | LG2 |  |  |  |  |
| DLA_LG2_001000 | LG2 |  |  |  |  |
| DLA_LG2_001020 | LG2 |  |  |  |  |
| DLA_LG2_001030 | LG2 |  |  |  |  |
| DLA_LG2_001040 | LG2 |  |  |  |  |
| DLA_LG2_001050 | LG2 |  |  |  |  |
| DLA_LG2_001060 | LG2 |  |  |  |  |
| DLA_LG2_001070 | LG2 |  |  |  |  |
| DLA_LG2_001080 | LG2 |  |  |  |  |
| DLA_LG2_001085 | LG2 |  |  |  |  |
| DLA_LG2_001090 | LG2 |  |  |  |  |
| DLA_LG2_001100 | LG2 |  |  |  |  |
| DLA_LG2_001110 | LG2 |  |  |  |  |
| DLA_LG2_001120 | LG2 |  |  |  |  |
| DLA_LG2_001130 | LG2 |  |  |  |  |
| DLA_LG2_001140 | LG2 |  |  |  |  |
| DLA_LG2_001150 | LG2 |  |  |  |  |
| DLA_LG2_001160 | LG2 |  |  |  |  |
| DLA_LG2_001170 | LG2 |  |  |  |  |
| DLA_LG2_001180 | LG2 |  |  |  |  |
| DLA_LG2_001190 | LG2 |  |  |  |  |
| DLA_LG2_001200 | LG2 |  |  |  |  |
| DLA_LG2_001210 | LG2 |  |  |  |  |
| DLA_LG2_001230 | LG2 |  |  |  |  |
| DLA_LG2_001240 | LG2 |  |  |  |  |
| DLA_LG2_001250 | LG2 |  |  |  |  |
| DLA_LG2_001260 | LG2 |  |  |  |  |
| DLA_LG2_001270 | LG2 |  |  |  |  |
| DLA_LG2_001280 | LG2 |  |  |  |  |
| DLA_LG2_001290 | LG2 |  |  |  |  |
| DLA_LG2_001320 | LG2 |  |  |  |  |
| DLA_LG2_001330 | LG2 |  |  |  |  |
| DLA_LG2_001340 | LG2 |  |  |  |  |
| DLA_LG2_001350 | LG2 |  |  |  |  |
| DLA_LG2_001360 | LG2 |  |  |  |  |
| DLA_LG2_001365 | LG2 |  |  |  |  |
| DLA_LG2_001380 | LG2 |  |  |  |  |
| DLA_LG2_001390 | LG2 |  |  |  |  |
| DLA_LG2_001400 | LG2 |  |  |  |  |
| DLA_LG2_001410 | LG2 |  |  |  |  |
| DLA_LG2_001430 | LG2 |  |  |  |  |
| DLA_LG2_001440 | LG2 |  |  |  |  |
| DLA_LG2_001450 | LG2 |  |  |  |  |
| DLA_LG2_001460 | LG2 |  |  |  |  |
| DLA_LG2_001470 | LG2 |  |  |  |  |
| DLA_LG2_001480 | LG2 |  |  |  |  |
| DLA_LG2_001485 | LG2 |  |  |  |  |
| DLA_LG2_001490 | LG2 |  |  |  |  |
| DLA_LG2_001500 | LG2 |  |  |  |  |
| DLA_LG2_001510 | LG2 |  |  |  |  |
| DLA_LG2_001530 | LG2 |  |  |  |  |
| DLA_LG2_001540 | LG2 |  |  |  |  |
| DLA_LG2_001550 | LG2 |  |  |  |  |
| DLA_LG2_001560 | LG2 |  |  |  |  |
| DLA_LG2_001580 | LG2 |  |  |  |  |
| DLA_LG2_001590 | LG2 |  |  |  |  |
| DLA_LG2_001600 | LG2 |  |  |  |  |
| DLA_LG2_001615 | LG2 |  |  |  |  |
| DLA_LG2_001620 | LG2 |  |  |  |  |
| DLA_LG2_001630 | LG2 |  |  |  |  |
| DLA_LG2_001640 | LG2 |  |  |  |  |
| DLA_LG2_001650 | LG2 |  |  |  |  |
| DLA_LG2_001660 | LG2 |  |  |  |  |
| DLA_LG2_001670 | LG2 |  |  |  |  |
| DLA_LG2_001680 | LG2 |  |  |  |  |
| DLA_LG2_001690 | LG2 |  |  |  |  |
| DLA_LG2_001700 | LG2 |  |  |  |  |
| DLA_LG2_001705 | LG2 |  |  |  |  |
| DLA_LG2_001710 | LG2 |  |  |  |  |
| DLA_LG2_001720 | LG2 |  |  |  |  |
| DLA_LG2_001730 | LG2 |  |  |  |  |
| DLA_LG2_001740 | LG2 |  |  |  |  |
| DLA_LG2_001750 | LG2 |  |  |  |  |
| DLA_LG2_001760 | LG2 |  |  |  |  |
| DLA_LG2_001770 | LG2 |  |  |  |  |
| DLA_LG2_001780 | LG2 |  |  |  |  |
| DLA_LG2_001790 | LG2 |  |  |  |  |
| DLA_LG2_001800 | LG2 |  |  |  |  |
| DLA_LG2_001810 | LG2 |  |  |  |  |
| DLA_LG2_001820 | LG2 |  |  |  |  |
| DLA_LG2_001840 | LG2 |  |  |  |  |
| DLA_LG2_001850 | LG2 |  |  |  |  |
| DLA_LG2_001870 | LG2 |  |  |  |  |
| DLA_LG2_001880 | LG2 |  |  |  |  |
| DLA_LG2_001890 | LG2 |  |  |  |  |
| DLA_LG2_001900 | LG2 |  |  |  |  |
| DLA_LG2_001910 | LG2 |  |  |  |  |
| DLA_LG2_001920 | LG2 |  |  |  |  |
| DLA_LG2_001930 | LG2 |  |  |  |  |
| DLA_LG2_001940 | LG2 |  |  |  |  |
| DLA_LG2_001950 | LG2 |  |  |  |  |
| DLA_LG2_001965 | LG2 |  |  |  |  |
| DLA_LG2_001970 | LG2 |  |  |  |  |
| DLA_LG2_001980 | LG2 |  |  |  |  |
| DLA_LG2_001990 | LG2 |  |  |  |  |
| DLA_LG2_002000 | LG2 |  |  |  |  |
| DLA_LG2_002010 | LG2 |  |  |  |  |
| DLA_LG2_002020 | LG2 |  |  |  |  |
| DLA_LG2_002030 | LG2 |  |  |  |  |
| DLA_LG2_002050 | LG2 |  |  |  |  |
| DLA_LG2_002060 | LG2 |  |  |  |  |
| DLA_LG2_002070 | LG2 |  |  |  |  |
| DLA_LG2_002080 | LG2 |  |  |  |  |
| DLA_LG2_002105_2 | LG2 |  |  |  |  |
| DLA_LG2_002120 | LG2 |  |  |  |  |
| DLA_LG2_002130 | LG2 |  |  |  |  |
| DLA_LG2_002140 | LG2 |  |  |  |  |
| DLA_LG2_002150 | LG2 |  |  |  |  |
| DLA_LG2_002160 | LG2 |  |  |  |  |
| DLA_LG2_002170 | LG2 |  |  |  |  |
| DLA_LG2_002180 | LG2 |  |  |  |  |
| DLA_LG2_002190 | LG2 |  |  |  |  |
| DLA_LG2_002200 | LG2 |  |  |  |  |
| DLA_LG2_002210 | LG2 |  |  |  |  |
| DLA_LG2_002220 | LG2 |  |  |  |  |
| DLA_LG2_002230 | LG2 |  |  |  |  |
| DLA_LG2_002240 | LG2 |  |  |  |  |
| DLA_LG2_002250 | LG2 |  |  |  |  |
| DLA_LG2_002260 | LG2 |  |  |  |  |
| DLA_LG2_002270 | LG2 |  |  |  |  |
| DLA_LG2_002280 | LG2 |  |  |  |  |
| DLA_LG2_002290 | LG2 |  |  |  |  |
| DLA_LG2_002300 | LG2 |  |  |  |  |
| DLA_LG2_002310 | LG2 |  |  |  |  |
| DLA_LG2_002320 | LG2 |  |  |  |  |
| DLA_LG2_002330 | LG2 |  |  |  |  |
| DLA_LG2_002350 | LG2 |  |  |  |  |
| DLA_LG2_002360 | LG2 |  |  |  |  |
| DLA_LG2_002370 | LG2 |  |  |  |  |
| DLA_LG2_002380 | LG2 |  |  |  |  |
| DLA_LG2_002390 | LG2 |  |  |  |  |
| DLA_LG2_002400 | LG2 |  |  |  |  |
| DLA_LG2_002410 | LG2 |  |  |  |  |
| DLA_LG2_002420 | LG2 |  |  |  |  |
| DLA_LG2_002430 | LG2 |  |  |  |  |
| DLA_LG2_002435 | LG2 |  |  |  |  |
| DLA_LG2_002440 | LG2 |  |  |  |  |
| DLA_LG2_002450 | LG2 |  |  |  |  |
| DLA_LG2_002460 | LG2 |  |  |  |  |
| DLA_LG2_002470 | LG2 |  |  |  |  |
| DLA_LG2_002480 | LG2 |  |  |  |  |
| DLA_LG2_002490 | LG2 |  |  |  |  |
| DLA_LG2_002500 | LG2 |  |  |  |  |
| DLA_LG2_002505 | LG2 |  |  |  |  |
| DLA_LG2_002510 | LG2 |  |  |  |  |
| DLA_LG2_002520 | LG2 |  |  |  |  |
| DLA_LG2_002530 | LG2 |  |  |  |  |
| DLA_LG2_002540 | LG2 |  |  |  |  |
| DLA_LG2_002550 | LG2 |  |  |  |  |
| DLA_LG2_002560 | LG2 |  |  |  |  |
| DLA_LG2_002570 | LG2 |  |  |  |  |
| DLA_LG2_002580 | LG2 |  |  |  |  |
| DLA_LG2_002600 | LG2 |  |  |  |  |
| DLA_LG2_002605 | LG2 |  |  |  |  |
| DLA_LG2_002605_2 | LG2 |  |  |  |  |
| DLA_LG2_002610 | LG2 |  |  |  |  |
| DLA_LG2_002615 | LG2 |  |  |  |  |
| DLA_LG2_002630 | LG2 |  |  |  |  |
| DLA_LG2_002640 | LG2 |  |  |  |  |
| DLA_LG2_002670 | LG2 |  |  |  |  |
| DLA_LG2_002680 | LG2 |  |  |  |  |
| DLA_LG2_002700 | LG2 |  |  |  |  |
| DLA_LG2_002710 | LG2 |  |  |  |  |
| DLA_LG2_002720 | LG2 |  |  |  |  |
| DLA_LG2_002730 | LG2 |  |  |  |  |
| DLA_LG2_002735 | LG2 |  |  |  |  |
| DLA_LG2_002740 | LG2 |  |  |  |  |
| DLA_LG2_002750 | LG2 |  |  |  |  |
| DLA_LG2_002760 | LG2 |  |  |  |  |
| DLA_LG2_002780 | LG2 |  |  |  |  |
| DLA_LG2_002800 | LG2 |  |  |  |  |
| DLA_LG2_002805 | LG2 |  |  |  |  |
| DLA_LG2_002805_2 | LG2 |  |  |  |  |
| DLA_LG2_002820 | LG2 |  |  |  |  |
| DLA_LG2_002830 | LG2 |  |  |  |  |
| DLA_LG2_002840 | LG2 |  |  |  |  |
| DLA_LG2_002850 | LG2 |  |  |  |  |
| DLA_LG2_002860 | LG2 |  |  |  |  |
| DLA_LG2_002870 | LG2 |  |  |  |  |
| DLA_LG2_002880 | LG2 |  |  |  |  |
| DLA_LG2_002900 | LG2 |  |  |  |  |
| DLA_LG2_002910 | LG2 |  |  |  |  |
| DLA_LG2_002920 | LG2 |  |  |  |  |
| DLA_LG2_002925 | LG2 |  |  |  |  |
| DLA_LG2_002930 | LG2 |  |  |  |  |
| DLA_LG2_002940 | LG2 |  |  |  |  |
| DLA_LG2_002950 | LG2 |  |  |  |  |
| DLA_LG2_002970 | LG2 |  |  |  |  |
| DLA_LG2_002990 | LG2 |  |  |  |  |
| DLA_LG2_003000 | LG2 |  |  |  |  |
| DLA_LG2_003010 | LG2 |  |  |  |  |
| DLA_LG2_003020 | LG2 |  |  |  |  |
| DLA_LG2_003030 | LG2 |  |  |  |  |
| DLA_LG2_003040 | LG2 |  |  |  |  |
| DLA_LG2_003050 | LG2 |  |  |  |  |
| DLA_LG2_003055 | LG2 |  |  |  |  |
| DLA_LG2_003080 | LG2 |  |  |  |  |
| DLA_LG2_003090 | LG2 |  |  |  |  |
| DLA_LG2_003100 | LG2 |  |  |  |  |
| DLA_LG2_003110 | LG2 |  |  |  |  |
| DLA_LG2_003120 | LG2 |  |  |  |  |
| DLA_LG2_003130 | LG2 |  |  |  |  |
| DLA_LG2_003140 | LG2 |  |  |  |  |
| DLA_LG2_003150 | LG2 |  |  |  |  |
| DLA_LG2_003160 | LG2 |  |  |  |  |
| DLA_LG2_003190 | LG2 |  |  |  |  |
| DLA_LG2_003210 | LG2 |  |  |  |  |
| DLA_LG2_003220 | LG2 |  |  |  |  |
| DLA_LG2_003230 | LG2 |  |  |  |  |
| DLA_LG2_003240 | LG2 |  |  |  |  |
| DLA_LG2_003250 | LG2 |  |  |  |  |
| DLA_LG2_003260 | LG2 |  |  |  |  |
| DLA_LG2_003270 | LG2 |  |  |  |  |
| DLA_LG2_003280 | LG2 |  |  |  |  |
| DLA_LG2_003290 | LG2 |  |  |  |  |
| DLA_LG2_003300 | LG2 |  |  |  |  |
| DLA_LG2_003305 | LG2 |  |  |  |  |
| DLA_LG2_003310 | LG2 |  |  |  |  |
| DLA_LG2_003320 | LG2 |  |  |  |  |
| DLA_LG2_003330 | LG2 |  |  |  |  |
| DLA_LG2_003340 | LG2 |  |  |  |  |
| DLA_LG2_003350 | LG2 |  |  |  |  |
| DLA_LG2_003360 | LG2 |  |  |  |  |
| DLA_LG2_003370 | LG2 |  |  |  |  |
| DLA_LG2_003390 | LG2 |  |  |  |  |
| DLA_LG2_003400 | LG2 |  |  |  |  |
| DLA_LG2_003410 | LG2 |  |  |  |  |
| DLA_LG2_003420 | LG2 |  |  |  |  |
| DLA_LG2_003430 | LG2 |  |  |  |  |
| DLA_LG2_003440 | LG2 |  |  |  |  |
| DLA_LG2_003450 | LG2 |  |  |  |  |
| DLA_LG2_003460 | LG2 |  |  |  |  |
| DLA_LG2_003470 | LG2 |  |  |  |  |
| DLA_LG2_003480 | LG2 |  |  |  |  |
| DLA_LG2_003490 | LG2 |  |  |  |  |
| DLA_LG2_003500 | LG2 |  |  |  |  |
| DLA_LG2_003510 | LG2 |  |  |  |  |
| DLA_LG2_003520 | LG2 |  |  |  |  |
| DLA_LG2_003540 | LG2 |  |  |  |  |
| DLA_LG2_003550 | LG2 |  |  |  |  |
| DLA_LG2_003560 | LG2 |  |  |  |  |
| DLA_LG2_003570 | LG2 |  |  |  |  |
| DLA_LG2_003580 | LG2 |  |  |  |  |
| DLA_LG2_003590 | LG2 |  |  |  |  |
| DLA_LG2_003610 | LG2 |  |  |  |  |
| DLA_LG2_003630 | LG2 |  |  |  |  |
| DLA_LG2_003640 | LG2 |  |  |  |  |
| DLA_LG2_003650 | LG2 |  |  |  |  |
| DLA_LG2_003660 | LG2 |  |  |  |  |
| DLA_LG2_003665 | LG2 |  |  |  |  |
| DLA_LG2_003680 | LG2 |  |  |  |  |
| DLA_LG2_003690 | LG2 |  |  |  |  |
| DLA_LG2_003700 | LG2 |  |  |  |  |
| DLA_LG2_003710 | LG2 |  |  |  |  |
| DLA_LG2_003720 | LG2 |  |  |  |  |
| DLA_LG2_003730 | LG2 |  |  |  |  |
| DLA_LG2_003740 | LG2 |  |  |  |  |
| DLA_LG2_003745 | LG2 |  |  |  |  |
| DLA_LG2_003750 | LG2 |  |  |  |  |
| DLA_LG2_003760 | LG2 |  |  |  |  |
| DLA_LG2_003780 | LG2 |  |  |  |  |
| DLA_LG2_003790 | LG2 |  |  |  |  |
| DLA_LG2_003810 | LG2 |  |  |  |  |
| DLA_LG2_003820 | LG2 |  |  |  |  |
| DLA_LG2_003830 | LG2 |  |  |  |  |
| DLA_LG2_003840 | LG2 |  |  |  |  |
| DLA_LG2_003850 | LG2 |  |  |  |  |
| DLA_LG2_003870 | LG2 |  |  |  |  |
| DLA_LG2_003880 | LG2 |  |  |  |  |
| DLA_LG2_003890 | LG2 |  |  |  |  |
| DLA_LG2_003910 | LG2 |  |  |  |  |
| DLA_LG2_003925 | LG2 |  |  |  |  |
| DLA_LG2_003940 | LG2 |  |  |  |  |
| DLA_LG2_003960 | LG2 |  |  |  |  |
| DLA_LG2_003970 | LG2 |  |  |  |  |
| DLA_LG2_003980 | LG2 |  |  |  |  |
| DLA_LG2_004000 | LG2 |  |  |  |  |
| DLA_LG2_004010 | LG2 |  |  |  |  |
| DLA_LG2_004040 | LG2 |  |  |  |  |
| DLA_LG2_004050 | LG2 |  |  |  |  |
| DLA_LG2_004070 | LG2 |  |  |  |  |
| DLA_LG2_004090 | LG2 |  |  |  |  |
| DLA_LG2_004100 | LG2 |  |  |  |  |
| DLA_LG2_004110 | LG2 |  |  |  |  |
| DLA_LG2_004120 | LG2 |  |  |  |  |
| DLA_LG2_004135 | LG2 |  |  |  |  |
| DLA_LG2_004140 | LG2 |  |  |  |  |
| DLA_LG2_004150 | LG2 |  |  |  |  |
| DLA_LG2_004160 | LG2 |  |  |  |  |
| DLA_LG2_004170 | LG2 |  |  |  |  |
| DLA_LG2_004190 | LG2 |  |  |  |  |
| DLA_LG2_004200 | LG2 |  |  |  |  |
| DLA_LG2_004210 | LG2 |  |  |  |  |
| DLA_LG2_004220 | LG2 |  |  |  |  |
| DLA_LG2_004230 | LG2 |  |  |  |  |
| DLA_LG2_004240 | LG2 |  |  |  |  |
| DLA_LG2_004260 | LG2 |  |  |  |  |
| DLA_LG2_004270 | LG2 |  |  |  |  |
| DLA_LG2_004280 | LG2 |  |  |  |  |
| DLA_LG2_004290 | LG2 |  |  |  |  |
| DLA_LG2_004300 | LG2 |  |  |  |  |
| DLA_LG2_004320 | LG2 |  |  |  |  |
| DLA_LG2_004330 | LG2 |  |  |  |  |
| DLA_LG2_004340 | LG2 |  |  |  |  |
| DLA_LG2_004350 | LG2 |  |  |  |  |
| DLA_LG2_004370 | LG2 |  |  |  |  |
| DLA_LG2_004375 | LG2 |  |  |  |  |
| DLA_LG2_004380 | LG2 |  |  |  |  |
| DLA_LG2_004390 | LG2 |  |  |  |  |
| DLA_LG2_004410 | LG2 |  |  |  |  |
| DLA_LG2_004420 | LG2 |  |  |  |  |
| DLA_LG2_004440 | LG2 |  |  |  |  |
| DLA_LG2_004450 | LG2 |  |  |  |  |
| DLA_LG2_004460 | LG2 |  |  |  |  |
| DLA_LG2_004470 | LG2 |  |  |  |  |
| DLA_LG2_004480 | LG2 |  |  |  |  |
| DLA_LG2_004490 | LG2 |  |  |  |  |
| DLA_LG2_004500 | LG2 |  |  |  |  |
| DLA_LG2_004510 | LG2 |  |  |  |  |
| DLA_LG2_004530 | LG2 |  |  |  |  |
| DLA_LG2_004540 | LG2 |  |  |  |  |
| DLA_LG2_004550 | LG2 |  |  |  |  |
| DLA_LG2_004560 | LG2 |  |  |  |  |
| DLA_LG2_004570 | LG2 |  |  |  |  |
| DLA_LG2_004580 | LG2 |  |  |  |  |
| DLA_LG2_004620 | LG2 |  |  |  |  |
| DLA_LG2_004630 | LG2 |  |  |  |  |
| DLA_LG2_004640 | LG2 |  |  |  |  |
| DLA_LG2_004650 | LG2 |  |  |  |  |
| DLA_LG2_004660 | LG2 |  |  |  |  |
| DLA_LG2_004670 | LG2 |  |  |  |  |
| DLA_LG2_004690 | LG2 |  |  |  |  |
| DLA_LG2_004710 | LG2 |  |  |  |  |
| DLA_LG2_004720 | LG2 |  |  |  |  |
| DLA_LG2_004730 | LG2 |  |  |  |  |
| DLA_LG2_004735 | LG2 |  |  |  |  |
| DLA_LG2_004750 | LG2 |  |  |  |  |
| DLA_LG2_004770 | LG2 |  |  |  |  |
| DLA_LG2_004780 | LG2 |  |  |  |  |
| DLA_LG2_004790 | LG2 |  |  |  |  |
| DLA_LG2_004800 | LG2 |  |  |  |  |
| DLA_LG2_004810 | LG2 |  |  |  |  |
| DLA_LG2_004830 | LG2 |  |  |  |  |
| DLA_LG2_004840 | LG2 |  |  |  |  |
| DLA_LG2_004845 | LG2 |  |  |  |  |
| DLA_LG2_004850 | LG2 |  |  |  |  |
| DLA_LG2_004870 | LG2 |  |  |  |  |
| DLA_LG2_004880 | LG2 |  |  |  |  |
| DLA_LG2_004890 | LG2 |  |  |  |  |
| DLA_LG2_004895 | LG2 |  |  |  |  |
| DLA_LG2_004910 | LG2 |  |  |  |  |
| DLA_LG2_004930 | LG2 |  |  |  |  |
| DLA_LG2_004940 | LG2 |  |  |  |  |
| DLA_LG2_004950 | LG2 |  |  |  |  |
| DLA_LG2_004965 | LG2 |  |  |  |  |
| DLA_LG2_004970 | LG2 |  |  |  |  |
| DLA_LG2_004980 | LG2 |  |  |  |  |
| DLA_LG2_004985 | LG2 |  |  |  |  |
| DLA_LG2_004990 | LG2 |  |  |  |  |
| DLA_LG2_005000 | LG2 |  |  |  |  |
| DLA_LG2_005010 | LG2 |  |  |  |  |
| DLA_LG2_005015 | LG2 |  |  |  |  |
| DLA_LG2_005020 | LG2 |  |  |  |  |
| DLA_LG2_005040 | LG2 |  |  |  |  |
| DLA_LG2_005050 | LG2 |  |  |  |  |
| DLA_LG2_005060 | LG2 |  |  |  |  |
| DLA_LG2_005070 | LG2 |  |  |  |  |
| DLA_LG2_005075 | LG2 |  |  |  |  |
| DLA_LG2_005090 | LG2 |  |  |  |  |
| DLA_LG2_005100 | LG2 |  |  |  |  |
| DLA_LG2_005110 | LG2 |  |  |  |  |
| DLA_LG2_005130 | LG2 |  |  |  |  |
| DLA_LG2_005140 | LG2 |  |  |  |  |
| DLA_LG2_005150 | LG2 |  |  |  |  |
| DLA_LG2_005160 | LG2 |  |  |  |  |
| DLA_LG2_005170 | LG2 |  |  |  |  |
| DLA_LG2_005180 | LG2 |  |  |  |  |
| DLA_LG2_005190 | LG2 |  |  |  |  |
| DLA_LG2_005200 | LG2 |  |  |  |  |
| DLA_LG2_005210 | LG2 |  |  |  |  |
| DLA_LG2_005220 | LG2 |  |  |  |  |
| DLA_LG2_005230 | LG2 |  |  |  |  |
| DLA_LG2_005240 | LG2 |  |  |  |  |
| DLA_LG2_005245 | LG2 |  |  |  |  |
| DLA_LG2_005250 | LG2 |  |  |  |  |
| DLA_LG2_005260 | LG2 |  |  |  |  |
| DLA_LG2_005270 | LG2 |  |  |  |  |
| DLA_LG2_005280 | LG2 |  |  |  |  |
| DLA_LG2_005290 | LG2 |  |  |  |  |
| DLA_LG2_005300 | LG2 |  |  |  |  |
| DLA_LG2_005310 | LG2 |  |  |  |  |
| DLA_LG2_005320 | LG2 |  |  |  |  |
| DLA_LG2_005330 | LG2 |  |  |  |  |
| DLA_LG2_005340 | LG2 |  |  |  |  |
| DLA_LG2_005360 | LG2 |  |  |  |  |
| DLA_LG2_005370 | LG2 |  |  |  |  |
| DLA_LG2_005390 | LG2 |  |  |  |  |
| DLA_LG2_005400 | LG2 |  |  |  |  |
| DLA_LG2_005420 | LG2 |  |  |  |  |
| DLA_LG2_005440 | LG2 |  |  |  |  |
| DLA_LG2_005450 | LG2 |  |  |  |  |
| DLA_LG2_005460 | LG2 |  |  |  |  |
| DLA_LG2_005470 | LG2 |  |  |  |  |
| DLA_LG2_005480 | LG2 |  |  |  |  |
| DLA_LG2_005490 | LG2 |  |  |  |  |
| DLA_LG2_005500 | LG2 |  |  |  |  |
| DLA_LG2_005510 | LG2 |  |  |  |  |
| DLA_LG2_005520 | LG2 |  |  |  |  |
| DLA_LG2_005530 | LG2 |  |  |  |  |
| DLA_LG2_005540 | LG2 |  |  |  |  |
| DLA_LG2_005550 | LG2 |  |  |  |  |
| DLA_LG2_005560 | LG2 |  |  |  |  |
| DLA_LG2_005570 | LG2 |  |  |  |  |
| DLA_LG2_005580 | LG2 |  |  |  |  |
| DLA_LG2_005590 | LG2 |  |  |  |  |
| DLA_LG2_005600 | LG2 |  |  |  |  |
| DLA_LG2_005610 | LG2 |  |  |  |  |
| DLA_LG2_005620 | LG2 |  |  |  |  |
| DLA_LG2_005630 | LG2 |  |  |  |  |
| DLA_LG2_005640 | LG2 |  |  |  |  |
| DLA_LG2_005660 | LG2 |  |  |  |  |
| DLA_LG2_005670 | LG2 |  |  |  |  |
| DLA_LG2_005680 | LG2 |  |  |  |  |
| DLA_LG2_005690 | LG2 |  |  |  |  |
| DLA_LG2_005710 | LG2 |  |  |  |  |
| DLA_LG2_005720 | LG2 |  |  |  |  |
| DLA_LG2_005735 | LG2 |  |  |  |  |
| DLA_LG2_005740 | LG2 |  |  |  |  |
| DLA_LG2_005750 | LG2 |  |  |  |  |
| DLA_LG2_005760 | LG2 |  |  |  |  |
| DLA_LG2_005770 | LG2 |  |  |  |  |
| DLA_LG2_005780 | LG2 |  |  |  |  |
| DLA_LG2_005790 | LG2 |  |  |  |  |
| DLA_LG2_005810 | LG2 |  |  |  |  |
| DLA_LG2_005820 | LG2 |  |  |  |  |
| DLA_LG2_005830 | LG2 |  |  |  |  |
| DLA_LG2_005840 | LG2 |  |  |  |  |
| DLA_LG2_005855 | LG2 |  |  |  |  |
| DLA_LG2_005870 | LG2 |  |  |  |  |
| DLA_LG2_005880 | LG2 |  |  |  |  |
| DLA_LG2_005885 | LG2 |  |  |  |  |
| DLA_LG2_005890 | LG2 |  |  |  |  |
| DLA_LG2_005900 | LG2 |  |  |  |  |
| DLA_LG2_005920 | LG2 |  |  |  |  |
| DLA_LG2_005930 | LG2 |  |  |  |  |
| DLA_LG2_005940 | LG2 |  |  |  |  |
| DLA_LG2_005950 | LG2 |  |  |  |  |
| DLA_LG2_005960 | LG2 |  |  |  |  |
| DLA_LG2_005970 | LG2 |  |  |  |  |
| DLA_LG2_005980 | LG2 |  |  |  |  |
| DLA_LG2_006000 | LG2 |  |  |  |  |
| DLA_LG2_006005_2 | LG2 |  |  |  |  |
| DLA_LG2_006010 | LG2 |  |  |  |  |
| DLA_LG2_006020 | LG2 |  |  |  |  |
| DLA_LG2_006030 | LG2 |  |  |  |  |
| DLA_LG2_006040 | LG2 |  |  |  |  |
| DLA_LG2_006050 | LG2 |  |  |  |  |
| DLA_LG2_006060 | LG2 |  |  |  |  |
| DLA_LG2_006070 | LG2 |  |  |  |  |
| DLA_LG2_006090 | LG2 |  |  |  |  |
| DLA_LG2_006100 | LG2 |  |  |  |  |
| DLA_LG2_006110 | LG2 |  |  |  |  |
| DLA_LG2_006120 | LG2 |  |  |  |  |
| DLA_LG2_006130 | LG2 |  |  |  |  |
| DLA_LG2_006145_2 | LG2 |  |  |  |  |
| DLA_LG2_006150 | LG2 |  |  |  |  |
| DLA_LG2_006160 | LG2 |  |  |  |  |
| DLA_LG2_006170 | LG2 |  |  |  |  |
| DLA_LG2_006180 | LG2 |  |  |  |  |
| DLA_LG2_006190 | LG2 |  |  |  |  |
| DLA_LG2_006200 | LG2 |  |  |  |  |
| DLA_LG2_006210 | LG2 |  |  |  |  |
| DLA_LG2_006220 | LG2 |  |  |  |  |
| DLA_LG2_006230 | LG2 |  |  |  |  |
| DLA_LG2_006240 | LG2 |  |  |  |  |
| DLA_LG2_006255 | LG2 |  |  |  |  |
| DLA_LG2_006260 | LG2 |  |  |  |  |
| DLA_LG2_006270 | LG2 |  |  |  |  |
| DLA_LG2_006280 | LG2 |  |  |  |  |
| DLA_LG2_006300 | LG2 |  |  |  |  |
| DLA_LG2_006310 | LG2 |  |  |  |  |
| DLA_LG2_006320 | LG2 |  |  |  |  |
| DLA_LG2_006330 | LG2 |  |  |  |  |
| DLA_LG2_006340 | LG2 |  |  |  |  |
| DLA_LG2_006350 | LG2 |  |  |  |  |
| DLA_LG2_006360 | LG2 |  |  |  |  |
| DLA_LG2_006370 | LG2 |  |  |  |  |
| DLA_LG2_006380 | LG2 |  |  |  |  |
| DLA_LG2_006390 | LG2 |  |  |  |  |
| DLA_LG2_006400 | LG2 |  |  |  |  |
| DLA_LG2_006410 | LG2 |  |  |  |  |
| DLA_LG2_006420 | LG2 |  |  |  |  |
| DLA_LG2_006430 | LG2 |  |  |  |  |
| DLA_LG2_006440 | LG2 |  |  |  |  |
| DLA_LG2_006450 | LG2 |  |  |  |  |
| DLA_LG2_006460 | LG2 |  |  |  |  |
| DLA_LG2_006470 | LG2 |  |  |  |  |
| DLA_LG2_006490 | LG2 |  |  |  |  |
| DLA_LG2_006500 | LG2 |  |  |  |  |
| DLA_LG2_006515 | LG2 |  |  |  |  |
| DLA_LG2_006550 | LG2 |  |  |  |  |
| DLA_LG2_006560 | LG2 |  |  |  |  |
| DLA_LG2_006580 | LG2 |  |  |  |  |
| DLA_LG2_006590 | LG2 |  |  |  |  |
| DLA_LG2_006600 | LG2 |  |  |  |  |
| DLA_LG2_006610 | LG2 |  |  |  |  |
| DLA_LG2_006620 | LG2 |  |  |  |  |
| DLA_LG2_006630 | LG2 |  |  |  |  |
| DLA_LG2_006640 | LG2 |  |  |  |  |
| DLA_LG2_006660 | LG2 |  |  |  |  |
| DLA_LG2_006670 | LG2 |  |  |  |  |
| DLA_LG2_006680 | LG2 |  |  |  |  |
| DLA_LG2_006690 | LG2 |  |  |  |  |
| DLA_LG2_006700 | LG2 |  |  |  |  |
| DLA_LG2_006710 | LG2 |  |  |  |  |
| DLA_LG2_006720 | LG2 |  |  |  |  |
| DLA_LG2_006740 | LG2 |  |  |  |  |
| DLA_LG2_006760 | LG2 |  |  |  |  |
| DLA_LG2_006790 | LG2 |  |  |  |  |
| DLA_LG2_006800 | LG2 |  |  |  |  |
| DLA_LG2_006820 | LG2 |  |  |  |  |
| DLA_LG2_006830 | LG2 |  |  |  |  |
| DLA_LG2_006840 | LG2 |  |  |  |  |
| DLA_LG2_006860 | LG2 |  |  |  |  |
| DLA_LG2_006870 | LG2 |  |  |  |  |
| DLA_LG2_006880 | LG2 |  |  |  |  |
| DLA_LG2_006900 | LG2 |  |  |  |  |
| DLA_LG2_006910 | LG2 |  |  |  |  |
| DLA_LG2_006915 | LG2 |  |  |  |  |
| DLA_LG2_006920 | LG2 |  |  |  |  |
| DLA_LG2_006930 | LG2 |  |  |  |  |
| DLA_LG2_006940 | LG2 |  |  |  |  |
| DLA_LG2_006950 | LG2 |  |  |  |  |
| DLA_LG2_006960 | LG2 |  |  |  |  |
| DLA_LG2_006970 | LG2 |  |  |  |  |
| DLA_LG2_006980 | LG2 |  |  |  |  |
| DLA_LG2_006990 | LG2 |  |  |  |  |
| DLA_LG2_007000 | LG2 |  |  |  |  |
| DLA_LG2_007010 | LG2 |  |  |  |  |
| DLA_LG2_007030 | LG2 |  |  |  |  |
| DLA_LG2_007040 | LG2 |  |  |  |  |
| DLA_LG2_007050 | LG2 |  |  |  |  |
| DLA_LG2_007060 | LG2 |  |  |  |  |
| DLA_LG2_007070 | LG2 |  |  |  |  |
| DLA_LG2_007080 | LG2 |  |  |  |  |
| DLA_LG2_007090 | LG2 |  |  |  |  |
| DLA_LG2_007100 | LG2 |  |  |  |  |
| DLA_LG2_007120 | LG2 |  |  |  |  |
| DLA_LG2_007130 | LG2 |  |  |  |  |
| DLA_LG2_007135 | LG2 |  |  |  |  |
| DLA_LG2_007135_2 | LG2 |  |  |  |  |
| DLA_LG3_000860 | LG3 |  |  |  |  |
| DLA_LG3_001520 | LG3 |  |  |  |  |
| DLA_LG3_002020 | LG3 |  |  |  |  |
| DLA_LG3_003150 | LG3 |  |  |  |  |
| DLA_LG3_000440 | LG3 |  |  |  |  |
| DLA_LG3_000510 | LG3 |  |  |  |  |
| DLA_LG3_000520 | LG3 |  |  |  |  |
| DLA_LG3_000550 | LG3 |  |  |  |  |
| DLA_LG3_000580 | LG3 |  |  |  |  |
| DLA_LG3_000640 | LG3 |  |  |  |  |
| DLA_LG3_000650 | LG3 |  |  |  |  |
| DLA_LG3_000680 | LG3 |  |  |  |  |
| DLA_LG3_000700 | LG3 |  |  |  |  |
| DLA_LG3_000710 | LG3 |  |  |  |  |
| DLA_LG3_000720 | LG3 |  |  |  |  |
| DLA_LG3_000790 | LG3 |  |  |  |  |
| DLA_LG3_000850 | LG3 |  |  |  |  |
| DLA_LG3_000970 | LG3 |  |  |  |  |
| DLA_LG3_001050 | LG3 |  |  |  |  |
| DLA_LG3_001260 | LG3 |  |  |  |  |
| DLA_LG3_001330 | LG3 |  |  |  |  |
| DLA_LG3_001420 | LG3 |  |  |  |  |
| DLA_LG3_001610 | LG3 |  |  |  |  |
| DLA_LG3_001670 | LG3 |  |  |  |  |
| DLA_LG3_001700 | LG3 |  |  |  |  |
| DLA_LG3_001920 | LG3 |  |  |  |  |
| DLA_LG3_001970 | LG3 |  |  |  |  |
| DLA_LG3_001990 | LG3 |  |  |  |  |
| DLA_LG3_002000 | LG3 |  |  |  |  |
| DLA_LG3_002080 | LG3 |  |  |  |  |
| DLA_LG3_002180 | LG3 |  |  |  |  |
| DLA_LG3_002185 | LG3 |  |  |  |  |
| DLA_LG3_002230 | LG3 |  |  |  |  |
| DLA_LG3_002380 | LG3 |  |  |  |  |
| DLA_LG3_002460 | LG3 |  |  |  |  |
| DLA_LG3_002740 | LG3 |  |  |  |  |
| DLA_LG3_002780 | LG3 |  |  |  |  |
| DLA_LG3_002945 | LG3 |  |  |  |  |
| DLA_LG3_003010 | LG3 |  |  |  |  |
| DLA_LG3_003210 | LG3 |  |  |  |  |
| DLA_LG3_003430 | LG3 |  |  |  |  |
| DLA_LG3_003480 | LG3 |  |  |  |  |
| DLA_LG3_003520 | LG3 |  |  |  |  |
| DLA_LG3_000005 | LG3 |  |  |  |  |
| DLA_LG3_000010 | LG3 |  |  |  |  |
| DLA_LG3_000020 | LG3 |  |  |  |  |
| DLA_LG3_000025 | LG3 |  |  |  |  |
| DLA_LG3_000030 | LG3 |  |  |  |  |
| DLA_LG3_000040 | LG3 |  |  |  |  |
| DLA_LG3_000050 | LG3 |  |  |  |  |
| DLA_LG3_000060 | LG3 |  |  |  |  |
| DLA_LG3_000065 | LG3 |  |  |  |  |
| DLA_LG3_000080 | LG3 |  |  |  |  |
| DLA_LG3_000090 | LG3 |  |  |  |  |
| DLA_LG3_000100 | LG3 |  |  |  |  |
| DLA_LG3_000110 | LG3 |  |  |  |  |
| DLA_LG3_000120 | LG3 |  |  |  |  |
| DLA_LG3_000125 | LG3 |  |  |  |  |
| DLA_LG3_000125_2 | LG3 |  |  |  |  |
| DLA_LG3_000130 | LG3 |  |  |  |  |
| DLA_LG3_000135 | LG3 |  |  |  |  |
| DLA_LG3_000140 | LG3 |  |  |  |  |
| DLA_LG3_000150 | LG3 |  |  |  |  |
| DLA_LG3_000160 | LG3 |  |  |  |  |
| DLA_LG3_000170 | LG3 |  |  |  |  |
| DLA_LG3_000180 | LG3 |  |  |  |  |
| DLA_LG3_000190 | LG3 |  |  |  |  |
| DLA_LG3_000200 | LG3 |  |  |  |  |
| DLA_LG3_000210 | LG3 |  |  |  |  |
| DLA_LG3_000220 | LG3 |  |  |  |  |
| DLA_LG3_000230 | LG3 |  |  |  |  |
| DLA_LG3_000240 | LG3 |  |  |  |  |
| DLA_LG3_000250 | LG3 |  |  |  |  |
| DLA_LG3_000260 | LG3 |  |  |  |  |
| DLA_LG3_000270 | LG3 |  |  |  |  |
| DLA_LG3_000280 | LG3 |  |  |  |  |
| DLA_LG3_000290 | LG3 |  |  |  |  |
| DLA_LG3_000300 | LG3 |  |  |  |  |
| DLA_LG3_000310 | LG3 |  |  |  |  |
| DLA_LG3_000330 | LG3 |  |  |  |  |
| DLA_LG3_000340 | LG3 |  |  |  |  |
| DLA_LG3_000345 | LG3 |  |  |  |  |
| DLA_LG3_000350 | LG3 |  |  |  |  |
| DLA_LG3_000360 | LG3 |  |  |  |  |
| DLA_LG3_000365 | LG3 |  |  |  |  |
| DLA_LG3_000370 | LG3 |  |  |  |  |
| DLA_LG3_000380 | LG3 |  |  |  |  |
| DLA_LG3_000390 | LG3 |  |  |  |  |
| DLA_LG3_000400 | LG3 |  |  |  |  |
| DLA_LG3_000410 | LG3 |  |  |  |  |
| DLA_LG3_000420 | LG3 |  |  |  |  |
| DLA_LG3_000430 | LG3 |  |  |  |  |
| DLA_LG3_000450 | LG3 |  |  |  |  |
| DLA_LG3_000460 | LG3 |  |  |  |  |
| DLA_LG3_000470 | LG3 |  |  |  |  |
| DLA_LG3_000480 | LG3 |  |  |  |  |
| DLA_LG3_000500 | LG3 |  |  |  |  |
| DLA_LG3_000530 | LG3 |  |  |  |  |
| DLA_LG3_000535 | LG3 |  |  |  |  |
| DLA_LG3_000540 | LG3 |  |  |  |  |
| DLA_LG3_000555 | LG3 |  |  |  |  |
| DLA_LG3_000555_2 | LG3 |  |  |  |  |
| DLA_LG3_000560 | LG3 |  |  |  |  |
| DLA_LG3_000570 | LG3 |  |  |  |  |
| DLA_LG3_000590 | LG3 |  |  |  |  |
| DLA_LG3_000600 | LG3 |  |  |  |  |
| DLA_LG3_000615 | LG3 |  |  |  |  |
| DLA_LG3_000630 | LG3 |  |  |  |  |
| DLA_LG3_000660 | LG3 |  |  |  |  |
| DLA_LG3_000670 | LG3 |  |  |  |  |
| DLA_LG3_000690 | LG3 |  |  |  |  |
| DLA_LG3_000730 | LG3 |  |  |  |  |
| DLA_LG3_000740 | LG3 |  |  |  |  |
| DLA_LG3_000745 | LG3 |  |  |  |  |
| DLA_LG3_000750 | LG3 |  |  |  |  |
| DLA_LG3_000760 | LG3 |  |  |  |  |
| DLA_LG3_000770 | LG3 |  |  |  |  |
| DLA_LG3_000780 | LG3 |  |  |  |  |
| DLA_LG3_000800 | LG3 |  |  |  |  |
| DLA_LG3_000820 | LG3 |  |  |  |  |
| DLA_LG3_000830 | LG3 |  |  |  |  |
| DLA_LG3_000870 | LG3 |  |  |  |  |
| DLA_LG3_000875 | LG3 |  |  |  |  |
| DLA_LG3_000880 | LG3 |  |  |  |  |
| DLA_LG3_000900 | LG3 |  |  |  |  |
| DLA_LG3_000910 | LG3 |  |  |  |  |
| DLA_LG3_000915 | LG3 |  |  |  |  |
| DLA_LG3_000920 | LG3 |  |  |  |  |
| DLA_LG3_000930 | LG3 |  |  |  |  |
| DLA_LG3_000940 | LG3 |  |  |  |  |
| DLA_LG3_000950 | LG3 |  |  |  |  |
| DLA_LG3_000960 | LG3 |  |  |  |  |
| DLA_LG3_000985 | LG3 |  |  |  |  |
| DLA_LG3_000985_2 | LG3 |  |  |  |  |
| DLA_LG3_000985_3 | LG3 |  |  |  |  |
| DLA_LG3_000985_4 | LG3 |  |  |  |  |
| DLA_LG3_000985_5 | LG3 |  |  |  |  |
| DLA_LG3_000985_6 | LG3 |  |  |  |  |
| DLA_LG3_000985_7 | LG3 |  |  |  |  |
| DLA_LG3_001000 | LG3 |  |  |  |  |
| DLA_LG3_001010 | LG3 |  |  |  |  |
| DLA_LG3_001020 | LG3 |  |  |  |  |
| DLA_LG3_001030 | LG3 |  |  |  |  |
| DLA_LG3_001040 | LG3 |  |  |  |  |
| DLA_LG3_001060 | LG3 |  |  |  |  |
| DLA_LG3_001070 | LG3 |  |  |  |  |
| DLA_LG3_001080 | LG3 |  |  |  |  |
| DLA_LG3_001090 | LG3 |  |  |  |  |
| DLA_LG3_001100 | LG3 |  |  |  |  |
| DLA_LG3_001130 | LG3 |  |  |  |  |
| DLA_LG3_001150 | LG3 |  |  |  |  |
| DLA_LG3_001160 | LG3 |  |  |  |  |
| DLA_LG3_001170 | LG3 |  |  |  |  |
| DLA_LG3_001180 | LG3 |  |  |  |  |
| DLA_LG3_001190 | LG3 |  |  |  |  |
| DLA_LG3_001200 | LG3 |  |  |  |  |
| DLA_LG3_001210 | LG3 |  |  |  |  |
| DLA_LG3_001220 | LG3 |  |  |  |  |
| DLA_LG3_001230 | LG3 |  |  |  |  |
| DLA_LG3_001240 | LG3 |  |  |  |  |
| DLA_LG3_001250 | LG3 |  |  |  |  |
| DLA_LG3_001270 | LG3 |  |  |  |  |
| DLA_LG3_001280 | LG3 |  |  |  |  |
| DLA_LG3_001290 | LG3 |  |  |  |  |
| DLA_LG3_001300 | LG3 |  |  |  |  |
| DLA_LG3_001310 | LG3 |  |  |  |  |
| DLA_LG3_001320 | LG3 |  |  |  |  |
| DLA_LG3_001340 | LG3 |  |  |  |  |
| DLA_LG3_001350 | LG3 |  |  |  |  |
| DLA_LG3_001360 | LG3 |  |  |  |  |
| DLA_LG3_001370 | LG3 |  |  |  |  |
| DLA_LG3_001380 | LG3 |  |  |  |  |
| DLA_LG3_001390 | LG3 |  |  |  |  |
| DLA_LG3_001400 | LG3 |  |  |  |  |
| DLA_LG3_001410 | LG3 |  |  |  |  |
| DLA_LG3_001430 | LG3 |  |  |  |  |
| DLA_LG3_001440 | LG3 |  |  |  |  |
| DLA_LG3_001450 | LG3 |  |  |  |  |
| DLA_LG3_001455 | LG3 |  |  |  |  |
| DLA_LG3_001460 | LG3 |  |  |  |  |
| DLA_LG3_001470 | LG3 |  |  |  |  |
| DLA_LG3_001480 | LG3 |  |  |  |  |
| DLA_LG3_001490 | LG3 |  |  |  |  |
| DLA_LG3_001500 | LG3 |  |  |  |  |
| DLA_LG3_001510 | LG3 |  |  |  |  |
| DLA_LG3_001530 | LG3 |  |  |  |  |
| DLA_LG3_001540 | LG3 |  |  |  |  |
| DLA_LG3_001550 | LG3 |  |  |  |  |
| DLA_LG3_001560 | LG3 |  |  |  |  |
| DLA_LG3_001570 | LG3 |  |  |  |  |
| DLA_LG3_001580 | LG3 |  |  |  |  |
| DLA_LG3_001590 | LG3 |  |  |  |  |
| DLA_LG3_001600 | LG3 |  |  |  |  |
| DLA_LG3_001620 | LG3 |  |  |  |  |
| DLA_LG3_001630 | LG3 |  |  |  |  |
| DLA_LG3_001640 | LG3 |  |  |  |  |
| DLA_LG3_001650 | LG3 |  |  |  |  |
| DLA_LG3_001655 | LG3 |  |  |  |  |
| DLA_LG3_001660 | LG3 |  |  |  |  |
| DLA_LG3_001680 | LG3 |  |  |  |  |
| DLA_LG3_001690 | LG3 |  |  |  |  |
| DLA_LG3_001710 | LG3 |  |  |  |  |
| DLA_LG3_001720 | LG3 |  |  |  |  |
| DLA_LG3_001730 | LG3 |  |  |  |  |
| DLA_LG3_001740 | LG3 |  |  |  |  |
| DLA_LG3_001750 | LG3 |  |  |  |  |
| DLA_LG3_001755 | LG3 |  |  |  |  |
| DLA_LG3_001760 | LG3 |  |  |  |  |
| DLA_LG3_001770 | LG3 |  |  |  |  |
| DLA_LG3_001780 | LG3 |  |  |  |  |
| DLA_LG3_001790 | LG3 |  |  |  |  |
| DLA_LG3_001800 | LG3 |  |  |  |  |
| DLA_LG3_001810 | LG3 |  |  |  |  |
| DLA_LG3_001820 | LG3 |  |  |  |  |
| DLA_LG3_001830 | LG3 |  |  |  |  |
| DLA_LG3_001840 | LG3 |  |  |  |  |
| DLA_LG3_001845 | LG3 |  |  |  |  |
| DLA_LG3_001845_2 | LG3 |  |  |  |  |
| DLA_LG3_001845_3 | LG3 |  |  |  |  |
| DLA_LG3_001850 | LG3 |  |  |  |  |
| DLA_LG3_001860 | LG3 |  |  |  |  |
| DLA_LG3_001870 | LG3 |  |  |  |  |
| DLA_LG3_001880 | LG3 |  |  |  |  |
| DLA_LG3_001890 | LG3 |  |  |  |  |
| DLA_LG3_001900 | LG3 |  |  |  |  |
| DLA_LG3_001910 | LG3 |  |  |  |  |
| DLA_LG3_001930 | LG3 |  |  |  |  |
| DLA_LG3_001940 | LG3 |  |  |  |  |
| DLA_LG3_001945 | LG3 |  |  |  |  |
| DLA_LG3_001950 | LG3 |  |  |  |  |
| DLA_LG3_001960 | LG3 |  |  |  |  |
| DLA_LG3_001980 | LG3 |  |  |  |  |
| DLA_LG3_002010 | LG3 |  |  |  |  |
| DLA_LG3_002030 | LG3 |  |  |  |  |
| DLA_LG3_002040 | LG3 |  |  |  |  |
| DLA_LG3_002050 | LG3 |  |  |  |  |
| DLA_LG3_002060 | LG3 |  |  |  |  |
| DLA_LG3_002070 | LG3 |  |  |  |  |
| DLA_LG3_002090 | LG3 |  |  |  |  |
| DLA_LG3_002100 | LG3 |  |  |  |  |
| DLA_LG3_002110 | LG3 |  |  |  |  |
| DLA_LG3_002120 | LG3 |  |  |  |  |
| DLA_LG3_002130 | LG3 |  |  |  |  |
| DLA_LG3_002140 | LG3 |  |  |  |  |
| DLA_LG3_002150 | LG3 |  |  |  |  |
| DLA_LG3_002160 | LG3 |  |  |  |  |
| DLA_LG3_002170 | LG3 |  |  |  |  |
| DLA_LG3_002190 | LG3 |  |  |  |  |
| DLA_LG3_002200 | LG3 |  |  |  |  |
| DLA_LG3_002205 | LG3 |  |  |  |  |
| DLA_LG3_002210 | LG3 |  |  |  |  |
| DLA_LG3_002220 | LG3 |  |  |  |  |
| DLA_LG3_002250 | LG3 |  |  |  |  |
| DLA_LG3_002260 | LG3 |  |  |  |  |
| DLA_LG3_002270 | LG3 |  |  |  |  |
| DLA_LG3_002280 | LG3 |  |  |  |  |
| DLA_LG3_002290 | LG3 |  |  |  |  |
| DLA_LG3_002300 | LG3 |  |  |  |  |
| DLA_LG3_002310 | LG3 |  |  |  |  |
| DLA_LG3_002320 | LG3 |  |  |  |  |
| DLA_LG3_002330 | LG3 |  |  |  |  |
| DLA_LG3_002340 | LG3 |  |  |  |  |
| DLA_LG3_002350 | LG3 |  |  |  |  |
| DLA_LG3_002360 | LG3 |  |  |  |  |
| DLA_LG3_002370 | LG3 |  |  |  |  |
| DLA_LG3_002390 | LG3 |  |  |  |  |
| DLA_LG3_002395 | LG3 |  |  |  |  |
| DLA_LG3_002400 | LG3 |  |  |  |  |
| DLA_LG3_002410 | LG3 |  |  |  |  |
| DLA_LG3_002420 | LG3 |  |  |  |  |
| DLA_LG3_002430 | LG3 |  |  |  |  |
| DLA_LG3_002440 | LG3 |  |  |  |  |
| DLA_LG3_002450 | LG3 |  |  |  |  |
| DLA_LG3_002470 | LG3 |  |  |  |  |
| DLA_LG3_002500 | LG3 |  |  |  |  |
| DLA_LG3_002510 | LG3 |  |  |  |  |
| DLA_LG3_002520 | LG3 |  |  |  |  |
| DLA_LG3_002530 | LG3 |  |  |  |  |
| DLA_LG3_002540 | LG3 |  |  |  |  |
| DLA_LG3_002550 | LG3 |  |  |  |  |
| DLA_LG3_002560 | LG3 |  |  |  |  |
| DLA_LG3_002565 | LG3 |  |  |  |  |
| DLA_LG3_002570 | LG3 |  |  |  |  |
| DLA_LG3_002580 | LG3 |  |  |  |  |
| DLA_LG3_002590 | LG3 |  |  |  |  |
| DLA_LG3_002600 | LG3 |  |  |  |  |
| DLA_LG3_002610 | LG3 |  |  |  |  |
| DLA_LG3_002620 | LG3 |  |  |  |  |
| DLA_LG3_002630 | LG3 |  |  |  |  |
| DLA_LG3_002640 | LG3 |  |  |  |  |
| DLA_LG3_002650 | LG3 |  |  |  |  |
| DLA_LG3_002660 | LG3 |  |  |  |  |
| DLA_LG3_002670 | LG3 |  |  |  |  |
| DLA_LG3_002675 | LG3 |  |  |  |  |
| DLA_LG3_002680 | LG3 |  |  |  |  |
| DLA_LG3_002690 | LG3 |  |  |  |  |
| DLA_LG3_002700 | LG3 |  |  |  |  |
| DLA_LG3_002720 | LG3 |  |  |  |  |
| DLA_LG3_002730 | LG3 |  |  |  |  |
| DLA_LG3_002750 | LG3 |  |  |  |  |
| DLA_LG3_002760 | LG3 |  |  |  |  |
| DLA_LG3_002770 | LG3 |  |  |  |  |
| DLA_LG3_002790 | LG3 |  |  |  |  |
| DLA_LG3_002800 | LG3 |  |  |  |  |
| DLA_LG3_002810 | LG3 |  |  |  |  |
| DLA_LG3_002820 | LG3 |  |  |  |  |
| DLA_LG3_002830 | LG3 |  |  |  |  |
| DLA_LG3_002840 | LG3 |  |  |  |  |
| DLA_LG3_002850 | LG3 |  |  |  |  |
| DLA_LG3_002860 | LG3 |  |  |  |  |
| DLA_LG3_002870 | LG3 |  |  |  |  |
| DLA_LG3_002880 | LG3 |  |  |  |  |
| DLA_LG3_002890 | LG3 |  |  |  |  |
| DLA_LG3_002900 | LG3 |  |  |  |  |
| DLA_LG3_002910 | LG3 |  |  |  |  |
| DLA_LG3_002915 | LG3 |  |  |  |  |
| DLA_LG3_002920 | LG3 |  |  |  |  |
| DLA_LG3_002930 | LG3 |  |  |  |  |
| DLA_LG3_002940 | LG3 |  |  |  |  |
| DLA_LG3_002950 | LG3 |  |  |  |  |
| DLA_LG3_002960 | LG3 |  |  |  |  |
| DLA_LG3_002965 | LG3 |  |  |  |  |
| DLA_LG3_002970 | LG3 |  |  |  |  |
| DLA_LG3_002980 | LG3 |  |  |  |  |
| DLA_LG3_002985 | LG3 |  |  |  |  |
| DLA_LG3_002985_2 | LG3 |  |  |  |  |
| DLA_LG3_002990 | LG3 |  |  |  |  |
| DLA_LG3_002995 | LG3 |  |  |  |  |
| DLA_LG3_003000 | LG3 |  |  |  |  |
| DLA_LG3_003020 | LG3 |  |  |  |  |
| DLA_LG3_003030 | LG3 |  |  |  |  |
| DLA_LG3_003040 | LG3 |  |  |  |  |
| DLA_LG3_003050 | LG3 |  |  |  |  |
| DLA_LG3_003060 | LG3 |  |  |  |  |
| DLA_LG3_003070 | LG3 |  |  |  |  |
| DLA_LG3_003080 | LG3 |  |  |  |  |
| DLA_LG3_003085 | LG3 |  |  |  |  |
| DLA_LG3_003085_2 | LG3 |  |  |  |  |
| DLA_LG3_003085_3 | LG3 |  |  |  |  |
| DLA_LG3_003090 | LG3 |  |  |  |  |
| DLA_LG3_003095 | LG3 |  |  |  |  |
| DLA_LG3_003100 | LG3 |  |  |  |  |
| DLA_LG3_003110 | LG3 |  |  |  |  |
| DLA_LG3_003120 | LG3 |  |  |  |  |
| DLA_LG3_003130 | LG3 |  |  |  |  |
| DLA_LG3_003140 | LG3 |  |  |  |  |
| DLA_LG3_003160 | LG3 |  |  |  |  |
| DLA_LG3_003170 | LG3 |  |  |  |  |
| DLA_LG3_003180 | LG3 |  |  |  |  |
| DLA_LG3_003190 | LG3 |  |  |  |  |
| DLA_LG3_003200 | LG3 |  |  |  |  |
| DLA_LG3_003220 | LG3 |  |  |  |  |
| DLA_LG3_003230 | LG3 |  |  |  |  |
| DLA_LG3_003240 | LG3 |  |  |  |  |
| DLA_LG3_003250 | LG3 |  |  |  |  |
| DLA_LG3_003260 | LG3 |  |  |  |  |
| DLA_LG3_003270 | LG3 |  |  |  |  |
| DLA_LG3_003280 | LG3 |  |  |  |  |
| DLA_LG3_003285 | LG3 |  |  |  |  |
| DLA_LG3_003290 | LG3 |  |  |  |  |
| DLA_LG3_003310 | LG3 |  |  |  |  |
| DLA_LG3_003320 | LG3 |  |  |  |  |
| DLA_LG3_003330 | LG3 |  |  |  |  |
| DLA_LG3_003340 | LG3 |  |  |  |  |
| DLA_LG3_003350 | LG3 |  |  |  |  |
| DLA_LG3_003360 | LG3 |  |  |  |  |
| DLA_LG3_003370 | LG3 |  |  |  |  |
| DLA_LG3_003380 | LG3 |  |  |  |  |
| DLA_LG3_003390 | LG3 |  |  |  |  |
| DLA_LG3_003400 | LG3 |  |  |  |  |
| DLA_LG3_003410 | LG3 |  |  |  |  |
| DLA_LG3_003420 | LG3 |  |  |  |  |
| DLA_LG3_003440 | LG3 |  |  |  |  |
| DLA_LG3_003450 | LG3 |  |  |  |  |
| DLA_LG3_003460 | LG3 |  |  |  |  |
| DLA_LG3_003470 | LG3 |  |  |  |  |
| DLA_LG3_003490 | LG3 |  |  |  |  |
| DLA_LG3_003500 | LG3 |  |  |  |  |
| DLA_LG3_003510 | LG3 |  |  |  |  |
| DLA_LG3_003530 | LG3 |  |  |  |  |
| DLA_LG3_003540 | LG3 |  |  |  |  |
| DLA_LG3_003550 | LG3 |  |  |  |  |
| DLA_LG4_008635 | LG4 |  |  |  |  |
| DLA_LG4_000440 | LG4 |  |  |  |  |
| DLA_LG4_001210 | LG4 |  |  |  |  |
| DLA_LG4_001650 | LG4 |  |  |  |  |
| DLA_LG4_003170 | LG4 |  |  |  |  |
| DLA_LG4_004440 | LG4 |  |  |  |  |
| DLA_LG4_005390 | LG4 |  |  |  |  |
| DLA_LG4_006350 | LG4 |  |  |  |  |
| DLA_LG4_007480 | LG4 |  |  |  |  |
| DLA_LG4_008000 | LG4 |  |  |  |  |
| DLA_LG4_008260 | LG4 |  |  |  |  |
| DLA_LG4_008800 | LG4 |  |  |  |  |
| DLA_LG4_000020 | LG4 |  |  |  |  |
| DLA_LG4_000150 | LG4 |  |  |  |  |
| DLA_LG4_000180 | LG4 |  |  |  |  |
| DLA_LG4_000400 | LG4 |  |  |  |  |
| DLA_LG4_000550 | LG4 |  |  |  |  |
| DLA_LG4_000750 | LG4 |  |  |  |  |
| DLA_LG4_000815_3 | LG4 |  |  |  |  |
| DLA_LG4_000820 | LG4 |  |  |  |  |
| DLA_LG4_000970 | LG4 |  |  |  |  |
| DLA_LG4_001300 | LG4 |  |  |  |  |
| DLA_LG4_001340 | LG4 |  |  |  |  |
| DLA_LG4_001460 | LG4 |  |  |  |  |
| DLA_LG4_001500 | LG4 |  |  |  |  |
| DLA_LG4_001520 | LG4 |  |  |  |  |
| DLA_LG4_001550 | LG4 |  |  |  |  |
| DLA_LG4_001630 | LG4 |  |  |  |  |
| DLA_LG4_001680 | LG4 |  |  |  |  |
| DLA_LG4_001730 | LG4 |  |  |  |  |
| DLA_LG4_002050 | LG4 |  |  |  |  |
| DLA_LG4_002270 | LG4 |  |  |  |  |
| DLA_LG4_002570 | LG4 |  |  |  |  |
| DLA_LG4_002620 | LG4 |  |  |  |  |
| DLA_LG4_002680 | LG4 |  |  |  |  |
| DLA_LG4_002950 | LG4 |  |  |  |  |
| DLA_LG4_003010 | LG4 |  |  |  |  |
| DLA_LG4_003060 | LG4 |  |  |  |  |
| DLA_LG4_003200 | LG4 |  |  |  |  |
| DLA_LG4_003350 | LG4 |  |  |  |  |
| DLA_LG4_003470 | LG4 |  |  |  |  |
| DLA_LG4_003480 | LG4 |  |  |  |  |
| DLA_LG4_003640 | LG4 |  |  |  |  |
| DLA_LG4_003800 | LG4 |  |  |  |  |
| DLA_LG4_003830 | LG4 |  |  |  |  |
| DLA_LG4_003970 | LG4 |  |  |  |  |
| DLA_LG4_004160 | LG4 |  |  |  |  |
| DLA_LG4_004500 | LG4 |  |  |  |  |
| DLA_LG4_004650 | LG4 |  |  |  |  |
| DLA_LG4_004730 | LG4 |  |  |  |  |
| DLA_LG4_005120 | LG4 |  |  |  |  |
| DLA_LG4_005160 | LG4 |  |  |  |  |
| DLA_LG4_005280 | LG4 |  |  |  |  |
| DLA_LG4_005320 | LG4 |  |  |  |  |
| DLA_LG4_005450 | LG4 |  |  |  |  |
| DLA_LG4_005540 | LG4 |  |  |  |  |
| DLA_LG4_005870 | LG4 |  |  |  |  |
| DLA_LG4_005910 | LG4 |  |  |  |  |
| DLA_LG4_006020 | LG4 |  |  |  |  |
| DLA_LG4_006130 | LG4 |  |  |  |  |
| DLA_LG4_006540 | LG4 |  |  |  |  |
| DLA_LG4_006720 | LG4 |  |  |  |  |
| DLA_LG4_006820 | LG4 |  |  |  |  |
| DLA_LG4_006940 | LG4 |  |  |  |  |
| DLA_LG4_007040 | LG4 |  |  |  |  |
| DLA_LG4_007130 | LG4 |  |  |  |  |
| DLA_LG4_007210 | LG4 |  |  |  |  |
| DLA_LG4_007630 | LG4 |  |  |  |  |
| DLA_LG4_007690 | LG4 |  |  |  |  |
| DLA_LG4_007720 | LG4 |  |  |  |  |
| DLA_LG4_007770 | LG4 |  |  |  |  |
| DLA_LG4_007900 | LG4 |  |  |  |  |
| DLA_LG4_008040 | LG4 |  |  |  |  |
| DLA_LG4_008140 | LG4 |  |  |  |  |
| DLA_LG4_008190 | LG4 |  |  |  |  |
| DLA_LG4_008300 | LG4 |  |  |  |  |
| DLA_LG4_008540 | LG4 |  |  |  |  |
| DLA_LG4_008650 | LG4 |  |  |  |  |
| DLA_LG4_008710 | LG4 |  |  |  |  |
| DLA_LG4_008730 | LG4 |  |  |  |  |
| DLA_LG4_008780 | LG4 |  |  |  |  |
| DLA_LG4_000010 | LG4 |  |  |  |  |
| DLA_LG4_000030 | LG4 |  |  |  |  |
| DLA_LG4_000040 | LG4 |  |  |  |  |
| DLA_LG4_000060 | LG4 |  |  |  |  |
| DLA_LG4_000070 | LG4 |  |  |  |  |
| DLA_LG4_000075 | LG4 |  |  |  |  |
| DLA_LG4_000080 | LG4 |  |  |  |  |
| DLA_LG4_000090 | LG4 |  |  |  |  |
| DLA_LG4_000100 | LG4 |  |  |  |  |
| DLA_LG4_000110 | LG4 |  |  |  |  |
| DLA_LG4_000120 | LG4 |  |  |  |  |
| DLA_LG4_000130 | LG4 |  |  |  |  |
| DLA_LG4_000140 | LG4 |  |  |  |  |
| DLA_LG4_000160 | LG4 |  |  |  |  |
| DLA_LG4_000170 | LG4 |  |  |  |  |
| DLA_LG4_000190 | LG4 |  |  |  |  |
| DLA_LG4_000200 | LG4 |  |  |  |  |
| DLA_LG4_000210 | LG4 |  |  |  |  |
| DLA_LG4_000220 | LG4 |  |  |  |  |
| DLA_LG4_000230 | LG4 |  |  |  |  |
| DLA_LG4_000240 | LG4 |  |  |  |  |
| DLA_LG4_000250 | LG4 |  |  |  |  |
| DLA_LG4_000270 | LG4 |  |  |  |  |
| DLA_LG4_000280 | LG4 |  |  |  |  |
| DLA_LG4_000290 | LG4 |  |  |  |  |
| DLA_LG4_000300 | LG4 |  |  |  |  |
| DLA_LG4_000305 | LG4 |  |  |  |  |
| DLA_LG4_000305_2 | LG4 |  |  |  |  |
| DLA_LG4_000310 | LG4 |  |  |  |  |
| DLA_LG4_000320 | LG4 |  |  |  |  |
| DLA_LG4_000330 | LG4 |  |  |  |  |
| DLA_LG4_000340 | LG4 |  |  |  |  |
| DLA_LG4_000350 | LG4 |  |  |  |  |
| DLA_LG4_000360 | LG4 |  |  |  |  |
| DLA_LG4_000370 | LG4 |  |  |  |  |
| DLA_LG4_000380 | LG4 |  |  |  |  |
| DLA_LG4_000390 | LG4 |  |  |  |  |
| DLA_LG4_000410 | LG4 |  |  |  |  |
| DLA_LG4_000420 | LG4 |  |  |  |  |
| DLA_LG4_000430 | LG4 |  |  |  |  |
| DLA_LG4_000450 | LG4 |  |  |  |  |
| DLA_LG4_000460 | LG4 |  |  |  |  |
| DLA_LG4_000470 | LG4 |  |  |  |  |
| DLA_LG4_000480 | LG4 |  |  |  |  |
| DLA_LG4_000490 | LG4 |  |  |  |  |
| DLA_LG4_000510 | LG4 |  |  |  |  |
| DLA_LG4_000530 | LG4 |  |  |  |  |
| DLA_LG4_000560 | LG4 |  |  |  |  |
| DLA_LG4_000570 | LG4 |  |  |  |  |
| DLA_LG4_000580 | LG4 |  |  |  |  |
| DLA_LG4_000590 | LG4 |  |  |  |  |
| DLA_LG4_000600 | LG4 |  |  |  |  |
| DLA_LG4_000610 | LG4 |  |  |  |  |
| DLA_LG4_000620 | LG4 |  |  |  |  |
| DLA_LG4_000630 | LG4 |  |  |  |  |
| DLA_LG4_000640 | LG4 |  |  |  |  |
| DLA_LG4_000650 | LG4 |  |  |  |  |
| DLA_LG4_000660 | LG4 |  |  |  |  |
| DLA_LG4_000670 | LG4 |  |  |  |  |
| DLA_LG4_000680 | LG4 |  |  |  |  |
| DLA_LG4_000690 | LG4 |  |  |  |  |
| DLA_LG4_000700 | LG4 |  |  |  |  |
| DLA_LG4_000710 | LG4 |  |  |  |  |
| DLA_LG4_000720 | LG4 |  |  |  |  |
| DLA_LG4_000730 | LG4 |  |  |  |  |
| DLA_LG4_000735 | LG4 |  |  |  |  |
| DLA_LG4_000740 | LG4 |  |  |  |  |
| DLA_LG4_000755 | LG4 |  |  |  |  |
| DLA_LG4_000760 | LG4 |  |  |  |  |
| DLA_LG4_000770 | LG4 |  |  |  |  |
| DLA_LG4_000780 | LG4 |  |  |  |  |
| DLA_LG4_000790 | LG4 |  |  |  |  |
| DLA_LG4_000800 | LG4 |  |  |  |  |
| DLA_LG4_000815 | LG4 |  |  |  |  |
| DLA_LG4_000815_2 | LG4 |  |  |  |  |
| DLA_LG4_000850 | LG4 |  |  |  |  |
| DLA_LG4_000860 | LG4 |  |  |  |  |
| DLA_LG4_000870 | LG4 |  |  |  |  |
| DLA_LG4_000880 | LG4 |  |  |  |  |
| DLA_LG4_000890 | LG4 |  |  |  |  |
| DLA_LG4_000910 | LG4 |  |  |  |  |
| DLA_LG4_000920 | LG4 |  |  |  |  |
| DLA_LG4_000925 | LG4 |  |  |  |  |
| DLA_LG4_000930 | LG4 |  |  |  |  |
| DLA_LG4_000940 | LG4 |  |  |  |  |
| DLA_LG4_000945 | LG4 |  |  |  |  |
| DLA_LG4_000950 | LG4 |  |  |  |  |
| DLA_LG4_000960 | LG4 |  |  |  |  |
| DLA_LG4_000980 | LG4 |  |  |  |  |
| DLA_LG4_000990 | LG4 |  |  |  |  |
| DLA_LG4_001000 | LG4 |  |  |  |  |
| DLA_LG4_001010 | LG4 |  |  |  |  |
| DLA_LG4_001020 | LG4 |  |  |  |  |
| DLA_LG4_001030 | LG4 |  |  |  |  |
| DLA_LG4_001040 | LG4 |  |  |  |  |
| DLA_LG4_001050 | LG4 |  |  |  |  |
| DLA_LG4_001060 | LG4 |  |  |  |  |
| DLA_LG4_001070 | LG4 |  |  |  |  |
| DLA_LG4_001080 | LG4 |  |  |  |  |
| DLA_LG4_001090 | LG4 |  |  |  |  |
| DLA_LG4_001100 | LG4 |  |  |  |  |
| DLA_LG4_001110 | LG4 |  |  |  |  |
| DLA_LG4_001120 | LG4 |  |  |  |  |
| DLA_LG4_001130 | LG4 |  |  |  |  |
| DLA_LG4_001160 | LG4 |  |  |  |  |
| DLA_LG4_001170 | LG4 |  |  |  |  |
| DLA_LG4_001175 | LG4 |  |  |  |  |
| DLA_LG4_001180 | LG4 |  |  |  |  |
| DLA_LG4_001190 | LG4 |  |  |  |  |
| DLA_LG4_001200 | LG4 |  |  |  |  |
| DLA_LG4_001220 | LG4 |  |  |  |  |
| DLA_LG4_001230 | LG4 |  |  |  |  |
| DLA_LG4_001240 | LG4 |  |  |  |  |
| DLA_LG4_001250 | LG4 |  |  |  |  |
| DLA_LG4_001260 | LG4 |  |  |  |  |
| DLA_LG4_001270 | LG4 |  |  |  |  |
| DLA_LG4_001280 | LG4 |  |  |  |  |
| DLA_LG4_001290 | LG4 |  |  |  |  |
| DLA_LG4_001310 | LG4 |  |  |  |  |
| DLA_LG4_001320 | LG4 |  |  |  |  |
| DLA_LG4_001330 | LG4 |  |  |  |  |
| DLA_LG4_001345 | LG4 |  |  |  |  |
| DLA_LG4_001350 | LG4 |  |  |  |  |
| DLA_LG4_001370 | LG4 |  |  |  |  |
| DLA_LG4_001380 | LG4 |  |  |  |  |
| DLA_LG4_001390 | LG4 |  |  |  |  |
| DLA_LG4_001400 | LG4 |  |  |  |  |
| DLA_LG4_001410 | LG4 |  |  |  |  |
| DLA_LG4_001420 | LG4 |  |  |  |  |
| DLA_LG4_001430 | LG4 |  |  |  |  |
| DLA_LG4_001440 | LG4 |  |  |  |  |
| DLA_LG4_001470 | LG4 |  |  |  |  |
| DLA_LG4_001480 | LG4 |  |  |  |  |
| DLA_LG4_001490 | LG4 |  |  |  |  |
| DLA_LG4_001510 | LG4 |  |  |  |  |
| DLA_LG4_001530 | LG4 |  |  |  |  |
| DLA_LG4_001540 | LG4 |  |  |  |  |
| DLA_LG4_001570 | LG4 |  |  |  |  |
| DLA_LG4_001580 | LG4 |  |  |  |  |
| DLA_LG4_001590 | LG4 |  |  |  |  |
| DLA_LG4_001595 | LG4 |  |  |  |  |
| DLA_LG4_001600 | LG4 |  |  |  |  |
| DLA_LG4_001610 | LG4 |  |  |  |  |
| DLA_LG4_001615 | LG4 |  |  |  |  |
| DLA_LG4_001620 | LG4 |  |  |  |  |
| DLA_LG4_001640 | LG4 |  |  |  |  |
| DLA_LG4_001660 | LG4 |  |  |  |  |
| DLA_LG4_001670 | LG4 |  |  |  |  |
| DLA_LG4_001685 | LG4 |  |  |  |  |
| DLA_LG4_001690 | LG4 |  |  |  |  |
| DLA_LG4_001700 | LG4 |  |  |  |  |
| DLA_LG4_001710 | LG4 |  |  |  |  |
| DLA_LG4_001720 | LG4 |  |  |  |  |
| DLA_LG4_001740 | LG4 |  |  |  |  |
| DLA_LG4_001750 | LG4 |  |  |  |  |
| DLA_LG4_001760 | LG4 |  |  |  |  |
| DLA_LG4_001770 | LG4 |  |  |  |  |
| DLA_LG4_001780 | LG4 |  |  |  |  |
| DLA_LG4_001790 | LG4 |  |  |  |  |
| DLA_LG4_001800 | LG4 |  |  |  |  |
| DLA_LG4_001810 | LG4 |  |  |  |  |
| DLA_LG4_001820 | LG4 |  |  |  |  |
| DLA_LG4_001830 | LG4 |  |  |  |  |
| DLA_LG4_001840 | LG4 |  |  |  |  |
| DLA_LG4_001850 | LG4 |  |  |  |  |
| DLA_LG4_001860 | LG4 |  |  |  |  |
| DLA_LG4_001870 | LG4 |  |  |  |  |
| DLA_LG4_001880 | LG4 |  |  |  |  |
| DLA_LG4_001890 | LG4 |  |  |  |  |
| DLA_LG4_001900 | LG4 |  |  |  |  |
| DLA_LG4_001910 | LG4 |  |  |  |  |
| DLA_LG4_001920 | LG4 |  |  |  |  |
| DLA_LG4_001930 | LG4 |  |  |  |  |
| DLA_LG4_001940 | LG4 |  |  |  |  |
| DLA_LG4_001950 | LG4 |  |  |  |  |
| DLA_LG4_001960 | LG4 |  |  |  |  |
| DLA_LG4_001970 | LG4 |  |  |  |  |
| DLA_LG4_001975 | LG4 |  |  |  |  |
| DLA_LG4_001990 | LG4 |  |  |  |  |
| DLA_LG4_002000 | LG4 |  |  |  |  |
| DLA_LG4_002010 | LG4 |  |  |  |  |
| DLA_LG4_002020 | LG4 |  |  |  |  |
| DLA_LG4_002030 | LG4 |  |  |  |  |
| DLA_LG4_002040 | LG4 |  |  |  |  |
| DLA_LG4_002070 | LG4 |  |  |  |  |
| DLA_LG4_002080 | LG4 |  |  |  |  |
| DLA_LG4_002090 | LG4 |  |  |  |  |
| DLA_LG4_002100 | LG4 |  |  |  |  |
| DLA_LG4_002120 | LG4 |  |  |  |  |
| DLA_LG4_002130 | LG4 |  |  |  |  |
| DLA_LG4_002140 | LG4 |  |  |  |  |
| DLA_LG4_002145 | LG4 |  |  |  |  |
| DLA_LG4_002150 | LG4 |  |  |  |  |
| DLA_LG4_002160 | LG4 |  |  |  |  |
| DLA_LG4_002170 | LG4 |  |  |  |  |
| DLA_LG4_002190 | LG4 |  |  |  |  |
| DLA_LG4_002200 | LG4 |  |  |  |  |
| DLA_LG4_002205 | LG4 |  |  |  |  |
| DLA_LG4_002210 | LG4 |  |  |  |  |
| DLA_LG4_002220 | LG4 |  |  |  |  |
| DLA_LG4_002230 | LG4 |  |  |  |  |
| DLA_LG4_002240 | LG4 |  |  |  |  |
| DLA_LG4_002250 | LG4 |  |  |  |  |
| DLA_LG4_002260 | LG4 |  |  |  |  |
| DLA_LG4_002280 | LG4 |  |  |  |  |
| DLA_LG4_002300 | LG4 |  |  |  |  |
| DLA_LG4_002310 | LG4 |  |  |  |  |
| DLA_LG4_002320 | LG4 |  |  |  |  |
| DLA_LG4_002330 | LG4 |  |  |  |  |
| DLA_LG4_002340 | LG4 |  |  |  |  |
| DLA_LG4_002350 | LG4 |  |  |  |  |
| DLA_LG4_002360 | LG4 |  |  |  |  |
| DLA_LG4_002370 | LG4 |  |  |  |  |
| DLA_LG4_002380 | LG4 |  |  |  |  |
| DLA_LG4_002390 | LG4 |  |  |  |  |
| DLA_LG4_002400 | LG4 |  |  |  |  |
| DLA_LG4_002410 | LG4 |  |  |  |  |
| DLA_LG4_002420 | LG4 |  |  |  |  |
| DLA_LG4_002430 | LG4 |  |  |  |  |
| DLA_LG4_002440 | LG4 |  |  |  |  |
| DLA_LG4_002450 | LG4 |  |  |  |  |
| DLA_LG4_002460 | LG4 |  |  |  |  |
| DLA_LG4_002470 | LG4 |  |  |  |  |
| DLA_LG4_002480 | LG4 |  |  |  |  |
| DLA_LG4_002500 | LG4 |  |  |  |  |
| DLA_LG4_002510 | LG4 |  |  |  |  |
| DLA_LG4_002520 | LG4 |  |  |  |  |
| DLA_LG4_002525 | LG4 |  |  |  |  |
| DLA_LG4_002540 | LG4 |  |  |  |  |
| DLA_LG4_002550 | LG4 |  |  |  |  |
| DLA_LG4_002580 | LG4 |  |  |  |  |
| DLA_LG4_002590 | LG4 |  |  |  |  |
| DLA_LG4_002600 | LG4 |  |  |  |  |
| DLA_LG4_002610 | LG4 |  |  |  |  |
| DLA_LG4_002630 | LG4 |  |  |  |  |
| DLA_LG4_002640 | LG4 |  |  |  |  |
| DLA_LG4_002650 | LG4 |  |  |  |  |
| DLA_LG4_002660 | LG4 |  |  |  |  |
| DLA_LG4_002670 | LG4 |  |  |  |  |
| DLA_LG4_002690 | LG4 |  |  |  |  |
| DLA_LG4_002700 | LG4 |  |  |  |  |
| DLA_LG4_002720 | LG4 |  |  |  |  |
| DLA_LG4_002730 | LG4 |  |  |  |  |
| DLA_LG4_002735 | LG4 |  |  |  |  |
| DLA_LG4_002740 | LG4 |  |  |  |  |
| DLA_LG4_002750 | LG4 |  |  |  |  |
| DLA_LG4_002760 | LG4 |  |  |  |  |
| DLA_LG4_002770 | LG4 |  |  |  |  |
| DLA_LG4_002780 | LG4 |  |  |  |  |
| DLA_LG4_002790 | LG4 |  |  |  |  |
| DLA_LG4_002810 | LG4 |  |  |  |  |
| DLA_LG4_002820 | LG4 |  |  |  |  |
| DLA_LG4_002830 | LG4 |  |  |  |  |
| DLA_LG4_002840 | LG4 |  |  |  |  |
| DLA_LG4_002845 | LG4 |  |  |  |  |
| DLA_LG4_002850 | LG4 |  |  |  |  |
| DLA_LG4_002860 | LG4 |  |  |  |  |
| DLA_LG4_002870 | LG4 |  |  |  |  |
| DLA_LG4_002880 | LG4 |  |  |  |  |
| DLA_LG4_002890 | LG4 |  |  |  |  |
| DLA_LG4_002895 | LG4 |  |  |  |  |
| DLA_LG4_002895_2 | LG4 |  |  |  |  |
| DLA_LG4_002900 | LG4 |  |  |  |  |
| DLA_LG4_002910 | LG4 |  |  |  |  |
| DLA_LG4_002920 | LG4 |  |  |  |  |
| DLA_LG4_002930 | LG4 |  |  |  |  |
| DLA_LG4_002935 | LG4 |  |  |  |  |
| DLA_LG4_002940 | LG4 |  |  |  |  |
| DLA_LG4_002970 | LG4 |  |  |  |  |
| DLA_LG4_002980 | LG4 |  |  |  |  |
| DLA_LG4_002990 | LG4 |  |  |  |  |
| DLA_LG4_003000 | LG4 |  |  |  |  |
| DLA_LG4_003020 | LG4 |  |  |  |  |
| DLA_LG4_003030 | LG4 |  |  |  |  |
| DLA_LG4_003035 | LG4 |  |  |  |  |
| DLA_LG4_003050 | LG4 |  |  |  |  |
| DLA_LG4_003070 | LG4 |  |  |  |  |
| DLA_LG4_003080 | LG4 |  |  |  |  |
| DLA_LG4_003090 | LG4 |  |  |  |  |
| DLA_LG4_003110 | LG4 |  |  |  |  |
| DLA_LG4_003120 | LG4 |  |  |  |  |
| DLA_LG4_003130 | LG4 |  |  |  |  |
| DLA_LG4_003140 | LG4 |  |  |  |  |
| DLA_LG4_003150 | LG4 |  |  |  |  |
| DLA_LG4_003160 | LG4 |  |  |  |  |
| DLA_LG4_003180 | LG4 |  |  |  |  |
| DLA_LG4_003185 | LG4 |  |  |  |  |
| DLA_LG4_003190 | LG4 |  |  |  |  |
| DLA_LG4_003210 | LG4 |  |  |  |  |
| DLA_LG4_003220 | LG4 |  |  |  |  |
| DLA_LG4_003230 | LG4 |  |  |  |  |
| DLA_LG4_003240 | LG4 |  |  |  |  |
| DLA_LG4_003250 | LG4 |  |  |  |  |
| DLA_LG4_003260 | LG4 |  |  |  |  |
| DLA_LG4_003270 | LG4 |  |  |  |  |
| DLA_LG4_003275 | LG4 |  |  |  |  |
| DLA_LG4_003280 | LG4 |  |  |  |  |
| DLA_LG4_003290 | LG4 |  |  |  |  |
| DLA_LG4_003300 | LG4 |  |  |  |  |
| DLA_LG4_003310 | LG4 |  |  |  |  |
| DLA_LG4_003320 | LG4 |  |  |  |  |
| DLA_LG4_003330 | LG4 |  |  |  |  |
| DLA_LG4_003340 | LG4 |  |  |  |  |
| DLA_LG4_003360 | LG4 |  |  |  |  |
| DLA_LG4_003370 | LG4 |  |  |  |  |
| DLA_LG4_003380 | LG4 |  |  |  |  |
| DLA_LG4_003390 | LG4 |  |  |  |  |
| DLA_LG4_003400 | LG4 |  |  |  |  |
| DLA_LG4_003410 | LG4 |  |  |  |  |
| DLA_LG4_003425 | LG4 |  |  |  |  |
| DLA_LG4_003430 | LG4 |  |  |  |  |
| DLA_LG4_003440 | LG4 |  |  |  |  |
| DLA_LG4_003450 | LG4 |  |  |  |  |
| DLA_LG4_003460 | LG4 |  |  |  |  |
| DLA_LG4_003485 | LG4 |  |  |  |  |
| DLA_LG4_003490 | LG4 |  |  |  |  |
| DLA_LG4_003500 | LG4 |  |  |  |  |
| DLA_LG4_003510 | LG4 |  |  |  |  |
| DLA_LG4_003520 | LG4 |  |  |  |  |
| DLA_LG4_003530 | LG4 |  |  |  |  |
| DLA_LG4_003540 | LG4 |  |  |  |  |
| DLA_LG4_003550 | LG4 |  |  |  |  |
| DLA_LG4_003560 | LG4 |  |  |  |  |
| DLA_LG4_003570 | LG4 |  |  |  |  |
| DLA_LG4_003580 | LG4 |  |  |  |  |
| DLA_LG4_003590 | LG4 |  |  |  |  |
| DLA_LG4_003600 | LG4 |  |  |  |  |
| DLA_LG4_003610 | LG4 |  |  |  |  |
| DLA_LG4_003620 | LG4 |  |  |  |  |
| DLA_LG4_003630 | LG4 |  |  |  |  |
| DLA_LG4_003650 | LG4 |  |  |  |  |
| DLA_LG4_003660 | LG4 |  |  |  |  |
| DLA_LG4_003670 | LG4 |  |  |  |  |
| DLA_LG4_003690 | LG4 |  |  |  |  |
| DLA_LG4_003700 | LG4 |  |  |  |  |
| DLA_LG4_003710 | LG4 |  |  |  |  |
| DLA_LG4_003720 | LG4 |  |  |  |  |
| DLA_LG4_003730 | LG4 |  |  |  |  |
| DLA_LG4_003740 | LG4 |  |  |  |  |
| DLA_LG4_003750 | LG4 |  |  |  |  |
| DLA_LG4_003760 | LG4 |  |  |  |  |
| DLA_LG4_003770 | LG4 |  |  |  |  |
| DLA_LG4_003780 | LG4 |  |  |  |  |
| DLA_LG4_003790 | LG4 |  |  |  |  |
| DLA_LG4_003820 | LG4 |  |  |  |  |
| DLA_LG4_003840 | LG4 |  |  |  |  |
| DLA_LG4_003850 | LG4 |  |  |  |  |
| DLA_LG4_003860 | LG4 |  |  |  |  |
| DLA_LG4_003870 | LG4 |  |  |  |  |
| DLA_LG4_003880 | LG4 |  |  |  |  |
| DLA_LG4_003890 | LG4 |  |  |  |  |
| DLA_LG4_003900 | LG4 |  |  |  |  |
| DLA_LG4_003910 | LG4 |  |  |  |  |
| DLA_LG4_003920 | LG4 |  |  |  |  |
| DLA_LG4_003930 | LG4 |  |  |  |  |
| DLA_LG4_003940 | LG4 |  |  |  |  |
| DLA_LG4_003950 | LG4 |  |  |  |  |
| DLA_LG4_003960 | LG4 |  |  |  |  |
| DLA_LG4_003980 | LG4 |  |  |  |  |
| DLA_LG4_003990 | LG4 |  |  |  |  |
| DLA_LG4_004000 | LG4 |  |  |  |  |
| DLA_LG4_004010 | LG4 |  |  |  |  |
| DLA_LG4_004020 | LG4 |  |  |  |  |
| DLA_LG4_004030 | LG4 |  |  |  |  |
| DLA_LG4_004040 | LG4 |  |  |  |  |
| DLA_LG4_004050 | LG4 |  |  |  |  |
| DLA_LG4_004060 | LG4 |  |  |  |  |
| DLA_LG4_004070 | LG4 |  |  |  |  |
| DLA_LG4_004080 | LG4 |  |  |  |  |
| DLA_LG4_004090 | LG4 |  |  |  |  |
| DLA_LG4_004100 | LG4 |  |  |  |  |
| DLA_LG4_004110 | LG4 |  |  |  |  |
| DLA_LG4_004120 | LG4 |  |  |  |  |
| DLA_LG4_004130 | LG4 |  |  |  |  |
| DLA_LG4_004140 | LG4 |  |  |  |  |
| DLA_LG4_004150 | LG4 |  |  |  |  |
| DLA_LG4_004170 | LG4 |  |  |  |  |
| DLA_LG4_004175 | LG4 |  |  |  |  |
| DLA_LG4_004180 | LG4 |  |  |  |  |
| DLA_LG4_004190 | LG4 |  |  |  |  |
| DLA_LG4_004200 | LG4 |  |  |  |  |
| DLA_LG4_004210 | LG4 |  |  |  |  |
| DLA_LG4_004220 | LG4 |  |  |  |  |
| DLA_LG4_004230 | LG4 |  |  |  |  |
| DLA_LG4_004240 | LG4 |  |  |  |  |
| DLA_LG4_004250 | LG4 |  |  |  |  |
| DLA_LG4_004260 | LG4 |  |  |  |  |
| DLA_LG4_004270 | LG4 |  |  |  |  |
| DLA_LG4_004280 | LG4 |  |  |  |  |
| DLA_LG4_004290 | LG4 |  |  |  |  |
| DLA_LG4_004300 | LG4 |  |  |  |  |
| DLA_LG4_004310 | LG4 |  |  |  |  |
| DLA_LG4_004330 | LG4 |  |  |  |  |
| DLA_LG4_004340 | LG4 |  |  |  |  |
| DLA_LG4_004360 | LG4 |  |  |  |  |
| DLA_LG4_004370 | LG4 |  |  |  |  |
| DLA_LG4_004380 | LG4 |  |  |  |  |
| DLA_LG4_004390 | LG4 |  |  |  |  |
| DLA_LG4_004400 | LG4 |  |  |  |  |
| DLA_LG4_004420 | LG4 |  |  |  |  |
| DLA_LG4_004430 | LG4 |  |  |  |  |
| DLA_LG4_004460 | LG4 |  |  |  |  |
| DLA_LG4_004480 | LG4 |  |  |  |  |
| DLA_LG4_004490 | LG4 |  |  |  |  |
| DLA_LG4_004510 | LG4 |  |  |  |  |
| DLA_LG4_004520 | LG4 |  |  |  |  |
| DLA_LG4_004530 | LG4 |  |  |  |  |
| DLA_LG4_004550 | LG4 |  |  |  |  |
| DLA_LG4_004560 | LG4 |  |  |  |  |
| DLA_LG4_004570 | LG4 |  |  |  |  |
| DLA_LG4_004580 | LG4 |  |  |  |  |
| DLA_LG4_004590 | LG4 |  |  |  |  |
| DLA_LG4_004600 | LG4 |  |  |  |  |
| DLA_LG4_004610 | LG4 |  |  |  |  |
| DLA_LG4_004620 | LG4 |  |  |  |  |
| DLA_LG4_004660 | LG4 |  |  |  |  |
| DLA_LG4_004670 | LG4 |  |  |  |  |
| DLA_LG4_004680 | LG4 |  |  |  |  |
| DLA_LG4_004690 | LG4 |  |  |  |  |
| DLA_LG4_004700 | LG4 |  |  |  |  |
| DLA_LG4_004705 | LG4 |  |  |  |  |
| DLA_LG4_004710 | LG4 |  |  |  |  |
| DLA_LG4_004720 | LG4 |  |  |  |  |
| DLA_LG4_004740 | LG4 |  |  |  |  |
| DLA_LG4_004750 | LG4 |  |  |  |  |
| DLA_LG4_004770 | LG4 |  |  |  |  |
| DLA_LG4_004800 | LG4 |  |  |  |  |
| DLA_LG4_004810 | LG4 |  |  |  |  |
| DLA_LG4_004820 | LG4 |  |  |  |  |
| DLA_LG4_004830 | LG4 |  |  |  |  |
| DLA_LG4_004840 | LG4 |  |  |  |  |
| DLA_LG4_004850 | LG4 |  |  |  |  |
| DLA_LG4_004860 | LG4 |  |  |  |  |
| DLA_LG4_004870 | LG4 |  |  |  |  |
| DLA_LG4_004880 | LG4 |  |  |  |  |
| DLA_LG4_004890 | LG4 |  |  |  |  |
| DLA_LG4_004900 | LG4 |  |  |  |  |
| DLA_LG4_004910 | LG4 |  |  |  |  |
| DLA_LG4_004920 | LG4 |  |  |  |  |
| DLA_LG4_004930 | LG4 |  |  |  |  |
| DLA_LG4_004940 | LG4 |  |  |  |  |
| DLA_LG4_004950 | LG4 |  |  |  |  |
| DLA_LG4_004960 | LG4 |  |  |  |  |
| DLA_LG4_004980 | LG4 |  |  |  |  |
| DLA_LG4_004990 | LG4 |  |  |  |  |
| DLA_LG4_005010 | LG4 |  |  |  |  |
| DLA_LG4_005020 | LG4 |  |  |  |  |
| DLA_LG4_005030 | LG4 |  |  |  |  |
| DLA_LG4_005050 | LG4 |  |  |  |  |
| DLA_LG4_005060 | LG4 |  |  |  |  |
| DLA_LG4_005070 | LG4 |  |  |  |  |
| DLA_LG4_005080 | LG4 |  |  |  |  |
| DLA_LG4_005090 | LG4 |  |  |  |  |
| DLA_LG4_005100 | LG4 |  |  |  |  |
| DLA_LG4_005110 | LG4 |  |  |  |  |
| DLA_LG4_005130 | LG4 |  |  |  |  |
| DLA_LG4_005140 | LG4 |  |  |  |  |
| DLA_LG4_005150 | LG4 |  |  |  |  |
| DLA_LG4_005170 | LG4 |  |  |  |  |
| DLA_LG4_005180 | LG4 |  |  |  |  |
| DLA_LG4_005190 | LG4 |  |  |  |  |
| DLA_LG4_005200 | LG4 |  |  |  |  |
| DLA_LG4_005210 | LG4 |  |  |  |  |
| DLA_LG4_005220 | LG4 |  |  |  |  |
| DLA_LG4_005230 | LG4 |  |  |  |  |
| DLA_LG4_005240 | LG4 |  |  |  |  |
| DLA_LG4_005250 | LG4 |  |  |  |  |
| DLA_LG4_005260 | LG4 |  |  |  |  |
| DLA_LG4_005270 | LG4 |  |  |  |  |
| DLA_LG4_005300 | LG4 |  |  |  |  |
| DLA_LG4_005310 | LG4 |  |  |  |  |
| DLA_LG4_005325 | LG4 |  |  |  |  |
| DLA_LG4_005330 | LG4 |  |  |  |  |
| DLA_LG4_005340 | LG4 |  |  |  |  |
| DLA_LG4_005350 | LG4 |  |  |  |  |
| DLA_LG4_005355 | LG4 |  |  |  |  |
| DLA_LG4_005360 | LG4 |  |  |  |  |
| DLA_LG4_005380 | LG4 |  |  |  |  |
| DLA_LG4_005400 | LG4 |  |  |  |  |
| DLA_LG4_005410 | LG4 |  |  |  |  |
| DLA_LG4_005420 | LG4 |  |  |  |  |
| DLA_LG4_005430 | LG4 |  |  |  |  |
| DLA_LG4_005440 | LG4 |  |  |  |  |
| DLA_LG4_005460 | LG4 |  |  |  |  |
| DLA_LG4_005470 | LG4 |  |  |  |  |
| DLA_LG4_005480 | LG4 |  |  |  |  |
| DLA_LG4_005490 | LG4 |  |  |  |  |
| DLA_LG4_005500 | LG4 |  |  |  |  |
| DLA_LG4_005510 | LG4 |  |  |  |  |
| DLA_LG4_005530 | LG4 |  |  |  |  |
| DLA_LG4_005550 | LG4 |  |  |  |  |
| DLA_LG4_005560 | LG4 |  |  |  |  |
| DLA_LG4_005570 | LG4 |  |  |  |  |
| DLA_LG4_005580 | LG4 |  |  |  |  |
| DLA_LG4_005590 | LG4 |  |  |  |  |
| DLA_LG4_005600 | LG4 |  |  |  |  |
| DLA_LG4_005610 | LG4 |  |  |  |  |
| DLA_LG4_005620 | LG4 |  |  |  |  |
| DLA_LG4_005630 | LG4 |  |  |  |  |
| DLA_LG4_005640 | LG4 |  |  |  |  |
| DLA_LG4_005650 | LG4 |  |  |  |  |
| DLA_LG4_005670 | LG4 |  |  |  |  |
| DLA_LG4_005680 | LG4 |  |  |  |  |
| DLA_LG4_005690 | LG4 |  |  |  |  |
| DLA_LG4_005700 | LG4 |  |  |  |  |
| DLA_LG4_005710 | LG4 |  |  |  |  |
| DLA_LG4_005715 | LG4 |  |  |  |  |
| DLA_LG4_005720 | LG4 |  |  |  |  |
| DLA_LG4_005730 | LG4 |  |  |  |  |
| DLA_LG4_005735 | LG4 |  |  |  |  |
| DLA_LG4_005740 | LG4 |  |  |  |  |
| DLA_LG4_005750 | LG4 |  |  |  |  |
| DLA_LG4_005760 | LG4 |  |  |  |  |
| DLA_LG4_005770 | LG4 |  |  |  |  |
| DLA_LG4_005780 | LG4 |  |  |  |  |
| DLA_LG4_005790 | LG4 |  |  |  |  |
| DLA_LG4_005800 | LG4 |  |  |  |  |
| DLA_LG4_005810 | LG4 |  |  |  |  |
| DLA_LG4_005820 | LG4 |  |  |  |  |
| DLA_LG4_005830 | LG4 |  |  |  |  |
| DLA_LG4_005850 | LG4 |  |  |  |  |
| DLA_LG4_005860 | LG4 |  |  |  |  |
| DLA_LG4_005865 | LG4 |  |  |  |  |
| DLA_LG4_005880 | LG4 |  |  |  |  |
| DLA_LG4_005890 | LG4 |  |  |  |  |
| DLA_LG4_005920 | LG4 |  |  |  |  |
| DLA_LG4_005930 | LG4 |  |  |  |  |
| DLA_LG4_005940 | LG4 |  |  |  |  |
| DLA_LG4_005950 | LG4 |  |  |  |  |
| DLA_LG4_005960 | LG4 |  |  |  |  |
| DLA_LG4_005970 | LG4 |  |  |  |  |
| DLA_LG4_005980 | LG4 |  |  |  |  |
| DLA_LG4_005990 | LG4 |  |  |  |  |
| DLA_LG4_006010 | LG4 |  |  |  |  |
| DLA_LG4_006030 | LG4 |  |  |  |  |
| DLA_LG4_006040 | LG4 |  |  |  |  |
| DLA_LG4_006060 | LG4 |  |  |  |  |
| DLA_LG4_006070 | LG4 |  |  |  |  |
| DLA_LG4_006080 | LG4 |  |  |  |  |
| DLA_LG4_006090 | LG4 |  |  |  |  |
| DLA_LG4_006100 | LG4 |  |  |  |  |
| DLA_LG4_006105 | LG4 |  |  |  |  |
| DLA_LG4_006110 | LG4 |  |  |  |  |
| DLA_LG4_006120 | LG4 |  |  |  |  |
| DLA_LG4_006140 | LG4 |  |  |  |  |
| DLA_LG4_006150 | LG4 |  |  |  |  |
| DLA_LG4_006160 | LG4 |  |  |  |  |
| DLA_LG4_006170 | LG4 |  |  |  |  |
| DLA_LG4_006180 | LG4 |  |  |  |  |
| DLA_LG4_006190 | LG4 |  |  |  |  |
| DLA_LG4_006200 | LG4 |  |  |  |  |
| DLA_LG4_006210 | LG4 |  |  |  |  |
| DLA_LG4_006215 | LG4 |  |  |  |  |
| DLA_LG4_006220 | LG4 |  |  |  |  |
| DLA_LG4_006230 | LG4 |  |  |  |  |
| DLA_LG4_006240 | LG4 |  |  |  |  |
| DLA_LG4_006250 | LG4 |  |  |  |  |
| DLA_LG4_006260 | LG4 |  |  |  |  |
| DLA_LG4_006280 | LG4 |  |  |  |  |
| DLA_LG4_006290 | LG4 |  |  |  |  |
| DLA_LG4_006300 | LG4 |  |  |  |  |
| DLA_LG4_006310 | LG4 |  |  |  |  |
| DLA_LG4_006320 | LG4 |  |  |  |  |
| DLA_LG4_006330 | LG4 |  |  |  |  |
| DLA_LG4_006360 | LG4 |  |  |  |  |
| DLA_LG4_006370 | LG4 |  |  |  |  |
| DLA_LG4_006380 | LG4 |  |  |  |  |
| DLA_LG4_006390 | LG4 |  |  |  |  |
| DLA_LG4_006400 | LG4 |  |  |  |  |
| DLA_LG4_006410 | LG4 |  |  |  |  |
| DLA_LG4_006420 | LG4 |  |  |  |  |
| DLA_LG4_006430 | LG4 |  |  |  |  |
| DLA_LG4_006440 | LG4 |  |  |  |  |
| DLA_LG4_006460 | LG4 |  |  |  |  |
| DLA_LG4_006470 | LG4 |  |  |  |  |
| DLA_LG4_006480 | LG4 |  |  |  |  |
| DLA_LG4_006490 | LG4 |  |  |  |  |
| DLA_LG4_006500 | LG4 |  |  |  |  |
| DLA_LG4_006510 | LG4 |  |  |  |  |
| DLA_LG4_006520 | LG4 |  |  |  |  |
| DLA_LG4_006530 | LG4 |  |  |  |  |
| DLA_LG4_006550 | LG4 |  |  |  |  |
| DLA_LG4_006560 | LG4 |  |  |  |  |
| DLA_LG4_006580 | LG4 |  |  |  |  |
| DLA_LG4_006590 | LG4 |  |  |  |  |
| DLA_LG4_006600 | LG4 |  |  |  |  |
| DLA_LG4_006605 | LG4 |  |  |  |  |
| DLA_LG4_006605_2 | LG4 |  |  |  |  |
| DLA_LG4_006610 | LG4 |  |  |  |  |
| DLA_LG4_006620 | LG4 |  |  |  |  |
| DLA_LG4_006630 | LG4 |  |  |  |  |
| DLA_LG4_006640 | LG4 |  |  |  |  |
| DLA_LG4_006650 | LG4 |  |  |  |  |
| DLA_LG4_006660 | LG4 |  |  |  |  |
| DLA_LG4_006670 | LG4 |  |  |  |  |
| DLA_LG4_006680 | LG4 |  |  |  |  |
| DLA_LG4_006690 | LG4 |  |  |  |  |
| DLA_LG4_006700 | LG4 |  |  |  |  |
| DLA_LG4_006710 | LG4 |  |  |  |  |
| DLA_LG4_006730 | LG4 |  |  |  |  |
| DLA_LG4_006740 | LG4 |  |  |  |  |
| DLA_LG4_006750 | LG4 |  |  |  |  |
| DLA_LG4_006760 | LG4 |  |  |  |  |
| DLA_LG4_006770 | LG4 |  |  |  |  |
| DLA_LG4_006790 | LG4 |  |  |  |  |
| DLA_LG4_006800 | LG4 |  |  |  |  |
| DLA_LG4_006810 | LG4 |  |  |  |  |
| DLA_LG4_006830 | LG4 |  |  |  |  |
| DLA_LG4_006840 | LG4 |  |  |  |  |
| DLA_LG4_006850 | LG4 |  |  |  |  |
| DLA_LG4_006860 | LG4 |  |  |  |  |
| DLA_LG4_006870 | LG4 |  |  |  |  |
| DLA_LG4_006880 | LG4 |  |  |  |  |
| DLA_LG4_006890 | LG4 |  |  |  |  |
| DLA_LG4_006900 | LG4 |  |  |  |  |
| DLA_LG4_006910 | LG4 |  |  |  |  |
| DLA_LG4_006920 | LG4 |  |  |  |  |
| DLA_LG4_006930 | LG4 |  |  |  |  |
| DLA_LG4_006945 | LG4 |  |  |  |  |
| DLA_LG4_006960 | LG4 |  |  |  |  |
| DLA_LG4_006970 | LG4 |  |  |  |  |
| DLA_LG4_006980 | LG4 |  |  |  |  |
| DLA_LG4_006990 | LG4 |  |  |  |  |
| DLA_LG4_007000 | LG4 |  |  |  |  |
| DLA_LG4_007010 | LG4 |  |  |  |  |
| DLA_LG4_007030 | LG4 |  |  |  |  |
| DLA_LG4_007035 | LG4 |  |  |  |  |
| DLA_LG4_007060 | LG4 |  |  |  |  |
| DLA_LG4_007070 | LG4 |  |  |  |  |
| DLA_LG4_007080 | LG4 |  |  |  |  |
| DLA_LG4_007090 | LG4 |  |  |  |  |
| DLA_LG4_007100 | LG4 |  |  |  |  |
| DLA_LG4_007110 | LG4 |  |  |  |  |
| DLA_LG4_007120 | LG4 |  |  |  |  |
| DLA_LG4_007140 | LG4 |  |  |  |  |
| DLA_LG4_007150 | LG4 |  |  |  |  |
| DLA_LG4_007160 | LG4 |  |  |  |  |
| DLA_LG4_007170 | LG4 |  |  |  |  |
| DLA_LG4_007180 | LG4 |  |  |  |  |
| DLA_LG4_007190 | LG4 |  |  |  |  |
| DLA_LG4_007230 | LG4 |  |  |  |  |
| DLA_LG4_007240 | LG4 |  |  |  |  |
| DLA_LG4_007260 | LG4 |  |  |  |  |
| DLA_LG4_007270 | LG4 |  |  |  |  |
| DLA_LG4_007280 | LG4 |  |  |  |  |
| DLA_LG4_007290 | LG4 |  |  |  |  |
| DLA_LG4_007300 | LG4 |  |  |  |  |
| DLA_LG4_007310 | LG4 |  |  |  |  |
| DLA_LG4_007330 | LG4 |  |  |  |  |
| DLA_LG4_007335 | LG4 |  |  |  |  |
| DLA_LG4_007340 | LG4 |  |  |  |  |
| DLA_LG4_007360 | LG4 |  |  |  |  |
| DLA_LG4_007380 | LG4 |  |  |  |  |
| DLA_LG4_007390 | LG4 |  |  |  |  |
| DLA_LG4_007400 | LG4 |  |  |  |  |
| DLA_LG4_007410 | LG4 |  |  |  |  |
| DLA_LG4_007420 | LG4 |  |  |  |  |
| DLA_LG4_007430 | LG4 |  |  |  |  |
| DLA_LG4_007460 | LG4 |  |  |  |  |
| DLA_LG4_007470 | LG4 |  |  |  |  |
| DLA_LG4_007490 | LG4 |  |  |  |  |
| DLA_LG4_007500 | LG4 |  |  |  |  |
| DLA_LG4_007510 | LG4 |  |  |  |  |
| DLA_LG4_007520 | LG4 |  |  |  |  |
| DLA_LG4_007530 | LG4 |  |  |  |  |
| DLA_LG4_007540 | LG4 |  |  |  |  |
| DLA_LG4_007550 | LG4 |  |  |  |  |
| DLA_LG4_007560 | LG4 |  |  |  |  |
| DLA_LG4_007570 | LG4 |  |  |  |  |
| DLA_LG4_007580 | LG4 |  |  |  |  |
| DLA_LG4_007590 | LG4 |  |  |  |  |
| DLA_LG4_007600 | LG4 |  |  |  |  |
| DLA_LG4_007610 | LG4 |  |  |  |  |
| DLA_LG4_007640 | LG4 |  |  |  |  |
| DLA_LG4_007660 | LG4 |  |  |  |  |
| DLA_LG4_007670 | LG4 |  |  |  |  |
| DLA_LG4_007680 | LG4 |  |  |  |  |
| DLA_LG4_007700 | LG4 |  |  |  |  |
| DLA_LG4_007710 | LG4 |  |  |  |  |
| DLA_LG4_007730 | LG4 |  |  |  |  |
| DLA_LG4_007740 | LG4 |  |  |  |  |
| DLA_LG4_007750 | LG4 |  |  |  |  |
| DLA_LG4_007780 | LG4 |  |  |  |  |
| DLA_LG4_007790 | LG4 |  |  |  |  |
| DLA_LG4_007800 | LG4 |  |  |  |  |
| DLA_LG4_007810 | LG4 |  |  |  |  |
| DLA_LG4_007820 | LG4 |  |  |  |  |
| DLA_LG4_007830 | LG4 |  |  |  |  |
| DLA_LG4_007840 | LG4 |  |  |  |  |
| DLA_LG4_007850 | LG4 |  |  |  |  |
| DLA_LG4_007890 | LG4 |  |  |  |  |
| DLA_LG4_007910 | LG4 |  |  |  |  |
| DLA_LG4_007920 | LG4 |  |  |  |  |
| DLA_LG4_007930 | LG4 |  |  |  |  |
| DLA_LG4_007950 | LG4 |  |  |  |  |
| DLA_LG4_007960 | LG4 |  |  |  |  |
| DLA_LG4_007970 | LG4 |  |  |  |  |
| DLA_LG4_007980 | LG4 |  |  |  |  |
| DLA_LG4_007990 | LG4 |  |  |  |  |
| DLA_LG4_008010 | LG4 |  |  |  |  |
| DLA_LG4_008020 | LG4 |  |  |  |  |
| DLA_LG4_008030 | LG4 |  |  |  |  |
| DLA_LG4_008050 | LG4 |  |  |  |  |
| DLA_LG4_008060 | LG4 |  |  |  |  |
| DLA_LG4_008070 | LG4 |  |  |  |  |
| DLA_LG4_008080 | LG4 |  |  |  |  |
| DLA_LG4_008090 | LG4 |  |  |  |  |
| DLA_LG4_008100 | LG4 |  |  |  |  |
| DLA_LG4_008110 | LG4 |  |  |  |  |
| DLA_LG4_008120 | LG4 |  |  |  |  |
| DLA_LG4_008130 | LG4 |  |  |  |  |
| DLA_LG4_008150 | LG4 |  |  |  |  |
| DLA_LG4_008160 | LG4 |  |  |  |  |
| DLA_LG4_008165 | LG4 |  |  |  |  |
| DLA_LG4_008180 | LG4 |  |  |  |  |
| DLA_LG4_008200 | LG4 |  |  |  |  |
| DLA_LG4_008210 | LG4 |  |  |  |  |
| DLA_LG4_008220 | LG4 |  |  |  |  |
| DLA_LG4_008240 | LG4 |  |  |  |  |
| DLA_LG4_008250 | LG4 |  |  |  |  |
| DLA_LG4_008270 | LG4 |  |  |  |  |
| DLA_LG4_008280 | LG4 |  |  |  |  |
| DLA_LG4_008290 | LG4 |  |  |  |  |
| DLA_LG4_008310 | LG4 |  |  |  |  |
| DLA_LG4_008320 | LG4 |  |  |  |  |
| DLA_LG4_008330 | LG4 |  |  |  |  |
| DLA_LG4_008350 | LG4 |  |  |  |  |
| DLA_LG4_008355 | LG4 |  |  |  |  |
| DLA_LG4_008360 | LG4 |  |  |  |  |
| DLA_LG4_008370 | LG4 |  |  |  |  |
| DLA_LG4_008380 | LG4 |  |  |  |  |
| DLA_LG4_008390 | LG4 |  |  |  |  |
| DLA_LG4_008400 | LG4 |  |  |  |  |
| DLA_LG4_008410 | LG4 |  |  |  |  |
| DLA_LG4_008420 | LG4 |  |  |  |  |
| DLA_LG4_008430 | LG4 |  |  |  |  |
| DLA_LG4_008440 | LG4 |  |  |  |  |
| DLA_LG4_008460 | LG4 |  |  |  |  |
| DLA_LG4_008470 | LG4 |  |  |  |  |
| DLA_LG4_008480 | LG4 |  |  |  |  |
| DLA_LG4_008490 | LG4 |  |  |  |  |
| DLA_LG4_008510 | LG4 |  |  |  |  |
| DLA_LG4_008520 | LG4 |  |  |  |  |
| DLA_LG4_008530 | LG4 |  |  |  |  |
| DLA_LG4_008545 | LG4 |  |  |  |  |
| DLA_LG4_008550 | LG4 |  |  |  |  |
| DLA_LG4_008560 | LG4 |  |  |  |  |
| DLA_LG4_008570 | LG4 |  |  |  |  |
| DLA_LG4_008580 | LG4 |  |  |  |  |
| DLA_LG4_008600 | LG4 |  |  |  |  |
| DLA_LG4_008605 | LG4 |  |  |  |  |
| DLA_LG4_008610 | LG4 |  |  |  |  |
| DLA_LG4_008620 | LG4 |  |  |  |  |
| DLA_LG4_008630 | LG4 |  |  |  |  |
| DLA_LG4_008635_2 | LG4 |  |  |  |  |
| DLA_LG4_008665 | LG4 |  |  |  |  |
| DLA_LG4_008670 | LG4 |  |  |  |  |
| DLA_LG4_008680 | LG4 |  |  |  |  |
| DLA_LG4_008700 | LG4 |  |  |  |  |
| DLA_LG4_008715 | LG4 |  |  |  |  |
| DLA_LG4_008720 | LG4 |  |  |  |  |
| DLA_LG4_008725 | LG4 |  |  |  |  |
| DLA_LG4_008725_2 | LG4 |  |  |  |  |
| DLA_LG4_008725_3 | LG4 |  |  |  |  |
| DLA_LG4_008725_4 | LG4 |  |  |  |  |
| DLA_LG4_008740 | LG4 |  |  |  |  |
| DLA_LG4_008750 | LG4 |  |  |  |  |
| DLA_LG4_008760 | LG4 |  |  |  |  |
| DLA_LG4_008770 | LG4 |  |  |  |  |
| DLA_LG4_008790 | LG4 |  |  |  |  |
| DLA_LG4_008810 | LG4 |  |  |  |  |
| DLA_LG4_008820 | LG4 |  |  |  |  |
| DLA_LG5_006680 | LG5 |  |  |  |  |
| DLA_LG5_000700 | LG5 |  |  |  |  |
| DLA_LG5_001170 | LG5 |  |  |  |  |
| DLA_LG5_001970 | LG5 |  |  |  |  |
| DLA_LG5_002210 | LG5 |  |  |  |  |
| DLA_LG5_003430 | LG5 |  |  |  |  |
| DLA_LG5_003630 | LG5 |  |  |  |  |
| DLA_LG5_004680 | LG5 |  |  |  |  |
| DLA_LG5_005120 | LG5 |  |  |  |  |
| DLA_LG5_006280 | LG5 |  |  |  |  |
| DLA_LG5_006890 | LG5 |  |  |  |  |
| DLA_LG5_007620 | LG5 |  |  |  |  |
| DLA_LG5_008350 | LG5 |  |  |  |  |
| DLA_LG5_008500 | LG5 |  |  |  |  |
| DLA_LG5_000030 | LG5 |  |  |  |  |
| DLA_LG5_000090 | LG5 |  |  |  |  |
| DLA_LG5_000200 | LG5 |  |  |  |  |
| DLA_LG5_000280 | LG5 |  |  |  |  |
| DLA_LG5_000310 | LG5 |  |  |  |  |
| DLA_LG5_000340 | LG5 |  |  |  |  |
| DLA_LG5_000460 | LG5 |  |  |  |  |
| DLA_LG5_000600 | LG5 |  |  |  |  |
| DLA_LG5_000890 | LG5 |  |  |  |  |
| DLA_LG5_000930 | LG5 |  |  |  |  |
| DLA_LG5_001015 | LG5 |  |  |  |  |
| DLA_LG5_001180 | LG5 |  |  |  |  |
| DLA_LG5_001220 | LG5 |  |  |  |  |
| DLA_LG5_001260 | LG5 |  |  |  |  |
| DLA_LG5_001315 | LG5 |  |  |  |  |
| DLA_LG5_001480 | LG5 |  |  |  |  |
| DLA_LG5_001510 | LG5 |  |  |  |  |
| DLA_LG5_001590 | LG5 |  |  |  |  |
| DLA_LG5_001810 | LG5 |  |  |  |  |
| DLA_LG5_001860 | LG5 |  |  |  |  |
| DLA_LG5_001990 | LG5 |  |  |  |  |
| DLA_LG5_002010 | LG5 |  |  |  |  |
| DLA_LG5_002580 | LG5 |  |  |  |  |
| DLA_LG5_002650 | LG5 |  |  |  |  |
| DLA_LG5_002750 | LG5 |  |  |  |  |
| DLA_LG5_003090 | LG5 |  |  |  |  |
| DLA_LG5_003210 | LG5 |  |  |  |  |
| DLA_LG5_003340 | LG5 |  |  |  |  |
| DLA_LG5_003370 | LG5 |  |  |  |  |
| DLA_LG5_003500 | LG5 |  |  |  |  |
| DLA_LG5_003550 | LG5 |  |  |  |  |
| DLA_LG5_003770 | LG5 |  |  |  |  |
| DLA_LG5_003900 | LG5 |  |  |  |  |
| DLA_LG5_004050 | LG5 |  |  |  |  |
| DLA_LG5_004180 | LG5 |  |  |  |  |
| DLA_LG5_004200 | LG5 |  |  |  |  |
| DLA_LG5_004220 | LG5 |  |  |  |  |
| DLA_LG5_004350 | LG5 |  |  |  |  |
| DLA_LG5_004370 | LG5 |  |  |  |  |
| DLA_LG5_004450 | LG5 |  |  |  |  |
| DLA_LG5_004560 | LG5 |  |  |  |  |
| DLA_LG5_004780 | LG5 |  |  |  |  |
| DLA_LG5_004790 | LG5 |  |  |  |  |
| DLA_LG5_004900 | LG5 |  |  |  |  |
| DLA_LG5_005010 | LG5 |  |  |  |  |
| DLA_LG5_005070 | LG5 |  |  |  |  |
| DLA_LG5_005100 | LG5 |  |  |  |  |
| DLA_LG5_005190 | LG5 |  |  |  |  |
| DLA_LG5_005230 | LG5 |  |  |  |  |
| DLA_LG5_005530 | LG5 |  |  |  |  |
| DLA_LG5_005830 | LG5 |  |  |  |  |
| DLA_LG5_006030 | LG5 |  |  |  |  |
| DLA_LG5_006050 | LG5 |  |  |  |  |
| DLA_LG5_006120 | LG5 |  |  |  |  |
| DLA_LG5_006170 | LG5 |  |  |  |  |
| DLA_LG5_006320 | LG5 |  |  |  |  |
| DLA_LG5_006410 | LG5 |  |  |  |  |
| DLA_LG5_006480 | LG5 |  |  |  |  |
| DLA_LG5_006510 | LG5 |  |  |  |  |
| DLA_LG5_006560 | LG5 |  |  |  |  |
| DLA_LG5_006650 | LG5 |  |  |  |  |
| DLA_LG5_006710 | LG5 |  |  |  |  |
| DLA_LG5_006740 | LG5 |  |  |  |  |
| DLA_LG5_006780 | LG5 |  |  |  |  |
| DLA_LG5_006950 | LG5 |  |  |  |  |
| DLA_LG5_007090 | LG5 |  |  |  |  |
| DLA_LG5_007130 | LG5 |  |  |  |  |
| DLA_LG5_007180 | LG5 |  |  |  |  |
| DLA_LG5_007665 | LG5 |  |  |  |  |
| DLA_LG5_007670 | LG5 |  |  |  |  |
| DLA_LG5_007760 | LG5 |  |  |  |  |
| DLA_LG5_007815_2 | LG5 |  |  |  |  |
| DLA_LG5_007890 | LG5 |  |  |  |  |
| DLA_LG5_007970 | LG5 |  |  |  |  |
| DLA_LG5_008210 | LG5 |  |  |  |  |
| DLA_LG5_008300 | LG5 |  |  |  |  |
| DLA_LG5_008320 | LG5 |  |  |  |  |
| DLA_LG5_008390 | LG5 |  |  |  |  |
| DLA_LG5_008410 | LG5 |  |  |  |  |
| DLA_LG5_008445 | LG5 |  |  |  |  |
| DLA_LG5_000010 | LG5 |  |  |  |  |
| DLA_LG5_000020 | LG5 |  |  |  |  |
| DLA_LG5_000035 | LG5 |  |  |  |  |
| DLA_LG5_000040 | LG5 |  |  |  |  |
| DLA_LG5_000050 | LG5 |  |  |  |  |
| DLA_LG5_000060 | LG5 |  |  |  |  |
| DLA_LG5_000070 | LG5 |  |  |  |  |
| DLA_LG5_000080 | LG5 |  |  |  |  |
| DLA_LG5_000100 | LG5 |  |  |  |  |
| DLA_LG5_000110 | LG5 |  |  |  |  |
| DLA_LG5_000120 | LG5 |  |  |  |  |
| DLA_LG5_000130 | LG5 |  |  |  |  |
| DLA_LG5_000140 | LG5 |  |  |  |  |
| DLA_LG5_000150 | LG5 |  |  |  |  |
| DLA_LG5_000160 | LG5 |  |  |  |  |
| DLA_LG5_000180 | LG5 |  |  |  |  |
| DLA_LG5_000190 | LG5 |  |  |  |  |
| DLA_LG5_000210 | LG5 |  |  |  |  |
| DLA_LG5_000220 | LG5 |  |  |  |  |
| DLA_LG5_000230 | LG5 |  |  |  |  |
| DLA_LG5_000240 | LG5 |  |  |  |  |
| DLA_LG5_000245 | LG5 |  |  |  |  |
| DLA_LG5_000250 | LG5 |  |  |  |  |
| DLA_LG5_000260 | LG5 |  |  |  |  |
| DLA_LG5_000270 | LG5 |  |  |  |  |
| DLA_LG5_000290 | LG5 |  |  |  |  |
| DLA_LG5_000300 | LG5 |  |  |  |  |
| DLA_LG5_000320 | LG5 |  |  |  |  |
| DLA_LG5_000330 | LG5 |  |  |  |  |
| DLA_LG5_000350 | LG5 |  |  |  |  |
| DLA_LG5_000360 | LG5 |  |  |  |  |
| DLA_LG5_000370 | LG5 |  |  |  |  |
| DLA_LG5_000380 | LG5 |  |  |  |  |
| DLA_LG5_000400 | LG5 |  |  |  |  |
| DLA_LG5_000410 | LG5 |  |  |  |  |
| DLA_LG5_000420 | LG5 |  |  |  |  |
| DLA_LG5_000440 | LG5 |  |  |  |  |
| DLA_LG5_000445 | LG5 |  |  |  |  |
| DLA_LG5_000450 | LG5 |  |  |  |  |
| DLA_LG5_000480 | LG5 |  |  |  |  |
| DLA_LG5_000490 | LG5 |  |  |  |  |
| DLA_LG5_000500 | LG5 |  |  |  |  |
| DLA_LG5_000510 | LG5 |  |  |  |  |
| DLA_LG5_000520 | LG5 |  |  |  |  |
| DLA_LG5_000530 | LG5 |  |  |  |  |
| DLA_LG5_000540 | LG5 |  |  |  |  |
| DLA_LG5_000550 | LG5 |  |  |  |  |
| DLA_LG5_000560 | LG5 |  |  |  |  |
| DLA_LG5_000570 | LG5 |  |  |  |  |
| DLA_LG5_000580 | LG5 |  |  |  |  |
| DLA_LG5_000590 | LG5 |  |  |  |  |
| DLA_LG5_000610 | LG5 |  |  |  |  |
| DLA_LG5_000620 | LG5 |  |  |  |  |
| DLA_LG5_000630 | LG5 |  |  |  |  |
| DLA_LG5_000650 | LG5 |  |  |  |  |
| DLA_LG5_000660 | LG5 |  |  |  |  |
| DLA_LG5_000670 | LG5 |  |  |  |  |
| DLA_LG5_000680 | LG5 |  |  |  |  |
| DLA_LG5_000690 | LG5 |  |  |  |  |
| DLA_LG5_000695 | LG5 |  |  |  |  |
| DLA_LG5_000710 | LG5 |  |  |  |  |
| DLA_LG5_000730 | LG5 |  |  |  |  |
| DLA_LG5_000750 | LG5 |  |  |  |  |
| DLA_LG5_000760 | LG5 |  |  |  |  |
| DLA_LG5_000770 | LG5 |  |  |  |  |
| DLA_LG5_000780 | LG5 |  |  |  |  |
| DLA_LG5_000790 | LG5 |  |  |  |  |
| DLA_LG5_000800 | LG5 |  |  |  |  |
| DLA_LG5_000810 | LG5 |  |  |  |  |
| DLA_LG5_000820 | LG5 |  |  |  |  |
| DLA_LG5_000830 | LG5 |  |  |  |  |
| DLA_LG5_000850 | LG5 |  |  |  |  |
| DLA_LG5_000860 | LG5 |  |  |  |  |
| DLA_LG5_000870 | LG5 |  |  |  |  |
| DLA_LG5_000880 | LG5 |  |  |  |  |
| DLA_LG5_000900 | LG5 |  |  |  |  |
| DLA_LG5_000910 | LG5 |  |  |  |  |
| DLA_LG5_000915 | LG5 |  |  |  |  |
| DLA_LG5_000920 | LG5 |  |  |  |  |
| DLA_LG5_000940 | LG5 |  |  |  |  |
| DLA_LG5_000950 | LG5 |  |  |  |  |
| DLA_LG5_000960 | LG5 |  |  |  |  |
| DLA_LG5_000970 | LG5 |  |  |  |  |
| DLA_LG5_000980 | LG5 |  |  |  |  |
| DLA_LG5_000990 | LG5 |  |  |  |  |
| DLA_LG5_001020 | LG5 |  |  |  |  |
| DLA_LG5_001030 | LG5 |  |  |  |  |
| DLA_LG5_001040 | LG5 |  |  |  |  |
| DLA_LG5_001050 | LG5 |  |  |  |  |
| DLA_LG5_001060 | LG5 |  |  |  |  |
| DLA_LG5_001070 | LG5 |  |  |  |  |
| DLA_LG5_001080 | LG5 |  |  |  |  |
| DLA_LG5_001090 | LG5 |  |  |  |  |
| DLA_LG5_001110 | LG5 |  |  |  |  |
| DLA_LG5_001120 | LG5 |  |  |  |  |
| DLA_LG5_001125 | LG5 |  |  |  |  |
| DLA_LG5_001130 | LG5 |  |  |  |  |
| DLA_LG5_001140 | LG5 |  |  |  |  |
| DLA_LG5_001150 | LG5 |  |  |  |  |
| DLA_LG5_001155 | LG5 |  |  |  |  |
| DLA_LG5_001160 | LG5 |  |  |  |  |
| DLA_LG5_001190 | LG5 |  |  |  |  |
| DLA_LG5_001200 | LG5 |  |  |  |  |
| DLA_LG5_001210 | LG5 |  |  |  |  |
| DLA_LG5_001230 | LG5 |  |  |  |  |
| DLA_LG5_001250 | LG5 |  |  |  |  |
| DLA_LG5_001255 | LG5 |  |  |  |  |
| DLA_LG5_001270 | LG5 |  |  |  |  |
| DLA_LG5_001290 | LG5 |  |  |  |  |
| DLA_LG5_001300 | LG5 |  |  |  |  |
| DLA_LG5_001310 | LG5 |  |  |  |  |
| DLA_LG5_001320 | LG5 |  |  |  |  |
| DLA_LG5_001325 | LG5 |  |  |  |  |
| DLA_LG5_001360 | LG5 |  |  |  |  |
| DLA_LG5_001370 | LG5 |  |  |  |  |
| DLA_LG5_001380 | LG5 |  |  |  |  |
| DLA_LG5_001390 | LG5 |  |  |  |  |
| DLA_LG5_001400 | LG5 |  |  |  |  |
| DLA_LG5_001410 | LG5 |  |  |  |  |
| DLA_LG5_001430 | LG5 |  |  |  |  |
| DLA_LG5_001440 | LG5 |  |  |  |  |
| DLA_LG5_001450 | LG5 |  |  |  |  |
| DLA_LG5_001460 | LG5 |  |  |  |  |
| DLA_LG5_001470 | LG5 |  |  |  |  |
| DLA_LG5_001490 | LG5 |  |  |  |  |
| DLA_LG5_001500 | LG5 |  |  |  |  |
| DLA_LG5_001520 | LG5 |  |  |  |  |
| DLA_LG5_001530 | LG5 |  |  |  |  |
| DLA_LG5_001540 | LG5 |  |  |  |  |
| DLA_LG5_001550 | LG5 |  |  |  |  |
| DLA_LG5_001570 | LG5 |  |  |  |  |
| DLA_LG5_001580 | LG5 |  |  |  |  |
| DLA_LG5_001600 | LG5 |  |  |  |  |
| DLA_LG5_001610 | LG5 |  |  |  |  |
| DLA_LG5_001620 | LG5 |  |  |  |  |
| DLA_LG5_001630 | LG5 |  |  |  |  |
| DLA_LG5_001640 | LG5 |  |  |  |  |
| DLA_LG5_001650 | LG5 |  |  |  |  |
| DLA_LG5_001660 | LG5 |  |  |  |  |
| DLA_LG5_001670 | LG5 |  |  |  |  |
| DLA_LG5_001680 | LG5 |  |  |  |  |
| DLA_LG5_001690 | LG5 |  |  |  |  |
| DLA_LG5_001700 | LG5 |  |  |  |  |
| DLA_LG5_001710 | LG5 |  |  |  |  |
| DLA_LG5_001720 | LG5 |  |  |  |  |
| DLA_LG5_001725 | LG5 |  |  |  |  |
| DLA_LG5_001730 | LG5 |  |  |  |  |
| DLA_LG5_001740 | LG5 |  |  |  |  |
| DLA_LG5_001750 | LG5 |  |  |  |  |
| DLA_LG5_001760 | LG5 |  |  |  |  |
| DLA_LG5_001770 | LG5 |  |  |  |  |
| DLA_LG5_001780 | LG5 |  |  |  |  |
| DLA_LG5_001790 | LG5 |  |  |  |  |
| DLA_LG5_001820 | LG5 |  |  |  |  |
| DLA_LG5_001825 | LG5 |  |  |  |  |
| DLA_LG5_001830 | LG5 |  |  |  |  |
| DLA_LG5_001840 | LG5 |  |  |  |  |
| DLA_LG5_001850 | LG5 |  |  |  |  |
| DLA_LG5_001870 | LG5 |  |  |  |  |
| DLA_LG5_001880 | LG5 |  |  |  |  |
| DLA_LG5_001890 | LG5 |  |  |  |  |
| DLA_LG5_001910 | LG5 |  |  |  |  |
| DLA_LG5_001920 | LG5 |  |  |  |  |
| DLA_LG5_001940 | LG5 |  |  |  |  |
| DLA_LG5_001950 | LG5 |  |  |  |  |
| DLA_LG5_001960 | LG5 |  |  |  |  |
| DLA_LG5_001965 | LG5 |  |  |  |  |
| DLA_LG5_001980 | LG5 |  |  |  |  |
| DLA_LG5_002000 | LG5 |  |  |  |  |
| DLA_LG5_002015 | LG5 |  |  |  |  |
| DLA_LG5_002020 | LG5 |  |  |  |  |
| DLA_LG5_002030 | LG5 |  |  |  |  |
| DLA_LG5_002040 | LG5 |  |  |  |  |
| DLA_LG5_002050 | LG5 |  |  |  |  |
| DLA_LG5_002060 | LG5 |  |  |  |  |
| DLA_LG5_002070 | LG5 |  |  |  |  |
| DLA_LG5_002080 | LG5 |  |  |  |  |
| DLA_LG5_002090 | LG5 |  |  |  |  |
| DLA_LG5_002100 | LG5 |  |  |  |  |
| DLA_LG5_002110 | LG5 |  |  |  |  |
| DLA_LG5_002115 | LG5 |  |  |  |  |
| DLA_LG5_002120 | LG5 |  |  |  |  |
| DLA_LG5_002130 | LG5 |  |  |  |  |
| DLA_LG5_002140 | LG5 |  |  |  |  |
| DLA_LG5_002150 | LG5 |  |  |  |  |
| DLA_LG5_002160 | LG5 |  |  |  |  |
| DLA_LG5_002170 | LG5 |  |  |  |  |
| DLA_LG5_002180 | LG5 |  |  |  |  |
| DLA_LG5_002190 | LG5 |  |  |  |  |
| DLA_LG5_002200 | LG5 |  |  |  |  |
| DLA_LG5_002220 | LG5 |  |  |  |  |
| DLA_LG5_002230 | LG5 |  |  |  |  |
| DLA_LG5_002250 | LG5 |  |  |  |  |
| DLA_LG5_002260 | LG5 |  |  |  |  |
| DLA_LG5_002270 | LG5 |  |  |  |  |
| DLA_LG5_002280 | LG5 |  |  |  |  |
| DLA_LG5_002290 | LG5 |  |  |  |  |
| DLA_LG5_002310 | LG5 |  |  |  |  |
| DLA_LG5_002320 | LG5 |  |  |  |  |
| DLA_LG5_002330 | LG5 |  |  |  |  |
| DLA_LG5_002340 | LG5 |  |  |  |  |
| DLA_LG5_002350 | LG5 |  |  |  |  |
| DLA_LG5_002360 | LG5 |  |  |  |  |
| DLA_LG5_002370 | LG5 |  |  |  |  |
| DLA_LG5_002380 | LG5 |  |  |  |  |
| DLA_LG5_002390 | LG5 |  |  |  |  |
| DLA_LG5_002400 | LG5 |  |  |  |  |
| DLA_LG5_002410 | LG5 |  |  |  |  |
| DLA_LG5_002420 | LG5 |  |  |  |  |
| DLA_LG5_002430 | LG5 |  |  |  |  |
| DLA_LG5_002450 | LG5 |  |  |  |  |
| DLA_LG5_002460 | LG5 |  |  |  |  |
| DLA_LG5_002470 | LG5 |  |  |  |  |
| DLA_LG5_002480 | LG5 |  |  |  |  |
| DLA_LG5_002490 | LG5 |  |  |  |  |
| DLA_LG5_002500 | LG5 |  |  |  |  |
| DLA_LG5_002510 | LG5 |  |  |  |  |
| DLA_LG5_002520 | LG5 |  |  |  |  |
| DLA_LG5_002530 | LG5 |  |  |  |  |
| DLA_LG5_002540 | LG5 |  |  |  |  |
| DLA_LG5_002550 | LG5 |  |  |  |  |
| DLA_LG5_002560 | LG5 |  |  |  |  |
| DLA_LG5_002570 | LG5 |  |  |  |  |
| DLA_LG5_002575 | LG5 |  |  |  |  |
| DLA_LG5_002590 | LG5 |  |  |  |  |
| DLA_LG5_002600 | LG5 |  |  |  |  |
| DLA_LG5_002610 | LG5 |  |  |  |  |
| DLA_LG5_002620 | LG5 |  |  |  |  |
| DLA_LG5_002630 | LG5 |  |  |  |  |
| DLA_LG5_002640 | LG5 |  |  |  |  |
| DLA_LG5_002660 | LG5 |  |  |  |  |
| DLA_LG5_002680 | LG5 |  |  |  |  |
| DLA_LG5_002690 | LG5 |  |  |  |  |
| DLA_LG5_002700 | LG5 |  |  |  |  |
| DLA_LG5_002710 | LG5 |  |  |  |  |
| DLA_LG5_002720 | LG5 |  |  |  |  |
| DLA_LG5_002730 | LG5 |  |  |  |  |
| DLA_LG5_002740 | LG5 |  |  |  |  |
| DLA_LG5_002760 | LG5 |  |  |  |  |
| DLA_LG5_002770 | LG5 |  |  |  |  |
| DLA_LG5_002790 | LG5 |  |  |  |  |
| DLA_LG5_002800 | LG5 |  |  |  |  |
| DLA_LG5_002810 | LG5 |  |  |  |  |
| DLA_LG5_002820 | LG5 |  |  |  |  |
| DLA_LG5_002835_2 | LG5 |  |  |  |  |
| DLA_LG5_002835_3 | LG5 |  |  |  |  |
| DLA_LG5_002840 | LG5 |  |  |  |  |
| DLA_LG5_002850 | LG5 |  |  |  |  |
| DLA_LG5_002860 | LG5 |  |  |  |  |
| DLA_LG5_002870 | LG5 |  |  |  |  |
| DLA_LG5_002880 | LG5 |  |  |  |  |
| DLA_LG5_002890 | LG5 |  |  |  |  |
| DLA_LG5_002910 | LG5 |  |  |  |  |
| DLA_LG5_002920 | LG5 |  |  |  |  |
| DLA_LG5_002930 | LG5 |  |  |  |  |
| DLA_LG5_002940 | LG5 |  |  |  |  |
| DLA_LG5_002950 | LG5 |  |  |  |  |
| DLA_LG5_002970 | LG5 |  |  |  |  |
| DLA_LG5_002980 | LG5 |  |  |  |  |
| DLA_LG5_002990 | LG5 |  |  |  |  |
| DLA_LG5_003000 | LG5 |  |  |  |  |
| DLA_LG5_003010 | LG5 |  |  |  |  |
| DLA_LG5_003020 | LG5 |  |  |  |  |
| DLA_LG5_003030 | LG5 |  |  |  |  |
| DLA_LG5_003040 | LG5 |  |  |  |  |
| DLA_LG5_003050 | LG5 |  |  |  |  |
| DLA_LG5_003060 | LG5 |  |  |  |  |
| DLA_LG5_003080 | LG5 |  |  |  |  |
| DLA_LG5_003085 | LG5 |  |  |  |  |
| DLA_LG5_003085_2 | LG5 |  |  |  |  |
| DLA_LG5_003100 | LG5 |  |  |  |  |
| DLA_LG5_003110 | LG5 |  |  |  |  |
| DLA_LG5_003120 | LG5 |  |  |  |  |
| DLA_LG5_003130 | LG5 |  |  |  |  |
| DLA_LG5_003140 | LG5 |  |  |  |  |
| DLA_LG5_003150 | LG5 |  |  |  |  |
| DLA_LG5_003160 | LG5 |  |  |  |  |
| DLA_LG5_003170 | LG5 |  |  |  |  |
| DLA_LG5_003190 | LG5 |  |  |  |  |
| DLA_LG5_003200 | LG5 |  |  |  |  |
| DLA_LG5_003220 | LG5 |  |  |  |  |
| DLA_LG5_003230 | LG5 |  |  |  |  |
| DLA_LG5_003240 | LG5 |  |  |  |  |
| DLA_LG5_003250 | LG5 |  |  |  |  |
| DLA_LG5_003260 | LG5 |  |  |  |  |
| DLA_LG5_003270 | LG5 |  |  |  |  |
| DLA_LG5_003280 | LG5 |  |  |  |  |
| DLA_LG5_003290 | LG5 |  |  |  |  |
| DLA_LG5_003300 | LG5 |  |  |  |  |
| DLA_LG5_003310 | LG5 |  |  |  |  |
| DLA_LG5_003320 | LG5 |  |  |  |  |
| DLA_LG5_003330 | LG5 |  |  |  |  |
| DLA_LG5_003350 | LG5 |  |  |  |  |
| DLA_LG5_003360 | LG5 |  |  |  |  |
| DLA_LG5_003375 | LG5 |  |  |  |  |
| DLA_LG5_003390 | LG5 |  |  |  |  |
| DLA_LG5_003395 | LG5 |  |  |  |  |
| DLA_LG5_003400 | LG5 |  |  |  |  |
| DLA_LG5_003410 | LG5 |  |  |  |  |
| DLA_LG5_003420 | LG5 |  |  |  |  |
| DLA_LG5_003440 | LG5 |  |  |  |  |
| DLA_LG5_003450 | LG5 |  |  |  |  |
| DLA_LG5_003460 | LG5 |  |  |  |  |
| DLA_LG5_003470 | LG5 |  |  |  |  |
| DLA_LG5_003480 | LG5 |  |  |  |  |
| DLA_LG5_003490 | LG5 |  |  |  |  |
| DLA_LG5_003495 | LG5 |  |  |  |  |
| DLA_LG5_003510 | LG5 |  |  |  |  |
| DLA_LG5_003520 | LG5 |  |  |  |  |
| DLA_LG5_003530 | LG5 |  |  |  |  |
| DLA_LG5_003560 | LG5 |  |  |  |  |
| DLA_LG5_003570 | LG5 |  |  |  |  |
| DLA_LG5_003580 | LG5 |  |  |  |  |
| DLA_LG5_003590 | LG5 |  |  |  |  |
| DLA_LG5_003600 | LG5 |  |  |  |  |
| DLA_LG5_003610 | LG5 |  |  |  |  |
| DLA_LG5_003620 | LG5 |  |  |  |  |
| DLA_LG5_003640 | LG5 |  |  |  |  |
| DLA_LG5_003650 | LG5 |  |  |  |  |
| DLA_LG5_003660 | LG5 |  |  |  |  |
| DLA_LG5_003670 | LG5 |  |  |  |  |
| DLA_LG5_003680 | LG5 |  |  |  |  |
| DLA_LG5_003690 | LG5 |  |  |  |  |
| DLA_LG5_003700 | LG5 |  |  |  |  |
| DLA_LG5_003710 | LG5 |  |  |  |  |
| DLA_LG5_003720 | LG5 |  |  |  |  |
| DLA_LG5_003730 | LG5 |  |  |  |  |
| DLA_LG5_003740 | LG5 |  |  |  |  |
| DLA_LG5_003750 | LG5 |  |  |  |  |
| DLA_LG5_003760 | LG5 |  |  |  |  |
| DLA_LG5_003780 | LG5 |  |  |  |  |
| DLA_LG5_003820 | LG5 |  |  |  |  |
| DLA_LG5_003830 | LG5 |  |  |  |  |
| DLA_LG5_003835 | LG5 |  |  |  |  |
| DLA_LG5_003840 | LG5 |  |  |  |  |
| DLA_LG5_003850 | LG5 |  |  |  |  |
| DLA_LG5_003860 | LG5 |  |  |  |  |
| DLA_LG5_003865 | LG5 |  |  |  |  |
| DLA_LG5_003870 | LG5 |  |  |  |  |
| DLA_LG5_003875 | LG5 |  |  |  |  |
| DLA_LG5_003880 | LG5 |  |  |  |  |
| DLA_LG5_003890 | LG5 |  |  |  |  |
| DLA_LG5_003910 | LG5 |  |  |  |  |
| DLA_LG5_003920 | LG5 |  |  |  |  |
| DLA_LG5_003930 | LG5 |  |  |  |  |
| DLA_LG5_003940 | LG5 |  |  |  |  |
| DLA_LG5_003950 | LG5 |  |  |  |  |
| DLA_LG5_003960 | LG5 |  |  |  |  |
| DLA_LG5_003970 | LG5 |  |  |  |  |
| DLA_LG5_003980 | LG5 |  |  |  |  |
| DLA_LG5_003990 | LG5 |  |  |  |  |
| DLA_LG5_004000 | LG5 |  |  |  |  |
| DLA_LG5_004010 | LG5 |  |  |  |  |
| DLA_LG5_004020 | LG5 |  |  |  |  |
| DLA_LG5_004030 | LG5 |  |  |  |  |
| DLA_LG5_004040 | LG5 |  |  |  |  |
| DLA_LG5_004060 | LG5 |  |  |  |  |
| DLA_LG5_004070 | LG5 |  |  |  |  |
| DLA_LG5_004080 | LG5 |  |  |  |  |
| DLA_LG5_004090 | LG5 |  |  |  |  |
| DLA_LG5_004100 | LG5 |  |  |  |  |
| DLA_LG5_004110 | LG5 |  |  |  |  |
| DLA_LG5_004120 | LG5 |  |  |  |  |
| DLA_LG5_004130 | LG5 |  |  |  |  |
| DLA_LG5_004140 | LG5 |  |  |  |  |
| DLA_LG5_004150 | LG5 |  |  |  |  |
| DLA_LG5_004160 | LG5 |  |  |  |  |
| DLA_LG5_004170 | LG5 |  |  |  |  |
| DLA_LG5_004190 | LG5 |  |  |  |  |
| DLA_LG5_004210 | LG5 |  |  |  |  |
| DLA_LG5_004215 | LG5 |  |  |  |  |
| DLA_LG5_004230 | LG5 |  |  |  |  |
| DLA_LG5_004240 | LG5 |  |  |  |  |
| DLA_LG5_004250 | LG5 |  |  |  |  |
| DLA_LG5_004255 | LG5 |  |  |  |  |
| DLA_LG5_004260 | LG5 |  |  |  |  |
| DLA_LG5_004270 | LG5 |  |  |  |  |
| DLA_LG5_004280 | LG5 |  |  |  |  |
| DLA_LG5_004290 | LG5 |  |  |  |  |
| DLA_LG5_004300 | LG5 |  |  |  |  |
| DLA_LG5_004310 | LG5 |  |  |  |  |
| DLA_LG5_004320 | LG5 |  |  |  |  |
| DLA_LG5_004330 | LG5 |  |  |  |  |
| DLA_LG5_004340 | LG5 |  |  |  |  |
| DLA_LG5_004360 | LG5 |  |  |  |  |
| DLA_LG5_004380 | LG5 |  |  |  |  |
| DLA_LG5_004390 | LG5 |  |  |  |  |
| DLA_LG5_004400 | LG5 |  |  |  |  |
| DLA_LG5_004410 | LG5 |  |  |  |  |
| DLA_LG5_004420 | LG5 |  |  |  |  |
| DLA_LG5_004430 | LG5 |  |  |  |  |
| DLA_LG5_004440 | LG5 |  |  |  |  |
| DLA_LG5_004455 | LG5 |  |  |  |  |
| DLA_LG5_004460 | LG5 |  |  |  |  |
| DLA_LG5_004470 | LG5 |  |  |  |  |
| DLA_LG5_004480 | LG5 |  |  |  |  |
| DLA_LG5_004490 | LG5 |  |  |  |  |
| DLA_LG5_004500 | LG5 |  |  |  |  |
| DLA_LG5_004510 | LG5 |  |  |  |  |
| DLA_LG5_004520 | LG5 |  |  |  |  |
| DLA_LG5_004530 | LG5 |  |  |  |  |
| DLA_LG5_004535 | LG5 |  |  |  |  |
| DLA_LG5_004540 | LG5 |  |  |  |  |
| DLA_LG5_004550 | LG5 |  |  |  |  |
| DLA_LG5_004570 | LG5 |  |  |  |  |
| DLA_LG5_004580 | LG5 |  |  |  |  |
| DLA_LG5_004590 | LG5 |  |  |  |  |
| DLA_LG5_004600 | LG5 |  |  |  |  |
| DLA_LG5_004605 | LG5 |  |  |  |  |
| DLA_LG5_004620 | LG5 |  |  |  |  |
| DLA_LG5_004630 | LG5 |  |  |  |  |
| DLA_LG5_004640 | LG5 |  |  |  |  |
| DLA_LG5_004645 | LG5 |  |  |  |  |
| DLA_LG5_004650 | LG5 |  |  |  |  |
| DLA_LG5_004670 | LG5 |  |  |  |  |
| DLA_LG5_004690 | LG5 |  |  |  |  |
| DLA_LG5_004700 | LG5 |  |  |  |  |
| DLA_LG5_004710 | LG5 |  |  |  |  |
| DLA_LG5_004720 | LG5 |  |  |  |  |
| DLA_LG5_004730 | LG5 |  |  |  |  |
| DLA_LG5_004740 | LG5 |  |  |  |  |
| DLA_LG5_004760 | LG5 |  |  |  |  |
| DLA_LG5_004770 | LG5 |  |  |  |  |
| DLA_LG5_004800 | LG5 |  |  |  |  |
| DLA_LG5_004815 | LG5 |  |  |  |  |
| DLA_LG5_004820 | LG5 |  |  |  |  |
| DLA_LG5_004830 | LG5 |  |  |  |  |
| DLA_LG5_004850 | LG5 |  |  |  |  |
| DLA_LG5_004860 | LG5 |  |  |  |  |
| DLA_LG5_004870 | LG5 |  |  |  |  |
| DLA_LG5_004880 | LG5 |  |  |  |  |
| DLA_LG5_004890 | LG5 |  |  |  |  |
| DLA_LG5_004910 | LG5 |  |  |  |  |
| DLA_LG5_004920 | LG5 |  |  |  |  |
| DLA_LG5_004930 | LG5 |  |  |  |  |
| DLA_LG5_004940 | LG5 |  |  |  |  |
| DLA_LG5_004950 | LG5 |  |  |  |  |
| DLA_LG5_004960 | LG5 |  |  |  |  |
| DLA_LG5_004970 | LG5 |  |  |  |  |
| DLA_LG5_004980 | LG5 |  |  |  |  |
| DLA_LG5_004990 | LG5 |  |  |  |  |
| DLA_LG5_005000 | LG5 |  |  |  |  |
| DLA_LG5_005020 | LG5 |  |  |  |  |
| DLA_LG5_005030 | LG5 |  |  |  |  |
| DLA_LG5_005040 | LG5 |  |  |  |  |
| DLA_LG5_005050 | LG5 |  |  |  |  |
| DLA_LG5_005060 | LG5 |  |  |  |  |
| DLA_LG5_005080 | LG5 |  |  |  |  |
| DLA_LG5_005110 | LG5 |  |  |  |  |
| DLA_LG5_005130 | LG5 |  |  |  |  |
| DLA_LG5_005140 | LG5 |  |  |  |  |
| DLA_LG5_005150 | LG5 |  |  |  |  |
| DLA_LG5_005160 | LG5 |  |  |  |  |
| DLA_LG5_005170 | LG5 |  |  |  |  |
| DLA_LG5_005200 | LG5 |  |  |  |  |
| DLA_LG5_005210 | LG5 |  |  |  |  |
| DLA_LG5_005220 | LG5 |  |  |  |  |
| DLA_LG5_005240 | LG5 |  |  |  |  |
| DLA_LG5_005250 | LG5 |  |  |  |  |
| DLA_LG5_005260 | LG5 |  |  |  |  |
| DLA_LG5_005270 | LG5 |  |  |  |  |
| DLA_LG5_005280 | LG5 |  |  |  |  |
| DLA_LG5_005300 | LG5 |  |  |  |  |
| DLA_LG5_005310 | LG5 |  |  |  |  |
| DLA_LG5_005320 | LG5 |  |  |  |  |
| DLA_LG5_005330 | LG5 |  |  |  |  |
| DLA_LG5_005340 | LG5 |  |  |  |  |
| DLA_LG5_005350 | LG5 |  |  |  |  |
| DLA_LG5_005360 | LG5 |  |  |  |  |
| DLA_LG5_005370 | LG5 |  |  |  |  |
| DLA_LG5_005380 | LG5 |  |  |  |  |
| DLA_LG5_005390 | LG5 |  |  |  |  |
| DLA_LG5_005400 | LG5 |  |  |  |  |
| DLA_LG5_005410 | LG5 |  |  |  |  |
| DLA_LG5_005420 | LG5 |  |  |  |  |
| DLA_LG5_005430 | LG5 |  |  |  |  |
| DLA_LG5_005440 | LG5 |  |  |  |  |
| DLA_LG5_005450 | LG5 |  |  |  |  |
| DLA_LG5_005460 | LG5 |  |  |  |  |
| DLA_LG5_005470 | LG5 |  |  |  |  |
| DLA_LG5_005480 | LG5 |  |  |  |  |
| DLA_LG5_005490 | LG5 |  |  |  |  |
| DLA_LG5_005495 | LG5 |  |  |  |  |
| DLA_LG5_005500 | LG5 |  |  |  |  |
| DLA_LG5_005510 | LG5 |  |  |  |  |
| DLA_LG5_005520 | LG5 |  |  |  |  |
| DLA_LG5_005540 | LG5 |  |  |  |  |
| DLA_LG5_005550 | LG5 |  |  |  |  |
| DLA_LG5_005560 | LG5 |  |  |  |  |
| DLA_LG5_005570 | LG5 |  |  |  |  |
| DLA_LG5_005580 | LG5 |  |  |  |  |
| DLA_LG5_005590 | LG5 |  |  |  |  |
| DLA_LG5_005600 | LG5 |  |  |  |  |
| DLA_LG5_005610 | LG5 |  |  |  |  |
| DLA_LG5_005620 | LG5 |  |  |  |  |
| DLA_LG5_005630 | LG5 |  |  |  |  |
| DLA_LG5_005640 | LG5 |  |  |  |  |
| DLA_LG5_005650 | LG5 |  |  |  |  |
| DLA_LG5_005660 | LG5 |  |  |  |  |
| DLA_LG5_005670 | LG5 |  |  |  |  |
| DLA_LG5_005680 | LG5 |  |  |  |  |
| DLA_LG5_005690 | LG5 |  |  |  |  |
| DLA_LG5_005700 | LG5 |  |  |  |  |
| DLA_LG5_005710 | LG5 |  |  |  |  |
| DLA_LG5_005720 | LG5 |  |  |  |  |
| DLA_LG5_005730 | LG5 |  |  |  |  |
| DLA_LG5_005740 | LG5 |  |  |  |  |
| DLA_LG5_005750 | LG5 |  |  |  |  |
| DLA_LG5_005760 | LG5 |  |  |  |  |
| DLA_LG5_005790 | LG5 |  |  |  |  |
| DLA_LG5_005800 | LG5 |  |  |  |  |
| DLA_LG5_005810 | LG5 |  |  |  |  |
| DLA_LG5_005820 | LG5 |  |  |  |  |
| DLA_LG5_005840 | LG5 |  |  |  |  |
| DLA_LG5_005850 | LG5 |  |  |  |  |
| DLA_LG5_005860 | LG5 |  |  |  |  |
| DLA_LG5_005870 | LG5 |  |  |  |  |
| DLA_LG5_005880 | LG5 |  |  |  |  |
| DLA_LG5_005890 | LG5 |  |  |  |  |
| DLA_LG5_005900 | LG5 |  |  |  |  |
| DLA_LG5_005905 | LG5 |  |  |  |  |
| DLA_LG5_005910 | LG5 |  |  |  |  |
| DLA_LG5_005920 | LG5 |  |  |  |  |
| DLA_LG5_005930 | LG5 |  |  |  |  |
| DLA_LG5_005940 | LG5 |  |  |  |  |
| DLA_LG5_005950 | LG5 |  |  |  |  |
| DLA_LG5_005970 | LG5 |  |  |  |  |
| DLA_LG5_005980 | LG5 |  |  |  |  |
| DLA_LG5_005990 | LG5 |  |  |  |  |
| DLA_LG5_006000 | LG5 |  |  |  |  |
| DLA_LG5_006010 | LG5 |  |  |  |  |
| DLA_LG5_006020 | LG5 |  |  |  |  |
| DLA_LG5_006040 | LG5 |  |  |  |  |
| DLA_LG5_006070 | LG5 |  |  |  |  |
| DLA_LG5_006080 | LG5 |  |  |  |  |
| DLA_LG5_006090 | LG5 |  |  |  |  |
| DLA_LG5_006100 | LG5 |  |  |  |  |
| DLA_LG5_006110 | LG5 |  |  |  |  |
| DLA_LG5_006130 | LG5 |  |  |  |  |
| DLA_LG5_006140 | LG5 |  |  |  |  |
| DLA_LG5_006150 | LG5 |  |  |  |  |
| DLA_LG5_006180 | LG5 |  |  |  |  |
| DLA_LG5_006190 | LG5 |  |  |  |  |
| DLA_LG5_006200 | LG5 |  |  |  |  |
| DLA_LG5_006210 | LG5 |  |  |  |  |
| DLA_LG5_006220 | LG5 |  |  |  |  |
| DLA_LG5_006230 | LG5 |  |  |  |  |
| DLA_LG5_006250 | LG5 |  |  |  |  |
| DLA_LG5_006260 | LG5 |  |  |  |  |
| DLA_LG5_006270 | LG5 |  |  |  |  |
| DLA_LG5_006290 | LG5 |  |  |  |  |
| DLA_LG5_006300 | LG5 |  |  |  |  |
| DLA_LG5_006310 | LG5 |  |  |  |  |
| DLA_LG5_006330 | LG5 |  |  |  |  |
| DLA_LG5_006340 | LG5 |  |  |  |  |
| DLA_LG5_006350 | LG5 |  |  |  |  |
| DLA_LG5_006360 | LG5 |  |  |  |  |
| DLA_LG5_006370 | LG5 |  |  |  |  |
| DLA_LG5_006380 | LG5 |  |  |  |  |
| DLA_LG5_006390 | LG5 |  |  |  |  |
| DLA_LG5_006400 | LG5 |  |  |  |  |
| DLA_LG5_006420 | LG5 |  |  |  |  |
| DLA_LG5_006430 | LG5 |  |  |  |  |
| DLA_LG5_006440 | LG5 |  |  |  |  |
| DLA_LG5_006455 | LG5 |  |  |  |  |
| DLA_LG5_006460 | LG5 |  |  |  |  |
| DLA_LG5_006470 | LG5 |  |  |  |  |
| DLA_LG5_006490 | LG5 |  |  |  |  |
| DLA_LG5_006530 | LG5 |  |  |  |  |
| DLA_LG5_006535 | LG5 |  |  |  |  |
| DLA_LG5_006540 | LG5 |  |  |  |  |
| DLA_LG5_006550 | LG5 |  |  |  |  |
| DLA_LG5_006570 | LG5 |  |  |  |  |
| DLA_LG5_006590 | LG5 |  |  |  |  |
| DLA_LG5_006600 | LG5 |  |  |  |  |
| DLA_LG5_006610 | LG5 |  |  |  |  |
| DLA_LG5_006620 | LG5 |  |  |  |  |
| DLA_LG5_006640 | LG5 |  |  |  |  |
| DLA_LG5_006645 | LG5 |  |  |  |  |
| DLA_LG5_006660 | LG5 |  |  |  |  |
| DLA_LG5_006670 | LG5 |  |  |  |  |
| DLA_LG5_006690 | LG5 |  |  |  |  |
| DLA_LG5_006700 | LG5 |  |  |  |  |
| DLA_LG5_006720 | LG5 |  |  |  |  |
| DLA_LG5_006730 | LG5 |  |  |  |  |
| DLA_LG5_006750 | LG5 |  |  |  |  |
| DLA_LG5_006760 | LG5 |  |  |  |  |
| DLA_LG5_006770 | LG5 |  |  |  |  |
| DLA_LG5_006800 | LG5 |  |  |  |  |
| DLA_LG5_006810 | LG5 |  |  |  |  |
| DLA_LG5_006820 | LG5 |  |  |  |  |
| DLA_LG5_006830 | LG5 |  |  |  |  |
| DLA_LG5_006840 | LG5 |  |  |  |  |
| DLA_LG5_006850 | LG5 |  |  |  |  |
| DLA_LG5_006870 | LG5 |  |  |  |  |
| DLA_LG5_006880 | LG5 |  |  |  |  |
| DLA_LG5_006900 | LG5 |  |  |  |  |
| DLA_LG5_006910 | LG5 |  |  |  |  |
| DLA_LG5_006920 | LG5 |  |  |  |  |
| DLA_LG5_006930 | LG5 |  |  |  |  |
| DLA_LG5_006940 | LG5 |  |  |  |  |
| DLA_LG5_006960 | LG5 |  |  |  |  |
| DLA_LG5_006970 | LG5 |  |  |  |  |
| DLA_LG5_006980 | LG5 |  |  |  |  |
| DLA_LG5_006990 | LG5 |  |  |  |  |
| DLA_LG5_007000 | LG5 |  |  |  |  |
| DLA_LG5_007010 | LG5 |  |  |  |  |
| DLA_LG5_007015 | LG5 |  |  |  |  |
| DLA_LG5_007015_2 | LG5 |  |  |  |  |
| DLA_LG5_007020 | LG5 |  |  |  |  |
| DLA_LG5_007030 | LG5 |  |  |  |  |
| DLA_LG5_007040 | LG5 |  |  |  |  |
| DLA_LG5_007050 | LG5 |  |  |  |  |
| DLA_LG5_007060 | LG5 |  |  |  |  |
| DLA_LG5_007070 | LG5 |  |  |  |  |
| DLA_LG5_007080 | LG5 |  |  |  |  |
| DLA_LG5_007110 | LG5 |  |  |  |  |
| DLA_LG5_007115 | LG5 |  |  |  |  |
| DLA_LG5_007120 | LG5 |  |  |  |  |
| DLA_LG5_007140 | LG5 |  |  |  |  |
| DLA_LG5_007150 | LG5 |  |  |  |  |
| DLA_LG5_007160 | LG5 |  |  |  |  |
| DLA_LG5_007170 | LG5 |  |  |  |  |
| DLA_LG5_007190 | LG5 |  |  |  |  |
| DLA_LG5_007200 | LG5 |  |  |  |  |
| DLA_LG5_007210 | LG5 |  |  |  |  |
| DLA_LG5_007220 | LG5 |  |  |  |  |
| DLA_LG5_007230 | LG5 |  |  |  |  |
| DLA_LG5_007240 | LG5 |  |  |  |  |
| DLA_LG5_007250 | LG5 |  |  |  |  |
| DLA_LG5_007260 | LG5 |  |  |  |  |
| DLA_LG5_007270 | LG5 |  |  |  |  |
| DLA_LG5_007290 | LG5 |  |  |  |  |
| DLA_LG5_007300 | LG5 |  |  |  |  |
| DLA_LG5_007320 | LG5 |  |  |  |  |
| DLA_LG5_007330 | LG5 |  |  |  |  |
| DLA_LG5_007340 | LG5 |  |  |  |  |
| DLA_LG5_007350 | LG5 |  |  |  |  |
| DLA_LG5_007360 | LG5 |  |  |  |  |
| DLA_LG5_007370 | LG5 |  |  |  |  |
| DLA_LG5_007380 | LG5 |  |  |  |  |
| DLA_LG5_007390 | LG5 |  |  |  |  |
| DLA_LG5_007400 | LG5 |  |  |  |  |
| DLA_LG5_007410 | LG5 |  |  |  |  |
| DLA_LG5_007420 | LG5 |  |  |  |  |
| DLA_LG5_007430 | LG5 |  |  |  |  |
| DLA_LG5_007440 | LG5 |  |  |  |  |
| DLA_LG5_007450 | LG5 |  |  |  |  |
| DLA_LG5_007460 | LG5 |  |  |  |  |
| DLA_LG5_007470 | LG5 |  |  |  |  |
| DLA_LG5_007480 | LG5 |  |  |  |  |
| DLA_LG5_007500 | LG5 |  |  |  |  |
| DLA_LG5_007510 | LG5 |  |  |  |  |
| DLA_LG5_007520 | LG5 |  |  |  |  |
| DLA_LG5_007530 | LG5 |  |  |  |  |
| DLA_LG5_007535 | LG5 |  |  |  |  |
| DLA_LG5_007535_2 | LG5 |  |  |  |  |
| DLA_LG5_007540 | LG5 |  |  |  |  |
| DLA_LG5_007550 | LG5 |  |  |  |  |
| DLA_LG5_007560 | LG5 |  |  |  |  |
| DLA_LG5_007565 | LG5 |  |  |  |  |
| DLA_LG5_007565_2 | LG5 |  |  |  |  |
| DLA_LG5_007565_3 | LG5 |  |  |  |  |
| DLA_LG5_007570 | LG5 |  |  |  |  |
| DLA_LG5_007580 | LG5 |  |  |  |  |
| DLA_LG5_007585 | LG5 |  |  |  |  |
| DLA_LG5_007590 | LG5 |  |  |  |  |
| DLA_LG5_007610 | LG5 |  |  |  |  |
| DLA_LG5_007630 | LG5 |  |  |  |  |
| DLA_LG5_007640 | LG5 |  |  |  |  |
| DLA_LG5_007650 | LG5 |  |  |  |  |
| DLA_LG5_007660 | LG5 |  |  |  |  |
| DLA_LG5_007665_2 | LG5 |  |  |  |  |
| DLA_LG5_007680 | LG5 |  |  |  |  |
| DLA_LG5_007685 | LG5 |  |  |  |  |
| DLA_LG5_007685_2 | LG5 |  |  |  |  |
| DLA_LG5_007690 | LG5 |  |  |  |  |
| DLA_LG5_007700 | LG5 |  |  |  |  |
| DLA_LG5_007720 | LG5 |  |  |  |  |
| DLA_LG5_007730 | LG5 |  |  |  |  |
| DLA_LG5_007740 | LG5 |  |  |  |  |
| DLA_LG5_007750 | LG5 |  |  |  |  |
| DLA_LG5_007770 | LG5 |  |  |  |  |
| DLA_LG5_007780 | LG5 |  |  |  |  |
| DLA_LG5_007785 | LG5 |  |  |  |  |
| DLA_LG5_007790 | LG5 |  |  |  |  |
| DLA_LG5_007800 | LG5 |  |  |  |  |
| DLA_LG5_007810 | LG5 |  |  |  |  |
| DLA_LG5_007820 | LG5 |  |  |  |  |
| DLA_LG5_007830 | LG5 |  |  |  |  |
| DLA_LG5_007840 | LG5 |  |  |  |  |
| DLA_LG5_007850 | LG5 |  |  |  |  |
| DLA_LG5_007860 | LG5 |  |  |  |  |
| DLA_LG5_007870 | LG5 |  |  |  |  |
| DLA_LG5_007880 | LG5 |  |  |  |  |
| DLA_LG5_007910 | LG5 |  |  |  |  |
| DLA_LG5_007920 | LG5 |  |  |  |  |
| DLA_LG5_007940 | LG5 |  |  |  |  |
| DLA_LG5_007950 | LG5 |  |  |  |  |
| DLA_LG5_007960 | LG5 |  |  |  |  |
| DLA_LG5_007980 | LG5 |  |  |  |  |
| DLA_LG5_007990 | LG5 |  |  |  |  |
| DLA_LG5_008000 | LG5 |  |  |  |  |
| DLA_LG5_008005 | LG5 |  |  |  |  |
| DLA_LG5_008010 | LG5 |  |  |  |  |
| DLA_LG5_008020 | LG5 |  |  |  |  |
| DLA_LG5_008030 | LG5 |  |  |  |  |
| DLA_LG5_008040 | LG5 |  |  |  |  |
| DLA_LG5_008050 | LG5 |  |  |  |  |
| DLA_LG5_008060 | LG5 |  |  |  |  |
| DLA_LG5_008070 | LG5 |  |  |  |  |
| DLA_LG5_008080 | LG5 |  |  |  |  |
| DLA_LG5_008090 | LG5 |  |  |  |  |
| DLA_LG5_008100 | LG5 |  |  |  |  |
| DLA_LG5_008110 | LG5 |  |  |  |  |
| DLA_LG5_008120 | LG5 |  |  |  |  |
| DLA_LG5_008130 | LG5 |  |  |  |  |
| DLA_LG5_008140 | LG5 |  |  |  |  |
| DLA_LG5_008150 | LG5 |  |  |  |  |
| DLA_LG5_008160 | LG5 |  |  |  |  |
| DLA_LG5_008170 | LG5 |  |  |  |  |
| DLA_LG5_008180 | LG5 |  |  |  |  |
| DLA_LG5_008200 | LG5 |  |  |  |  |
| DLA_LG5_008220 | LG5 |  |  |  |  |
| DLA_LG5_008230 | LG5 |  |  |  |  |
| DLA_LG5_008240 | LG5 |  |  |  |  |
| DLA_LG5_008250 | LG5 |  |  |  |  |
| DLA_LG5_008260 | LG5 |  |  |  |  |
| DLA_LG5_008280 | LG5 |  |  |  |  |
| DLA_LG5_008290 | LG5 |  |  |  |  |
| DLA_LG5_008295 | LG5 |  |  |  |  |
| DLA_LG5_008310 | LG5 |  |  |  |  |
| DLA_LG5_008325 | LG5 |  |  |  |  |
| DLA_LG5_008330 | LG5 |  |  |  |  |
| DLA_LG5_008340 | LG5 |  |  |  |  |
| DLA_LG5_008360 | LG5 |  |  |  |  |
| DLA_LG5_008370 | LG5 |  |  |  |  |
| DLA_LG5_008400 | LG5 |  |  |  |  |
| DLA_LG5_008405 | LG5 |  |  |  |  |
| DLA_LG5_008420 | LG5 |  |  |  |  |
| DLA_LG5_008430 | LG5 |  |  |  |  |
| DLA_LG5_008440 | LG5 |  |  |  |  |
| DLA_LG5_008450 | LG5 |  |  |  |  |
| DLA_LG5_008460 | LG5 |  |  |  |  |
| DLA_LG5_008470 | LG5 |  |  |  |  |
| DLA_LG5_008480 | LG5 |  |  |  |  |
| DLA_LG5_008490 | LG5 |  |  |  |  |
| DLA_LG5_008495 | LG5 |  |  |  |  |
| DLA_LG5_008510 | LG5 |  |  |  |  |
| DLA_LG5_008520 | LG5 |  |  |  |  |
| DLA_LG5_008530 | LG5 |  |  |  |  |
| DLA_LG6_008505 | LG6 |  |  |  |  |
| DLA_LG6_000660 | LG6 |  |  |  |  |
| DLA_LG6_001195 | LG6 |  |  |  |  |
| DLA_LG6_002090 | LG6 |  |  |  |  |
| DLA_LG6_002990 | LG6 |  |  |  |  |
| DLA_LG6_003300 | LG6 |  |  |  |  |
| DLA_LG6_003700 | LG6 |  |  |  |  |
| DLA_LG6_004270 | LG6 |  |  |  |  |
| DLA_LG6_005940 | LG6 |  |  |  |  |
| DLA_LG6_006710 | LG6 |  |  |  |  |
| DLA_LG6_007380 | LG6 |  |  |  |  |
| DLA_LG6_008160 | LG6 |  |  |  |  |
| DLA_LG6_009190 | LG6 |  |  |  |  |
| DLA_LG6_000070 | LG6 |  |  |  |  |
| DLA_LG6_000100 | LG6 |  |  |  |  |
| DLA_LG6_000160 | LG6 |  |  |  |  |
| DLA_LG6_000235 | LG6 |  |  |  |  |
| DLA_LG6_000510 | LG6 |  |  |  |  |
| DLA_LG6_000750 | LG6 |  |  |  |  |
| DLA_LG6_000790 | LG6 |  |  |  |  |
| DLA_LG6_000830 | LG6 |  |  |  |  |
| DLA_LG6_001000 | LG6 |  |  |  |  |
| DLA_LG6_001020 | LG6 |  |  |  |  |
| DLA_LG6_001050 | LG6 |  |  |  |  |
| DLA_LG6_001130 | LG6 |  |  |  |  |
| DLA_LG6_001210 | LG6 |  |  |  |  |
| DLA_LG6_001270 | LG6 |  |  |  |  |
| DLA_LG6_001790 | LG6 |  |  |  |  |
| DLA_LG6_001830 | LG6 |  |  |  |  |
| DLA_LG6_001900 | LG6 |  |  |  |  |
| DLA_LG6_001980 | LG6 |  |  |  |  |
| DLA_LG6_002045 | LG6 |  |  |  |  |
| DLA_LG6_002180 | LG6 |  |  |  |  |
| DLA_LG6_002280 | LG6 |  |  |  |  |
| DLA_LG6_002320 | LG6 |  |  |  |  |
| DLA_LG6_002430 | LG6 |  |  |  |  |
| DLA_LG6_002540 | LG6 |  |  |  |  |
| DLA_LG6_002680 | LG6 |  |  |  |  |
| DLA_LG6_002770 | LG6 |  |  |  |  |
| DLA_LG6_002860 | LG6 |  |  |  |  |
| DLA_LG6_003150 | LG6 |  |  |  |  |
| DLA_LG6_003230 | LG6 |  |  |  |  |
| DLA_LG6_003260 | LG6 |  |  |  |  |
| DLA_LG6_003500 | LG6 |  |  |  |  |
| DLA_LG6_003610 | LG6 |  |  |  |  |
| DLA_LG6_003860 | LG6 |  |  |  |  |
| DLA_LG6_003950 | LG6 |  |  |  |  |
| DLA_LG6_004040 | LG6 |  |  |  |  |
| DLA_LG6_004480 | LG6 |  |  |  |  |
| DLA_LG6_004600 | LG6 |  |  |  |  |
| DLA_LG6_004700 | LG6 |  |  |  |  |
| DLA_LG6_004800 | LG6 |  |  |  |  |
| DLA_LG6_004910 | LG6 |  |  |  |  |
| DLA_LG6_005180 | LG6 |  |  |  |  |
| DLA_LG6_005210 | LG6 |  |  |  |  |
| DLA_LG6_005250 | LG6 |  |  |  |  |
| DLA_LG6_005390 | LG6 |  |  |  |  |
| DLA_LG6_005540 | LG6 |  |  |  |  |
| DLA_LG6_005760 | LG6 |  |  |  |  |
| DLA_LG6_005820 | LG6 |  |  |  |  |
| DLA_LG6_005910 | LG6 |  |  |  |  |
| DLA_LG6_005990 | LG6 |  |  |  |  |
| DLA_LG6_006110 | LG6 |  |  |  |  |
| DLA_LG6_006160 | LG6 |  |  |  |  |
| DLA_LG6_006210 | LG6 |  |  |  |  |
| DLA_LG6_006260 | LG6 |  |  |  |  |
| DLA_LG6_006290 | LG6 |  |  |  |  |
| DLA_LG6_006440 | LG6 |  |  |  |  |
| DLA_LG6_006510 | LG6 |  |  |  |  |
| DLA_LG6_006610 | LG6 |  |  |  |  |
| DLA_LG6_006870 | LG6 |  |  |  |  |
| DLA_LG6_006920 | LG6 |  |  |  |  |
| DLA_LG6_007040 | LG6 |  |  |  |  |
| DLA_LG6_007250 | LG6 |  |  |  |  |
| DLA_LG6_007270 | LG6 |  |  |  |  |
| DLA_LG6_007420 | LG6 |  |  |  |  |
| DLA_LG6_007440 | LG6 |  |  |  |  |
| DLA_LG6_007620 | LG6 |  |  |  |  |
| DLA_LG6_007710 | LG6 |  |  |  |  |
| DLA_LG6_007730 | LG6 |  |  |  |  |
| DLA_LG6_007760 | LG6 |  |  |  |  |
| DLA_LG6_007810 | LG6 |  |  |  |  |
| DLA_LG6_007840 | LG6 |  |  |  |  |
| DLA_LG6_007870 | LG6 |  |  |  |  |
| DLA_LG6_007960 | LG6 |  |  |  |  |
| DLA_LG6_008050 | LG6 |  |  |  |  |
| DLA_LG6_008110 | LG6 |  |  |  |  |
| DLA_LG6_008140 | LG6 |  |  |  |  |
| DLA_LG6_008300 | LG6 |  |  |  |  |
| DLA_LG6_008340 | LG6 |  |  |  |  |
| DLA_LG6_008440 | LG6 |  |  |  |  |
| DLA_LG6_008450 | LG6 |  |  |  |  |
| DLA_LG6_008560 | LG6 |  |  |  |  |
| DLA_LG6_008650 | LG6 |  |  |  |  |
| DLA_LG6_008700 | LG6 |  |  |  |  |
| DLA_LG6_008770 | LG6 |  |  |  |  |
| DLA_LG6_009050 | LG6 |  |  |  |  |
| DLA_LG6_009240 | LG6 |  |  |  |  |
| DLA_LG6_000010 | LG6 |  |  |  |  |
| DLA_LG6_000015 | LG6 |  |  |  |  |
| DLA_LG6_000020 | LG6 |  |  |  |  |
| DLA_LG6_000030 | LG6 |  |  |  |  |
| DLA_LG6_000040 | LG6 |  |  |  |  |
| DLA_LG6_000060 | LG6 |  |  |  |  |
| DLA_LG6_000080 | LG6 |  |  |  |  |
| DLA_LG6_000085 | LG6 |  |  |  |  |
| DLA_LG6_000085_2 | LG6 |  |  |  |  |
| DLA_LG6_000090 | LG6 |  |  |  |  |
| DLA_LG6_000110 | LG6 |  |  |  |  |
| DLA_LG6_000120 | LG6 |  |  |  |  |
| DLA_LG6_000130 | LG6 |  |  |  |  |
| DLA_LG6_000140 | LG6 |  |  |  |  |
| DLA_LG6_000150 | LG6 |  |  |  |  |
| DLA_LG6_000170 | LG6 |  |  |  |  |
| DLA_LG6_000180 | LG6 |  |  |  |  |
| DLA_LG6_000190 | LG6 |  |  |  |  |
| DLA_LG6_000200 | LG6 |  |  |  |  |
| DLA_LG6_000205 | LG6 |  |  |  |  |
| DLA_LG6_000210 | LG6 |  |  |  |  |
| DLA_LG6_000220 | LG6 |  |  |  |  |
| DLA_LG6_000230 | LG6 |  |  |  |  |
| DLA_LG6_000240 | LG6 |  |  |  |  |
| DLA_LG6_000250 | LG6 |  |  |  |  |
| DLA_LG6_000260 | LG6 |  |  |  |  |
| DLA_LG6_000265 | LG6 |  |  |  |  |
| DLA_LG6_000265_2 | LG6 |  |  |  |  |
| DLA_LG6_000270 | LG6 |  |  |  |  |
| DLA_LG6_000280 | LG6 |  |  |  |  |
| DLA_LG6_000290 | LG6 |  |  |  |  |
| DLA_LG6_000300 | LG6 |  |  |  |  |
| DLA_LG6_000310 | LG6 |  |  |  |  |
| DLA_LG6_000330 | LG6 |  |  |  |  |
| DLA_LG6_000340 | LG6 |  |  |  |  |
| DLA_LG6_000350 | LG6 |  |  |  |  |
| DLA_LG6_000360 | LG6 |  |  |  |  |
| DLA_LG6_000365 | LG6 |  |  |  |  |
| DLA_LG6_000370 | LG6 |  |  |  |  |
| DLA_LG6_000380 | LG6 |  |  |  |  |
| DLA_LG6_000400 | LG6 |  |  |  |  |
| DLA_LG6_000410 | LG6 |  |  |  |  |
| DLA_LG6_000420 | LG6 |  |  |  |  |
| DLA_LG6_000430 | LG6 |  |  |  |  |
| DLA_LG6_000440 | LG6 |  |  |  |  |
| DLA_LG6_000450 | LG6 |  |  |  |  |
| DLA_LG6_000460 | LG6 |  |  |  |  |
| DLA_LG6_000470 | LG6 |  |  |  |  |
| DLA_LG6_000480 | LG6 |  |  |  |  |
| DLA_LG6_000490 | LG6 |  |  |  |  |
| DLA_LG6_000495 | LG6 |  |  |  |  |
| DLA_LG6_000520 | LG6 |  |  |  |  |
| DLA_LG6_000530 | LG6 |  |  |  |  |
| DLA_LG6_000540 | LG6 |  |  |  |  |
| DLA_LG6_000550 | LG6 |  |  |  |  |
| DLA_LG6_000555 | LG6 |  |  |  |  |
| DLA_LG6_000560 | LG6 |  |  |  |  |
| DLA_LG6_000570 | LG6 |  |  |  |  |
| DLA_LG6_000580 | LG6 |  |  |  |  |
| DLA_LG6_000590 | LG6 |  |  |  |  |
| DLA_LG6_000600 | LG6 |  |  |  |  |
| DLA_LG6_000620 | LG6 |  |  |  |  |
| DLA_LG6_000630 | LG6 |  |  |  |  |
| DLA_LG6_000640 | LG6 |  |  |  |  |
| DLA_LG6_000650 | LG6 |  |  |  |  |
| DLA_LG6_000670 | LG6 |  |  |  |  |
| DLA_LG6_000680 | LG6 |  |  |  |  |
| DLA_LG6_000690 | LG6 |  |  |  |  |
| DLA_LG6_000700 | LG6 |  |  |  |  |
| DLA_LG6_000710 | LG6 |  |  |  |  |
| DLA_LG6_000720 | LG6 |  |  |  |  |
| DLA_LG6_000725 | LG6 |  |  |  |  |
| DLA_LG6_000730 | LG6 |  |  |  |  |
| DLA_LG6_000740 | LG6 |  |  |  |  |
| DLA_LG6_000760 | LG6 |  |  |  |  |
| DLA_LG6_000770 | LG6 |  |  |  |  |
| DLA_LG6_000780 | LG6 |  |  |  |  |
| DLA_LG6_000800 | LG6 |  |  |  |  |
| DLA_LG6_000810 | LG6 |  |  |  |  |
| DLA_LG6_000820 | LG6 |  |  |  |  |
| DLA_LG6_000840 | LG6 |  |  |  |  |
| DLA_LG6_000850 | LG6 |  |  |  |  |
| DLA_LG6_000860 | LG6 |  |  |  |  |
| DLA_LG6_000870 | LG6 |  |  |  |  |
| DLA_LG6_000880 | LG6 |  |  |  |  |
| DLA_LG6_000890 | LG6 |  |  |  |  |
| DLA_LG6_000900 | LG6 |  |  |  |  |
| DLA_LG6_000910 | LG6 |  |  |  |  |
| DLA_LG6_000920 | LG6 |  |  |  |  |
| DLA_LG6_000930 | LG6 |  |  |  |  |
| DLA_LG6_000940 | LG6 |  |  |  |  |
| DLA_LG6_000950 | LG6 |  |  |  |  |
| DLA_LG6_000960 | LG6 |  |  |  |  |
| DLA_LG6_000970 | LG6 |  |  |  |  |
| DLA_LG6_000980 | LG6 |  |  |  |  |
| DLA_LG6_000990 | LG6 |  |  |  |  |
| DLA_LG6_000995 | LG6 |  |  |  |  |
| DLA_LG6_001005 | LG6 |  |  |  |  |
| DLA_LG6_001010 | LG6 |  |  |  |  |
| DLA_LG6_001030 | LG6 |  |  |  |  |
| DLA_LG6_001040 | LG6 |  |  |  |  |
| DLA_LG6_001060 | LG6 |  |  |  |  |
| DLA_LG6_001070 | LG6 |  |  |  |  |
| DLA_LG6_001080 | LG6 |  |  |  |  |
| DLA_LG6_001090 | LG6 |  |  |  |  |
| DLA_LG6_001100 | LG6 |  |  |  |  |
| DLA_LG6_001110 | LG6 |  |  |  |  |
| DLA_LG6_001120 | LG6 |  |  |  |  |
| DLA_LG6_001140 | LG6 |  |  |  |  |
| DLA_LG6_001150 | LG6 |  |  |  |  |
| DLA_LG6_001160 | LG6 |  |  |  |  |
| DLA_LG6_001170 | LG6 |  |  |  |  |
| DLA_LG6_001180 | LG6 |  |  |  |  |
| DLA_LG6_001190 | LG6 |  |  |  |  |
| DLA_LG6_001220 | LG6 |  |  |  |  |
| DLA_LG6_001230 | LG6 |  |  |  |  |
| DLA_LG6_001240 | LG6 |  |  |  |  |
| DLA_LG6_001250 | LG6 |  |  |  |  |
| DLA_LG6_001260 | LG6 |  |  |  |  |
| DLA_LG6_001280 | LG6 |  |  |  |  |
| DLA_LG6_001290 | LG6 |  |  |  |  |
| DLA_LG6_001300 | LG6 |  |  |  |  |
| DLA_LG6_001310 | LG6 |  |  |  |  |
| DLA_LG6_001320 | LG6 |  |  |  |  |
| DLA_LG6_001330 | LG6 |  |  |  |  |
| DLA_LG6_001340 | LG6 |  |  |  |  |
| DLA_LG6_001370 | LG6 |  |  |  |  |
| DLA_LG6_001380 | LG6 |  |  |  |  |
| DLA_LG6_001390 | LG6 |  |  |  |  |
| DLA_LG6_001405 | LG6 |  |  |  |  |
| DLA_LG6_001410 | LG6 |  |  |  |  |
| DLA_LG6_001420 | LG6 |  |  |  |  |
| DLA_LG6_001430 | LG6 |  |  |  |  |
| DLA_LG6_001440 | LG6 |  |  |  |  |
| DLA_LG6_001450 | LG6 |  |  |  |  |
| DLA_LG6_001470 | LG6 |  |  |  |  |
| DLA_LG6_001480 | LG6 |  |  |  |  |
| DLA_LG6_001500 | LG6 |  |  |  |  |
| DLA_LG6_001510 | LG6 |  |  |  |  |
| DLA_LG6_001520 | LG6 |  |  |  |  |
| DLA_LG6_001530 | LG6 |  |  |  |  |
| DLA_LG6_001540 | LG6 |  |  |  |  |
| DLA_LG6_001560 | LG6 |  |  |  |  |
| DLA_LG6_001570 | LG6 |  |  |  |  |
| DLA_LG6_001580 | LG6 |  |  |  |  |
| DLA_LG6_001590 | LG6 |  |  |  |  |
| DLA_LG6_001600 | LG6 |  |  |  |  |
| DLA_LG6_001610 | LG6 |  |  |  |  |
| DLA_LG6_001630 | LG6 |  |  |  |  |
| DLA_LG6_001640 | LG6 |  |  |  |  |
| DLA_LG6_001650 | LG6 |  |  |  |  |
| DLA_LG6_001660 | LG6 |  |  |  |  |
| DLA_LG6_001670 | LG6 |  |  |  |  |
| DLA_LG6_001680 | LG6 |  |  |  |  |
| DLA_LG6_001690 | LG6 |  |  |  |  |
| DLA_LG6_001700 | LG6 |  |  |  |  |
| DLA_LG6_001710 | LG6 |  |  |  |  |
| DLA_LG6_001715 | LG6 |  |  |  |  |
| DLA_LG6_001720 | LG6 |  |  |  |  |
| DLA_LG6_001730 | LG6 |  |  |  |  |
| DLA_LG6_001740 | LG6 |  |  |  |  |
| DLA_LG6_001750 | LG6 |  |  |  |  |
| DLA_LG6_001760 | LG6 |  |  |  |  |
| DLA_LG6_001780 | LG6 |  |  |  |  |
| DLA_LG6_001810 | LG6 |  |  |  |  |
| DLA_LG6_001820 | LG6 |  |  |  |  |
| DLA_LG6_001840 | LG6 |  |  |  |  |
| DLA_LG6_001870 | LG6 |  |  |  |  |
| DLA_LG6_001880 | LG6 |  |  |  |  |
| DLA_LG6_001890 | LG6 |  |  |  |  |
| DLA_LG6_001910 | LG6 |  |  |  |  |
| DLA_LG6_001920 | LG6 |  |  |  |  |
| DLA_LG6_001930 | LG6 |  |  |  |  |
| DLA_LG6_001940 | LG6 |  |  |  |  |
| DLA_LG6_001970 | LG6 |  |  |  |  |
| DLA_LG6_001990 | LG6 |  |  |  |  |
| DLA_LG6_002000 | LG6 |  |  |  |  |
| DLA_LG6_002010 | LG6 |  |  |  |  |
| DLA_LG6_002020 | LG6 |  |  |  |  |
| DLA_LG6_002030 | LG6 |  |  |  |  |
| DLA_LG6_002035 | LG6 |  |  |  |  |
| DLA_LG6_002040 | LG6 |  |  |  |  |
| DLA_LG6_002050 | LG6 |  |  |  |  |
| DLA_LG6_002060 | LG6 |  |  |  |  |
| DLA_LG6_002070 | LG6 |  |  |  |  |
| DLA_LG6_002080 | LG6 |  |  |  |  |
| DLA_LG6_002100 | LG6 |  |  |  |  |
| DLA_LG6_002110 | LG6 |  |  |  |  |
| DLA_LG6_002120 | LG6 |  |  |  |  |
| DLA_LG6_002140 | LG6 |  |  |  |  |
| DLA_LG6_002150 | LG6 |  |  |  |  |
| DLA_LG6_002160 | LG6 |  |  |  |  |
| DLA_LG6_002170 | LG6 |  |  |  |  |
| DLA_LG6_002190 | LG6 |  |  |  |  |
| DLA_LG6_002200 | LG6 |  |  |  |  |
| DLA_LG6_002210 | LG6 |  |  |  |  |
| DLA_LG6_002220 | LG6 |  |  |  |  |
| DLA_LG6_002230 | LG6 |  |  |  |  |
| DLA_LG6_002240 | LG6 |  |  |  |  |
| DLA_LG6_002250 | LG6 |  |  |  |  |
| DLA_LG6_002260 | LG6 |  |  |  |  |
| DLA_LG6_002270 | LG6 |  |  |  |  |
| DLA_LG6_002290 | LG6 |  |  |  |  |
| DLA_LG6_002300 | LG6 |  |  |  |  |
| DLA_LG6_002310 | LG6 |  |  |  |  |
| DLA_LG6_002330 | LG6 |  |  |  |  |
| DLA_LG6_002340 | LG6 |  |  |  |  |
| DLA_LG6_002350 | LG6 |  |  |  |  |
| DLA_LG6_002360 | LG6 |  |  |  |  |
| DLA_LG6_002370 | LG6 |  |  |  |  |
| DLA_LG6_002380 | LG6 |  |  |  |  |
| DLA_LG6_002390 | LG6 |  |  |  |  |
| DLA_LG6_002400 | LG6 |  |  |  |  |
| DLA_LG6_002410 | LG6 |  |  |  |  |
| DLA_LG6_002415 | LG6 |  |  |  |  |
| DLA_LG6_002440 | LG6 |  |  |  |  |
| DLA_LG6_002450 | LG6 |  |  |  |  |
| DLA_LG6_002460 | LG6 |  |  |  |  |
| DLA_LG6_002480 | LG6 |  |  |  |  |
| DLA_LG6_002490 | LG6 |  |  |  |  |
| DLA_LG6_002500 | LG6 |  |  |  |  |
| DLA_LG6_002510 | LG6 |  |  |  |  |
| DLA_LG6_002520 | LG6 |  |  |  |  |
| DLA_LG6_002530 | LG6 |  |  |  |  |
| DLA_LG6_002560 | LG6 |  |  |  |  |
| DLA_LG6_002570 | LG6 |  |  |  |  |
| DLA_LG6_002580 | LG6 |  |  |  |  |
| DLA_LG6_002590 | LG6 |  |  |  |  |
| DLA_LG6_002600 | LG6 |  |  |  |  |
| DLA_LG6_002610 | LG6 |  |  |  |  |
| DLA_LG6_002620 | LG6 |  |  |  |  |
| DLA_LG6_002630 | LG6 |  |  |  |  |
| DLA_LG6_002650 | LG6 |  |  |  |  |
| DLA_LG6_002660 | LG6 |  |  |  |  |
| DLA_LG6_002670 | LG6 |  |  |  |  |
| DLA_LG6_002690 | LG6 |  |  |  |  |
| DLA_LG6_002700 | LG6 |  |  |  |  |
| DLA_LG6_002710 | LG6 |  |  |  |  |
| DLA_LG6_002720 | LG6 |  |  |  |  |
| DLA_LG6_002725 | LG6 |  |  |  |  |
| DLA_LG6_002730 | LG6 |  |  |  |  |
| DLA_LG6_002740 | LG6 |  |  |  |  |
| DLA_LG6_002750 | LG6 |  |  |  |  |
| DLA_LG6_002760 | LG6 |  |  |  |  |
| DLA_LG6_002775 | LG6 |  |  |  |  |
| DLA_LG6_002780 | LG6 |  |  |  |  |
| DLA_LG6_002790 | LG6 |  |  |  |  |
| DLA_LG6_002810 | LG6 |  |  |  |  |
| DLA_LG6_002820 | LG6 |  |  |  |  |
| DLA_LG6_002825 | LG6 |  |  |  |  |
| DLA_LG6_002840 | LG6 |  |  |  |  |
| DLA_LG6_002850 | LG6 |  |  |  |  |
| DLA_LG6_002870 | LG6 |  |  |  |  |
| DLA_LG6_002880 | LG6 |  |  |  |  |
| DLA_LG6_002890 | LG6 |  |  |  |  |
| DLA_LG6_002900 | LG6 |  |  |  |  |
| DLA_LG6_002910 | LG6 |  |  |  |  |
| DLA_LG6_002920 | LG6 |  |  |  |  |
| DLA_LG6_002930 | LG6 |  |  |  |  |
| DLA_LG6_002940 | LG6 |  |  |  |  |
| DLA_LG6_002950 | LG6 |  |  |  |  |
| DLA_LG6_002960 | LG6 |  |  |  |  |
| DLA_LG6_002970 | LG6 |  |  |  |  |
| DLA_LG6_002980 | LG6 |  |  |  |  |
| DLA_LG6_003000 | LG6 |  |  |  |  |
| DLA_LG6_003010 | LG6 |  |  |  |  |
| DLA_LG6_003020 | LG6 |  |  |  |  |
| DLA_LG6_003030 | LG6 |  |  |  |  |
| DLA_LG6_003040 | LG6 |  |  |  |  |
| DLA_LG6_003050 | LG6 |  |  |  |  |
| DLA_LG6_003060 | LG6 |  |  |  |  |
| DLA_LG6_003070 | LG6 |  |  |  |  |
| DLA_LG6_003080 | LG6 |  |  |  |  |
| DLA_LG6_003090 | LG6 |  |  |  |  |
| DLA_LG6_003100 | LG6 |  |  |  |  |
| DLA_LG6_003110 | LG6 |  |  |  |  |
| DLA_LG6_003120 | LG6 |  |  |  |  |
| DLA_LG6_003130 | LG6 |  |  |  |  |
| DLA_LG6_003140 | LG6 |  |  |  |  |
| DLA_LG6_003160 | LG6 |  |  |  |  |
| DLA_LG6_003170 | LG6 |  |  |  |  |
| DLA_LG6_003180 | LG6 |  |  |  |  |
| DLA_LG6_003190 | LG6 |  |  |  |  |
| DLA_LG6_003200 | LG6 |  |  |  |  |
| DLA_LG6_003210 | LG6 |  |  |  |  |
| DLA_LG6_003220 | LG6 |  |  |  |  |
| DLA_LG6_003240 | LG6 |  |  |  |  |
| DLA_LG6_003250 | LG6 |  |  |  |  |
| DLA_LG6_003270 | LG6 |  |  |  |  |
| DLA_LG6_003280 | LG6 |  |  |  |  |
| DLA_LG6_003290 | LG6 |  |  |  |  |
| DLA_LG6_003310 | LG6 |  |  |  |  |
| DLA_LG6_003320 | LG6 |  |  |  |  |
| DLA_LG6_003330 | LG6 |  |  |  |  |
| DLA_LG6_003340 | LG6 |  |  |  |  |
| DLA_LG6_003350 | LG6 |  |  |  |  |
| DLA_LG6_003360 | LG6 |  |  |  |  |
| DLA_LG6_003370 | LG6 |  |  |  |  |
| DLA_LG6_003380 | LG6 |  |  |  |  |
| DLA_LG6_003390 | LG6 |  |  |  |  |
| DLA_LG6_003400 | LG6 |  |  |  |  |
| DLA_LG6_003410 | LG6 |  |  |  |  |
| DLA_LG6_003420 | LG6 |  |  |  |  |
| DLA_LG6_003430 | LG6 |  |  |  |  |
| DLA_LG6_003440 | LG6 |  |  |  |  |
| DLA_LG6_003460 | LG6 |  |  |  |  |
| DLA_LG6_003470 | LG6 |  |  |  |  |
| DLA_LG6_003480 | LG6 |  |  |  |  |
| DLA_LG6_003490 | LG6 |  |  |  |  |
| DLA_LG6_003510 | LG6 |  |  |  |  |
| DLA_LG6_003520 | LG6 |  |  |  |  |
| DLA_LG6_003530 | LG6 |  |  |  |  |
| DLA_LG6_003540 | LG6 |  |  |  |  |
| DLA_LG6_003560 | LG6 |  |  |  |  |
| DLA_LG6_003580 | LG6 |  |  |  |  |
| DLA_LG6_003590 | LG6 |  |  |  |  |
| DLA_LG6_003600 | LG6 |  |  |  |  |
| DLA_LG6_003620 | LG6 |  |  |  |  |
| DLA_LG6_003630 | LG6 |  |  |  |  |
| DLA_LG6_003640 | LG6 |  |  |  |  |
| DLA_LG6_003650 | LG6 |  |  |  |  |
| DLA_LG6_003660 | LG6 |  |  |  |  |
| DLA_LG6_003680 | LG6 |  |  |  |  |
| DLA_LG6_003710 | LG6 |  |  |  |  |
| DLA_LG6_003720 | LG6 |  |  |  |  |
| DLA_LG6_003740 | LG6 |  |  |  |  |
| DLA_LG6_003760 | LG6 |  |  |  |  |
| DLA_LG6_003770 | LG6 |  |  |  |  |
| DLA_LG6_003780 | LG6 |  |  |  |  |
| DLA_LG6_003790 | LG6 |  |  |  |  |
| DLA_LG6_003800 | LG6 |  |  |  |  |
| DLA_LG6_003810 | LG6 |  |  |  |  |
| DLA_LG6_003820 | LG6 |  |  |  |  |
| DLA_LG6_003825 | LG6 |  |  |  |  |
| DLA_LG6_003830 | LG6 |  |  |  |  |
| DLA_LG6_003840 | LG6 |  |  |  |  |
| DLA_LG6_003850 | LG6 |  |  |  |  |
| DLA_LG6_003870 | LG6 |  |  |  |  |
| DLA_LG6_003880 | LG6 |  |  |  |  |
| DLA_LG6_003890 | LG6 |  |  |  |  |
| DLA_LG6_003900 | LG6 |  |  |  |  |
| DLA_LG6_003905 | LG6 |  |  |  |  |
| DLA_LG6_003910 | LG6 |  |  |  |  |
| DLA_LG6_003920 | LG6 |  |  |  |  |
| DLA_LG6_003930 | LG6 |  |  |  |  |
| DLA_LG6_003940 | LG6 |  |  |  |  |
| DLA_LG6_003970 | LG6 |  |  |  |  |
| DLA_LG6_003980 | LG6 |  |  |  |  |
| DLA_LG6_003990 | LG6 |  |  |  |  |
| DLA_LG6_004000 | LG6 |  |  |  |  |
| DLA_LG6_004010 | LG6 |  |  |  |  |
| DLA_LG6_004020 | LG6 |  |  |  |  |
| DLA_LG6_004030 | LG6 |  |  |  |  |
| DLA_LG6_004050 | LG6 |  |  |  |  |
| DLA_LG6_004060 | LG6 |  |  |  |  |
| DLA_LG6_004065 | LG6 |  |  |  |  |
| DLA_LG6_004070 | LG6 |  |  |  |  |
| DLA_LG6_004080 | LG6 |  |  |  |  |
| DLA_LG6_004090 | LG6 |  |  |  |  |
| DLA_LG6_004100 | LG6 |  |  |  |  |
| DLA_LG6_004110 | LG6 |  |  |  |  |
| DLA_LG6_004120 | LG6 |  |  |  |  |
| DLA_LG6_004130 | LG6 |  |  |  |  |
| DLA_LG6_004140 | LG6 |  |  |  |  |
| DLA_LG6_004150 | LG6 |  |  |  |  |
| DLA_LG6_004160 | LG6 |  |  |  |  |
| DLA_LG6_004170 | LG6 |  |  |  |  |
| DLA_LG6_004180 | LG6 |  |  |  |  |
| DLA_LG6_004185 | LG6 |  |  |  |  |
| DLA_LG6_004190 | LG6 |  |  |  |  |
| DLA_LG6_004200 | LG6 |  |  |  |  |
| DLA_LG6_004210 | LG6 |  |  |  |  |
| DLA_LG6_004230 | LG6 |  |  |  |  |
| DLA_LG6_004240 | LG6 |  |  |  |  |
| DLA_LG6_004250 | LG6 |  |  |  |  |
| DLA_LG6_004260 | LG6 |  |  |  |  |
| DLA_LG6_004280 | LG6 |  |  |  |  |
| DLA_LG6_004290 | LG6 |  |  |  |  |
| DLA_LG6_004300 | LG6 |  |  |  |  |
| DLA_LG6_004310 | LG6 |  |  |  |  |
| DLA_LG6_004320 | LG6 |  |  |  |  |
| DLA_LG6_004330 | LG6 |  |  |  |  |
| DLA_LG6_004340 | LG6 |  |  |  |  |
| DLA_LG6_004350 | LG6 |  |  |  |  |
| DLA_LG6_004360 | LG6 |  |  |  |  |
| DLA_LG6_004370 | LG6 |  |  |  |  |
| DLA_LG6_004380 | LG6 |  |  |  |  |
| DLA_LG6_004390 | LG6 |  |  |  |  |
| DLA_LG6_004410 | LG6 |  |  |  |  |
| DLA_LG6_004420 | LG6 |  |  |  |  |
| DLA_LG6_004430 | LG6 |  |  |  |  |
| DLA_LG6_004440 | LG6 |  |  |  |  |
| DLA_LG6_004450 | LG6 |  |  |  |  |
| DLA_LG6_004470 | LG6 |  |  |  |  |
| DLA_LG6_004490 | LG6 |  |  |  |  |
| DLA_LG6_004500 | LG6 |  |  |  |  |
| DLA_LG6_004510 | LG6 |  |  |  |  |
| DLA_LG6_004520 | LG6 |  |  |  |  |
| DLA_LG6_004530 | LG6 |  |  |  |  |
| DLA_LG6_004540 | LG6 |  |  |  |  |
| DLA_LG6_004550 | LG6 |  |  |  |  |
| DLA_LG6_004560 | LG6 |  |  |  |  |
| DLA_LG6_004570 | LG6 |  |  |  |  |
| DLA_LG6_004580 | LG6 |  |  |  |  |
| DLA_LG6_004590 | LG6 |  |  |  |  |
| DLA_LG6_004610 | LG6 |  |  |  |  |
| DLA_LG6_004620 | LG6 |  |  |  |  |
| DLA_LG6_004630 | LG6 |  |  |  |  |
| DLA_LG6_004640 | LG6 |  |  |  |  |
| DLA_LG6_004650 | LG6 |  |  |  |  |
| DLA_LG6_004660 | LG6 |  |  |  |  |
| DLA_LG6_004680 | LG6 |  |  |  |  |
| DLA_LG6_004705 | LG6 |  |  |  |  |
| DLA_LG6_004710 | LG6 |  |  |  |  |
| DLA_LG6_004730 | LG6 |  |  |  |  |
| DLA_LG6_004740 | LG6 |  |  |  |  |
| DLA_LG6_004750 | LG6 |  |  |  |  |
| DLA_LG6_004755 | LG6 |  |  |  |  |
| DLA_LG6_004760 | LG6 |  |  |  |  |
| DLA_LG6_004770 | LG6 |  |  |  |  |
| DLA_LG6_004780 | LG6 |  |  |  |  |
| DLA_LG6_004790 | LG6 |  |  |  |  |
| DLA_LG6_004810 | LG6 |  |  |  |  |
| DLA_LG6_004820 | LG6 |  |  |  |  |
| DLA_LG6_004830 | LG6 |  |  |  |  |
| DLA_LG6_004840 | LG6 |  |  |  |  |
| DLA_LG6_004850 | LG6 |  |  |  |  |
| DLA_LG6_004860 | LG6 |  |  |  |  |
| DLA_LG6_004870 | LG6 |  |  |  |  |
| DLA_LG6_004880 | LG6 |  |  |  |  |
| DLA_LG6_004890 | LG6 |  |  |  |  |
| DLA_LG6_004900 | LG6 |  |  |  |  |
| DLA_LG6_004920 | LG6 |  |  |  |  |
| DLA_LG6_004930 | LG6 |  |  |  |  |
| DLA_LG6_004940 | LG6 |  |  |  |  |
| DLA_LG6_004950 | LG6 |  |  |  |  |
| DLA_LG6_004960 | LG6 |  |  |  |  |
| DLA_LG6_004970 | LG6 |  |  |  |  |
| DLA_LG6_004980 | LG6 |  |  |  |  |
| DLA_LG6_004990 | LG6 |  |  |  |  |
| DLA_LG6_005000 | LG6 |  |  |  |  |
| DLA_LG6_005010 | LG6 |  |  |  |  |
| DLA_LG6_005020 | LG6 |  |  |  |  |
| DLA_LG6_005030 | LG6 |  |  |  |  |
| DLA_LG6_005040 | LG6 |  |  |  |  |
| DLA_LG6_005050 | LG6 |  |  |  |  |
| DLA_LG6_005060 | LG6 |  |  |  |  |
| DLA_LG6_005070 | LG6 |  |  |  |  |
| DLA_LG6_005080 | LG6 |  |  |  |  |
| DLA_LG6_005090 | LG6 |  |  |  |  |
| DLA_LG6_005100 | LG6 |  |  |  |  |
| DLA_LG6_005110 | LG6 |  |  |  |  |
| DLA_LG6_005115 | LG6 |  |  |  |  |
| DLA_LG6_005120 | LG6 |  |  |  |  |
| DLA_LG6_005125 | LG6 |  |  |  |  |
| DLA_LG6_005125_2 | LG6 |  |  |  |  |
| DLA_LG6_005130 | LG6 |  |  |  |  |
| DLA_LG6_005140 | LG6 |  |  |  |  |
| DLA_LG6_005150 | LG6 |  |  |  |  |
| DLA_LG6_005160 | LG6 |  |  |  |  |
| DLA_LG6_005170 | LG6 |  |  |  |  |
| DLA_LG6_005190 | LG6 |  |  |  |  |
| DLA_LG6_005200 | LG6 |  |  |  |  |
| DLA_LG6_005220 | LG6 |  |  |  |  |
| DLA_LG6_005230 | LG6 |  |  |  |  |
| DLA_LG6_005240 | LG6 |  |  |  |  |
| DLA_LG6_005260 | LG6 |  |  |  |  |
| DLA_LG6_005270 | LG6 |  |  |  |  |
| DLA_LG6_005280 | LG6 |  |  |  |  |
| DLA_LG6_005290 | LG6 |  |  |  |  |
| DLA_LG6_005300 | LG6 |  |  |  |  |
| DLA_LG6_005310 | LG6 |  |  |  |  |
| DLA_LG6_005320 | LG6 |  |  |  |  |
| DLA_LG6_005340 | LG6 |  |  |  |  |
| DLA_LG6_005350 | LG6 |  |  |  |  |
| DLA_LG6_005360 | LG6 |  |  |  |  |
| DLA_LG6_005370 | LG6 |  |  |  |  |
| DLA_LG6_005380 | LG6 |  |  |  |  |
| DLA_LG6_005400 | LG6 |  |  |  |  |
| DLA_LG6_005410 | LG6 |  |  |  |  |
| DLA_LG6_005420 | LG6 |  |  |  |  |
| DLA_LG6_005430 | LG6 |  |  |  |  |
| DLA_LG6_005450 | LG6 |  |  |  |  |
| DLA_LG6_005460 | LG6 |  |  |  |  |
| DLA_LG6_005470 | LG6 |  |  |  |  |
| DLA_LG6_005480 | LG6 |  |  |  |  |
| DLA_LG6_005490 | LG6 |  |  |  |  |
| DLA_LG6_005500 | LG6 |  |  |  |  |
| DLA_LG6_005510 | LG6 |  |  |  |  |
| DLA_LG6_005520 | LG6 |  |  |  |  |
| DLA_LG6_005530 | LG6 |  |  |  |  |
| DLA_LG6_005550 | LG6 |  |  |  |  |
| DLA_LG6_005560 | LG6 |  |  |  |  |
| DLA_LG6_005570 | LG6 |  |  |  |  |
| DLA_LG6_005580 | LG6 |  |  |  |  |
| DLA_LG6_005590 | LG6 |  |  |  |  |
| DLA_LG6_005600 | LG6 |  |  |  |  |
| DLA_LG6_005620 | LG6 |  |  |  |  |
| DLA_LG6_005630 | LG6 |  |  |  |  |
| DLA_LG6_005640 | LG6 |  |  |  |  |
| DLA_LG6_005650 | LG6 |  |  |  |  |
| DLA_LG6_005660 | LG6 |  |  |  |  |
| DLA_LG6_005700 | LG6 |  |  |  |  |
| DLA_LG6_005710 | LG6 |  |  |  |  |
| DLA_LG6_005720 | LG6 |  |  |  |  |
| DLA_LG6_005730 | LG6 |  |  |  |  |
| DLA_LG6_005740 | LG6 |  |  |  |  |
| DLA_LG6_005750 | LG6 |  |  |  |  |
| DLA_LG6_005770 | LG6 |  |  |  |  |
| DLA_LG6_005780 | LG6 |  |  |  |  |
| DLA_LG6_005790 | LG6 |  |  |  |  |
| DLA_LG6_005800 | LG6 |  |  |  |  |
| DLA_LG6_005810 | LG6 |  |  |  |  |
| DLA_LG6_005850 | LG6 |  |  |  |  |
| DLA_LG6_005860 | LG6 |  |  |  |  |
| DLA_LG6_005870 | LG6 |  |  |  |  |
| DLA_LG6_005880 | LG6 |  |  |  |  |
| DLA_LG6_005890 | LG6 |  |  |  |  |
| DLA_LG6_005900 | LG6 |  |  |  |  |
| DLA_LG6_005920 | LG6 |  |  |  |  |
| DLA_LG6_005930 | LG6 |  |  |  |  |
| DLA_LG6_005960 | LG6 |  |  |  |  |
| DLA_LG6_005970 | LG6 |  |  |  |  |
| DLA_LG6_005980 | LG6 |  |  |  |  |
| DLA_LG6_006000 | LG6 |  |  |  |  |
| DLA_LG6_006010 | LG6 |  |  |  |  |
| DLA_LG6_006020 | LG6 |  |  |  |  |
| DLA_LG6_006030 | LG6 |  |  |  |  |
| DLA_LG6_006040 | LG6 |  |  |  |  |
| DLA_LG6_006050 | LG6 |  |  |  |  |
| DLA_LG6_006060 | LG6 |  |  |  |  |
| DLA_LG6_006070 | LG6 |  |  |  |  |
| DLA_LG6_006090 | LG6 |  |  |  |  |
| DLA_LG6_006100 | LG6 |  |  |  |  |
| DLA_LG6_006120 | LG6 |  |  |  |  |
| DLA_LG6_006130 | LG6 |  |  |  |  |
| DLA_LG6_006140 | LG6 |  |  |  |  |
| DLA_LG6_006145 | LG6 |  |  |  |  |
| DLA_LG6_006150 | LG6 |  |  |  |  |
| DLA_LG6_006170 | LG6 |  |  |  |  |
| DLA_LG6_006180 | LG6 |  |  |  |  |
| DLA_LG6_006190 | LG6 |  |  |  |  |
| DLA_LG6_006200 | LG6 |  |  |  |  |
| DLA_LG6_006220 | LG6 |  |  |  |  |
| DLA_LG6_006230 | LG6 |  |  |  |  |
| DLA_LG6_006240 | LG6 |  |  |  |  |
| DLA_LG6_006250 | LG6 |  |  |  |  |
| DLA_LG6_006270 | LG6 |  |  |  |  |
| DLA_LG6_006280 | LG6 |  |  |  |  |
| DLA_LG6_006310 | LG6 |  |  |  |  |
| DLA_LG6_006320 | LG6 |  |  |  |  |
| DLA_LG6_006330 | LG6 |  |  |  |  |
| DLA_LG6_006340 | LG6 |  |  |  |  |
| DLA_LG6_006350 | LG6 |  |  |  |  |
| DLA_LG6_006360 | LG6 |  |  |  |  |
| DLA_LG6_006365 | LG6 |  |  |  |  |
| DLA_LG6_006365_2 | LG6 |  |  |  |  |
| DLA_LG6_006365_3 | LG6 |  |  |  |  |
| DLA_LG6_006365_4 | LG6 |  |  |  |  |
| DLA_LG6_006370 | LG6 |  |  |  |  |
| DLA_LG6_006380 | LG6 |  |  |  |  |
| DLA_LG6_006390 | LG6 |  |  |  |  |
| DLA_LG6_006400 | LG6 |  |  |  |  |
| DLA_LG6_006420 | LG6 |  |  |  |  |
| DLA_LG6_006430 | LG6 |  |  |  |  |
| DLA_LG6_006450 | LG6 |  |  |  |  |
| DLA_LG6_006460 | LG6 |  |  |  |  |
| DLA_LG6_006470 | LG6 |  |  |  |  |
| DLA_LG6_006480 | LG6 |  |  |  |  |
| DLA_LG6_006490 | LG6 |  |  |  |  |
| DLA_LG6_006500 | LG6 |  |  |  |  |
| DLA_LG6_006530 | LG6 |  |  |  |  |
| DLA_LG6_006550 | LG6 |  |  |  |  |
| DLA_LG6_006560 | LG6 |  |  |  |  |
| DLA_LG6_006570 | LG6 |  |  |  |  |
| DLA_LG6_006580 | LG6 |  |  |  |  |
| DLA_LG6_006590 | LG6 |  |  |  |  |
| DLA_LG6_006600 | LG6 |  |  |  |  |
| DLA_LG6_006615 | LG6 |  |  |  |  |
| DLA_LG6_006620 | LG6 |  |  |  |  |
| DLA_LG6_006630 | LG6 |  |  |  |  |
| DLA_LG6_006640 | LG6 |  |  |  |  |
| DLA_LG6_006650 | LG6 |  |  |  |  |
| DLA_LG6_006660 | LG6 |  |  |  |  |
| DLA_LG6_006670 | LG6 |  |  |  |  |
| DLA_LG6_006680 | LG6 |  |  |  |  |
| DLA_LG6_006690 | LG6 |  |  |  |  |
| DLA_LG6_006700 | LG6 |  |  |  |  |
| DLA_LG6_006720 | LG6 |  |  |  |  |
| DLA_LG6_006730 | LG6 |  |  |  |  |
| DLA_LG6_006740 | LG6 |  |  |  |  |
| DLA_LG6_006750 | LG6 |  |  |  |  |
| DLA_LG6_006760 | LG6 |  |  |  |  |
| DLA_LG6_006770 | LG6 |  |  |  |  |
| DLA_LG6_006780 | LG6 |  |  |  |  |
| DLA_LG6_006790 | LG6 |  |  |  |  |
| DLA_LG6_006800 | LG6 |  |  |  |  |
| DLA_LG6_006810 | LG6 |  |  |  |  |
| DLA_LG6_006820 | LG6 |  |  |  |  |
| DLA_LG6_006830 | LG6 |  |  |  |  |
| DLA_LG6_006835 | LG6 |  |  |  |  |
| DLA_LG6_006840 | LG6 |  |  |  |  |
| DLA_LG6_006850 | LG6 |  |  |  |  |
| DLA_LG6_006880 | LG6 |  |  |  |  |
| DLA_LG6_006890 | LG6 |  |  |  |  |
| DLA_LG6_006900 | LG6 |  |  |  |  |
| DLA_LG6_006910 | LG6 |  |  |  |  |
| DLA_LG6_006930 | LG6 |  |  |  |  |
| DLA_LG6_006940 | LG6 |  |  |  |  |
| DLA_LG6_006950 | LG6 |  |  |  |  |
| DLA_LG6_006970 | LG6 |  |  |  |  |
| DLA_LG6_006980 | LG6 |  |  |  |  |
| DLA_LG6_006990 | LG6 |  |  |  |  |
| DLA_LG6_007000 | LG6 |  |  |  |  |
| DLA_LG6_007010 | LG6 |  |  |  |  |
| DLA_LG6_007020 | LG6 |  |  |  |  |
| DLA_LG6_007030 | LG6 |  |  |  |  |
| DLA_LG6_007035 | LG6 |  |  |  |  |
| DLA_LG6_007050 | LG6 |  |  |  |  |
| DLA_LG6_007060 | LG6 |  |  |  |  |
| DLA_LG6_007070 | LG6 |  |  |  |  |
| DLA_LG6_007080 | LG6 |  |  |  |  |
| DLA_LG6_007090 | LG6 |  |  |  |  |
| DLA_LG6_007100 | LG6 |  |  |  |  |
| DLA_LG6_007110 | LG6 |  |  |  |  |
| DLA_LG6_007120 | LG6 |  |  |  |  |
| DLA_LG6_007130 | LG6 |  |  |  |  |
| DLA_LG6_007140 | LG6 |  |  |  |  |
| DLA_LG6_007150 | LG6 |  |  |  |  |
| DLA_LG6_007160 | LG6 |  |  |  |  |
| DLA_LG6_007170 | LG6 |  |  |  |  |
| DLA_LG6_007180 | LG6 |  |  |  |  |
| DLA_LG6_007190 | LG6 |  |  |  |  |
| DLA_LG6_007210 | LG6 |  |  |  |  |
| DLA_LG6_007230 | LG6 |  |  |  |  |
| DLA_LG6_007235 | LG6 |  |  |  |  |
| DLA_LG6_007240 | LG6 |  |  |  |  |
| DLA_LG6_007260 | LG6 |  |  |  |  |
| DLA_LG6_007280 | LG6 |  |  |  |  |
| DLA_LG6_007290 | LG6 |  |  |  |  |
| DLA_LG6_007300 | LG6 |  |  |  |  |
| DLA_LG6_007310 | LG6 |  |  |  |  |
| DLA_LG6_007320 | LG6 |  |  |  |  |
| DLA_LG6_007330 | LG6 |  |  |  |  |
| DLA_LG6_007360 | LG6 |  |  |  |  |
| DLA_LG6_007370 | LG6 |  |  |  |  |
| DLA_LG6_007390 | LG6 |  |  |  |  |
| DLA_LG6_007400 | LG6 |  |  |  |  |
| DLA_LG6_007410 | LG6 |  |  |  |  |
| DLA_LG6_007430 | LG6 |  |  |  |  |
| DLA_LG6_007450 | LG6 |  |  |  |  |
| DLA_LG6_007460 | LG6 |  |  |  |  |
| DLA_LG6_007470 | LG6 |  |  |  |  |
| DLA_LG6_007480 | LG6 |  |  |  |  |
| DLA_LG6_007490 | LG6 |  |  |  |  |
| DLA_LG6_007500 | LG6 |  |  |  |  |
| DLA_LG6_007510 | LG6 |  |  |  |  |
| DLA_LG6_007520 | LG6 |  |  |  |  |
| DLA_LG6_007530 | LG6 |  |  |  |  |
| DLA_LG6_007540 | LG6 |  |  |  |  |
| DLA_LG6_007550 | LG6 |  |  |  |  |
| DLA_LG6_007555 | LG6 |  |  |  |  |
| DLA_LG6_007560 | LG6 |  |  |  |  |
| DLA_LG6_007570 | LG6 |  |  |  |  |
| DLA_LG6_007580 | LG6 |  |  |  |  |
| DLA_LG6_007590 | LG6 |  |  |  |  |
| DLA_LG6_007610 | LG6 |  |  |  |  |
| DLA_LG6_007615 | LG6 |  |  |  |  |
| DLA_LG6_007630 | LG6 |  |  |  |  |
| DLA_LG6_007640 | LG6 |  |  |  |  |
| DLA_LG6_007645 | LG6 |  |  |  |  |
| DLA_LG6_007650 | LG6 |  |  |  |  |
| DLA_LG6_007660 | LG6 |  |  |  |  |
| DLA_LG6_007670 | LG6 |  |  |  |  |
| DLA_LG6_007680 | LG6 |  |  |  |  |
| DLA_LG6_007690 | LG6 |  |  |  |  |
| DLA_LG6_007700 | LG6 |  |  |  |  |
| DLA_LG6_007720 | LG6 |  |  |  |  |
| DLA_LG6_007740 | LG6 |  |  |  |  |
| DLA_LG6_007745 | LG6 |  |  |  |  |
| DLA_LG6_007750 | LG6 |  |  |  |  |
| DLA_LG6_007770 | LG6 |  |  |  |  |
| DLA_LG6_007780 | LG6 |  |  |  |  |
| DLA_LG6_007790 | LG6 |  |  |  |  |
| DLA_LG6_007795 | LG6 |  |  |  |  |
| DLA_LG6_007800 | LG6 |  |  |  |  |
| DLA_LG6_007815 | LG6 |  |  |  |  |
| DLA_LG6_007820 | LG6 |  |  |  |  |
| DLA_LG6_007830 | LG6 |  |  |  |  |
| DLA_LG6_007850 | LG6 |  |  |  |  |
| DLA_LG6_007860 | LG6 |  |  |  |  |
| DLA_LG6_007880 | LG6 |  |  |  |  |
| DLA_LG6_007890 | LG6 |  |  |  |  |
| DLA_LG6_007910 | LG6 |  |  |  |  |
| DLA_LG6_007920 | LG6 |  |  |  |  |
| DLA_LG6_007930 | LG6 |  |  |  |  |
| DLA_LG6_007935 | LG6 |  |  |  |  |
| DLA_LG6_007940 | LG6 |  |  |  |  |
| DLA_LG6_007950 | LG6 |  |  |  |  |
| DLA_LG6_007970 | LG6 |  |  |  |  |
| DLA_LG6_007980 | LG6 |  |  |  |  |
| DLA_LG6_007990 | LG6 |  |  |  |  |
| DLA_LG6_008000 | LG6 |  |  |  |  |
| DLA_LG6_008010 | LG6 |  |  |  |  |
| DLA_LG6_008020 | LG6 |  |  |  |  |
| DLA_LG6_008030 | LG6 |  |  |  |  |
| DLA_LG6_008040 | LG6 |  |  |  |  |
| DLA_LG6_008060 | LG6 |  |  |  |  |
| DLA_LG6_008070 | LG6 |  |  |  |  |
| DLA_LG6_008080 | LG6 |  |  |  |  |
| DLA_LG6_008090 | LG6 |  |  |  |  |
| DLA_LG6_008120 | LG6 |  |  |  |  |
| DLA_LG6_008150 | LG6 |  |  |  |  |
| DLA_LG6_008170 | LG6 |  |  |  |  |
| DLA_LG6_008180 | LG6 |  |  |  |  |
| DLA_LG6_008190 | LG6 |  |  |  |  |
| DLA_LG6_008200 | LG6 |  |  |  |  |
| DLA_LG6_008210 | LG6 |  |  |  |  |
| DLA_LG6_008220 | LG6 |  |  |  |  |
| DLA_LG6_008230 | LG6 |  |  |  |  |
| DLA_LG6_008240 | LG6 |  |  |  |  |
| DLA_LG6_008250 | LG6 |  |  |  |  |
| DLA_LG6_008260 | LG6 |  |  |  |  |
| DLA_LG6_008270 | LG6 |  |  |  |  |
| DLA_LG6_008280 | LG6 |  |  |  |  |
| DLA_LG6_008290 | LG6 |  |  |  |  |
| DLA_LG6_008310 | LG6 |  |  |  |  |
| DLA_LG6_008320 | LG6 |  |  |  |  |
| DLA_LG6_008325 | LG6 |  |  |  |  |
| DLA_LG6_008330 | LG6 |  |  |  |  |
| DLA_LG6_008360 | LG6 |  |  |  |  |
| DLA_LG6_008370 | LG6 |  |  |  |  |
| DLA_LG6_008380 | LG6 |  |  |  |  |
| DLA_LG6_008390 | LG6 |  |  |  |  |
| DLA_LG6_008410 | LG6 |  |  |  |  |
| DLA_LG6_008420 | LG6 |  |  |  |  |
| DLA_LG6_008430 | LG6 |  |  |  |  |
| DLA_LG6_008460 | LG6 |  |  |  |  |
| DLA_LG6_008470 | LG6 |  |  |  |  |
| DLA_LG6_008480 | LG6 |  |  |  |  |
| DLA_LG6_008490 | LG6 |  |  |  |  |
| DLA_LG6_008500 | LG6 |  |  |  |  |
| DLA_LG6_008510 | LG6 |  |  |  |  |
| DLA_LG6_008530 | LG6 |  |  |  |  |
| DLA_LG6_008535 | LG6 |  |  |  |  |
| DLA_LG6_008540 | LG6 |  |  |  |  |
| DLA_LG6_008550 | LG6 |  |  |  |  |
| DLA_LG6_008555 | LG6 |  |  |  |  |
| DLA_LG6_008570 | LG6 |  |  |  |  |
| DLA_LG6_008590 | LG6 |  |  |  |  |
| DLA_LG6_008600 | LG6 |  |  |  |  |
| DLA_LG6_008610 | LG6 |  |  |  |  |
| DLA_LG6_008620 | LG6 |  |  |  |  |
| DLA_LG6_008630 | LG6 |  |  |  |  |
| DLA_LG6_008640 | LG6 |  |  |  |  |
| DLA_LG6_008645 | LG6 |  |  |  |  |
| DLA_LG6_008660 | LG6 |  |  |  |  |
| DLA_LG6_008670 | LG6 |  |  |  |  |
| DLA_LG6_008680 | LG6 |  |  |  |  |
| DLA_LG6_008710 | LG6 |  |  |  |  |
| DLA_LG6_008720 | LG6 |  |  |  |  |
| DLA_LG6_008730 | LG6 |  |  |  |  |
| DLA_LG6_008740 | LG6 |  |  |  |  |
| DLA_LG6_008760 | LG6 |  |  |  |  |
| DLA_LG6_008780 | LG6 |  |  |  |  |
| DLA_LG6_008790 | LG6 |  |  |  |  |
| DLA_LG6_008800 | LG6 |  |  |  |  |
| DLA_LG6_008810 | LG6 |  |  |  |  |
| DLA_LG6_008820 | LG6 |  |  |  |  |
| DLA_LG6_008830 | LG6 |  |  |  |  |
| DLA_LG6_008840 | LG6 |  |  |  |  |
| DLA_LG6_008860 | LG6 |  |  |  |  |
| DLA_LG6_008870 | LG6 |  |  |  |  |
| DLA_LG6_008880 | LG6 |  |  |  |  |
| DLA_LG6_008890 | LG6 |  |  |  |  |
| DLA_LG6_008900 | LG6 |  |  |  |  |
| DLA_LG6_008920 | LG6 |  |  |  |  |
| DLA_LG6_008930 | LG6 |  |  |  |  |
| DLA_LG6_008940 | LG6 |  |  |  |  |
| DLA_LG6_008950 | LG6 |  |  |  |  |
| DLA_LG6_008960 | LG6 |  |  |  |  |
| DLA_LG6_008970 | LG6 |  |  |  |  |
| DLA_LG6_008980 | LG6 |  |  |  |  |
| DLA_LG6_008990 | LG6 |  |  |  |  |
| DLA_LG6_009000 | LG6 |  |  |  |  |
| DLA_LG6_009010 | LG6 |  |  |  |  |
| DLA_LG6_009020 | LG6 |  |  |  |  |
| DLA_LG6_009030 | LG6 |  |  |  |  |
| DLA_LG6_009040 | LG6 |  |  |  |  |
| DLA_LG6_009060 | LG6 |  |  |  |  |
| DLA_LG6_009070 | LG6 |  |  |  |  |
| DLA_LG6_009080 | LG6 |  |  |  |  |
| DLA_LG6_009095 | LG6 |  |  |  |  |
| DLA_LG6_009100 | LG6 |  |  |  |  |
| DLA_LG6_009120 | LG6 |  |  |  |  |
| DLA_LG6_009130 | LG6 |  |  |  |  |
| DLA_LG6_009140 | LG6 |  |  |  |  |
| DLA_LG6_009150 | LG6 |  |  |  |  |
| DLA_LG6_009160 | LG6 |  |  |  |  |
| DLA_LG6_009170 | LG6 |  |  |  |  |
| DLA_LG6_009180 | LG6 |  |  |  |  |
| DLA_LG6_009185 | LG6 |  |  |  |  |
| DLA_LG6_009200 | LG6 |  |  |  |  |
| DLA_LG6_009205 | LG6 |  |  |  |  |
| DLA_LG6_009210 | LG6 |  |  |  |  |
| DLA_LG6_009220 | LG6 |  |  |  |  |
| DLA_LG6_009230 | LG6 |  |  |  |  |
| DLA_LG6_009245 | LG6 |  |  |  |  |
| DLA_LG6_009250 | LG6 |  |  |  |  |
| DLA_LG6_009260 | LG6 |  |  |  |  |
| DLA_LG6_009270 | LG6 |  |  |  |  |
| DLA_LG6_009280 | LG6 |  |  |  |  |
| DLA_LG7_002140 | LG7 |  |  |  |  |
| DLA_LG7_000560 | LG7 |  |  |  |  |
| DLA_LG7_002750 | LG7 |  |  |  |  |
| DLA_LG7_003480 | LG7 |  |  |  |  |
| DLA_LG7_003950 | LG7 |  |  |  |  |
| DLA_LG7_004250 | LG7 |  |  |  |  |
| DLA_LG7_004570 | LG7 |  |  |  |  |
| DLA_LG7_004860 | LG7 |  |  |  |  |
| DLA_LG7_005210 | LG7 |  |  |  |  |
| DLA_LG7_006400 | LG7 |  |  |  |  |
| DLA_LG7_006850 | LG7 |  |  |  |  |
| DLA_LG7_008030 | LG7 |  |  |  |  |
| DLA_LG7_008950 | LG7 |  |  |  |  |
| DLA_LG7_009370 | LG7 |  |  |  |  |
| DLA_LG7_009560 | LG7 |  |  |  |  |
| DLA_LG7_000010 | LG7 |  |  |  |  |
| DLA_LG7_000110 | LG7 |  |  |  |  |
| DLA_LG7_000530 | LG7 |  |  |  |  |
| DLA_LG7_000740 | LG7 |  |  |  |  |
| DLA_LG7_000810 | LG7 |  |  |  |  |
| DLA_LG7_000840 | LG7 |  |  |  |  |
| DLA_LG7_001080 | LG7 |  |  |  |  |
| DLA_LG7_001110 | LG7 |  |  |  |  |
| DLA_LG7_001150 | LG7 |  |  |  |  |
| DLA_LG7_001190 | LG7 |  |  |  |  |
| DLA_LG7_001200 | LG7 |  |  |  |  |
| DLA_LG7_001460 | LG7 |  |  |  |  |
| DLA_LG7_001480 | LG7 |  |  |  |  |
| DLA_LG7_001740 | LG7 |  |  |  |  |
| DLA_LG7_001980 | LG7 |  |  |  |  |
| DLA_LG7_002470 | LG7 |  |  |  |  |
| DLA_LG7_002530 | LG7 |  |  |  |  |
| DLA_LG7_002600 | LG7 |  |  |  |  |
| DLA_LG7_002910 | LG7 |  |  |  |  |
| DLA_LG7_002970 | LG7 |  |  |  |  |
| DLA_LG7_003090 | LG7 |  |  |  |  |
| DLA_LG7_003210 | LG7 |  |  |  |  |
| DLA_LG7_003320 | LG7 |  |  |  |  |
| DLA_LG7_003360 | LG7 |  |  |  |  |
| DLA_LG7_003770 | LG7 |  |  |  |  |
| DLA_LG7_003820 | LG7 |  |  |  |  |
| DLA_LG7_003920 | LG7 |  |  |  |  |
| DLA_LG7_004210 | LG7 |  |  |  |  |
| DLA_LG7_004240 | LG7 |  |  |  |  |
| DLA_LG7_004270 | LG7 |  |  |  |  |
| DLA_LG7_004360 | LG7 |  |  |  |  |
| DLA_LG7_004540 | LG7 |  |  |  |  |
| DLA_LG7_004710 | LG7 |  |  |  |  |
| DLA_LG7_004770 | LG7 |  |  |  |  |
| DLA_LG7_004820 | LG7 |  |  |  |  |
| DLA_LG7_005020 | LG7 |  |  |  |  |
| DLA_LG7_005050 | LG7 |  |  |  |  |
| DLA_LG7_005360 | LG7 |  |  |  |  |
| DLA_LG7_005400 | LG7 |  |  |  |  |
| DLA_LG7_005500 | LG7 |  |  |  |  |
| DLA_LG7_005540 | LG7 |  |  |  |  |
| DLA_LG7_005570 | LG7 |  |  |  |  |
| DLA_LG7_005790 | LG7 |  |  |  |  |
| DLA_LG7_005930 | LG7 |  |  |  |  |
| DLA_LG7_006130 | LG7 |  |  |  |  |
| DLA_LG7_006290 | LG7 |  |  |  |  |
| DLA_LG7_006560 | LG7 |  |  |  |  |
| DLA_LG7_006780 | LG7 |  |  |  |  |
| DLA_LG7_006905 | LG7 |  |  |  |  |
| DLA_LG7_007030 | LG7 |  |  |  |  |
| DLA_LG7_007060 | LG7 |  |  |  |  |
| DLA_LG7_007100 | LG7 |  |  |  |  |
| DLA_LG7_007150 | LG7 |  |  |  |  |
| DLA_LG7_007215 | LG7 |  |  |  |  |
| DLA_LG7_007230 | LG7 |  |  |  |  |
| DLA_LG7_007390 | LG7 |  |  |  |  |
| DLA_LG7_007490 | LG7 |  |  |  |  |
| DLA_LG7_007630 | LG7 |  |  |  |  |
| DLA_LG7_007660 | LG7 |  |  |  |  |
| DLA_LG7_007690 | LG7 |  |  |  |  |
| DLA_LG7_007780 | LG7 |  |  |  |  |
| DLA_LG7_007870 | LG7 |  |  |  |  |
| DLA_LG7_007930 | LG7 |  |  |  |  |
| DLA_LG7_007990 | LG7 |  |  |  |  |
| DLA_LG7_008110 | LG7 |  |  |  |  |
| DLA_LG7_008170 | LG7 |  |  |  |  |
| DLA_LG7_008250 | LG7 |  |  |  |  |
| DLA_LG7_008300 | LG7 |  |  |  |  |
| DLA_LG7_008310 | LG7 |  |  |  |  |
| DLA_LG7_008380 | LG7 |  |  |  |  |
| DLA_LG7_008690 | LG7 |  |  |  |  |
| DLA_LG7_008750 | LG7 |  |  |  |  |
| DLA_LG7_008780 | LG7 |  |  |  |  |
| DLA_LG7_009040 | LG7 |  |  |  |  |
| DLA_LG7_009620 | LG7 |  |  |  |  |
| DLA_LG7_000020 | LG7 |  |  |  |  |
| DLA_LG7_000030 | LG7 |  |  |  |  |
| DLA_LG7_000040 | LG7 |  |  |  |  |
| DLA_LG7_000050 | LG7 |  |  |  |  |
| DLA_LG7_000060 | LG7 |  |  |  |  |
| DLA_LG7_000065 | LG7 |  |  |  |  |
| DLA_LG7_000070 | LG7 |  |  |  |  |
| DLA_LG7_000080 | LG7 |  |  |  |  |
| DLA_LG7_000090 | LG7 |  |  |  |  |
| DLA_LG7_000120 | LG7 |  |  |  |  |
| DLA_LG7_000130 | LG7 |  |  |  |  |
| DLA_LG7_000140 | LG7 |  |  |  |  |
| DLA_LG7_000150 | LG7 |  |  |  |  |
| DLA_LG7_000160 | LG7 |  |  |  |  |
| DLA_LG7_000170 | LG7 |  |  |  |  |
| DLA_LG7_000175 | LG7 |  |  |  |  |
| DLA_LG7_000190 | LG7 |  |  |  |  |
| DLA_LG7_000195 | LG7 |  |  |  |  |
| DLA_LG7_000200 | LG7 |  |  |  |  |
| DLA_LG7_000210 | LG7 |  |  |  |  |
| DLA_LG7_000220 | LG7 |  |  |  |  |
| DLA_LG7_000230 | LG7 |  |  |  |  |
| DLA_LG7_000240 | LG7 |  |  |  |  |
| DLA_LG7_000250 | LG7 |  |  |  |  |
| DLA_LG7_000265 | LG7 |  |  |  |  |
| DLA_LG7_000270 | LG7 |  |  |  |  |
| DLA_LG7_000280 | LG7 |  |  |  |  |
| DLA_LG7_000290 | LG7 |  |  |  |  |
| DLA_LG7_000300 | LG7 |  |  |  |  |
| DLA_LG7_000310 | LG7 |  |  |  |  |
| DLA_LG7_000320 | LG7 |  |  |  |  |
| DLA_LG7_000330 | LG7 |  |  |  |  |
| DLA_LG7_000340 | LG7 |  |  |  |  |
| DLA_LG7_000360 | LG7 |  |  |  |  |
| DLA_LG7_000370 | LG7 |  |  |  |  |
| DLA_LG7_000380 | LG7 |  |  |  |  |
| DLA_LG7_000390 | LG7 |  |  |  |  |
| DLA_LG7_000400 | LG7 |  |  |  |  |
| DLA_LG7_000410 | LG7 |  |  |  |  |
| DLA_LG7_000430 | LG7 |  |  |  |  |
| DLA_LG7_000440 | LG7 |  |  |  |  |
| DLA_LG7_000450 | LG7 |  |  |  |  |
| DLA_LG7_000460 | LG7 |  |  |  |  |
| DLA_LG7_000470 | LG7 |  |  |  |  |
| DLA_LG7_000480 | LG7 |  |  |  |  |
| DLA_LG7_000490 | LG7 |  |  |  |  |
| DLA_LG7_000500 | LG7 |  |  |  |  |
| DLA_LG7_000510 | LG7 |  |  |  |  |
| DLA_LG7_000520 | LG7 |  |  |  |  |
| DLA_LG7_000540 | LG7 |  |  |  |  |
| DLA_LG7_000550 | LG7 |  |  |  |  |
| DLA_LG7_000570 | LG7 |  |  |  |  |
| DLA_LG7_000580 | LG7 |  |  |  |  |
| DLA_LG7_000590 | LG7 |  |  |  |  |
| DLA_LG7_000600 | LG7 |  |  |  |  |
| DLA_LG7_000610 | LG7 |  |  |  |  |
| DLA_LG7_000620 | LG7 |  |  |  |  |
| DLA_LG7_000630 | LG7 |  |  |  |  |
| DLA_LG7_000640 | LG7 |  |  |  |  |
| DLA_LG7_000650 | LG7 |  |  |  |  |
| DLA_LG7_000660 | LG7 |  |  |  |  |
| DLA_LG7_000670 | LG7 |  |  |  |  |
| DLA_LG7_000680 | LG7 |  |  |  |  |
| DLA_LG7_000685 | LG7 |  |  |  |  |
| DLA_LG7_000690 | LG7 |  |  |  |  |
| DLA_LG7_000700 | LG7 |  |  |  |  |
| DLA_LG7_000720 | LG7 |  |  |  |  |
| DLA_LG7_000730 | LG7 |  |  |  |  |
| DLA_LG7_000745 | LG7 |  |  |  |  |
| DLA_LG7_000750 | LG7 |  |  |  |  |
| DLA_LG7_000760 | LG7 |  |  |  |  |
| DLA_LG7_000770 | LG7 |  |  |  |  |
| DLA_LG7_000780 | LG7 |  |  |  |  |
| DLA_LG7_000790 | LG7 |  |  |  |  |
| DLA_LG7_000800 | LG7 |  |  |  |  |
| DLA_LG7_000805 | LG7 |  |  |  |  |
| DLA_LG7_000820 | LG7 |  |  |  |  |
| DLA_LG7_000825 | LG7 |  |  |  |  |
| DLA_LG7_000830 | LG7 |  |  |  |  |
| DLA_LG7_000845 | LG7 |  |  |  |  |
| DLA_LG7_000850 | LG7 |  |  |  |  |
| DLA_LG7_000860 | LG7 |  |  |  |  |
| DLA_LG7_000870 | LG7 |  |  |  |  |
| DLA_LG7_000880 | LG7 |  |  |  |  |
| DLA_LG7_000890 | LG7 |  |  |  |  |
| DLA_LG7_000900 | LG7 |  |  |  |  |
| DLA_LG7_000910 | LG7 |  |  |  |  |
| DLA_LG7_000920 | LG7 |  |  |  |  |
| DLA_LG7_000930 | LG7 |  |  |  |  |
| DLA_LG7_000935_2 | LG7 |  |  |  |  |
| DLA_LG7_000940 | LG7 |  |  |  |  |
| DLA_LG7_000945 | LG7 |  |  |  |  |
| DLA_LG7_000950 | LG7 |  |  |  |  |
| DLA_LG7_000960 | LG7 |  |  |  |  |
| DLA_LG7_000970 | LG7 |  |  |  |  |
| DLA_LG7_000980 | LG7 |  |  |  |  |
| DLA_LG7_000990 | LG7 |  |  |  |  |
| DLA_LG7_001000 | LG7 |  |  |  |  |
| DLA_LG7_001010 | LG7 |  |  |  |  |
| DLA_LG7_001020 | LG7 |  |  |  |  |
| DLA_LG7_001030 | LG7 |  |  |  |  |
| DLA_LG7_001040 | LG7 |  |  |  |  |
| DLA_LG7_001050 | LG7 |  |  |  |  |
| DLA_LG7_001060 | LG7 |  |  |  |  |
| DLA_LG7_001070 | LG7 |  |  |  |  |
| DLA_LG7_001075 | LG7 |  |  |  |  |
| DLA_LG7_001090 | LG7 |  |  |  |  |
| DLA_LG7_001095 | LG7 |  |  |  |  |
| DLA_LG7_001100 | LG7 |  |  |  |  |
| DLA_LG7_001120 | LG7 |  |  |  |  |
| DLA_LG7_001130 | LG7 |  |  |  |  |
| DLA_LG7_001140 | LG7 |  |  |  |  |
| DLA_LG7_001160 | LG7 |  |  |  |  |
| DLA_LG7_001170 | LG7 |  |  |  |  |
| DLA_LG7_001175 | LG7 |  |  |  |  |
| DLA_LG7_001180 | LG7 |  |  |  |  |
| DLA_LG7_001210 | LG7 |  |  |  |  |
| DLA_LG7_001220 | LG7 |  |  |  |  |
| DLA_LG7_001230 | LG7 |  |  |  |  |
| DLA_LG7_001240 | LG7 |  |  |  |  |
| DLA_LG7_001250 | LG7 |  |  |  |  |
| DLA_LG7_001270 | LG7 |  |  |  |  |
| DLA_LG7_001280 | LG7 |  |  |  |  |
| DLA_LG7_001290 | LG7 |  |  |  |  |
| DLA_LG7_001300 | LG7 |  |  |  |  |
| DLA_LG7_001310 | LG7 |  |  |  |  |
| DLA_LG7_001320 | LG7 |  |  |  |  |
| DLA_LG7_001330 | LG7 |  |  |  |  |
| DLA_LG7_001350 | LG7 |  |  |  |  |
| DLA_LG7_001360 | LG7 |  |  |  |  |
| DLA_LG7_001370 | LG7 |  |  |  |  |
| DLA_LG7_001375 | LG7 |  |  |  |  |
| DLA_LG7_001380 | LG7 |  |  |  |  |
| DLA_LG7_001390 | LG7 |  |  |  |  |
| DLA_LG7_001400 | LG7 |  |  |  |  |
| DLA_LG7_001410 | LG7 |  |  |  |  |
| DLA_LG7_001420 | LG7 |  |  |  |  |
| DLA_LG7_001430 | LG7 |  |  |  |  |
| DLA_LG7_001440 | LG7 |  |  |  |  |
| DLA_LG7_001450 | LG7 |  |  |  |  |
| DLA_LG7_001470 | LG7 |  |  |  |  |
| DLA_LG7_001490 | LG7 |  |  |  |  |
| DLA_LG7_001500 | LG7 |  |  |  |  |
| DLA_LG7_001510 | LG7 |  |  |  |  |
| DLA_LG7_001520 | LG7 |  |  |  |  |
| DLA_LG7_001530 | LG7 |  |  |  |  |
| DLA_LG7_001540 | LG7 |  |  |  |  |
| DLA_LG7_001550 | LG7 |  |  |  |  |
| DLA_LG7_001560 | LG7 |  |  |  |  |
| DLA_LG7_001570 | LG7 |  |  |  |  |
| DLA_LG7_001580 | LG7 |  |  |  |  |
| DLA_LG7_001590 | LG7 |  |  |  |  |
| DLA_LG7_001600 | LG7 |  |  |  |  |
| DLA_LG7_001610 | LG7 |  |  |  |  |
| DLA_LG7_001620 | LG7 |  |  |  |  |
| DLA_LG7_001640 | LG7 |  |  |  |  |
| DLA_LG7_001650 | LG7 |  |  |  |  |
| DLA_LG7_001660 | LG7 |  |  |  |  |
| DLA_LG7_001670 | LG7 |  |  |  |  |
| DLA_LG7_001680 | LG7 |  |  |  |  |
| DLA_LG7_001690 | LG7 |  |  |  |  |
| DLA_LG7_001695 | LG7 |  |  |  |  |
| DLA_LG7_001700 | LG7 |  |  |  |  |
| DLA_LG7_001710 | LG7 |  |  |  |  |
| DLA_LG7_001720 | LG7 |  |  |  |  |
| DLA_LG7_001730 | LG7 |  |  |  |  |
| DLA_LG7_001750 | LG7 |  |  |  |  |
| DLA_LG7_001760 | LG7 |  |  |  |  |
| DLA_LG7_001770 | LG7 |  |  |  |  |
| DLA_LG7_001780 | LG7 |  |  |  |  |
| DLA_LG7_001785 | LG7 |  |  |  |  |
| DLA_LG7_001790 | LG7 |  |  |  |  |
| DLA_LG7_001800 | LG7 |  |  |  |  |
| DLA_LG7_001805 | LG7 |  |  |  |  |
| DLA_LG7_001810 | LG7 |  |  |  |  |
| DLA_LG7_001820 | LG7 |  |  |  |  |
| DLA_LG7_001830 | LG7 |  |  |  |  |
| DLA_LG7_001840 | LG7 |  |  |  |  |
| DLA_LG7_001850 | LG7 |  |  |  |  |
| DLA_LG7_001860 | LG7 |  |  |  |  |
| DLA_LG7_001870 | LG7 |  |  |  |  |
| DLA_LG7_001875 | LG7 |  |  |  |  |
| DLA_LG7_001880 | LG7 |  |  |  |  |
| DLA_LG7_001900 | LG7 |  |  |  |  |
| DLA_LG7_001920 | LG7 |  |  |  |  |
| DLA_LG7_001930 | LG7 |  |  |  |  |
| DLA_LG7_001940 | LG7 |  |  |  |  |
| DLA_LG7_001950 | LG7 |  |  |  |  |
| DLA_LG7_001960 | LG7 |  |  |  |  |
| DLA_LG7_001970 | LG7 |  |  |  |  |
| DLA_LG7_001990 | LG7 |  |  |  |  |
| DLA_LG7_002000 | LG7 |  |  |  |  |
| DLA_LG7_002010 | LG7 |  |  |  |  |
| DLA_LG7_002020 | LG7 |  |  |  |  |
| DLA_LG7_002030 | LG7 |  |  |  |  |
| DLA_LG7_002040 | LG7 |  |  |  |  |
| DLA_LG7_002050 | LG7 |  |  |  |  |
| DLA_LG7_002060 | LG7 |  |  |  |  |
| DLA_LG7_002070 | LG7 |  |  |  |  |
| DLA_LG7_002080 | LG7 |  |  |  |  |
| DLA_LG7_002090 | LG7 |  |  |  |  |
| DLA_LG7_002110 | LG7 |  |  |  |  |
| DLA_LG7_002120 | LG7 |  |  |  |  |
| DLA_LG7_002130 | LG7 |  |  |  |  |
| DLA_LG7_002150 | LG7 |  |  |  |  |
| DLA_LG7_002160 | LG7 |  |  |  |  |
| DLA_LG7_002190 | LG7 |  |  |  |  |
| DLA_LG7_002200 | LG7 |  |  |  |  |
| DLA_LG7_002220 | LG7 |  |  |  |  |
| DLA_LG7_002230 | LG7 |  |  |  |  |
| DLA_LG7_002240 | LG7 |  |  |  |  |
| DLA_LG7_002250 | LG7 |  |  |  |  |
| DLA_LG7_002260 | LG7 |  |  |  |  |
| DLA_LG7_002270 | LG7 |  |  |  |  |
| DLA_LG7_002280 | LG7 |  |  |  |  |
| DLA_LG7_002290 | LG7 |  |  |  |  |
| DLA_LG7_002300 | LG7 |  |  |  |  |
| DLA_LG7_002310 | LG7 |  |  |  |  |
| DLA_LG7_002320 | LG7 |  |  |  |  |
| DLA_LG7_002360 | LG7 |  |  |  |  |
| DLA_LG7_002370 | LG7 |  |  |  |  |
| DLA_LG7_002380 | LG7 |  |  |  |  |
| DLA_LG7_002390 | LG7 |  |  |  |  |
| DLA_LG7_002400 | LG7 |  |  |  |  |
| DLA_LG7_002410 | LG7 |  |  |  |  |
| DLA_LG7_002420 | LG7 |  |  |  |  |
| DLA_LG7_002430 | LG7 |  |  |  |  |
| DLA_LG7_002440 | LG7 |  |  |  |  |
| DLA_LG7_002450 | LG7 |  |  |  |  |
| DLA_LG7_002460 | LG7 |  |  |  |  |
| DLA_LG7_002480 | LG7 |  |  |  |  |
| DLA_LG7_002490 | LG7 |  |  |  |  |
| DLA_LG7_002500 | LG7 |  |  |  |  |
| DLA_LG7_002510 | LG7 |  |  |  |  |
| DLA_LG7_002520 | LG7 |  |  |  |  |
| DLA_LG7_002540 | LG7 |  |  |  |  |
| DLA_LG7_002550 | LG7 |  |  |  |  |
| DLA_LG7_002560 | LG7 |  |  |  |  |
| DLA_LG7_002570 | LG7 |  |  |  |  |
| DLA_LG7_002580 | LG7 |  |  |  |  |
| DLA_LG7_002590 | LG7 |  |  |  |  |
| DLA_LG7_002610 | LG7 |  |  |  |  |
| DLA_LG7_002620 | LG7 |  |  |  |  |
| DLA_LG7_002630 | LG7 |  |  |  |  |
| DLA_LG7_002640 | LG7 |  |  |  |  |
| DLA_LG7_002650 | LG7 |  |  |  |  |
| DLA_LG7_002670 | LG7 |  |  |  |  |
| DLA_LG7_002680 | LG7 |  |  |  |  |
| DLA_LG7_002690 | LG7 |  |  |  |  |
| DLA_LG7_002700 | LG7 |  |  |  |  |
| DLA_LG7_002710 | LG7 |  |  |  |  |
| DLA_LG7_002720 | LG7 |  |  |  |  |
| DLA_LG7_002730 | LG7 |  |  |  |  |
| DLA_LG7_002740 | LG7 |  |  |  |  |
| DLA_LG7_002760 | LG7 |  |  |  |  |
| DLA_LG7_002770 | LG7 |  |  |  |  |
| DLA_LG7_002780 | LG7 |  |  |  |  |
| DLA_LG7_002790 | LG7 |  |  |  |  |
| DLA_LG7_002800 | LG7 |  |  |  |  |
| DLA_LG7_002810 | LG7 |  |  |  |  |
| DLA_LG7_002820 | LG7 |  |  |  |  |
| DLA_LG7_002830 | LG7 |  |  |  |  |
| DLA_LG7_002840 | LG7 |  |  |  |  |
| DLA_LG7_002845 | LG7 |  |  |  |  |
| DLA_LG7_002850 | LG7 |  |  |  |  |
| DLA_LG7_002860 | LG7 |  |  |  |  |
| DLA_LG7_002870 | LG7 |  |  |  |  |
| DLA_LG7_002880 | LG7 |  |  |  |  |
| DLA_LG7_002890 | LG7 |  |  |  |  |
| DLA_LG7_002900 | LG7 |  |  |  |  |
| DLA_LG7_002920 | LG7 |  |  |  |  |
| DLA_LG7_002930 | LG7 |  |  |  |  |
| DLA_LG7_002940 | LG7 |  |  |  |  |
| DLA_LG7_002950 | LG7 |  |  |  |  |
| DLA_LG7_002960 | LG7 |  |  |  |  |
| DLA_LG7_002980 | LG7 |  |  |  |  |
| DLA_LG7_002990 | LG7 |  |  |  |  |
| DLA_LG7_003000 | LG7 |  |  |  |  |
| DLA_LG7_003010 | LG7 |  |  |  |  |
| DLA_LG7_003020 | LG7 |  |  |  |  |
| DLA_LG7_003030 | LG7 |  |  |  |  |
| DLA_LG7_003040 | LG7 |  |  |  |  |
| DLA_LG7_003050 | LG7 |  |  |  |  |
| DLA_LG7_003060 | LG7 |  |  |  |  |
| DLA_LG7_003070 | LG7 |  |  |  |  |
| DLA_LG7_003080 | LG7 |  |  |  |  |
| DLA_LG7_003100 | LG7 |  |  |  |  |
| DLA_LG7_003110 | LG7 |  |  |  |  |
| DLA_LG7_003120 | LG7 |  |  |  |  |
| DLA_LG7_003130 | LG7 |  |  |  |  |
| DLA_LG7_003140 | LG7 |  |  |  |  |
| DLA_LG7_003150 | LG7 |  |  |  |  |
| DLA_LG7_003160 | LG7 |  |  |  |  |
| DLA_LG7_003170 | LG7 |  |  |  |  |
| DLA_LG7_003175 | LG7 |  |  |  |  |
| DLA_LG7_003180 | LG7 |  |  |  |  |
| DLA_LG7_003190 | LG7 |  |  |  |  |
| DLA_LG7_003220 | LG7 |  |  |  |  |
| DLA_LG7_003225 | LG7 |  |  |  |  |
| DLA_LG7_003230 | LG7 |  |  |  |  |
| DLA_LG7_003240 | LG7 |  |  |  |  |
| DLA_LG7_003250 | LG7 |  |  |  |  |
| DLA_LG7_003260 | LG7 |  |  |  |  |
| DLA_LG7_003270 | LG7 |  |  |  |  |
| DLA_LG7_003280 | LG7 |  |  |  |  |
| DLA_LG7_003290 | LG7 |  |  |  |  |
| DLA_LG7_003300 | LG7 |  |  |  |  |
| DLA_LG7_003310 | LG7 |  |  |  |  |
| DLA_LG7_003340 | LG7 |  |  |  |  |
| DLA_LG7_003350 | LG7 |  |  |  |  |
| DLA_LG7_003370 | LG7 |  |  |  |  |
| DLA_LG7_003380 | LG7 |  |  |  |  |
| DLA_LG7_003390 | LG7 |  |  |  |  |
| DLA_LG7_003400 | LG7 |  |  |  |  |
| DLA_LG7_003405 | LG7 |  |  |  |  |
| DLA_LG7_003410 | LG7 |  |  |  |  |
| DLA_LG7_003420 | LG7 |  |  |  |  |
| DLA_LG7_003430 | LG7 |  |  |  |  |
| DLA_LG7_003440 | LG7 |  |  |  |  |
| DLA_LG7_003450 | LG7 |  |  |  |  |
| DLA_LG7_003460 | LG7 |  |  |  |  |
| DLA_LG7_003470 | LG7 |  |  |  |  |
| DLA_LG7_003490 | LG7 |  |  |  |  |
| DLA_LG7_003500 | LG7 |  |  |  |  |
| DLA_LG7_003510 | LG7 |  |  |  |  |
| DLA_LG7_003515 | LG7 |  |  |  |  |
| DLA_LG7_003540 | LG7 |  |  |  |  |
| DLA_LG7_003550 | LG7 |  |  |  |  |
| DLA_LG7_003560 | LG7 |  |  |  |  |
| DLA_LG7_003570 | LG7 |  |  |  |  |
| DLA_LG7_003580 | LG7 |  |  |  |  |
| DLA_LG7_003590 | LG7 |  |  |  |  |
| DLA_LG7_003595 | LG7 |  |  |  |  |
| DLA_LG7_003600 | LG7 |  |  |  |  |
| DLA_LG7_003610 | LG7 |  |  |  |  |
| DLA_LG7_003620 | LG7 |  |  |  |  |
| DLA_LG7_003630 | LG7 |  |  |  |  |
| DLA_LG7_003640 | LG7 |  |  |  |  |
| DLA_LG7_003650 | LG7 |  |  |  |  |
| DLA_LG7_003660 | LG7 |  |  |  |  |
| DLA_LG7_003670 | LG7 |  |  |  |  |
| DLA_LG7_003680 | LG7 |  |  |  |  |
| DLA_LG7_003690 | LG7 |  |  |  |  |
| DLA_LG7_003700 | LG7 |  |  |  |  |
| DLA_LG7_003710 | LG7 |  |  |  |  |
| DLA_LG7_003720 | LG7 |  |  |  |  |
| DLA_LG7_003730 | LG7 |  |  |  |  |
| DLA_LG7_003740 | LG7 |  |  |  |  |
| DLA_LG7_003750 | LG7 |  |  |  |  |
| DLA_LG7_003760 | LG7 |  |  |  |  |
| DLA_LG7_003780 | LG7 |  |  |  |  |
| DLA_LG7_003790 | LG7 |  |  |  |  |
| DLA_LG7_003800 | LG7 |  |  |  |  |
| DLA_LG7_003810 | LG7 |  |  |  |  |
| DLA_LG7_003830 | LG7 |  |  |  |  |
| DLA_LG7_003835 | LG7 |  |  |  |  |
| DLA_LG7_003840 | LG7 |  |  |  |  |
| DLA_LG7_003850 | LG7 |  |  |  |  |
| DLA_LG7_003860 | LG7 |  |  |  |  |
| DLA_LG7_003865 | LG7 |  |  |  |  |
| DLA_LG7_003870 | LG7 |  |  |  |  |
| DLA_LG7_003880 | LG7 |  |  |  |  |
| DLA_LG7_003890 | LG7 |  |  |  |  |
| DLA_LG7_003900 | LG7 |  |  |  |  |
| DLA_LG7_003910 | LG7 |  |  |  |  |
| DLA_LG7_003930 | LG7 |  |  |  |  |
| DLA_LG7_003940 | LG7 |  |  |  |  |
| DLA_LG7_003960 | LG7 |  |  |  |  |
| DLA_LG7_003970 | LG7 |  |  |  |  |
| DLA_LG7_003975 | LG7 |  |  |  |  |
| DLA_LG7_003975_2 | LG7 |  |  |  |  |
| DLA_LG7_003980 | LG7 |  |  |  |  |
| DLA_LG7_004000 | LG7 |  |  |  |  |
| DLA_LG7_004010 | LG7 |  |  |  |  |
| DLA_LG7_004020 | LG7 |  |  |  |  |
| DLA_LG7_004030 | LG7 |  |  |  |  |
| DLA_LG7_004040 | LG7 |  |  |  |  |
| DLA_LG7_004060 | LG7 |  |  |  |  |
| DLA_LG7_004070 | LG7 |  |  |  |  |
| DLA_LG7_004090 | LG7 |  |  |  |  |
| DLA_LG7_004110 | LG7 |  |  |  |  |
| DLA_LG7_004120 | LG7 |  |  |  |  |
| DLA_LG7_004140 | LG7 |  |  |  |  |
| DLA_LG7_004150 | LG7 |  |  |  |  |
| DLA_LG7_004160 | LG7 |  |  |  |  |
| DLA_LG7_004165 | LG7 |  |  |  |  |
| DLA_LG7_004170 | LG7 |  |  |  |  |
| DLA_LG7_004180 | LG7 |  |  |  |  |
| DLA_LG7_004190 | LG7 |  |  |  |  |
| DLA_LG7_004200 | LG7 |  |  |  |  |
| DLA_LG7_004220 | LG7 |  |  |  |  |
| DLA_LG7_004225 | LG7 |  |  |  |  |
| DLA_LG7_004230 | LG7 |  |  |  |  |
| DLA_LG7_004235 | LG7 |  |  |  |  |
| DLA_LG7_004260 | LG7 |  |  |  |  |
| DLA_LG7_004290 | LG7 |  |  |  |  |
| DLA_LG7_004300 | LG7 |  |  |  |  |
| DLA_LG7_004310 | LG7 |  |  |  |  |
| DLA_LG7_004320 | LG7 |  |  |  |  |
| DLA_LG7_004330 | LG7 |  |  |  |  |
| DLA_LG7_004340 | LG7 |  |  |  |  |
| DLA_LG7_004350 | LG7 |  |  |  |  |
| DLA_LG7_004370 | LG7 |  |  |  |  |
| DLA_LG7_004375 | LG7 |  |  |  |  |
| DLA_LG7_004380 | LG7 |  |  |  |  |
| DLA_LG7_004390 | LG7 |  |  |  |  |
| DLA_LG7_004395 | LG7 |  |  |  |  |
| DLA_LG7_004400 | LG7 |  |  |  |  |
| DLA_LG7_004410 | LG7 |  |  |  |  |
| DLA_LG7_004420 | LG7 |  |  |  |  |
| DLA_LG7_004430 | LG7 |  |  |  |  |
| DLA_LG7_004440 | LG7 |  |  |  |  |
| DLA_LG7_004445 | LG7 |  |  |  |  |
| DLA_LG7_004460 | LG7 |  |  |  |  |
| DLA_LG7_004470 | LG7 |  |  |  |  |
| DLA_LG7_004480 | LG7 |  |  |  |  |
| DLA_LG7_004490 | LG7 |  |  |  |  |
| DLA_LG7_004495 | LG7 |  |  |  |  |
| DLA_LG7_004500 | LG7 |  |  |  |  |
| DLA_LG7_004520 | LG7 |  |  |  |  |
| DLA_LG7_004530 | LG7 |  |  |  |  |
| DLA_LG7_004550 | LG7 |  |  |  |  |
| DLA_LG7_004560 | LG7 |  |  |  |  |
| DLA_LG7_004580 | LG7 |  |  |  |  |
| DLA_LG7_004590 | LG7 |  |  |  |  |
| DLA_LG7_004600 | LG7 |  |  |  |  |
| DLA_LG7_004610 | LG7 |  |  |  |  |
| DLA_LG7_004620 | LG7 |  |  |  |  |
| DLA_LG7_004630 | LG7 |  |  |  |  |
| DLA_LG7_004650 | LG7 |  |  |  |  |
| DLA_LG7_004655 | LG7 |  |  |  |  |
| DLA_LG7_004660 | LG7 |  |  |  |  |
| DLA_LG7_004670 | LG7 |  |  |  |  |
| DLA_LG7_004680 | LG7 |  |  |  |  |
| DLA_LG7_004690 | LG7 |  |  |  |  |
| DLA_LG7_004705 | LG7 |  |  |  |  |
| DLA_LG7_004705_2 | LG7 |  |  |  |  |
| DLA_LG7_004730 | LG7 |  |  |  |  |
| DLA_LG7_004740 | LG7 |  |  |  |  |
| DLA_LG7_004750 | LG7 |  |  |  |  |
| DLA_LG7_004760 | LG7 |  |  |  |  |
| DLA_LG7_004765 | LG7 |  |  |  |  |
| DLA_LG7_004780 | LG7 |  |  |  |  |
| DLA_LG7_004790 | LG7 |  |  |  |  |
| DLA_LG7_004810 | LG7 |  |  |  |  |
| DLA_LG7_004825 | LG7 |  |  |  |  |
| DLA_LG7_004840 | LG7 |  |  |  |  |
| DLA_LG7_004870 | LG7 |  |  |  |  |
| DLA_LG7_004880 | LG7 |  |  |  |  |
| DLA_LG7_004890 | LG7 |  |  |  |  |
| DLA_LG7_004900 | LG7 |  |  |  |  |
| DLA_LG7_004920 | LG7 |  |  |  |  |
| DLA_LG7_004930 | LG7 |  |  |  |  |
| DLA_LG7_004940 | LG7 |  |  |  |  |
| DLA_LG7_004950 | LG7 |  |  |  |  |
| DLA_LG7_004960 | LG7 |  |  |  |  |
| DLA_LG7_004970 | LG7 |  |  |  |  |
| DLA_LG7_004980 | LG7 |  |  |  |  |
| DLA_LG7_004985 | LG7 |  |  |  |  |
| DLA_LG7_004985_2 | LG7 |  |  |  |  |
| DLA_LG7_004985_4 | LG7 |  |  |  |  |
| DLA_LG7_004985_5 | LG7 |  |  |  |  |
| DLA_LG7_004990 | LG7 |  |  |  |  |
| DLA_LG7_005000 | LG7 |  |  |  |  |
| DLA_LG7_005005 | LG7 |  |  |  |  |
| DLA_LG7_005010 | LG7 |  |  |  |  |
| DLA_LG7_005030 | LG7 |  |  |  |  |
| DLA_LG7_005040 | LG7 |  |  |  |  |
| DLA_LG7_005045 | LG7 |  |  |  |  |
| DLA_LG7_005055 | LG7 |  |  |  |  |
| DLA_LG7_005060 | LG7 |  |  |  |  |
| DLA_LG7_005070 | LG7 |  |  |  |  |
| DLA_LG7_005080 | LG7 |  |  |  |  |
| DLA_LG7_005090 | LG7 |  |  |  |  |
| DLA_LG7_005100 | LG7 |  |  |  |  |
| DLA_LG7_005105 | LG7 |  |  |  |  |
| DLA_LG7_005110 | LG7 |  |  |  |  |
| DLA_LG7_005120 | LG7 |  |  |  |  |
| DLA_LG7_005140 | LG7 |  |  |  |  |
| DLA_LG7_005150 | LG7 |  |  |  |  |
| DLA_LG7_005160 | LG7 |  |  |  |  |
| DLA_LG7_005170 | LG7 |  |  |  |  |
| DLA_LG7_005180 | LG7 |  |  |  |  |
| DLA_LG7_005190 | LG7 |  |  |  |  |
| DLA_LG7_005200 | LG7 |  |  |  |  |
| DLA_LG7_005220 | LG7 |  |  |  |  |
| DLA_LG7_005230 | LG7 |  |  |  |  |
| DLA_LG7_005240 | LG7 |  |  |  |  |
| DLA_LG7_005250 | LG7 |  |  |  |  |
| DLA_LG7_005260 | LG7 |  |  |  |  |
| DLA_LG7_005270 | LG7 |  |  |  |  |
| DLA_LG7_005280 | LG7 |  |  |  |  |
| DLA_LG7_005290 | LG7 |  |  |  |  |
| DLA_LG7_005300 | LG7 |  |  |  |  |
| DLA_LG7_005310 | LG7 |  |  |  |  |
| DLA_LG7_005330 | LG7 |  |  |  |  |
| DLA_LG7_005340 | LG7 |  |  |  |  |
| DLA_LG7_005350 | LG7 |  |  |  |  |
| DLA_LG7_005365 | LG7 |  |  |  |  |
| DLA_LG7_005370 | LG7 |  |  |  |  |
| DLA_LG7_005380 | LG7 |  |  |  |  |
| DLA_LG7_005390 | LG7 |  |  |  |  |
| DLA_LG7_005410 | LG7 |  |  |  |  |
| DLA_LG7_005415 | LG7 |  |  |  |  |
| DLA_LG7_005420 | LG7 |  |  |  |  |
| DLA_LG7_005430 | LG7 |  |  |  |  |
| DLA_LG7_005440 | LG7 |  |  |  |  |
| DLA_LG7_005450 | LG7 |  |  |  |  |
| DLA_LG7_005460 | LG7 |  |  |  |  |
| DLA_LG7_005465 | LG7 |  |  |  |  |
| DLA_LG7_005470 | LG7 |  |  |  |  |
| DLA_LG7_005480 | LG7 |  |  |  |  |
| DLA_LG7_005490 | LG7 |  |  |  |  |
| DLA_LG7_005505 | LG7 |  |  |  |  |
| DLA_LG7_005510 | LG7 |  |  |  |  |
| DLA_LG7_005520 | LG7 |  |  |  |  |
| DLA_LG7_005530 | LG7 |  |  |  |  |
| DLA_LG7_005550 | LG7 |  |  |  |  |
| DLA_LG7_005560 | LG7 |  |  |  |  |
| DLA_LG7_005580 | LG7 |  |  |  |  |
| DLA_LG7_005600 | LG7 |  |  |  |  |
| DLA_LG7_005610 | LG7 |  |  |  |  |
| DLA_LG7_005620 | LG7 |  |  |  |  |
| DLA_LG7_005630 | LG7 |  |  |  |  |
| DLA_LG7_005635 | LG7 |  |  |  |  |
| DLA_LG7_005640 | LG7 |  |  |  |  |
| DLA_LG7_005650 | LG7 |  |  |  |  |
| DLA_LG7_005660 | LG7 |  |  |  |  |
| DLA_LG7_005670 | LG7 |  |  |  |  |
| DLA_LG7_005680 | LG7 |  |  |  |  |
| DLA_LG7_005690 | LG7 |  |  |  |  |
| DLA_LG7_005700 | LG7 |  |  |  |  |
| DLA_LG7_005710 | LG7 |  |  |  |  |
| DLA_LG7_005720 | LG7 |  |  |  |  |
| DLA_LG7_005725 | LG7 |  |  |  |  |
| DLA_LG7_005730 | LG7 |  |  |  |  |
| DLA_LG7_005740 | LG7 |  |  |  |  |
| DLA_LG7_005750 | LG7 |  |  |  |  |
| DLA_LG7_005760 | LG7 |  |  |  |  |
| DLA_LG7_005770 | LG7 |  |  |  |  |
| DLA_LG7_005780 | LG7 |  |  |  |  |
| DLA_LG7_005800 | LG7 |  |  |  |  |
| DLA_LG7_005810 | LG7 |  |  |  |  |
| DLA_LG7_005820 | LG7 |  |  |  |  |
| DLA_LG7_005830 | LG7 |  |  |  |  |
| DLA_LG7_005840 | LG7 |  |  |  |  |
| DLA_LG7_005850 | LG7 |  |  |  |  |
| DLA_LG7_005860 | LG7 |  |  |  |  |
| DLA_LG7_005870 | LG7 |  |  |  |  |
| DLA_LG7_005880 | LG7 |  |  |  |  |
| DLA_LG7_005890 | LG7 |  |  |  |  |
| DLA_LG7_005900 | LG7 |  |  |  |  |
| DLA_LG7_005910 | LG7 |  |  |  |  |
| DLA_LG7_005920 | LG7 |  |  |  |  |
| DLA_LG7_005940 | LG7 |  |  |  |  |
| DLA_LG7_005950 | LG7 |  |  |  |  |
| DLA_LG7_005960 | LG7 |  |  |  |  |
| DLA_LG7_005965 | LG7 |  |  |  |  |
| DLA_LG7_005970 | LG7 |  |  |  |  |
| DLA_LG7_005980 | LG7 |  |  |  |  |
| DLA_LG7_005990 | LG7 |  |  |  |  |
| DLA_LG7_006010 | LG7 |  |  |  |  |
| DLA_LG7_006020 | LG7 |  |  |  |  |
| DLA_LG7_006040 | LG7 |  |  |  |  |
| DLA_LG7_006050 | LG7 |  |  |  |  |
| DLA_LG7_006060 | LG7 |  |  |  |  |
| DLA_LG7_006070 | LG7 |  |  |  |  |
| DLA_LG7_006080 | LG7 |  |  |  |  |
| DLA_LG7_006090 | LG7 |  |  |  |  |
| DLA_LG7_006100 | LG7 |  |  |  |  |
| DLA_LG7_006110 | LG7 |  |  |  |  |
| DLA_LG7_006120 | LG7 |  |  |  |  |
| DLA_LG7_006140 | LG7 |  |  |  |  |
| DLA_LG7_006150 | LG7 |  |  |  |  |
| DLA_LG7_006160 | LG7 |  |  |  |  |
| DLA_LG7_006170 | LG7 |  |  |  |  |
| DLA_LG7_006180 | LG7 |  |  |  |  |
| DLA_LG7_006190 | LG7 |  |  |  |  |
| DLA_LG7_006200 | LG7 |  |  |  |  |
| DLA_LG7_006210 | LG7 |  |  |  |  |
| DLA_LG7_006220 | LG7 |  |  |  |  |
| DLA_LG7_006230 | LG7 |  |  |  |  |
| DLA_LG7_006240 | LG7 |  |  |  |  |
| DLA_LG7_006250 | LG7 |  |  |  |  |
| DLA_LG7_006260 | LG7 |  |  |  |  |
| DLA_LG7_006270 | LG7 |  |  |  |  |
| DLA_LG7_006280 | LG7 |  |  |  |  |
| DLA_LG7_006285 | LG7 |  |  |  |  |
| DLA_LG7_006285_2 | LG7 |  |  |  |  |
| DLA_LG7_006300 | LG7 |  |  |  |  |
| DLA_LG7_006310 | LG7 |  |  |  |  |
| DLA_LG7_006320 | LG7 |  |  |  |  |
| DLA_LG7_006330 | LG7 |  |  |  |  |
| DLA_LG7_006350 | LG7 |  |  |  |  |
| DLA_LG7_006360 | LG7 |  |  |  |  |
| DLA_LG7_006370 | LG7 |  |  |  |  |
| DLA_LG7_006420 | LG7 |  |  |  |  |
| DLA_LG7_006430 | LG7 |  |  |  |  |
| DLA_LG7_006440 | LG7 |  |  |  |  |
| DLA_LG7_006450 | LG7 |  |  |  |  |
| DLA_LG7_006460 | LG7 |  |  |  |  |
| DLA_LG7_006470 | LG7 |  |  |  |  |
| DLA_LG7_006475 | LG7 |  |  |  |  |
| DLA_LG7_006475_2 | LG7 |  |  |  |  |
| DLA_LG7_006475_3 | LG7 |  |  |  |  |
| DLA_LG7_006480 | LG7 |  |  |  |  |
| DLA_LG7_006490 | LG7 |  |  |  |  |
| DLA_LG7_006500 | LG7 |  |  |  |  |
| DLA_LG7_006510 | LG7 |  |  |  |  |
| DLA_LG7_006520 | LG7 |  |  |  |  |
| DLA_LG7_006530 | LG7 |  |  |  |  |
| DLA_LG7_006540 | LG7 |  |  |  |  |
| DLA_LG7_006550 | LG7 |  |  |  |  |
| DLA_LG7_006570 | LG7 |  |  |  |  |
| DLA_LG7_006575 | LG7 |  |  |  |  |
| DLA_LG7_006590 | LG7 |  |  |  |  |
| DLA_LG7_006600 | LG7 |  |  |  |  |
| DLA_LG7_006620 | LG7 |  |  |  |  |
| DLA_LG7_006630 | LG7 |  |  |  |  |
| DLA_LG7_006635 | LG7 |  |  |  |  |
| DLA_LG7_006650 | LG7 |  |  |  |  |
| DLA_LG7_006660 | LG7 |  |  |  |  |
| DLA_LG7_006670 | LG7 |  |  |  |  |
| DLA_LG7_006680 | LG7 |  |  |  |  |
| DLA_LG7_006690 | LG7 |  |  |  |  |
| DLA_LG7_006700 | LG7 |  |  |  |  |
| DLA_LG7_006710 | LG7 |  |  |  |  |
| DLA_LG7_006720 | LG7 |  |  |  |  |
| DLA_LG7_006730 | LG7 |  |  |  |  |
| DLA_LG7_006740 | LG7 |  |  |  |  |
| DLA_LG7_006750 | LG7 |  |  |  |  |
| DLA_LG7_006770 | LG7 |  |  |  |  |
| DLA_LG7_006790 | LG7 |  |  |  |  |
| DLA_LG7_006800 | LG7 |  |  |  |  |
| DLA_LG7_006810 | LG7 |  |  |  |  |
| DLA_LG7_006820 | LG7 |  |  |  |  |
| DLA_LG7_006830 | LG7 |  |  |  |  |
| DLA_LG7_006840 | LG7 |  |  |  |  |
| DLA_LG7_006860 | LG7 |  |  |  |  |
| DLA_LG7_006870 | LG7 |  |  |  |  |
| DLA_LG7_006880 | LG7 |  |  |  |  |
| DLA_LG7_006890 | LG7 |  |  |  |  |
| DLA_LG7_006900 | LG7 |  |  |  |  |
| DLA_LG7_006920 | LG7 |  |  |  |  |
| DLA_LG7_006930 | LG7 |  |  |  |  |
| DLA_LG7_006940 | LG7 |  |  |  |  |
| DLA_LG7_006970 | LG7 |  |  |  |  |
| DLA_LG7_006975 | LG7 |  |  |  |  |
| DLA_LG7_006980 | LG7 |  |  |  |  |
| DLA_LG7_006990 | LG7 |  |  |  |  |
| DLA_LG7_007000 | LG7 |  |  |  |  |
| DLA_LG7_007010 | LG7 |  |  |  |  |
| DLA_LG7_007020 | LG7 |  |  |  |  |
| DLA_LG7_007040 | LG7 |  |  |  |  |
| DLA_LG7_007045 | LG7 |  |  |  |  |
| DLA_LG7_007050 | LG7 |  |  |  |  |
| DLA_LG7_007080 | LG7 |  |  |  |  |
| DLA_LG7_007090 | LG7 |  |  |  |  |
| DLA_LG7_007110 | LG7 |  |  |  |  |
| DLA_LG7_007120 | LG7 |  |  |  |  |
| DLA_LG7_007130 | LG7 |  |  |  |  |
| DLA_LG7_007140 | LG7 |  |  |  |  |
| DLA_LG7_007160 | LG7 |  |  |  |  |
| DLA_LG7_007170 | LG7 |  |  |  |  |
| DLA_LG7_007180 | LG7 |  |  |  |  |
| DLA_LG7_007190 | LG7 |  |  |  |  |
| DLA_LG7_007200 | LG7 |  |  |  |  |
| DLA_LG7_007210 | LG7 |  |  |  |  |
| DLA_LG7_007220 | LG7 |  |  |  |  |
| DLA_LG7_007240 | LG7 |  |  |  |  |
| DLA_LG7_007280 | LG7 |  |  |  |  |
| DLA_LG7_007290 | LG7 |  |  |  |  |
| DLA_LG7_007295 | LG7 |  |  |  |  |
| DLA_LG7_007300 | LG7 |  |  |  |  |
| DLA_LG7_007310 | LG7 |  |  |  |  |
| DLA_LG7_007320 | LG7 |  |  |  |  |
| DLA_LG7_007330 | LG7 |  |  |  |  |
| DLA_LG7_007350 | LG7 |  |  |  |  |
| DLA_LG7_007360 | LG7 |  |  |  |  |
| DLA_LG7_007370 | LG7 |  |  |  |  |
| DLA_LG7_007400 | LG7 |  |  |  |  |
| DLA_LG7_007410 | LG7 |  |  |  |  |
| DLA_LG7_007420 | LG7 |  |  |  |  |
| DLA_LG7_007430 | LG7 |  |  |  |  |
| DLA_LG7_007440 | LG7 |  |  |  |  |
| DLA_LG7_007450 | LG7 |  |  |  |  |
| DLA_LG7_007455 | LG7 |  |  |  |  |
| DLA_LG7_007460 | LG7 |  |  |  |  |
| DLA_LG7_007470 | LG7 |  |  |  |  |
| DLA_LG7_007480 | LG7 |  |  |  |  |
| DLA_LG7_007500 | LG7 |  |  |  |  |
| DLA_LG7_007510 | LG7 |  |  |  |  |
| DLA_LG7_007520 | LG7 |  |  |  |  |
| DLA_LG7_007530 | LG7 |  |  |  |  |
| DLA_LG7_007540 | LG7 |  |  |  |  |
| DLA_LG7_007550 | LG7 |  |  |  |  |
| DLA_LG7_007555 | LG7 |  |  |  |  |
| DLA_LG7_007570 | LG7 |  |  |  |  |
| DLA_LG7_007580 | LG7 |  |  |  |  |
| DLA_LG7_007590 | LG7 |  |  |  |  |
| DLA_LG7_007600 | LG7 |  |  |  |  |
| DLA_LG7_007610 | LG7 |  |  |  |  |
| DLA_LG7_007620 | LG7 |  |  |  |  |
| DLA_LG7_007640 | LG7 |  |  |  |  |
| DLA_LG7_007650 | LG7 |  |  |  |  |
| DLA_LG7_007680 | LG7 |  |  |  |  |
| DLA_LG7_007700 | LG7 |  |  |  |  |
| DLA_LG7_007710 | LG7 |  |  |  |  |
| DLA_LG7_007730 | LG7 |  |  |  |  |
| DLA_LG7_007735 | LG7 |  |  |  |  |
| DLA_LG7_007740 | LG7 |  |  |  |  |
| DLA_LG7_007750 | LG7 |  |  |  |  |
| DLA_LG7_007760 | LG7 |  |  |  |  |
| DLA_LG7_007770 | LG7 |  |  |  |  |
| DLA_LG7_007790 | LG7 |  |  |  |  |
| DLA_LG7_007800 | LG7 |  |  |  |  |
| DLA_LG7_007820 | LG7 |  |  |  |  |
| DLA_LG7_007830 | LG7 |  |  |  |  |
| DLA_LG7_007840 | LG7 |  |  |  |  |
| DLA_LG7_007850 | LG7 |  |  |  |  |
| DLA_LG7_007860 | LG7 |  |  |  |  |
| DLA_LG7_007880 | LG7 |  |  |  |  |
| DLA_LG7_007890 | LG7 |  |  |  |  |
| DLA_LG7_007900 | LG7 |  |  |  |  |
| DLA_LG7_007910 | LG7 |  |  |  |  |
| DLA_LG7_007915 | LG7 |  |  |  |  |
| DLA_LG7_007920 | LG7 |  |  |  |  |
| DLA_LG7_007950 | LG7 |  |  |  |  |
| DLA_LG7_007960 | LG7 |  |  |  |  |
| DLA_LG7_007970 | LG7 |  |  |  |  |
| DLA_LG7_007980 | LG7 |  |  |  |  |
| DLA_LG7_008000 | LG7 |  |  |  |  |
| DLA_LG7_008010 | LG7 |  |  |  |  |
| DLA_LG7_008020 | LG7 |  |  |  |  |
| DLA_LG7_008040 | LG7 |  |  |  |  |
| DLA_LG7_008050 | LG7 |  |  |  |  |
| DLA_LG7_008060 | LG7 |  |  |  |  |
| DLA_LG7_008070 | LG7 |  |  |  |  |
| DLA_LG7_008080 | LG7 |  |  |  |  |
| DLA_LG7_008090 | LG7 |  |  |  |  |
| DLA_LG7_008100 | LG7 |  |  |  |  |
| DLA_LG7_008120 | LG7 |  |  |  |  |
| DLA_LG7_008140 | LG7 |  |  |  |  |
| DLA_LG7_008150 | LG7 |  |  |  |  |
| DLA_LG7_008160 | LG7 |  |  |  |  |
| DLA_LG7_008180 | LG7 |  |  |  |  |
| DLA_LG7_008190 | LG7 |  |  |  |  |
| DLA_LG7_008200 | LG7 |  |  |  |  |
| DLA_LG7_008210 | LG7 |  |  |  |  |
| DLA_LG7_008220 | LG7 |  |  |  |  |
| DLA_LG7_008230 | LG7 |  |  |  |  |
| DLA_LG7_008240 | LG7 |  |  |  |  |
| DLA_LG7_008255 | LG7 |  |  |  |  |
| DLA_LG7_008260 | LG7 |  |  |  |  |
| DLA_LG7_008270 | LG7 |  |  |  |  |
| DLA_LG7_008280 | LG7 |  |  |  |  |
| DLA_LG7_008290 | LG7 |  |  |  |  |
| DLA_LG7_008305 | LG7 |  |  |  |  |
| DLA_LG7_008320 | LG7 |  |  |  |  |
| DLA_LG7_008330 | LG7 |  |  |  |  |
| DLA_LG7_008340 | LG7 |  |  |  |  |
| DLA_LG7_008350 | LG7 |  |  |  |  |
| DLA_LG7_008360 | LG7 |  |  |  |  |
| DLA_LG7_008370 | LG7 |  |  |  |  |
| DLA_LG7_008390 | LG7 |  |  |  |  |
| DLA_LG7_008400 | LG7 |  |  |  |  |
| DLA_LG7_008405 | LG7 |  |  |  |  |
| DLA_LG7_008410 | LG7 |  |  |  |  |
| DLA_LG7_008420 | LG7 |  |  |  |  |
| DLA_LG7_008430 | LG7 |  |  |  |  |
| DLA_LG7_008440 | LG7 |  |  |  |  |
| DLA_LG7_008450 | LG7 |  |  |  |  |
| DLA_LG7_008460 | LG7 |  |  |  |  |
| DLA_LG7_008470 | LG7 |  |  |  |  |
| DLA_LG7_008490 | LG7 |  |  |  |  |
| DLA_LG7_008500 | LG7 |  |  |  |  |
| DLA_LG7_008510 | LG7 |  |  |  |  |
| DLA_LG7_008520 | LG7 |  |  |  |  |
| DLA_LG7_008530 | LG7 |  |  |  |  |
| DLA_LG7_008540 | LG7 |  |  |  |  |
| DLA_LG7_008550 | LG7 |  |  |  |  |
| DLA_LG7_008560 | LG7 |  |  |  |  |
| DLA_LG7_008570 | LG7 |  |  |  |  |
| DLA_LG7_008580 | LG7 |  |  |  |  |
| DLA_LG7_008590 | LG7 |  |  |  |  |
| DLA_LG7_008600 | LG7 |  |  |  |  |
| DLA_LG7_008605 | LG7 |  |  |  |  |
| DLA_LG7_008610 | LG7 |  |  |  |  |
| DLA_LG7_008615 | LG7 |  |  |  |  |
| DLA_LG7_008620 | LG7 |  |  |  |  |
| DLA_LG7_008630 | LG7 |  |  |  |  |
| DLA_LG7_008640 | LG7 |  |  |  |  |
| DLA_LG7_008650 | LG7 |  |  |  |  |
| DLA_LG7_008660 | LG7 |  |  |  |  |
| DLA_LG7_008680 | LG7 |  |  |  |  |
| DLA_LG7_008700 | LG7 |  |  |  |  |
| DLA_LG7_008710 | LG7 |  |  |  |  |
| DLA_LG7_008720 | LG7 |  |  |  |  |
| DLA_LG7_008730 | LG7 |  |  |  |  |
| DLA_LG7_008740 | LG7 |  |  |  |  |
| DLA_LG7_008760 | LG7 |  |  |  |  |
| DLA_LG7_008770 | LG7 |  |  |  |  |
| DLA_LG7_008790 | LG7 |  |  |  |  |
| DLA_LG7_008800 | LG7 |  |  |  |  |
| DLA_LG7_008810 | LG7 |  |  |  |  |
| DLA_LG7_008820 | LG7 |  |  |  |  |
| DLA_LG7_008830 | LG7 |  |  |  |  |
| DLA_LG7_008840 | LG7 |  |  |  |  |
| DLA_LG7_008850 | LG7 |  |  |  |  |
| DLA_LG7_008860 | LG7 |  |  |  |  |
| DLA_LG7_008870 | LG7 |  |  |  |  |
| DLA_LG7_008880 | LG7 |  |  |  |  |
| DLA_LG7_008890 | LG7 |  |  |  |  |
| DLA_LG7_008900 | LG7 |  |  |  |  |
| DLA_LG7_008910 | LG7 |  |  |  |  |
| DLA_LG7_008920 | LG7 |  |  |  |  |
| DLA_LG7_008930 | LG7 |  |  |  |  |
| DLA_LG7_008940 | LG7 |  |  |  |  |
| DLA_LG7_008960 | LG7 |  |  |  |  |
| DLA_LG7_008980 | LG7 |  |  |  |  |
| DLA_LG7_008990 | LG7 |  |  |  |  |
| DLA_LG7_009000 | LG7 |  |  |  |  |
| DLA_LG7_009010 | LG7 |  |  |  |  |
| DLA_LG7_009015 | LG7 |  |  |  |  |
| DLA_LG7_009020 | LG7 |  |  |  |  |
| DLA_LG7_009030 | LG7 |  |  |  |  |
| DLA_LG7_009045 | LG7 |  |  |  |  |
| DLA_LG7_009050 | LG7 |  |  |  |  |
| DLA_LG7_009060 | LG7 |  |  |  |  |
| DLA_LG7_009070 | LG7 |  |  |  |  |
| DLA_LG7_009080 | LG7 |  |  |  |  |
| DLA_LG7_009090 | LG7 |  |  |  |  |
| DLA_LG7_009120 | LG7 |  |  |  |  |
| DLA_LG7_009130 | LG7 |  |  |  |  |
| DLA_LG7_009135 | LG7 |  |  |  |  |
| DLA_LG7_009135_2 | LG7 |  |  |  |  |
| DLA_LG7_009140 | LG7 |  |  |  |  |
| DLA_LG7_009170 | LG7 |  |  |  |  |
| DLA_LG7_009190 | LG7 |  |  |  |  |
| DLA_LG7_009200 | LG7 |  |  |  |  |
| DLA_LG7_009220 | LG7 |  |  |  |  |
| DLA_LG7_009240 | LG7 |  |  |  |  |
| DLA_LG7_009250 | LG7 |  |  |  |  |
| DLA_LG7_009255 | LG7 |  |  |  |  |
| DLA_LG7_009260 | LG7 |  |  |  |  |
| DLA_LG7_009270 | LG7 |  |  |  |  |
| DLA_LG7_009275 | LG7 |  |  |  |  |
| DLA_LG7_009280 | LG7 |  |  |  |  |
| DLA_LG7_009290 | LG7 |  |  |  |  |
| DLA_LG7_009295 | LG7 |  |  |  |  |
| DLA_LG7_009300 | LG7 |  |  |  |  |
| DLA_LG7_009320 | LG7 |  |  |  |  |
| DLA_LG7_009330 | LG7 |  |  |  |  |
| DLA_LG7_009340 | LG7 |  |  |  |  |
| DLA_LG7_009345 | LG7 |  |  |  |  |
| DLA_LG7_009350 | LG7 |  |  |  |  |
| DLA_LG7_009360 | LG7 |  |  |  |  |
| DLA_LG7_009380 | LG7 |  |  |  |  |
| DLA_LG7_009390 | LG7 |  |  |  |  |
| DLA_LG7_009400 | LG7 |  |  |  |  |
| DLA_LG7_009410 | LG7 |  |  |  |  |
| DLA_LG7_009420 | LG7 |  |  |  |  |
| DLA_LG7_009425 | LG7 |  |  |  |  |
| DLA_LG7_009425_2 | LG7 |  |  |  |  |
| DLA_LG7_009425_3 | LG7 |  |  |  |  |
| DLA_LG7_009430 | LG7 |  |  |  |  |
| DLA_LG7_009450 | LG7 |  |  |  |  |
| DLA_LG7_009460 | LG7 |  |  |  |  |
| DLA_LG7_009470 | LG7 |  |  |  |  |
| DLA_LG7_009480 | LG7 |  |  |  |  |
| DLA_LG7_009490 | LG7 |  |  |  |  |
| DLA_LG7_009500 | LG7 |  |  |  |  |
| DLA_LG7_009510 | LG7 |  |  |  |  |
| DLA_LG7_009520 | LG7 |  |  |  |  |
| DLA_LG7_009530 | LG7 |  |  |  |  |
| DLA_LG7_009550 | LG7 |  |  |  |  |
| DLA_LG7_009570 | LG7 |  |  |  |  |
| DLA_LG7_009580 | LG7 |  |  |  |  |
| DLA_LG7_009590 | LG7 |  |  |  |  |
| DLA_LG7_009610 | LG7 |  |  |  |  |
| DLA_LG7_009625 | LG7 |  |  |  |  |
| DLA_LG7_009625_2 | LG7 |  |  |  |  |
| DLA_LG8_003910 | LG8 |  |  |  |  |
| DLA_LG8_001130 | LG8 |  |  |  |  |
| DLA_LG8_002010 | LG8 |  |  |  |  |
| DLA_LG8_004330 | LG8 |  |  |  |  |
| DLA_LG8_008500 | LG8 |  |  |  |  |
| DLA_LG8_008730 | LG8 |  |  |  |  |
| DLA_LG8_000080 | LG8 |  |  |  |  |
| DLA_LG8_000160 | LG8 |  |  |  |  |
| DLA_LG8_000300 | LG8 |  |  |  |  |
| DLA_LG8_000460 | LG8 |  |  |  |  |
| DLA_LG8_000510 | LG8 |  |  |  |  |
| DLA_LG8_000660 | LG8 |  |  |  |  |
| DLA_LG8_000780 | LG8 |  |  |  |  |
| DLA_LG8_000890 | LG8 |  |  |  |  |
| DLA_LG8_001110 | LG8 |  |  |  |  |
| DLA_LG8_001140 | LG8 |  |  |  |  |
| DLA_LG8_001220 | LG8 |  |  |  |  |
| DLA_LG8_001280 | LG8 |  |  |  |  |
| DLA_LG8_001435 | LG8 |  |  |  |  |
| DLA_LG8_001780 | LG8 |  |  |  |  |
| DLA_LG8_001910 | LG8 |  |  |  |  |
| DLA_LG8_002330 | LG8 |  |  |  |  |
| DLA_LG8_002670 | LG8 |  |  |  |  |
| DLA_LG8_002720 | LG8 |  |  |  |  |
| DLA_LG8_003220 | LG8 |  |  |  |  |
| DLA_LG8_003300 | LG8 |  |  |  |  |
| DLA_LG8_003450 | LG8 |  |  |  |  |
| DLA_LG8_003580 | LG8 |  |  |  |  |
| DLA_LG8_003710 | LG8 |  |  |  |  |
| DLA_LG8_003820 | LG8 |  |  |  |  |
| DLA_LG8_004020 | LG8 |  |  |  |  |
| DLA_LG8_004120 | LG8 |  |  |  |  |
| DLA_LG8_004460 | LG8 |  |  |  |  |
| DLA_LG8_004750 | LG8 |  |  |  |  |
| DLA_LG8_004870 | LG8 |  |  |  |  |
| DLA_LG8_005060 | LG8 |  |  |  |  |
| DLA_LG8_005200 | LG8 |  |  |  |  |
| DLA_LG8_005250 | LG8 |  |  |  |  |
| DLA_LG8_005680 | LG8 |  |  |  |  |
| DLA_LG8_005810 | LG8 |  |  |  |  |
| DLA_LG8_006140 | LG8 |  |  |  |  |
| DLA_LG8_006410 | LG8 |  |  |  |  |
| DLA_LG8_006640 | LG8 |  |  |  |  |
| DLA_LG8_006670 | LG8 |  |  |  |  |
| DLA_LG8_006910 | LG8 |  |  |  |  |
| DLA_LG8_007000 | LG8 |  |  |  |  |
| DLA_LG8_007025 | LG8 |  |  |  |  |
| DLA_LG8_007250 | LG8 |  |  |  |  |
| DLA_LG8_007490 | LG8 |  |  |  |  |
| DLA_LG8_007550 | LG8 |  |  |  |  |
| DLA_LG8_007660 | LG8 |  |  |  |  |
| DLA_LG8_007720 | LG8 |  |  |  |  |
| DLA_LG8_007870 | LG8 |  |  |  |  |
| DLA_LG8_007880 | LG8 |  |  |  |  |
| DLA_LG8_008110 | LG8 |  |  |  |  |
| DLA_LG8_008310 | LG8 |  |  |  |  |
| DLA_LG8_008370 | LG8 |  |  |  |  |
| DLA_LG8_008580 | LG8 |  |  |  |  |
| DLA_LG8_008610 | LG8 |  |  |  |  |
| DLA_LG8_008670 | LG8 |  |  |  |  |
| DLA_LG8_008760 | LG8 |  |  |  |  |
| DLA_LG8_008830 | LG8 |  |  |  |  |
| DLA_LG8_009000 | LG8 |  |  |  |  |
| DLA_LG8_009610 | LG8 |  |  |  |  |
| DLA_LG8_000010 | LG8 |  |  |  |  |
| DLA_LG8_000020 | LG8 |  |  |  |  |
| DLA_LG8_000030 | LG8 |  |  |  |  |
| DLA_LG8_000040 | LG8 |  |  |  |  |
| DLA_LG8_000050 | LG8 |  |  |  |  |
| DLA_LG8_000070 | LG8 |  |  |  |  |
| DLA_LG8_000090 | LG8 |  |  |  |  |
| DLA_LG8_000100 | LG8 |  |  |  |  |
| DLA_LG8_000110 | LG8 |  |  |  |  |
| DLA_LG8_000130 | LG8 |  |  |  |  |
| DLA_LG8_000150 | LG8 |  |  |  |  |
| DLA_LG8_000155 | LG8 |  |  |  |  |
| DLA_LG8_000170 | LG8 |  |  |  |  |
| DLA_LG8_000190 | LG8 |  |  |  |  |
| DLA_LG8_000200 | LG8 |  |  |  |  |
| DLA_LG8_000210 | LG8 |  |  |  |  |
| DLA_LG8_000220 | LG8 |  |  |  |  |
| DLA_LG8_000225 | LG8 |  |  |  |  |
| DLA_LG8_000230 | LG8 |  |  |  |  |
| DLA_LG8_000240 | LG8 |  |  |  |  |
| DLA_LG8_000250 | LG8 |  |  |  |  |
| DLA_LG8_000260 | LG8 |  |  |  |  |
| DLA_LG8_000270 | LG8 |  |  |  |  |
| DLA_LG8_000280 | LG8 |  |  |  |  |
| DLA_LG8_000290 | LG8 |  |  |  |  |
| DLA_LG8_000310 | LG8 |  |  |  |  |
| DLA_LG8_000315 | LG8 |  |  |  |  |
| DLA_LG8_000320 | LG8 |  |  |  |  |
| DLA_LG8_000330 | LG8 |  |  |  |  |
| DLA_LG8_000340 | LG8 |  |  |  |  |
| DLA_LG8_000360 | LG8 |  |  |  |  |
| DLA_LG8_000370 | LG8 |  |  |  |  |
| DLA_LG8_000380 | LG8 |  |  |  |  |
| DLA_LG8_000390 | LG8 |  |  |  |  |
| DLA_LG8_000400 | LG8 |  |  |  |  |
| DLA_LG8_000410 | LG8 |  |  |  |  |
| DLA_LG8_000420 | LG8 |  |  |  |  |
| DLA_LG8_000430 | LG8 |  |  |  |  |
| DLA_LG8_000440 | LG8 |  |  |  |  |
| DLA_LG8_000450 | LG8 |  |  |  |  |
| DLA_LG8_000470 | LG8 |  |  |  |  |
| DLA_LG8_000480 | LG8 |  |  |  |  |
| DLA_LG8_000490 | LG8 |  |  |  |  |
| DLA_LG8_000520 | LG8 |  |  |  |  |
| DLA_LG8_000530 | LG8 |  |  |  |  |
| DLA_LG8_000540 | LG8 |  |  |  |  |
| DLA_LG8_000550 | LG8 |  |  |  |  |
| DLA_LG8_000560 | LG8 |  |  |  |  |
| DLA_LG8_000570 | LG8 |  |  |  |  |
| DLA_LG8_000580 | LG8 |  |  |  |  |
| DLA_LG8_000590 | LG8 |  |  |  |  |
| DLA_LG8_000600 | LG8 |  |  |  |  |
| DLA_LG8_000610 | LG8 |  |  |  |  |
| DLA_LG8_000620 | LG8 |  |  |  |  |
| DLA_LG8_000630 | LG8 |  |  |  |  |
| DLA_LG8_000640 | LG8 |  |  |  |  |
| DLA_LG8_000650 | LG8 |  |  |  |  |
| DLA_LG8_000670 | LG8 |  |  |  |  |
| DLA_LG8_000680 | LG8 |  |  |  |  |
| DLA_LG8_000690 | LG8 |  |  |  |  |
| DLA_LG8_000700 | LG8 |  |  |  |  |
| DLA_LG8_000710 | LG8 |  |  |  |  |
| DLA_LG8_000730 | LG8 |  |  |  |  |
| DLA_LG8_000740 | LG8 |  |  |  |  |
| DLA_LG8_000750 | LG8 |  |  |  |  |
| DLA_LG8_000760 | LG8 |  |  |  |  |
| DLA_LG8_000770 | LG8 |  |  |  |  |
| DLA_LG8_000790 | LG8 |  |  |  |  |
| DLA_LG8_000800 | LG8 |  |  |  |  |
| DLA_LG8_000810 | LG8 |  |  |  |  |
| DLA_LG8_000820 | LG8 |  |  |  |  |
| DLA_LG8_000830 | LG8 |  |  |  |  |
| DLA_LG8_000840 | LG8 |  |  |  |  |
| DLA_LG8_000850 | LG8 |  |  |  |  |
| DLA_LG8_000860 | LG8 |  |  |  |  |
| DLA_LG8_000870 | LG8 |  |  |  |  |
| DLA_LG8_000880 | LG8 |  |  |  |  |
| DLA_LG8_000900 | LG8 |  |  |  |  |
| DLA_LG8_000910 | LG8 |  |  |  |  |
| DLA_LG8_000920 | LG8 |  |  |  |  |
| DLA_LG8_000930 | LG8 |  |  |  |  |
| DLA_LG8_000940 | LG8 |  |  |  |  |
| DLA_LG8_000950 | LG8 |  |  |  |  |
| DLA_LG8_000960 | LG8 |  |  |  |  |
| DLA_LG8_000970 | LG8 |  |  |  |  |
| DLA_LG8_000980 | LG8 |  |  |  |  |
| DLA_LG8_000990 | LG8 |  |  |  |  |
| DLA_LG8_001000 | LG8 |  |  |  |  |
| DLA_LG8_001010 | LG8 |  |  |  |  |
| DLA_LG8_001020 | LG8 |  |  |  |  |
| DLA_LG8_001030 | LG8 |  |  |  |  |
| DLA_LG8_001050 | LG8 |  |  |  |  |
| DLA_LG8_001060 | LG8 |  |  |  |  |
| DLA_LG8_001070 | LG8 |  |  |  |  |
| DLA_LG8_001080 | LG8 |  |  |  |  |
| DLA_LG8_001090 | LG8 |  |  |  |  |
| DLA_LG8_001100 | LG8 |  |  |  |  |
| DLA_LG8_001120 | LG8 |  |  |  |  |
| DLA_LG8_001145 | LG8 |  |  |  |  |
| DLA_LG8_001150 | LG8 |  |  |  |  |
| DLA_LG8_001160 | LG8 |  |  |  |  |
| DLA_LG8_001170 | LG8 |  |  |  |  |
| DLA_LG8_001180 | LG8 |  |  |  |  |
| DLA_LG8_001190 | LG8 |  |  |  |  |
| DLA_LG8_001210 | LG8 |  |  |  |  |
| DLA_LG8_001230 | LG8 |  |  |  |  |
| DLA_LG8_001240 | LG8 |  |  |  |  |
| DLA_LG8_001250 | LG8 |  |  |  |  |
| DLA_LG8_001260 | LG8 |  |  |  |  |
| DLA_LG8_001270 | LG8 |  |  |  |  |
| DLA_LG8_001290 | LG8 |  |  |  |  |
| DLA_LG8_001300 | LG8 |  |  |  |  |
| DLA_LG8_001310 | LG8 |  |  |  |  |
| DLA_LG8_001320 | LG8 |  |  |  |  |
| DLA_LG8_001330 | LG8 |  |  |  |  |
| DLA_LG8_001340 | LG8 |  |  |  |  |
| DLA_LG8_001350 | LG8 |  |  |  |  |
| DLA_LG8_001360 | LG8 |  |  |  |  |
| DLA_LG8_001370 | LG8 |  |  |  |  |
| DLA_LG8_001380 | LG8 |  |  |  |  |
| DLA_LG8_001390 | LG8 |  |  |  |  |
| DLA_LG8_001395 | LG8 |  |  |  |  |
| DLA_LG8_001400 | LG8 |  |  |  |  |
| DLA_LG8_001410 | LG8 |  |  |  |  |
| DLA_LG8_001420 | LG8 |  |  |  |  |
| DLA_LG8_001430 | LG8 |  |  |  |  |
| DLA_LG8_001440 | LG8 |  |  |  |  |
| DLA_LG8_001450 | LG8 |  |  |  |  |
| DLA_LG8_001460 | LG8 |  |  |  |  |
| DLA_LG8_001470 | LG8 |  |  |  |  |
| DLA_LG8_001480 | LG8 |  |  |  |  |
| DLA_LG8_001490 | LG8 |  |  |  |  |
| DLA_LG8_001500 | LG8 |  |  |  |  |
| DLA_LG8_001510 | LG8 |  |  |  |  |
| DLA_LG8_001520 | LG8 |  |  |  |  |
| DLA_LG8_001530 | LG8 |  |  |  |  |
| DLA_LG8_001540 | LG8 |  |  |  |  |
| DLA_LG8_001545 | LG8 |  |  |  |  |
| DLA_LG8_001550 | LG8 |  |  |  |  |
| DLA_LG8_001560 | LG8 |  |  |  |  |
| DLA_LG8_001570 | LG8 |  |  |  |  |
| DLA_LG8_001590 | LG8 |  |  |  |  |
| DLA_LG8_001600 | LG8 |  |  |  |  |
| DLA_LG8_001610 | LG8 |  |  |  |  |
| DLA_LG8_001620 | LG8 |  |  |  |  |
| DLA_LG8_001630 | LG8 |  |  |  |  |
| DLA_LG8_001640 | LG8 |  |  |  |  |
| DLA_LG8_001650 | LG8 |  |  |  |  |
| DLA_LG8_001660 | LG8 |  |  |  |  |
| DLA_LG8_001670 | LG8 |  |  |  |  |
| DLA_LG8_001680 | LG8 |  |  |  |  |
| DLA_LG8_001690 | LG8 |  |  |  |  |
| DLA_LG8_001700 | LG8 |  |  |  |  |
| DLA_LG8_001710 | LG8 |  |  |  |  |
| DLA_LG8_001720 | LG8 |  |  |  |  |
| DLA_LG8_001730 | LG8 |  |  |  |  |
| DLA_LG8_001740 | LG8 |  |  |  |  |
| DLA_LG8_001750 | LG8 |  |  |  |  |
| DLA_LG8_001760 | LG8 |  |  |  |  |
| DLA_LG8_001770 | LG8 |  |  |  |  |
| DLA_LG8_001790 | LG8 |  |  |  |  |
| DLA_LG8_001800 | LG8 |  |  |  |  |
| DLA_LG8_001810 | LG8 |  |  |  |  |
| DLA_LG8_001820 | LG8 |  |  |  |  |
| DLA_LG8_001830 | LG8 |  |  |  |  |
| DLA_LG8_001840 | LG8 |  |  |  |  |
| DLA_LG8_001860 | LG8 |  |  |  |  |
| DLA_LG8_001870 | LG8 |  |  |  |  |
| DLA_LG8_001880 | LG8 |  |  |  |  |
| DLA_LG8_001890 | LG8 |  |  |  |  |
| DLA_LG8_001900 | LG8 |  |  |  |  |
| DLA_LG8_001920 | LG8 |  |  |  |  |
| DLA_LG8_001930 | LG8 |  |  |  |  |
| DLA_LG8_001950 | LG8 |  |  |  |  |
| DLA_LG8_001960 | LG8 |  |  |  |  |
| DLA_LG8_001970 | LG8 |  |  |  |  |
| DLA_LG8_001980 | LG8 |  |  |  |  |
| DLA_LG8_001990 | LG8 |  |  |  |  |
| DLA_LG8_002000 | LG8 |  |  |  |  |
| DLA_LG8_002020 | LG8 |  |  |  |  |
| DLA_LG8_002030 | LG8 |  |  |  |  |
| DLA_LG8_002040 | LG8 |  |  |  |  |
| DLA_LG8_002045 | LG8 |  |  |  |  |
| DLA_LG8_002050 | LG8 |  |  |  |  |
| DLA_LG8_002060 | LG8 |  |  |  |  |
| DLA_LG8_002070 | LG8 |  |  |  |  |
| DLA_LG8_002080 | LG8 |  |  |  |  |
| DLA_LG8_002090 | LG8 |  |  |  |  |
| DLA_LG8_002100 | LG8 |  |  |  |  |
| DLA_LG8_002110 | LG8 |  |  |  |  |
| DLA_LG8_002120 | LG8 |  |  |  |  |
| DLA_LG8_002150 | LG8 |  |  |  |  |
| DLA_LG8_002160 | LG8 |  |  |  |  |
| DLA_LG8_002170 | LG8 |  |  |  |  |
| DLA_LG8_002180 | LG8 |  |  |  |  |
| DLA_LG8_002190 | LG8 |  |  |  |  |
| DLA_LG8_002200 | LG8 |  |  |  |  |
| DLA_LG8_002210 | LG8 |  |  |  |  |
| DLA_LG8_002220 | LG8 |  |  |  |  |
| DLA_LG8_002230 | LG8 |  |  |  |  |
| DLA_LG8_002240 | LG8 |  |  |  |  |
| DLA_LG8_002250 | LG8 |  |  |  |  |
| DLA_LG8_002260 | LG8 |  |  |  |  |
| DLA_LG8_002270 | LG8 |  |  |  |  |
| DLA_LG8_002280 | LG8 |  |  |  |  |
| DLA_LG8_002285 | LG8 |  |  |  |  |
| DLA_LG8_002290 | LG8 |  |  |  |  |
| DLA_LG8_002300 | LG8 |  |  |  |  |
| DLA_LG8_002310 | LG8 |  |  |  |  |
| DLA_LG8_002320 | LG8 |  |  |  |  |
| DLA_LG8_002340 | LG8 |  |  |  |  |
| DLA_LG8_002350 | LG8 |  |  |  |  |
| DLA_LG8_002360 | LG8 |  |  |  |  |
| DLA_LG8_002370 | LG8 |  |  |  |  |
| DLA_LG8_002380 | LG8 |  |  |  |  |
| DLA_LG8_002400 | LG8 |  |  |  |  |
| DLA_LG8_002410 | LG8 |  |  |  |  |
| DLA_LG8_002420 | LG8 |  |  |  |  |
| DLA_LG8_002430 | LG8 |  |  |  |  |
| DLA_LG8_002440 | LG8 |  |  |  |  |
| DLA_LG8_002450 | LG8 |  |  |  |  |
| DLA_LG8_002460 | LG8 |  |  |  |  |
| DLA_LG8_002485 | LG8 |  |  |  |  |
| DLA_LG8_002490 | LG8 |  |  |  |  |
| DLA_LG8_002500 | LG8 |  |  |  |  |
| DLA_LG8_002510 | LG8 |  |  |  |  |
| DLA_LG8_002520 | LG8 |  |  |  |  |
| DLA_LG8_002530 | LG8 |  |  |  |  |
| DLA_LG8_002540 | LG8 |  |  |  |  |
| DLA_LG8_002550 | LG8 |  |  |  |  |
| DLA_LG8_002570 | LG8 |  |  |  |  |
| DLA_LG8_002585 | LG8 |  |  |  |  |
| DLA_LG8_002590 | LG8 |  |  |  |  |
| DLA_LG8_002600 | LG8 |  |  |  |  |
| DLA_LG8_002620 | LG8 |  |  |  |  |
| DLA_LG8_002630 | LG8 |  |  |  |  |
| DLA_LG8_002640 | LG8 |  |  |  |  |
| DLA_LG8_002650 | LG8 |  |  |  |  |
| DLA_LG8_002680 | LG8 |  |  |  |  |
| DLA_LG8_002690 | LG8 |  |  |  |  |
| DLA_LG8_002700 | LG8 |  |  |  |  |
| DLA_LG8_002710 | LG8 |  |  |  |  |
| DLA_LG8_002750 | LG8 |  |  |  |  |
| DLA_LG8_002760 | LG8 |  |  |  |  |
| DLA_LG8_002770 | LG8 |  |  |  |  |
| DLA_LG8_002790 | LG8 |  |  |  |  |
| DLA_LG8_002795 | LG8 |  |  |  |  |
| DLA_LG8_002800 | LG8 |  |  |  |  |
| DLA_LG8_002810 | LG8 |  |  |  |  |
| DLA_LG8_002815 | LG8 |  |  |  |  |
| DLA_LG8_002815_2 | LG8 |  |  |  |  |
| DLA_LG8_002820 | LG8 |  |  |  |  |
| DLA_LG8_002840 | LG8 |  |  |  |  |
| DLA_LG8_002850 | LG8 |  |  |  |  |
| DLA_LG8_002860 | LG8 |  |  |  |  |
| DLA_LG8_002865 | LG8 |  |  |  |  |
| DLA_LG8_002870 | LG8 |  |  |  |  |
| DLA_LG8_002880 | LG8 |  |  |  |  |
| DLA_LG8_002890 | LG8 |  |  |  |  |
| DLA_LG8_002900 | LG8 |  |  |  |  |
| DLA_LG8_002910 | LG8 |  |  |  |  |
| DLA_LG8_002920 | LG8 |  |  |  |  |
| DLA_LG8_002930 | LG8 |  |  |  |  |
| DLA_LG8_002940 | LG8 |  |  |  |  |
| DLA_LG8_002950 | LG8 |  |  |  |  |
| DLA_LG8_002970 | LG8 |  |  |  |  |
| DLA_LG8_002980 | LG8 |  |  |  |  |
| DLA_LG8_002990 | LG8 |  |  |  |  |
| DLA_LG8_003000 | LG8 |  |  |  |  |
| DLA_LG8_003010 | LG8 |  |  |  |  |
| DLA_LG8_003020 | LG8 |  |  |  |  |
| DLA_LG8_003030 | LG8 |  |  |  |  |
| DLA_LG8_003040 | LG8 |  |  |  |  |
| DLA_LG8_003050 | LG8 |  |  |  |  |
| DLA_LG8_003060 | LG8 |  |  |  |  |
| DLA_LG8_003070 | LG8 |  |  |  |  |
| DLA_LG8_003080 | LG8 |  |  |  |  |
| DLA_LG8_003090 | LG8 |  |  |  |  |
| DLA_LG8_003100 | LG8 |  |  |  |  |
| DLA_LG8_003110 | LG8 |  |  |  |  |
| DLA_LG8_003120 | LG8 |  |  |  |  |
| DLA_LG8_003130 | LG8 |  |  |  |  |
| DLA_LG8_003140 | LG8 |  |  |  |  |
| DLA_LG8_003150 | LG8 |  |  |  |  |
| DLA_LG8_003160 | LG8 |  |  |  |  |
| DLA_LG8_003170 | LG8 |  |  |  |  |
| DLA_LG8_003180 | LG8 |  |  |  |  |
| DLA_LG8_003190 | LG8 |  |  |  |  |
| DLA_LG8_003200 | LG8 |  |  |  |  |
| DLA_LG8_003210 | LG8 |  |  |  |  |
| DLA_LG8_003230 | LG8 |  |  |  |  |
| DLA_LG8_003240 | LG8 |  |  |  |  |
| DLA_LG8_003250 | LG8 |  |  |  |  |
| DLA_LG8_003260 | LG8 |  |  |  |  |
| DLA_LG8_003270 | LG8 |  |  |  |  |
| DLA_LG8_003280 | LG8 |  |  |  |  |
| DLA_LG8_003290 | LG8 |  |  |  |  |
| DLA_LG8_003310 | LG8 |  |  |  |  |
| DLA_LG8_003320 | LG8 |  |  |  |  |
| DLA_LG8_003330 | LG8 |  |  |  |  |
| DLA_LG8_003340 | LG8 |  |  |  |  |
| DLA_LG8_003350 | LG8 |  |  |  |  |
| DLA_LG8_003360 | LG8 |  |  |  |  |
| DLA_LG8_003370 | LG8 |  |  |  |  |
| DLA_LG8_003380 | LG8 |  |  |  |  |
| DLA_LG8_003385 | LG8 |  |  |  |  |
| DLA_LG8_003390 | LG8 |  |  |  |  |
| DLA_LG8_003395 | LG8 |  |  |  |  |
| DLA_LG8_003400 | LG8 |  |  |  |  |
| DLA_LG8_003420 | LG8 |  |  |  |  |
| DLA_LG8_003430 | LG8 |  |  |  |  |
| DLA_LG8_003440 | LG8 |  |  |  |  |
| DLA_LG8_003460 | LG8 |  |  |  |  |
| DLA_LG8_003470 | LG8 |  |  |  |  |
| DLA_LG8_003480 | LG8 |  |  |  |  |
| DLA_LG8_003490 | LG8 |  |  |  |  |
| DLA_LG8_003500 | LG8 |  |  |  |  |
| DLA_LG8_003510 | LG8 |  |  |  |  |
| DLA_LG8_003520 | LG8 |  |  |  |  |
| DLA_LG8_003530 | LG8 |  |  |  |  |
| DLA_LG8_003540 | LG8 |  |  |  |  |
| DLA_LG8_003550 | LG8 |  |  |  |  |
| DLA_LG8_003560 | LG8 |  |  |  |  |
| DLA_LG8_003570 | LG8 |  |  |  |  |
| DLA_LG8_003590 | LG8 |  |  |  |  |
| DLA_LG8_003600 | LG8 |  |  |  |  |
| DLA_LG8_003605 | LG8 |  |  |  |  |
| DLA_LG8_003610 | LG8 |  |  |  |  |
| DLA_LG8_003620 | LG8 |  |  |  |  |
| DLA_LG8_003630 | LG8 |  |  |  |  |
| DLA_LG8_003640 | LG8 |  |  |  |  |
| DLA_LG8_003650 | LG8 |  |  |  |  |
| DLA_LG8_003660 | LG8 |  |  |  |  |
| DLA_LG8_003670 | LG8 |  |  |  |  |
| DLA_LG8_003680 | LG8 |  |  |  |  |
| DLA_LG8_003690 | LG8 |  |  |  |  |
| DLA_LG8_003700 | LG8 |  |  |  |  |
| DLA_LG8_003720 | LG8 |  |  |  |  |
| DLA_LG8_003730 | LG8 |  |  |  |  |
| DLA_LG8_003740 | LG8 |  |  |  |  |
| DLA_LG8_003750 | LG8 |  |  |  |  |
| DLA_LG8_003760 | LG8 |  |  |  |  |
| DLA_LG8_003770 | LG8 |  |  |  |  |
| DLA_LG8_003780 | LG8 |  |  |  |  |
| DLA_LG8_003785 | LG8 |  |  |  |  |
| DLA_LG8_003790 | LG8 |  |  |  |  |
| DLA_LG8_003800 | LG8 |  |  |  |  |
| DLA_LG8_003810 | LG8 |  |  |  |  |
| DLA_LG8_003830 | LG8 |  |  |  |  |
| DLA_LG8_003840 | LG8 |  |  |  |  |
| DLA_LG8_003850 | LG8 |  |  |  |  |
| DLA_LG8_003860 | LG8 |  |  |  |  |
| DLA_LG8_003870 | LG8 |  |  |  |  |
| DLA_LG8_003880 | LG8 |  |  |  |  |
| DLA_LG8_003890 | LG8 |  |  |  |  |
| DLA_LG8_003900 | LG8 |  |  |  |  |
| DLA_LG8_003930 | LG8 |  |  |  |  |
| DLA_LG8_003940 | LG8 |  |  |  |  |
| DLA_LG8_003945 | LG8 |  |  |  |  |
| DLA_LG8_003950 | LG8 |  |  |  |  |
| DLA_LG8_003960 | LG8 |  |  |  |  |
| DLA_LG8_003970 | LG8 |  |  |  |  |
| DLA_LG8_003980 | LG8 |  |  |  |  |
| DLA_LG8_003985 | LG8 |  |  |  |  |
| DLA_LG8_003990 | LG8 |  |  |  |  |
| DLA_LG8_004000 | LG8 |  |  |  |  |
| DLA_LG8_004010 | LG8 |  |  |  |  |
| DLA_LG8_004030 | LG8 |  |  |  |  |
| DLA_LG8_004040 | LG8 |  |  |  |  |
| DLA_LG8_004050 | LG8 |  |  |  |  |
| DLA_LG8_004060 | LG8 |  |  |  |  |
| DLA_LG8_004080 | LG8 |  |  |  |  |
| DLA_LG8_004085 | LG8 |  |  |  |  |
| DLA_LG8_004085_2 | LG8 |  |  |  |  |
| DLA_LG8_004090 | LG8 |  |  |  |  |
| DLA_LG8_004095 | LG8 |  |  |  |  |
| DLA_LG8_004100 | LG8 |  |  |  |  |
| DLA_LG8_004110 | LG8 |  |  |  |  |
| DLA_LG8_004130 | LG8 |  |  |  |  |
| DLA_LG8_004140 | LG8 |  |  |  |  |
| DLA_LG8_004160 | LG8 |  |  |  |  |
| DLA_LG8_004170 | LG8 |  |  |  |  |
| DLA_LG8_004175 | LG8 |  |  |  |  |
| DLA_LG8_004175_2 | LG8 |  |  |  |  |
| DLA_LG8_004180 | LG8 |  |  |  |  |
| DLA_LG8_004190 | LG8 |  |  |  |  |
| DLA_LG8_004200 | LG8 |  |  |  |  |
| DLA_LG8_004210 | LG8 |  |  |  |  |
| DLA_LG8_004220 | LG8 |  |  |  |  |
| DLA_LG8_004230 | LG8 |  |  |  |  |
| DLA_LG8_004240 | LG8 |  |  |  |  |
| DLA_LG8_004260 | LG8 |  |  |  |  |
| DLA_LG8_004270 | LG8 |  |  |  |  |
| DLA_LG8_004280 | LG8 |  |  |  |  |
| DLA_LG8_004285 | LG8 |  |  |  |  |
| DLA_LG8_004285_2 | LG8 |  |  |  |  |
| DLA_LG8_004290 | LG8 |  |  |  |  |
| DLA_LG8_004300 | LG8 |  |  |  |  |
| DLA_LG8_004340 | LG8 |  |  |  |  |
| DLA_LG8_004350 | LG8 |  |  |  |  |
| DLA_LG8_004360 | LG8 |  |  |  |  |
| DLA_LG8_004365 | LG8 |  |  |  |  |
| DLA_LG8_004390 | LG8 |  |  |  |  |
| DLA_LG8_004400 | LG8 |  |  |  |  |
| DLA_LG8_004410 | LG8 |  |  |  |  |
| DLA_LG8_004420 | LG8 |  |  |  |  |
| DLA_LG8_004430 | LG8 |  |  |  |  |
| DLA_LG8_004440 | LG8 |  |  |  |  |
| DLA_LG8_004445 | LG8 |  |  |  |  |
| DLA_LG8_004450 | LG8 |  |  |  |  |
| DLA_LG8_004455 | LG8 |  |  |  |  |
| DLA_LG8_004470 | LG8 |  |  |  |  |
| DLA_LG8_004480 | LG8 |  |  |  |  |
| DLA_LG8_004490 | LG8 |  |  |  |  |
| DLA_LG8_004500 | LG8 |  |  |  |  |
| DLA_LG8_004520 | LG8 |  |  |  |  |
| DLA_LG8_004530 | LG8 |  |  |  |  |
| DLA_LG8_004540 | LG8 |  |  |  |  |
| DLA_LG8_004560 | LG8 |  |  |  |  |
| DLA_LG8_004570 | LG8 |  |  |  |  |
| DLA_LG8_004580 | LG8 |  |  |  |  |
| DLA_LG8_004590 | LG8 |  |  |  |  |
| DLA_LG8_004600 | LG8 |  |  |  |  |
| DLA_LG8_004620 | LG8 |  |  |  |  |
| DLA_LG8_004630 | LG8 |  |  |  |  |
| DLA_LG8_004640 | LG8 |  |  |  |  |
| DLA_LG8_004650 | LG8 |  |  |  |  |
| DLA_LG8_004660 | LG8 |  |  |  |  |
| DLA_LG8_004670 | LG8 |  |  |  |  |
| DLA_LG8_004680 | LG8 |  |  |  |  |
| DLA_LG8_004700 | LG8 |  |  |  |  |
| DLA_LG8_004720 | LG8 |  |  |  |  |
| DLA_LG8_004730 | LG8 |  |  |  |  |
| DLA_LG8_004740 | LG8 |  |  |  |  |
| DLA_LG8_004745 | LG8 |  |  |  |  |
| DLA_LG8_004770 | LG8 |  |  |  |  |
| DLA_LG8_004780 | LG8 |  |  |  |  |
| DLA_LG8_004790 | LG8 |  |  |  |  |
| DLA_LG8_004800 | LG8 |  |  |  |  |
| DLA_LG8_004810 | LG8 |  |  |  |  |
| DLA_LG8_004820 | LG8 |  |  |  |  |
| DLA_LG8_004830 | LG8 |  |  |  |  |
| DLA_LG8_004840 | LG8 |  |  |  |  |
| DLA_LG8_004850 | LG8 |  |  |  |  |
| DLA_LG8_004860 | LG8 |  |  |  |  |
| DLA_LG8_004880 | LG8 |  |  |  |  |
| DLA_LG8_004890 | LG8 |  |  |  |  |
| DLA_LG8_004900 | LG8 |  |  |  |  |
| DLA_LG8_004910 | LG8 |  |  |  |  |
| DLA_LG8_004920 | LG8 |  |  |  |  |
| DLA_LG8_004930 | LG8 |  |  |  |  |
| DLA_LG8_004940 | LG8 |  |  |  |  |
| DLA_LG8_004950 | LG8 |  |  |  |  |
| DLA_LG8_004960 | LG8 |  |  |  |  |
| DLA_LG8_004980 | LG8 |  |  |  |  |
| DLA_LG8_004990 | LG8 |  |  |  |  |
| DLA_LG8_005000 | LG8 |  |  |  |  |
| DLA_LG8_005010 | LG8 |  |  |  |  |
| DLA_LG8_005020 | LG8 |  |  |  |  |
| DLA_LG8_005030 | LG8 |  |  |  |  |
| DLA_LG8_005040 | LG8 |  |  |  |  |
| DLA_LG8_005050 | LG8 |  |  |  |  |
| DLA_LG8_005070 | LG8 |  |  |  |  |
| DLA_LG8_005080 | LG8 |  |  |  |  |
| DLA_LG8_005090 | LG8 |  |  |  |  |
| DLA_LG8_005100 | LG8 |  |  |  |  |
| DLA_LG8_005110 | LG8 |  |  |  |  |
| DLA_LG8_005120 | LG8 |  |  |  |  |
| DLA_LG8_005130 | LG8 |  |  |  |  |
| DLA_LG8_005140 | LG8 |  |  |  |  |
| DLA_LG8_005150 | LG8 |  |  |  |  |
| DLA_LG8_005160 | LG8 |  |  |  |  |
| DLA_LG8_005170 | LG8 |  |  |  |  |
| DLA_LG8_005180 | LG8 |  |  |  |  |
| DLA_LG8_005190 | LG8 |  |  |  |  |
| DLA_LG8_005210 | LG8 |  |  |  |  |
| DLA_LG8_005220 | LG8 |  |  |  |  |
| DLA_LG8_005225 | LG8 |  |  |  |  |
| DLA_LG8_005240 | LG8 |  |  |  |  |
| DLA_LG8_005270 | LG8 |  |  |  |  |
| DLA_LG8_005280 | LG8 |  |  |  |  |
| DLA_LG8_005300 | LG8 |  |  |  |  |
| DLA_LG8_005310 | LG8 |  |  |  |  |
| DLA_LG8_005320 | LG8 |  |  |  |  |
| DLA_LG8_005330 | LG8 |  |  |  |  |
| DLA_LG8_005340 | LG8 |  |  |  |  |
| DLA_LG8_005350 | LG8 |  |  |  |  |
| DLA_LG8_005360 | LG8 |  |  |  |  |
| DLA_LG8_005370 | LG8 |  |  |  |  |
| DLA_LG8_005390 | LG8 |  |  |  |  |
| DLA_LG8_005400 | LG8 |  |  |  |  |
| DLA_LG8_005405 | LG8 |  |  |  |  |
| DLA_LG8_005410 | LG8 |  |  |  |  |
| DLA_LG8_005420 | LG8 |  |  |  |  |
| DLA_LG8_005430 | LG8 |  |  |  |  |
| DLA_LG8_005440 | LG8 |  |  |  |  |
| DLA_LG8_005445 | LG8 |  |  |  |  |
| DLA_LG8_005450 | LG8 |  |  |  |  |
| DLA_LG8_005460 | LG8 |  |  |  |  |
| DLA_LG8_005470 | LG8 |  |  |  |  |
| DLA_LG8_005490 | LG8 |  |  |  |  |
| DLA_LG8_005495 | LG8 |  |  |  |  |
| DLA_LG8_005500 | LG8 |  |  |  |  |
| DLA_LG8_005510 | LG8 |  |  |  |  |
| DLA_LG8_005520 | LG8 |  |  |  |  |
| DLA_LG8_005530 | LG8 |  |  |  |  |
| DLA_LG8_005540 | LG8 |  |  |  |  |
| DLA_LG8_005550 | LG8 |  |  |  |  |
| DLA_LG8_005555 | LG8 |  |  |  |  |
| DLA_LG8_005560 | LG8 |  |  |  |  |
| DLA_LG8_005570 | LG8 |  |  |  |  |
| DLA_LG8_005590 | LG8 |  |  |  |  |
| DLA_LG8_005620 | LG8 |  |  |  |  |
| DLA_LG8_005630 | LG8 |  |  |  |  |
| DLA_LG8_005640 | LG8 |  |  |  |  |
| DLA_LG8_005650 | LG8 |  |  |  |  |
| DLA_LG8_005660 | LG8 |  |  |  |  |
| DLA_LG8_005670 | LG8 |  |  |  |  |
| DLA_LG8_005690 | LG8 |  |  |  |  |
| DLA_LG8_005700 | LG8 |  |  |  |  |
| DLA_LG8_005720 | LG8 |  |  |  |  |
| DLA_LG8_005730 | LG8 |  |  |  |  |
| DLA_LG8_005740 | LG8 |  |  |  |  |
| DLA_LG8_005750 | LG8 |  |  |  |  |
| DLA_LG8_005770 | LG8 |  |  |  |  |
| DLA_LG8_005780 | LG8 |  |  |  |  |
| DLA_LG8_005790 | LG8 |  |  |  |  |
| DLA_LG8_005800 | LG8 |  |  |  |  |
| DLA_LG8_005820 | LG8 |  |  |  |  |
| DLA_LG8_005825 | LG8 |  |  |  |  |
| DLA_LG8_005840 | LG8 |  |  |  |  |
| DLA_LG8_005850 | LG8 |  |  |  |  |
| DLA_LG8_005860 | LG8 |  |  |  |  |
| DLA_LG8_005870 | LG8 |  |  |  |  |
| DLA_LG8_005880 | LG8 |  |  |  |  |
| DLA_LG8_005890 | LG8 |  |  |  |  |
| DLA_LG8_005900 | LG8 |  |  |  |  |
| DLA_LG8_005920 | LG8 |  |  |  |  |
| DLA_LG8_005930 | LG8 |  |  |  |  |
| DLA_LG8_005940 | LG8 |  |  |  |  |
| DLA_LG8_005950 | LG8 |  |  |  |  |
| DLA_LG8_005960 | LG8 |  |  |  |  |
| DLA_LG8_005970 | LG8 |  |  |  |  |
| DLA_LG8_005980 | LG8 |  |  |  |  |
| DLA_LG8_005990 | LG8 |  |  |  |  |
| DLA_LG8_006000 | LG8 |  |  |  |  |
| DLA_LG8_006010 | LG8 |  |  |  |  |
| DLA_LG8_006020 | LG8 |  |  |  |  |
| DLA_LG8_006030 | LG8 |  |  |  |  |
| DLA_LG8_006040 | LG8 |  |  |  |  |
| DLA_LG8_006050 | LG8 |  |  |  |  |
| DLA_LG8_006060 | LG8 |  |  |  |  |
| DLA_LG8_006080 | LG8 |  |  |  |  |
| DLA_LG8_006090 | LG8 |  |  |  |  |
| DLA_LG8_006095 | LG8 |  |  |  |  |
| DLA_LG8_006100 | LG8 |  |  |  |  |
| DLA_LG8_006110 | LG8 |  |  |  |  |
| DLA_LG8_006115 | LG8 |  |  |  |  |
| DLA_LG8_006150 | LG8 |  |  |  |  |
| DLA_LG8_006160 | LG8 |  |  |  |  |
| DLA_LG8_006170 | LG8 |  |  |  |  |
| DLA_LG8_006180 | LG8 |  |  |  |  |
| DLA_LG8_006190 | LG8 |  |  |  |  |
| DLA_LG8_006210 | LG8 |  |  |  |  |
| DLA_LG8_006220 | LG8 |  |  |  |  |
| DLA_LG8_006240 | LG8 |  |  |  |  |
| DLA_LG8_006250 | LG8 |  |  |  |  |
| DLA_LG8_006255 | LG8 |  |  |  |  |
| DLA_LG8_006260 | LG8 |  |  |  |  |
| DLA_LG8_006270 | LG8 |  |  |  |  |
| DLA_LG8_006280 | LG8 |  |  |  |  |
| DLA_LG8_006290 | LG8 |  |  |  |  |
| DLA_LG8_006310 | LG8 |  |  |  |  |
| DLA_LG8_006320 | LG8 |  |  |  |  |
| DLA_LG8_006330 | LG8 |  |  |  |  |
| DLA_LG8_006340 | LG8 |  |  |  |  |
| DLA_LG8_006350 | LG8 |  |  |  |  |
| DLA_LG8_006360 | LG8 |  |  |  |  |
| DLA_LG8_006370 | LG8 |  |  |  |  |
| DLA_LG8_006380 | LG8 |  |  |  |  |
| DLA_LG8_006390 | LG8 |  |  |  |  |
| DLA_LG8_006400 | LG8 |  |  |  |  |
| DLA_LG8_006420 | LG8 |  |  |  |  |
| DLA_LG8_006430 | LG8 |  |  |  |  |
| DLA_LG8_006440 | LG8 |  |  |  |  |
| DLA_LG8_006460 | LG8 |  |  |  |  |
| DLA_LG8_006480 | LG8 |  |  |  |  |
| DLA_LG8_006490 | LG8 |  |  |  |  |
| DLA_LG8_006500 | LG8 |  |  |  |  |
| DLA_LG8_006510 | LG8 |  |  |  |  |
| DLA_LG8_006520 | LG8 |  |  |  |  |
| DLA_LG8_006530 | LG8 |  |  |  |  |
| DLA_LG8_006550 | LG8 |  |  |  |  |
| DLA_LG8_006560 | LG8 |  |  |  |  |
| DLA_LG8_006570 | LG8 |  |  |  |  |
| DLA_LG8_006580 | LG8 |  |  |  |  |
| DLA_LG8_006590 | LG8 |  |  |  |  |
| DLA_LG8_006600 | LG8 |  |  |  |  |
| DLA_LG8_006610 | LG8 |  |  |  |  |
| DLA_LG8_006620 | LG8 |  |  |  |  |
| DLA_LG8_006630 | LG8 |  |  |  |  |
| DLA_LG8_006650 | LG8 |  |  |  |  |
| DLA_LG8_006660 | LG8 |  |  |  |  |
| DLA_LG8_006680 | LG8 |  |  |  |  |
| DLA_LG8_006690 | LG8 |  |  |  |  |
| DLA_LG8_006700 | LG8 |  |  |  |  |
| DLA_LG8_006710 | LG8 |  |  |  |  |
| DLA_LG8_006720 | LG8 |  |  |  |  |
| DLA_LG8_006730 | LG8 |  |  |  |  |
| DLA_LG8_006740 | LG8 |  |  |  |  |
| DLA_LG8_006750 | LG8 |  |  |  |  |
| DLA_LG8_006760 | LG8 |  |  |  |  |
| DLA_LG8_006770 | LG8 |  |  |  |  |
| DLA_LG8_006780 | LG8 |  |  |  |  |
| DLA_LG8_006790 | LG8 |  |  |  |  |
| DLA_LG8_006800 | LG8 |  |  |  |  |
| DLA_LG8_006810 | LG8 |  |  |  |  |
| DLA_LG8_006820 | LG8 |  |  |  |  |
| DLA_LG8_006830 | LG8 |  |  |  |  |
| DLA_LG8_006840 | LG8 |  |  |  |  |
| DLA_LG8_006850 | LG8 |  |  |  |  |
| DLA_LG8_006860 | LG8 |  |  |  |  |
| DLA_LG8_006870 | LG8 |  |  |  |  |
| DLA_LG8_006880 | LG8 |  |  |  |  |
| DLA_LG8_006890 | LG8 |  |  |  |  |
| DLA_LG8_006900 | LG8 |  |  |  |  |
| DLA_LG8_006920 | LG8 |  |  |  |  |
| DLA_LG8_006930 | LG8 |  |  |  |  |
| DLA_LG8_006940 | LG8 |  |  |  |  |
| DLA_LG8_006950 | LG8 |  |  |  |  |
| DLA_LG8_006960 | LG8 |  |  |  |  |
| DLA_LG8_006970 | LG8 |  |  |  |  |
| DLA_LG8_006980 | LG8 |  |  |  |  |
| DLA_LG8_006990 | LG8 |  |  |  |  |
| DLA_LG8_007010 | LG8 |  |  |  |  |
| DLA_LG8_007020 | LG8 |  |  |  |  |
| DLA_LG8_007030 | LG8 |  |  |  |  |
| DLA_LG8_007040 | LG8 |  |  |  |  |
| DLA_LG8_007050 | LG8 |  |  |  |  |
| DLA_LG8_007060 | LG8 |  |  |  |  |
| DLA_LG8_007070 | LG8 |  |  |  |  |
| DLA_LG8_007080 | LG8 |  |  |  |  |
| DLA_LG8_007090 | LG8 |  |  |  |  |
| DLA_LG8_007100 | LG8 |  |  |  |  |
| DLA_LG8_007110 | LG8 |  |  |  |  |
| DLA_LG8_007120 | LG8 |  |  |  |  |
| DLA_LG8_007130 | LG8 |  |  |  |  |
| DLA_LG8_007140 | LG8 |  |  |  |  |
| DLA_LG8_007150 | LG8 |  |  |  |  |
| DLA_LG8_007160 | LG8 |  |  |  |  |
| DLA_LG8_007165 | LG8 |  |  |  |  |
| DLA_LG8_007170 | LG8 |  |  |  |  |
| DLA_LG8_007180 | LG8 |  |  |  |  |
| DLA_LG8_007190 | LG8 |  |  |  |  |
| DLA_LG8_007200 | LG8 |  |  |  |  |
| DLA_LG8_007210 | LG8 |  |  |  |  |
| DLA_LG8_007220 | LG8 |  |  |  |  |
| DLA_LG8_007230 | LG8 |  |  |  |  |
| DLA_LG8_007240 | LG8 |  |  |  |  |
| DLA_LG8_007270 | LG8 |  |  |  |  |
| DLA_LG8_007280 | LG8 |  |  |  |  |
| DLA_LG8_007290 | LG8 |  |  |  |  |
| DLA_LG8_007300 | LG8 |  |  |  |  |
| DLA_LG8_007310 | LG8 |  |  |  |  |
| DLA_LG8_007320 | LG8 |  |  |  |  |
| DLA_LG8_007330 | LG8 |  |  |  |  |
| DLA_LG8_007350 | LG8 |  |  |  |  |
| DLA_LG8_007370 | LG8 |  |  |  |  |
| DLA_LG8_007380 | LG8 |  |  |  |  |
| DLA_LG8_007385 | LG8 |  |  |  |  |
| DLA_LG8_007390 | LG8 |  |  |  |  |
| DLA_LG8_007400 | LG8 |  |  |  |  |
| DLA_LG8_007410 | LG8 |  |  |  |  |
| DLA_LG8_007420 | LG8 |  |  |  |  |
| DLA_LG8_007430 | LG8 |  |  |  |  |
| DLA_LG8_007440 | LG8 |  |  |  |  |
| DLA_LG8_007460 | LG8 |  |  |  |  |
| DLA_LG8_007470 | LG8 |  |  |  |  |
| DLA_LG8_007480 | LG8 |  |  |  |  |
| DLA_LG8_007510 | LG8 |  |  |  |  |
| DLA_LG8_007520 | LG8 |  |  |  |  |
| DLA_LG8_007530 | LG8 |  |  |  |  |
| DLA_LG8_007540 | LG8 |  |  |  |  |
| DLA_LG8_007560 | LG8 |  |  |  |  |
| DLA_LG8_007570 | LG8 |  |  |  |  |
| DLA_LG8_007590 | LG8 |  |  |  |  |
| DLA_LG8_007600 | LG8 |  |  |  |  |
| DLA_LG8_007610 | LG8 |  |  |  |  |
| DLA_LG8_007620 | LG8 |  |  |  |  |
| DLA_LG8_007630 | LG8 |  |  |  |  |
| DLA_LG8_007640 | LG8 |  |  |  |  |
| DLA_LG8_007650 | LG8 |  |  |  |  |
| DLA_LG8_007670 | LG8 |  |  |  |  |
| DLA_LG8_007680 | LG8 |  |  |  |  |
| DLA_LG8_007690 | LG8 |  |  |  |  |
| DLA_LG8_007700 | LG8 |  |  |  |  |
| DLA_LG8_007710 | LG8 |  |  |  |  |
| DLA_LG8_007730 | LG8 |  |  |  |  |
| DLA_LG8_007740 | LG8 |  |  |  |  |
| DLA_LG8_007750 | LG8 |  |  |  |  |
| DLA_LG8_007760 | LG8 |  |  |  |  |
| DLA_LG8_007770 | LG8 |  |  |  |  |
| DLA_LG8_007780 | LG8 |  |  |  |  |
| DLA_LG8_007790 | LG8 |  |  |  |  |
| DLA_LG8_007820 | LG8 |  |  |  |  |
| DLA_LG8_007840 | LG8 |  |  |  |  |
| DLA_LG8_007850 | LG8 |  |  |  |  |
| DLA_LG8_007860 | LG8 |  |  |  |  |
| DLA_LG8_007890 | LG8 |  |  |  |  |
| DLA_LG8_007900 | LG8 |  |  |  |  |
| DLA_LG8_007910 | LG8 |  |  |  |  |
| DLA_LG8_007920 | LG8 |  |  |  |  |
| DLA_LG8_007930 | LG8 |  |  |  |  |
| DLA_LG8_007940 | LG8 |  |  |  |  |
| DLA_LG8_007950 | LG8 |  |  |  |  |
| DLA_LG8_007960 | LG8 |  |  |  |  |
| DLA_LG8_007965 | LG8 |  |  |  |  |
| DLA_LG8_007970 | LG8 |  |  |  |  |
| DLA_LG8_007980 | LG8 |  |  |  |  |
| DLA_LG8_007990 | LG8 |  |  |  |  |
| DLA_LG8_008000 | LG8 |  |  |  |  |
| DLA_LG8_008010 | LG8 |  |  |  |  |
| DLA_LG8_008020 | LG8 |  |  |  |  |
| DLA_LG8_008030 | LG8 |  |  |  |  |
| DLA_LG8_008040 | LG8 |  |  |  |  |
| DLA_LG8_008050 | LG8 |  |  |  |  |
| DLA_LG8_008060 | LG8 |  |  |  |  |
| DLA_LG8_008070 | LG8 |  |  |  |  |
| DLA_LG8_008080 | LG8 |  |  |  |  |
| DLA_LG8_008085 | LG8 |  |  |  |  |
| DLA_LG8_008095_3 | LG8 |  |  |  |  |
| DLA_LG8_008095_4 | LG8 |  |  |  |  |
| DLA_LG8_008095_6 | LG8 |  |  |  |  |
| DLA_LG8_008100 | LG8 |  |  |  |  |
| DLA_LG8_008120 | LG8 |  |  |  |  |
| DLA_LG8_008130 | LG8 |  |  |  |  |
| DLA_LG8_008150 | LG8 |  |  |  |  |
| DLA_LG8_008160 | LG8 |  |  |  |  |
| DLA_LG8_008170 | LG8 |  |  |  |  |
| DLA_LG8_008180 | LG8 |  |  |  |  |
| DLA_LG8_008190 | LG8 |  |  |  |  |
| DLA_LG8_008200 | LG8 |  |  |  |  |
| DLA_LG8_008210 | LG8 |  |  |  |  |
| DLA_LG8_008220 | LG8 |  |  |  |  |
| DLA_LG8_008225_2 | LG8 |  |  |  |  |
| DLA_LG8_008230 | LG8 |  |  |  |  |
| DLA_LG8_008235 | LG8 |  |  |  |  |
| DLA_LG8_008250 | LG8 |  |  |  |  |
| DLA_LG8_008260 | LG8 |  |  |  |  |
| DLA_LG8_008270 | LG8 |  |  |  |  |
| DLA_LG8_008280 | LG8 |  |  |  |  |
| DLA_LG8_008285_3 | LG8 |  |  |  |  |
| DLA_LG8_008290 | LG8 |  |  |  |  |
| DLA_LG8_008300 | LG8 |  |  |  |  |
| DLA_LG8_008320 | LG8 |  |  |  |  |
| DLA_LG8_008330 | LG8 |  |  |  |  |
| DLA_LG8_008340 | LG8 |  |  |  |  |
| DLA_LG8_008350 | LG8 |  |  |  |  |
| DLA_LG8_008355 | LG8 |  |  |  |  |
| DLA_LG8_008355_2 | LG8 |  |  |  |  |
| DLA_LG8_008360 | LG8 |  |  |  |  |
| DLA_LG8_008380 | LG8 |  |  |  |  |
| DLA_LG8_008390 | LG8 |  |  |  |  |
| DLA_LG8_008395 | LG8 |  |  |  |  |
| DLA_LG8_008400 | LG8 |  |  |  |  |
| DLA_LG8_008405 | LG8 |  |  |  |  |
| DLA_LG8_008410 | LG8 |  |  |  |  |
| DLA_LG8_008420 | LG8 |  |  |  |  |
| DLA_LG8_008430 | LG8 |  |  |  |  |
| DLA_LG8_008440 | LG8 |  |  |  |  |
| DLA_LG8_008450 | LG8 |  |  |  |  |
| DLA_LG8_008455 | LG8 |  |  |  |  |
| DLA_LG8_008470 | LG8 |  |  |  |  |
| DLA_LG8_008480 | LG8 |  |  |  |  |
| DLA_LG8_008490 | LG8 |  |  |  |  |
| DLA_LG8_008510 | LG8 |  |  |  |  |
| DLA_LG8_008520 | LG8 |  |  |  |  |
| DLA_LG8_008530 | LG8 |  |  |  |  |
| DLA_LG8_008540 | LG8 |  |  |  |  |
| DLA_LG8_008550 | LG8 |  |  |  |  |
| DLA_LG8_008560 | LG8 |  |  |  |  |
| DLA_LG8_008570 | LG8 |  |  |  |  |
| DLA_LG8_008595 | LG8 |  |  |  |  |
| DLA_LG8_008600 | LG8 |  |  |  |  |
| DLA_LG8_008620 | LG8 |  |  |  |  |
| DLA_LG8_008630 | LG8 |  |  |  |  |
| DLA_LG8_008640 | LG8 |  |  |  |  |
| DLA_LG8_008650 | LG8 |  |  |  |  |
| DLA_LG8_008660 | LG8 |  |  |  |  |
| DLA_LG8_008680 | LG8 |  |  |  |  |
| DLA_LG8_008690 | LG8 |  |  |  |  |
| DLA_LG8_008700 | LG8 |  |  |  |  |
| DLA_LG8_008710 | LG8 |  |  |  |  |
| DLA_LG8_008720 | LG8 |  |  |  |  |
| DLA_LG8_008740 | LG8 |  |  |  |  |
| DLA_LG8_008750 | LG8 |  |  |  |  |
| DLA_LG8_008770 | LG8 |  |  |  |  |
| DLA_LG8_008780 | LG8 |  |  |  |  |
| DLA_LG8_008790 | LG8 |  |  |  |  |
| DLA_LG8_008800 | LG8 |  |  |  |  |
| DLA_LG8_008810 | LG8 |  |  |  |  |
| DLA_LG8_008820 | LG8 |  |  |  |  |
| DLA_LG8_008840 | LG8 |  |  |  |  |
| DLA_LG8_008845 | LG8 |  |  |  |  |
| DLA_LG8_008850 | LG8 |  |  |  |  |
| DLA_LG8_008860 | LG8 |  |  |  |  |
| DLA_LG8_008870 | LG8 |  |  |  |  |
| DLA_LG8_008880 | LG8 |  |  |  |  |
| DLA_LG8_008890 | LG8 |  |  |  |  |
| DLA_LG8_008900 | LG8 |  |  |  |  |
| DLA_LG8_008910 | LG8 |  |  |  |  |
| DLA_LG8_008920 | LG8 |  |  |  |  |
| DLA_LG8_008930 | LG8 |  |  |  |  |
| DLA_LG8_008940 | LG8 |  |  |  |  |
| DLA_LG8_008950 | LG8 |  |  |  |  |
| DLA_LG8_008960 | LG8 |  |  |  |  |
| DLA_LG8_008970 | LG8 |  |  |  |  |
| DLA_LG8_008980 | LG8 |  |  |  |  |
| DLA_LG8_008990 | LG8 |  |  |  |  |
| DLA_LG8_009010 | LG8 |  |  |  |  |
| DLA_LG8_009020 | LG8 |  |  |  |  |
| DLA_LG8_009025 | LG8 |  |  |  |  |
| DLA_LG8_009030 | LG8 |  |  |  |  |
| DLA_LG8_009040 | LG8 |  |  |  |  |
| DLA_LG8_009050 | LG8 |  |  |  |  |
| DLA_LG8_009060 | LG8 |  |  |  |  |
| DLA_LG8_009070 | LG8 |  |  |  |  |
| DLA_LG8_009080 | LG8 |  |  |  |  |
| DLA_LG8_009090 | LG8 |  |  |  |  |
| DLA_LG8_009100 | LG8 |  |  |  |  |
| DLA_LG8_009110 | LG8 |  |  |  |  |
| DLA_LG8_009120 | LG8 |  |  |  |  |
| DLA_LG8_009130 | LG8 |  |  |  |  |
| DLA_LG8_009140 | LG8 |  |  |  |  |
| DLA_LG8_009145_2 | LG8 |  |  |  |  |
| DLA_LG8_009150 | LG8 |  |  |  |  |
| DLA_LG8_009160 | LG8 |  |  |  |  |
| DLA_LG8_009170 | LG8 |  |  |  |  |
| DLA_LG8_009180 | LG8 |  |  |  |  |
| DLA_LG8_009190 | LG8 |  |  |  |  |
| DLA_LG8_009200 | LG8 |  |  |  |  |
| DLA_LG8_009210 | LG8 |  |  |  |  |
| DLA_LG8_009215 | LG8 |  |  |  |  |
| DLA_LG8_009220 | LG8 |  |  |  |  |
| DLA_LG8_009230 | LG8 |  |  |  |  |
| DLA_LG8_009240 | LG8 |  |  |  |  |
| DLA_LG8_009250 | LG8 |  |  |  |  |
| DLA_LG8_009260 | LG8 |  |  |  |  |
| DLA_LG8_009270 | LG8 |  |  |  |  |
| DLA_LG8_009290 | LG8 |  |  |  |  |
| DLA_LG8_009300 | LG8 |  |  |  |  |
| DLA_LG8_009310 | LG8 |  |  |  |  |
| DLA_LG8_009320 | LG8 |  |  |  |  |
| DLA_LG8_009330 | LG8 |  |  |  |  |
| DLA_LG8_009340 | LG8 |  |  |  |  |
| DLA_LG8_009350 | LG8 |  |  |  |  |
| DLA_LG8_009360 | LG8 |  |  |  |  |
| DLA_LG8_009370 | LG8 |  |  |  |  |
| DLA_LG8_009380 | LG8 |  |  |  |  |
| DLA_LG8_009390 | LG8 |  |  |  |  |
| DLA_LG8_009400 | LG8 |  |  |  |  |
| DLA_LG8_009410 | LG8 |  |  |  |  |
| DLA_LG8_009420 | LG8 |  |  |  |  |
| DLA_LG8_009425 | LG8 |  |  |  |  |
| DLA_LG8_009425_2 | LG8 |  |  |  |  |
| DLA_LG8_009430 | LG8 |  |  |  |  |
| DLA_LG8_009440 | LG8 |  |  |  |  |
| DLA_LG8_009450 | LG8 |  |  |  |  |
| DLA_LG8_009460 | LG8 |  |  |  |  |
| DLA_LG8_009480 | LG8 |  |  |  |  |
| DLA_LG8_009490 | LG8 |  |  |  |  |
| DLA_LG8_009495 | LG8 |  |  |  |  |
| DLA_LG8_009500 | LG8 |  |  |  |  |
| DLA_LG8_009510 | LG8 |  |  |  |  |
| DLA_LG8_009530 | LG8 |  |  |  |  |
| DLA_LG8_009540 | LG8 |  |  |  |  |
| DLA_LG8_009550 | LG8 |  |  |  |  |
| DLA_LG8_009560 | LG8 |  |  |  |  |
| DLA_LG8_009570 | LG8 |  |  |  |  |
| DLA_LG8_009580 | LG8 |  |  |  |  |
| DLA_LG8_009585 | LG8 |  |  |  |  |
| DLA_LG8_009590 | LG8 |  |  |  |  |
| DLA_LG8_009600 | LG8 |  |  |  |  |
| DLA_LG8_009620 | LG8 |  |  |  |  |
| DLA_LG8_009625 | LG8 |  |  |  |  |
| DLA_LG8_009630 | LG8 |  |  |  |  |
| DLA_LG8_009640 | LG8 |  |  |  |  |
| DLA_LG8_009650 | LG8 |  |  |  |  |
| DLA_LG8_009660 | LG8 |  |  |  |  |
| DLA_LG8_009670 | LG8 |  |  |  |  |
| DLA_LG8_009680 | LG8 |  |  |  |  |
| DLA_LG8_009690 | LG8 |  |  |  |  |
| DLA_LG9_000590 | LG9 |  |  |  |  |
| DLA_LG9_001020 | LG9 |  |  |  |  |
| DLA_LG9_003290 | LG9 |  |  |  |  |
| DLA_LG9_003810 | LG9 |  |  |  |  |
| DLA_LG9_004220 | LG9 |  |  |  |  |
| DLA_LG9_004510 | LG9 |  |  |  |  |
| DLA_LG9_005320 | LG9 |  |  |  |  |
| DLA_LG9_006480 | LG9 |  |  |  |  |
| DLA_LG9_000100 | LG9 |  |  |  |  |
| DLA_LG9_000170 | LG9 |  |  |  |  |
| DLA_LG9_000230 | LG9 |  |  |  |  |
| DLA_LG9_000440 | LG9 |  |  |  |  |
| DLA_LG9_000460 | LG9 |  |  |  |  |
| DLA_LG9_000490 | LG9 |  |  |  |  |
| DLA_LG9_000600 | LG9 |  |  |  |  |
| DLA_LG9_000660 | LG9 |  |  |  |  |
| DLA_LG9_000690 | LG9 |  |  |  |  |
| DLA_LG9_000705 | LG9 |  |  |  |  |
| DLA_LG9_000900 | LG9 |  |  |  |  |
| DLA_LG9_000970 | LG9 |  |  |  |  |
| DLA_LG9_001070 | LG9 |  |  |  |  |
| DLA_LG9_001160 | LG9 |  |  |  |  |
| DLA_LG9_001240 | LG9 |  |  |  |  |
| DLA_LG9_001260 | LG9 |  |  |  |  |
| DLA_LG9_001350 | LG9 |  |  |  |  |
| DLA_LG9_001660 | LG9 |  |  |  |  |
| DLA_LG9_001680 | LG9 |  |  |  |  |
| DLA_LG9_001720 | LG9 |  |  |  |  |
| DLA_LG9_001790 | LG9 |  |  |  |  |
| DLA_LG9_002080 | LG9 |  |  |  |  |
| DLA_LG9_002100 | LG9 |  |  |  |  |
| DLA_LG9_002120 | LG9 |  |  |  |  |
| DLA_LG9_002480 | LG9 |  |  |  |  |
| DLA_LG9_002620 | LG9 |  |  |  |  |
| DLA_LG9_002670 | LG9 |  |  |  |  |
| DLA_LG9_002720 | LG9 |  |  |  |  |
| DLA_LG9_002790 | LG9 |  |  |  |  |
| DLA_LG9_002990 | LG9 |  |  |  |  |
| DLA_LG9_003150 | LG9 |  |  |  |  |
| DLA_LG9_003160 | LG9 |  |  |  |  |
| DLA_LG9_003200 | LG9 |  |  |  |  |
| DLA_LG9_003370 | LG9 |  |  |  |  |
| DLA_LG9_003530 | LG9 |  |  |  |  |
| DLA_LG9_003600 | LG9 |  |  |  |  |
| DLA_LG9_003890 | LG9 |  |  |  |  |
| DLA_LG9_004100 | LG9 |  |  |  |  |
| DLA_LG9_004500 | LG9 |  |  |  |  |
| DLA_LG9_004530 | LG9 |  |  |  |  |
| DLA_LG9_004580 | LG9 |  |  |  |  |
| DLA_LG9_004710 | LG9 |  |  |  |  |
| DLA_LG9_004800 | LG9 |  |  |  |  |
| DLA_LG9_004825 | LG9 |  |  |  |  |
| DLA_LG9_005050 | LG9 |  |  |  |  |
| DLA_LG9_005145 | LG9 |  |  |  |  |
| DLA_LG9_005340 | LG9 |  |  |  |  |
| DLA_LG9_005350 | LG9 |  |  |  |  |
| DLA_LG9_005490 | LG9 |  |  |  |  |
| DLA_LG9_005710 | LG9 |  |  |  |  |
| DLA_LG9_005725 | LG9 |  |  |  |  |
| DLA_LG9_005830 | LG9 |  |  |  |  |
| DLA_LG9_005840 | LG9 |  |  |  |  |
| DLA_LG9_005930 | LG9 |  |  |  |  |
| DLA_LG9_006060 | LG9 |  |  |  |  |
| DLA_LG9_006090 | LG9 |  |  |  |  |
| DLA_LG9_006290 | LG9 |  |  |  |  |
| DLA_LG9_006360 | LG9 |  |  |  |  |
| DLA_LG9_006440 | LG9 |  |  |  |  |
| DLA_LG9_006570 | LG9 |  |  |  |  |
| DLA_LG9_006760 | LG9 |  |  |  |  |
| DLA_LG9_000010 | LG9 |  |  |  |  |
| DLA_LG9_000020 | LG9 |  |  |  |  |
| DLA_LG9_000030 | LG9 |  |  |  |  |
| DLA_LG9_000040 | LG9 |  |  |  |  |
| DLA_LG9_000050 | LG9 |  |  |  |  |
| DLA_LG9_000060 | LG9 |  |  |  |  |
| DLA_LG9_000070 | LG9 |  |  |  |  |
| DLA_LG9_000080 | LG9 |  |  |  |  |
| DLA_LG9_000090 | LG9 |  |  |  |  |
| DLA_LG9_000110 | LG9 |  |  |  |  |
| DLA_LG9_000130 | LG9 |  |  |  |  |
| DLA_LG9_000135 | LG9 |  |  |  |  |
| DLA_LG9_000140 | LG9 |  |  |  |  |
| DLA_LG9_000150 | LG9 |  |  |  |  |
| DLA_LG9_000160 | LG9 |  |  |  |  |
| DLA_LG9_000180 | LG9 |  |  |  |  |
| DLA_LG9_000190 | LG9 |  |  |  |  |
| DLA_LG9_000200 | LG9 |  |  |  |  |
| DLA_LG9_000210 | LG9 |  |  |  |  |
| DLA_LG9_000220 | LG9 |  |  |  |  |
| DLA_LG9_000240 | LG9 |  |  |  |  |
| DLA_LG9_000250 | LG9 |  |  |  |  |
| DLA_LG9_000260 | LG9 |  |  |  |  |
| DLA_LG9_000270 | LG9 |  |  |  |  |
| DLA_LG9_000280 | LG9 |  |  |  |  |
| DLA_LG9_000290 | LG9 |  |  |  |  |
| DLA_LG9_000300 | LG9 |  |  |  |  |
| DLA_LG9_000310 | LG9 |  |  |  |  |
| DLA_LG9_000320 | LG9 |  |  |  |  |
| DLA_LG9_000330 | LG9 |  |  |  |  |
| DLA_LG9_000340 | LG9 |  |  |  |  |
| DLA_LG9_000350 | LG9 |  |  |  |  |
| DLA_LG9_000360 | LG9 |  |  |  |  |
| DLA_LG9_000370 | LG9 |  |  |  |  |
| DLA_LG9_000380 | LG9 |  |  |  |  |
| DLA_LG9_000390 | LG9 |  |  |  |  |
| DLA_LG9_000400 | LG9 |  |  |  |  |
| DLA_LG9_000410 | LG9 |  |  |  |  |
| DLA_LG9_000420 | LG9 |  |  |  |  |
| DLA_LG9_000430 | LG9 |  |  |  |  |
| DLA_LG9_000450 | LG9 |  |  |  |  |
| DLA_LG9_000470 | LG9 |  |  |  |  |
| DLA_LG9_000480 | LG9 |  |  |  |  |
| DLA_LG9_000510 | LG9 |  |  |  |  |
| DLA_LG9_000520 | LG9 |  |  |  |  |
| DLA_LG9_000525 | LG9 |  |  |  |  |
| DLA_LG9_000525_2 | LG9 |  |  |  |  |
| DLA_LG9_000530 | LG9 |  |  |  |  |
| DLA_LG9_000540 | LG9 |  |  |  |  |
| DLA_LG9_000550 | LG9 |  |  |  |  |
| DLA_LG9_000555 | LG9 |  |  |  |  |
| DLA_LG9_000560 | LG9 |  |  |  |  |
| DLA_LG9_000570 | LG9 |  |  |  |  |
| DLA_LG9_000580 | LG9 |  |  |  |  |
| DLA_LG9_000595 | LG9 |  |  |  |  |
| DLA_LG9_000610 | LG9 |  |  |  |  |
| DLA_LG9_000620 | LG9 |  |  |  |  |
| DLA_LG9_000630 | LG9 |  |  |  |  |
| DLA_LG9_000635 | LG9 |  |  |  |  |
| DLA_LG9_000640 | LG9 |  |  |  |  |
| DLA_LG9_000650 | LG9 |  |  |  |  |
| DLA_LG9_000680 | LG9 |  |  |  |  |
| DLA_LG9_000710 | LG9 |  |  |  |  |
| DLA_LG9_000720 | LG9 |  |  |  |  |
| DLA_LG9_000730 | LG9 |  |  |  |  |
| DLA_LG9_000740 | LG9 |  |  |  |  |
| DLA_LG9_000750 | LG9 |  |  |  |  |
| DLA_LG9_000760 | LG9 |  |  |  |  |
| DLA_LG9_000770 | LG9 |  |  |  |  |
| DLA_LG9_000780 | LG9 |  |  |  |  |
| DLA_LG9_000800 | LG9 |  |  |  |  |
| DLA_LG9_000810 | LG9 |  |  |  |  |
| DLA_LG9_000820 | LG9 |  |  |  |  |
| DLA_LG9_000830 | LG9 |  |  |  |  |
| DLA_LG9_000835 | LG9 |  |  |  |  |
| DLA_LG9_000840 | LG9 |  |  |  |  |
| DLA_LG9_000850 | LG9 |  |  |  |  |
| DLA_LG9_000860 | LG9 |  |  |  |  |
| DLA_LG9_000870 | LG9 |  |  |  |  |
| DLA_LG9_000890 | LG9 |  |  |  |  |
| DLA_LG9_000895 | LG9 |  |  |  |  |
| DLA_LG9_000910 | LG9 |  |  |  |  |
| DLA_LG9_000920 | LG9 |  |  |  |  |
| DLA_LG9_000930 | LG9 |  |  |  |  |
| DLA_LG9_000940 | LG9 |  |  |  |  |
| DLA_LG9_000950 | LG9 |  |  |  |  |
| DLA_LG9_000960 | LG9 |  |  |  |  |
| DLA_LG9_000980 | LG9 |  |  |  |  |
| DLA_LG9_000990 | LG9 |  |  |  |  |
| DLA_LG9_001000 | LG9 |  |  |  |  |
| DLA_LG9_001010 | LG9 |  |  |  |  |
| DLA_LG9_001040 | LG9 |  |  |  |  |
| DLA_LG9_001050 | LG9 |  |  |  |  |
| DLA_LG9_001060 | LG9 |  |  |  |  |
| DLA_LG9_001080 | LG9 |  |  |  |  |
| DLA_LG9_001085 | LG9 |  |  |  |  |
| DLA_LG9_001090 | LG9 |  |  |  |  |
| DLA_LG9_001100 | LG9 |  |  |  |  |
| DLA_LG9_001110 | LG9 |  |  |  |  |
| DLA_LG9_001120 | LG9 |  |  |  |  |
| DLA_LG9_001130 | LG9 |  |  |  |  |
| DLA_LG9_001140 | LG9 |  |  |  |  |
| DLA_LG9_001150 | LG9 |  |  |  |  |
| DLA_LG9_001155 | LG9 |  |  |  |  |
| DLA_LG9_001155_2 | LG9 |  |  |  |  |
| DLA_LG9_001170 | LG9 |  |  |  |  |
| DLA_LG9_001180 | LG9 |  |  |  |  |
| DLA_LG9_001190 | LG9 |  |  |  |  |
| DLA_LG9_001200 | LG9 |  |  |  |  |
| DLA_LG9_001220 | LG9 |  |  |  |  |
| DLA_LG9_001245_2 | LG9 |  |  |  |  |
| DLA_LG9_001250 | LG9 |  |  |  |  |
| DLA_LG9_001270 | LG9 |  |  |  |  |
| DLA_LG9_001280 | LG9 |  |  |  |  |
| DLA_LG9_001290 | LG9 |  |  |  |  |
| DLA_LG9_001300 | LG9 |  |  |  |  |
| DLA_LG9_001310 | LG9 |  |  |  |  |
| DLA_LG9_001320 | LG9 |  |  |  |  |
| DLA_LG9_001340 | LG9 |  |  |  |  |
| DLA_LG9_001360 | LG9 |  |  |  |  |
| DLA_LG9_001370 | LG9 |  |  |  |  |
| DLA_LG9_001375 | LG9 |  |  |  |  |
| DLA_LG9_001380 | LG9 |  |  |  |  |
| DLA_LG9_001390 | LG9 |  |  |  |  |
| DLA_LG9_001395 | LG9 |  |  |  |  |
| DLA_LG9_001400 | LG9 |  |  |  |  |
| DLA_LG9_001405_2 | LG9 |  |  |  |  |
| DLA_LG9_001410 | LG9 |  |  |  |  |
| DLA_LG9_001420 | LG9 |  |  |  |  |
| DLA_LG9_001430 | LG9 |  |  |  |  |
| DLA_LG9_001440 | LG9 |  |  |  |  |
| DLA_LG9_001450 | LG9 |  |  |  |  |
| DLA_LG9_001460 | LG9 |  |  |  |  |
| DLA_LG9_001470 | LG9 |  |  |  |  |
| DLA_LG9_001480 | LG9 |  |  |  |  |
| DLA_LG9_001490 | LG9 |  |  |  |  |
| DLA_LG9_001495 | LG9 |  |  |  |  |
| DLA_LG9_001500 | LG9 |  |  |  |  |
| DLA_LG9_001510 | LG9 |  |  |  |  |
| DLA_LG9_001520 | LG9 |  |  |  |  |
| DLA_LG9_001530 | LG9 |  |  |  |  |
| DLA_LG9_001540 | LG9 |  |  |  |  |
| DLA_LG9_001560 | LG9 |  |  |  |  |
| DLA_LG9_001570 | LG9 |  |  |  |  |
| DLA_LG9_001580 | LG9 |  |  |  |  |
| DLA_LG9_001590 | LG9 |  |  |  |  |
| DLA_LG9_001600 | LG9 |  |  |  |  |
| DLA_LG9_001610 | LG9 |  |  |  |  |
| DLA_LG9_001620 | LG9 |  |  |  |  |
| DLA_LG9_001630 | LG9 |  |  |  |  |
| DLA_LG9_001640 | LG9 |  |  |  |  |
| DLA_LG9_001650 | LG9 |  |  |  |  |
| DLA_LG9_001670 | LG9 |  |  |  |  |
| DLA_LG9_001690 | LG9 |  |  |  |  |
| DLA_LG9_001700 | LG9 |  |  |  |  |
| DLA_LG9_001710 | LG9 |  |  |  |  |
| DLA_LG9_001730 | LG9 |  |  |  |  |
| DLA_LG9_001740 | LG9 |  |  |  |  |
| DLA_LG9_001750 | LG9 |  |  |  |  |
| DLA_LG9_001760 | LG9 |  |  |  |  |
| DLA_LG9_001770 | LG9 |  |  |  |  |
| DLA_LG9_001780 | LG9 |  |  |  |  |
| DLA_LG9_001800 | LG9 |  |  |  |  |
| DLA_LG9_001810 | LG9 |  |  |  |  |
| DLA_LG9_001820 | LG9 |  |  |  |  |
| DLA_LG9_001830 | LG9 |  |  |  |  |
| DLA_LG9_001850 | LG9 |  |  |  |  |
| DLA_LG9_001860 | LG9 |  |  |  |  |
| DLA_LG9_001870 | LG9 |  |  |  |  |
| DLA_LG9_001880 | LG9 |  |  |  |  |
| DLA_LG9_001890 | LG9 |  |  |  |  |
| DLA_LG9_001900 | LG9 |  |  |  |  |
| DLA_LG9_001910 | LG9 |  |  |  |  |
| DLA_LG9_001920 | LG9 |  |  |  |  |
| DLA_LG9_001930 | LG9 |  |  |  |  |
| DLA_LG9_001940 | LG9 |  |  |  |  |
| DLA_LG9_001950 | LG9 |  |  |  |  |
| DLA_LG9_001955 | LG9 |  |  |  |  |
| DLA_LG9_001960 | LG9 |  |  |  |  |
| DLA_LG9_001970 | LG9 |  |  |  |  |
| DLA_LG9_001980 | LG9 |  |  |  |  |
| DLA_LG9_001985 | LG9 |  |  |  |  |
| DLA_LG9_001990 | LG9 |  |  |  |  |
| DLA_LG9_002000 | LG9 |  |  |  |  |
| DLA_LG9_002010 | LG9 |  |  |  |  |
| DLA_LG9_002020 | LG9 |  |  |  |  |
| DLA_LG9_002030 | LG9 |  |  |  |  |
| DLA_LG9_002060 | LG9 |  |  |  |  |
| DLA_LG9_002070 | LG9 |  |  |  |  |
| DLA_LG9_002090 | LG9 |  |  |  |  |
| DLA_LG9_002110 | LG9 |  |  |  |  |
| DLA_LG9_002125 | LG9 |  |  |  |  |
| DLA_LG9_002130 | LG9 |  |  |  |  |
| DLA_LG9_002135 | LG9 |  |  |  |  |
| DLA_LG9_002140 | LG9 |  |  |  |  |
| DLA_LG9_002150 | LG9 |  |  |  |  |
| DLA_LG9_002160 | LG9 |  |  |  |  |
| DLA_LG9_002170 | LG9 |  |  |  |  |
| DLA_LG9_002180 | LG9 |  |  |  |  |
| DLA_LG9_002190 | LG9 |  |  |  |  |
| DLA_LG9_002200 | LG9 |  |  |  |  |
| DLA_LG9_002210 | LG9 |  |  |  |  |
| DLA_LG9_002220 | LG9 |  |  |  |  |
| DLA_LG9_002230 | LG9 |  |  |  |  |
| DLA_LG9_002240 | LG9 |  |  |  |  |
| DLA_LG9_002250 | LG9 |  |  |  |  |
| DLA_LG9_002260 | LG9 |  |  |  |  |
| DLA_LG9_002270 | LG9 |  |  |  |  |
| DLA_LG9_002280 | LG9 |  |  |  |  |
| DLA_LG9_002290 | LG9 |  |  |  |  |
| DLA_LG9_002300 | LG9 |  |  |  |  |
| DLA_LG9_002310 | LG9 |  |  |  |  |
| DLA_LG9_002320 | LG9 |  |  |  |  |
| DLA_LG9_002330 | LG9 |  |  |  |  |
| DLA_LG9_002340 | LG9 |  |  |  |  |
| DLA_LG9_002350 | LG9 |  |  |  |  |
| DLA_LG9_002360 | LG9 |  |  |  |  |
| DLA_LG9_002370 | LG9 |  |  |  |  |
| DLA_LG9_002380 | LG9 |  |  |  |  |
| DLA_LG9_002390 | LG9 |  |  |  |  |
| DLA_LG9_002400 | LG9 |  |  |  |  |
| DLA_LG9_002410 | LG9 |  |  |  |  |
| DLA_LG9_002420 | LG9 |  |  |  |  |
| DLA_LG9_002430 | LG9 |  |  |  |  |
| DLA_LG9_002450 | LG9 |  |  |  |  |
| DLA_LG9_002460 | LG9 |  |  |  |  |
| DLA_LG9_002470 | LG9 |  |  |  |  |
| DLA_LG9_002490 | LG9 |  |  |  |  |
| DLA_LG9_002500 | LG9 |  |  |  |  |
| DLA_LG9_002510 | LG9 |  |  |  |  |
| DLA_LG9_002520 | LG9 |  |  |  |  |
| DLA_LG9_002530 | LG9 |  |  |  |  |
| DLA_LG9_002540 | LG9 |  |  |  |  |
| DLA_LG9_002550 | LG9 |  |  |  |  |
| DLA_LG9_002560 | LG9 |  |  |  |  |
| DLA_LG9_002570 | LG9 |  |  |  |  |
| DLA_LG9_002580 | LG9 |  |  |  |  |
| DLA_LG9_002590 | LG9 |  |  |  |  |
| DLA_LG9_002600 | LG9 |  |  |  |  |
| DLA_LG9_002610 | LG9 |  |  |  |  |
| DLA_LG9_002630 | LG9 |  |  |  |  |
| DLA_LG9_002640 | LG9 |  |  |  |  |
| DLA_LG9_002650 | LG9 |  |  |  |  |
| DLA_LG9_002660 | LG9 |  |  |  |  |
| DLA_LG9_002680 | LG9 |  |  |  |  |
| DLA_LG9_002690 | LG9 |  |  |  |  |
| DLA_LG9_002700 | LG9 |  |  |  |  |
| DLA_LG9_002710 | LG9 |  |  |  |  |
| DLA_LG9_002730 | LG9 |  |  |  |  |
| DLA_LG9_002740 | LG9 |  |  |  |  |
| DLA_LG9_002750 | LG9 |  |  |  |  |
| DLA_LG9_002760 | LG9 |  |  |  |  |
| DLA_LG9_002770 | LG9 |  |  |  |  |
| DLA_LG9_002780 | LG9 |  |  |  |  |
| DLA_LG9_002800 | LG9 |  |  |  |  |
| DLA_LG9_002810 | LG9 |  |  |  |  |
| DLA_LG9_002820 | LG9 |  |  |  |  |
| DLA_LG9_002830 | LG9 |  |  |  |  |
| DLA_LG9_002840 | LG9 |  |  |  |  |
| DLA_LG9_002850 | LG9 |  |  |  |  |
| DLA_LG9_002855 | LG9 |  |  |  |  |
| DLA_LG9_002860 | LG9 |  |  |  |  |
| DLA_LG9_002890 | LG9 |  |  |  |  |
| DLA_LG9_002900 | LG9 |  |  |  |  |
| DLA_LG9_002920 | LG9 |  |  |  |  |
| DLA_LG9_002930 | LG9 |  |  |  |  |
| DLA_LG9_002940 | LG9 |  |  |  |  |
| DLA_LG9_002950 | LG9 |  |  |  |  |
| DLA_LG9_002980 | LG9 |  |  |  |  |
| DLA_LG9_002985 | LG9 |  |  |  |  |
| DLA_LG9_003000 | LG9 |  |  |  |  |
| DLA_LG9_003010 | LG9 |  |  |  |  |
| DLA_LG9_003020 | LG9 |  |  |  |  |
| DLA_LG9_003030 | LG9 |  |  |  |  |
| DLA_LG9_003040 | LG9 |  |  |  |  |
| DLA_LG9_003050 | LG9 |  |  |  |  |
| DLA_LG9_003060 | LG9 |  |  |  |  |
| DLA_LG9_003070 | LG9 |  |  |  |  |
| DLA_LG9_003080 | LG9 |  |  |  |  |
| DLA_LG9_003090 | LG9 |  |  |  |  |
| DLA_LG9_003100 | LG9 |  |  |  |  |
| DLA_LG9_003110 | LG9 |  |  |  |  |
| DLA_LG9_003120 | LG9 |  |  |  |  |
| DLA_LG9_003130 | LG9 |  |  |  |  |
| DLA_LG9_003140 | LG9 |  |  |  |  |
| DLA_LG9_003170 | LG9 |  |  |  |  |
| DLA_LG9_003180 | LG9 |  |  |  |  |
| DLA_LG9_003185 | LG9 |  |  |  |  |
| DLA_LG9_003190 | LG9 |  |  |  |  |
| DLA_LG9_003210 | LG9 |  |  |  |  |
| DLA_LG9_003220 | LG9 |  |  |  |  |
| DLA_LG9_003230 | LG9 |  |  |  |  |
| DLA_LG9_003240 | LG9 |  |  |  |  |
| DLA_LG9_003260 | LG9 |  |  |  |  |
| DLA_LG9_003270 | LG9 |  |  |  |  |
| DLA_LG9_003280 | LG9 |  |  |  |  |
| DLA_LG9_003285 | LG9 |  |  |  |  |
| DLA_LG9_003300 | LG9 |  |  |  |  |
| DLA_LG9_003310 | LG9 |  |  |  |  |
| DLA_LG9_003320 | LG9 |  |  |  |  |
| DLA_LG9_003330 | LG9 |  |  |  |  |
| DLA_LG9_003340 | LG9 |  |  |  |  |
| DLA_LG9_003350 | LG9 |  |  |  |  |
| DLA_LG9_003360 | LG9 |  |  |  |  |
| DLA_LG9_003380 | LG9 |  |  |  |  |
| DLA_LG9_003390 | LG9 |  |  |  |  |
| DLA_LG9_003400 | LG9 |  |  |  |  |
| DLA_LG9_003410 | LG9 |  |  |  |  |
| DLA_LG9_003420 | LG9 |  |  |  |  |
| DLA_LG9_003430 | LG9 |  |  |  |  |
| DLA_LG9_003440 | LG9 |  |  |  |  |
| DLA_LG9_003460 | LG9 |  |  |  |  |
| DLA_LG9_003470 | LG9 |  |  |  |  |
| DLA_LG9_003480 | LG9 |  |  |  |  |
| DLA_LG9_003485 | LG9 |  |  |  |  |
| DLA_LG9_003490 | LG9 |  |  |  |  |
| DLA_LG9_003500 | LG9 |  |  |  |  |
| DLA_LG9_003510 | LG9 |  |  |  |  |
| DLA_LG9_003520 | LG9 |  |  |  |  |
| DLA_LG9_003540 | LG9 |  |  |  |  |
| DLA_LG9_003560 | LG9 |  |  |  |  |
| DLA_LG9_003580 | LG9 |  |  |  |  |
| DLA_LG9_003590 | LG9 |  |  |  |  |
| DLA_LG9_003610 | LG9 |  |  |  |  |
| DLA_LG9_003620 | LG9 |  |  |  |  |
| DLA_LG9_003630 | LG9 |  |  |  |  |
| DLA_LG9_003640 | LG9 |  |  |  |  |
| DLA_LG9_003650 | LG9 |  |  |  |  |
| DLA_LG9_003660 | LG9 |  |  |  |  |
| DLA_LG9_003670 | LG9 |  |  |  |  |
| DLA_LG9_003680 | LG9 |  |  |  |  |
| DLA_LG9_003690 | LG9 |  |  |  |  |
| DLA_LG9_003700 | LG9 |  |  |  |  |
| DLA_LG9_003705 | LG9 |  |  |  |  |
| DLA_LG9_003710 | LG9 |  |  |  |  |
| DLA_LG9_003720 | LG9 |  |  |  |  |
| DLA_LG9_003730 | LG9 |  |  |  |  |
| DLA_LG9_003740 | LG9 |  |  |  |  |
| DLA_LG9_003750 | LG9 |  |  |  |  |
| DLA_LG9_003760 | LG9 |  |  |  |  |
| DLA_LG9_003770 | LG9 |  |  |  |  |
| DLA_LG9_003780 | LG9 |  |  |  |  |
| DLA_LG9_003790 | LG9 |  |  |  |  |
| DLA_LG9_003800 | LG9 |  |  |  |  |
| DLA_LG9_003815 | LG9 |  |  |  |  |
| DLA_LG9_003820 | LG9 |  |  |  |  |
| DLA_LG9_003830 | LG9 |  |  |  |  |
| DLA_LG9_003840 | LG9 |  |  |  |  |
| DLA_LG9_003850 | LG9 |  |  |  |  |
| DLA_LG9_003860 | LG9 |  |  |  |  |
| DLA_LG9_003870 | LG9 |  |  |  |  |
| DLA_LG9_003880 | LG9 |  |  |  |  |
| DLA_LG9_003900 | LG9 |  |  |  |  |
| DLA_LG9_003910 | LG9 |  |  |  |  |
| DLA_LG9_003920 | LG9 |  |  |  |  |
| DLA_LG9_003930 | LG9 |  |  |  |  |
| DLA_LG9_003940 | LG9 |  |  |  |  |
| DLA_LG9_003950 | LG9 |  |  |  |  |
| DLA_LG9_003960 | LG9 |  |  |  |  |
| DLA_LG9_003970 | LG9 |  |  |  |  |
| DLA_LG9_003980 | LG9 |  |  |  |  |
| DLA_LG9_003990 | LG9 |  |  |  |  |
| DLA_LG9_004000 | LG9 |  |  |  |  |
| DLA_LG9_004010 | LG9 |  |  |  |  |
| DLA_LG9_004020 | LG9 |  |  |  |  |
| DLA_LG9_004030 | LG9 |  |  |  |  |
| DLA_LG9_004040 | LG9 |  |  |  |  |
| DLA_LG9_004050 | LG9 |  |  |  |  |
| DLA_LG9_004060 | LG9 |  |  |  |  |
| DLA_LG9_004070 | LG9 |  |  |  |  |
| DLA_LG9_004080 | LG9 |  |  |  |  |
| DLA_LG9_004090 | LG9 |  |  |  |  |
| DLA_LG9_004110 | LG9 |  |  |  |  |
| DLA_LG9_004120 | LG9 |  |  |  |  |
| DLA_LG9_004130 | LG9 |  |  |  |  |
| DLA_LG9_004140 | LG9 |  |  |  |  |
| DLA_LG9_004150 | LG9 |  |  |  |  |
| DLA_LG9_004160 | LG9 |  |  |  |  |
| DLA_LG9_004170 | LG9 |  |  |  |  |
| DLA_LG9_004180 | LG9 |  |  |  |  |
| DLA_LG9_004190 | LG9 |  |  |  |  |
| DLA_LG9_004200 | LG9 |  |  |  |  |
| DLA_LG9_004205 | LG9 |  |  |  |  |
| DLA_LG9_004210 | LG9 |  |  |  |  |
| DLA_LG9_004230 | LG9 |  |  |  |  |
| DLA_LG9_004240 | LG9 |  |  |  |  |
| DLA_LG9_004245 | LG9 |  |  |  |  |
| DLA_LG9_004250 | LG9 |  |  |  |  |
| DLA_LG9_004260 | LG9 |  |  |  |  |
| DLA_LG9_004270 | LG9 |  |  |  |  |
| DLA_LG9_004280 | LG9 |  |  |  |  |
| DLA_LG9_004290 | LG9 |  |  |  |  |
| DLA_LG9_004300 | LG9 |  |  |  |  |
| DLA_LG9_004310 | LG9 |  |  |  |  |
| DLA_LG9_004320 | LG9 |  |  |  |  |
| DLA_LG9_004330 | LG9 |  |  |  |  |
| DLA_LG9_004340 | LG9 |  |  |  |  |
| DLA_LG9_004350 | LG9 |  |  |  |  |
| DLA_LG9_004355 | LG9 |  |  |  |  |
| DLA_LG9_004360 | LG9 |  |  |  |  |
| DLA_LG9_004370 | LG9 |  |  |  |  |
| DLA_LG9_004380 | LG9 |  |  |  |  |
| DLA_LG9_004390 | LG9 |  |  |  |  |
| DLA_LG9_004400 | LG9 |  |  |  |  |
| DLA_LG9_004410 | LG9 |  |  |  |  |
| DLA_LG9_004420 | LG9 |  |  |  |  |
| DLA_LG9_004430 | LG9 |  |  |  |  |
| DLA_LG9_004450 | LG9 |  |  |  |  |
| DLA_LG9_004460 | LG9 |  |  |  |  |
| DLA_LG9_004470 | LG9 |  |  |  |  |
| DLA_LG9_004480 | LG9 |  |  |  |  |
| DLA_LG9_004490 | LG9 |  |  |  |  |
| DLA_LG9_004505 | LG9 |  |  |  |  |
| DLA_LG9_004520 | LG9 |  |  |  |  |
| DLA_LG9_004540 | LG9 |  |  |  |  |
| DLA_LG9_004550 | LG9 |  |  |  |  |
| DLA_LG9_004560 | LG9 |  |  |  |  |
| DLA_LG9_004570 | LG9 |  |  |  |  |
| DLA_LG9_004590 | LG9 |  |  |  |  |
| DLA_LG9_004610 | LG9 |  |  |  |  |
| DLA_LG9_004620 | LG9 |  |  |  |  |
| DLA_LG9_004625 | LG9 |  |  |  |  |
| DLA_LG9_004630 | LG9 |  |  |  |  |
| DLA_LG9_004640 | LG9 |  |  |  |  |
| DLA_LG9_004650 | LG9 |  |  |  |  |
| DLA_LG9_004660 | LG9 |  |  |  |  |
| DLA_LG9_004670 | LG9 |  |  |  |  |
| DLA_LG9_004680 | LG9 |  |  |  |  |
| DLA_LG9_004690 | LG9 |  |  |  |  |
| DLA_LG9_004700 | LG9 |  |  |  |  |
| DLA_LG9_004705 | LG9 |  |  |  |  |
| DLA_LG9_004720 | LG9 |  |  |  |  |
| DLA_LG9_004730 | LG9 |  |  |  |  |
| DLA_LG9_004750 | LG9 |  |  |  |  |
| DLA_LG9_004755 | LG9 |  |  |  |  |
| DLA_LG9_004760 | LG9 |  |  |  |  |
| DLA_LG9_004770 | LG9 |  |  |  |  |
| DLA_LG9_004780 | LG9 |  |  |  |  |
| DLA_LG9_004790 | LG9 |  |  |  |  |
| DLA_LG9_004810 | LG9 |  |  |  |  |
| DLA_LG9_004820 | LG9 |  |  |  |  |
| DLA_LG9_004840 | LG9 |  |  |  |  |
| DLA_LG9_004850 | LG9 |  |  |  |  |
| DLA_LG9_004860 | LG9 |  |  |  |  |
| DLA_LG9_004870 | LG9 |  |  |  |  |
| DLA_LG9_004880 | LG9 |  |  |  |  |
| DLA_LG9_004890 | LG9 |  |  |  |  |
| DLA_LG9_004900 | LG9 |  |  |  |  |
| DLA_LG9_004910 | LG9 |  |  |  |  |
| DLA_LG9_004920 | LG9 |  |  |  |  |
| DLA_LG9_004930 | LG9 |  |  |  |  |
| DLA_LG9_004940 | LG9 |  |  |  |  |
| DLA_LG9_004950 | LG9 |  |  |  |  |
| DLA_LG9_004960 | LG9 |  |  |  |  |
| DLA_LG9_004970 | LG9 |  |  |  |  |
| DLA_LG9_004980 | LG9 |  |  |  |  |
| DLA_LG9_004990 | LG9 |  |  |  |  |
| DLA_LG9_005000 | LG9 |  |  |  |  |
| DLA_LG9_005010 | LG9 |  |  |  |  |
| DLA_LG9_005020 | LG9 |  |  |  |  |
| DLA_LG9_005025 | LG9 |  |  |  |  |
| DLA_LG9_005030 | LG9 |  |  |  |  |
| DLA_LG9_005040 | LG9 |  |  |  |  |
| DLA_LG9_005060 | LG9 |  |  |  |  |
| DLA_LG9_005070 | LG9 |  |  |  |  |
| DLA_LG9_005090 | LG9 |  |  |  |  |
| DLA_LG9_005100 | LG9 |  |  |  |  |
| DLA_LG9_005105 | LG9 |  |  |  |  |
| DLA_LG9_005110 | LG9 |  |  |  |  |
| DLA_LG9_005120 | LG9 |  |  |  |  |
| DLA_LG9_005130 | LG9 |  |  |  |  |
| DLA_LG9_005140 | LG9 |  |  |  |  |
| DLA_LG9_005150 | LG9 |  |  |  |  |
| DLA_LG9_005160 | LG9 |  |  |  |  |
| DLA_LG9_005170 | LG9 |  |  |  |  |
| DLA_LG9_005180 | LG9 |  |  |  |  |
| DLA_LG9_005190 | LG9 |  |  |  |  |
| DLA_LG9_005200 | LG9 |  |  |  |  |
| DLA_LG9_005210 | LG9 |  |  |  |  |
| DLA_LG9_005215_2 | LG9 |  |  |  |  |
| DLA_LG9_005220 | LG9 |  |  |  |  |
| DLA_LG9_005230 | LG9 |  |  |  |  |
| DLA_LG9_005240 | LG9 |  |  |  |  |
| DLA_LG9_005250 | LG9 |  |  |  |  |
| DLA_LG9_005270 | LG9 |  |  |  |  |
| DLA_LG9_005280 | LG9 |  |  |  |  |
| DLA_LG9_005290 | LG9 |  |  |  |  |
| DLA_LG9_005300 | LG9 |  |  |  |  |
| DLA_LG9_005310 | LG9 |  |  |  |  |
| DLA_LG9_005330 | LG9 |  |  |  |  |
| DLA_LG9_005360 | LG9 |  |  |  |  |
| DLA_LG9_005370 | LG9 |  |  |  |  |
| DLA_LG9_005380 | LG9 |  |  |  |  |
| DLA_LG9_005390 | LG9 |  |  |  |  |
| DLA_LG9_005400 | LG9 |  |  |  |  |
| DLA_LG9_005410 | LG9 |  |  |  |  |
| DLA_LG9_005420 | LG9 |  |  |  |  |
| DLA_LG9_005430 | LG9 |  |  |  |  |
| DLA_LG9_005440 | LG9 |  |  |  |  |
| DLA_LG9_005450 | LG9 |  |  |  |  |
| DLA_LG9_005460 | LG9 |  |  |  |  |
| DLA_LG9_005470 | LG9 |  |  |  |  |
| DLA_LG9_005480 | LG9 |  |  |  |  |
| DLA_LG9_005500 | LG9 |  |  |  |  |
| DLA_LG9_005510 | LG9 |  |  |  |  |
| DLA_LG9_005520 | LG9 |  |  |  |  |
| DLA_LG9_005530 | LG9 |  |  |  |  |
| DLA_LG9_005540 | LG9 |  |  |  |  |
| DLA_LG9_005550 | LG9 |  |  |  |  |
| DLA_LG9_005560 | LG9 |  |  |  |  |
| DLA_LG9_005570 | LG9 |  |  |  |  |
| DLA_LG9_005580 | LG9 |  |  |  |  |
| DLA_LG9_005590 | LG9 |  |  |  |  |
| DLA_LG9_005600 | LG9 |  |  |  |  |
| DLA_LG9_005610 | LG9 |  |  |  |  |
| DLA_LG9_005620 | LG9 |  |  |  |  |
| DLA_LG9_005630 | LG9 |  |  |  |  |
| DLA_LG9_005640 | LG9 |  |  |  |  |
| DLA_LG9_005650 | LG9 |  |  |  |  |
| DLA_LG9_005660 | LG9 |  |  |  |  |
| DLA_LG9_005670 | LG9 |  |  |  |  |
| DLA_LG9_005680 | LG9 |  |  |  |  |
| DLA_LG9_005690 | LG9 |  |  |  |  |
| DLA_LG9_005695 | LG9 |  |  |  |  |
| DLA_LG9_005700 | LG9 |  |  |  |  |
| DLA_LG9_005720 | LG9 |  |  |  |  |
| DLA_LG9_005730 | LG9 |  |  |  |  |
| DLA_LG9_005740 | LG9 |  |  |  |  |
| DLA_LG9_005750 | LG9 |  |  |  |  |
| DLA_LG9_005755 | LG9 |  |  |  |  |
| DLA_LG9_005760 | LG9 |  |  |  |  |
| DLA_LG9_005770 | LG9 |  |  |  |  |
| DLA_LG9_005780 | LG9 |  |  |  |  |
| DLA_LG9_005790 | LG9 |  |  |  |  |
| DLA_LG9_005800 | LG9 |  |  |  |  |
| DLA_LG9_005810 | LG9 |  |  |  |  |
| DLA_LG9_005820 | LG9 |  |  |  |  |
| DLA_LG9_005850 | LG9 |  |  |  |  |
| DLA_LG9_005870 | LG9 |  |  |  |  |
| DLA_LG9_005880 | LG9 |  |  |  |  |
| DLA_LG9_005900 | LG9 |  |  |  |  |
| DLA_LG9_005910 | LG9 |  |  |  |  |
| DLA_LG9_005920 | LG9 |  |  |  |  |
| DLA_LG9_005940 | LG9 |  |  |  |  |
| DLA_LG9_005950 | LG9 |  |  |  |  |
| DLA_LG9_005960 | LG9 |  |  |  |  |
| DLA_LG9_005970 | LG9 |  |  |  |  |
| DLA_LG9_005980 | LG9 |  |  |  |  |
| DLA_LG9_005990 | LG9 |  |  |  |  |
| DLA_LG9_006000 | LG9 |  |  |  |  |
| DLA_LG9_006010 | LG9 |  |  |  |  |
| DLA_LG9_006020 | LG9 |  |  |  |  |
| DLA_LG9_006025 | LG9 |  |  |  |  |
| DLA_LG9_006030 | LG9 |  |  |  |  |
| DLA_LG9_006040 | LG9 |  |  |  |  |
| DLA_LG9_006050 | LG9 |  |  |  |  |
| DLA_LG9_006070 | LG9 |  |  |  |  |
| DLA_LG9_006100 | LG9 |  |  |  |  |
| DLA_LG9_006110 | LG9 |  |  |  |  |
| DLA_LG9_006120 | LG9 |  |  |  |  |
| DLA_LG9_006130 | LG9 |  |  |  |  |
| DLA_LG9_006140 | LG9 |  |  |  |  |
| DLA_LG9_006150 | LG9 |  |  |  |  |
| DLA_LG9_006160 | LG9 |  |  |  |  |
| DLA_LG9_006170 | LG9 |  |  |  |  |
| DLA_LG9_006175 | LG9 |  |  |  |  |
| DLA_LG9_006180 | LG9 |  |  |  |  |
| DLA_LG9_006190 | LG9 |  |  |  |  |
| DLA_LG9_006200 | LG9 |  |  |  |  |
| DLA_LG9_006205 | LG9 |  |  |  |  |
| DLA_LG9_006210 | LG9 |  |  |  |  |
| DLA_LG9_006220 | LG9 |  |  |  |  |
| DLA_LG9_006230 | LG9 |  |  |  |  |
| DLA_LG9_006240 | LG9 |  |  |  |  |
| DLA_LG9_006260 | LG9 |  |  |  |  |
| DLA_LG9_006270 | LG9 |  |  |  |  |
| DLA_LG9_006280 | LG9 |  |  |  |  |
| DLA_LG9_006300 | LG9 |  |  |  |  |
| DLA_LG9_006310 | LG9 |  |  |  |  |
| DLA_LG9_006320 | LG9 |  |  |  |  |
| DLA_LG9_006330 | LG9 |  |  |  |  |
| DLA_LG9_006340 | LG9 |  |  |  |  |
| DLA_LG9_006350 | LG9 |  |  |  |  |
| DLA_LG9_006370 | LG9 |  |  |  |  |
| DLA_LG9_006380 | LG9 |  |  |  |  |
| DLA_LG9_006390 | LG9 |  |  |  |  |
| DLA_LG9_006400 | LG9 |  |  |  |  |
| DLA_LG9_006410 | LG9 |  |  |  |  |
| DLA_LG9_006415 | LG9 |  |  |  |  |
| DLA_LG9_006420 | LG9 |  |  |  |  |
| DLA_LG9_006430 | LG9 |  |  |  |  |
| DLA_LG9_006435 | LG9 |  |  |  |  |
| DLA_LG9_006450 | LG9 |  |  |  |  |
| DLA_LG9_006460 | LG9 |  |  |  |  |
| DLA_LG9_006470 | LG9 |  |  |  |  |
| DLA_LG9_006490 | LG9 |  |  |  |  |
| DLA_LG9_006500 | LG9 |  |  |  |  |
| DLA_LG9_006510 | LG9 |  |  |  |  |
| DLA_LG9_006520 | LG9 |  |  |  |  |
| DLA_LG9_006530 | LG9 |  |  |  |  |
| DLA_LG9_006540 | LG9 |  |  |  |  |
| DLA_LG9_006560 | LG9 |  |  |  |  |
| DLA_LG9_006580 | LG9 |  |  |  |  |
| DLA_LG9_006590 | LG9 |  |  |  |  |
| DLA_LG9_006605 | LG9 |  |  |  |  |
| DLA_LG9_006610 | LG9 |  |  |  |  |
| DLA_LG9_006620 | LG9 |  |  |  |  |
| DLA_LG9_006630 | LG9 |  |  |  |  |
| DLA_LG9_006635 | LG9 |  |  |  |  |
| DLA_LG9_006640 | LG9 |  |  |  |  |
| DLA_LG9_006645 | LG9 |  |  |  |  |
| DLA_LG9_006650 | LG9 |  |  |  |  |
| DLA_LG9_006660 | LG9 |  |  |  |  |
| DLA_LG9_006670 | LG9 |  |  |  |  |
| DLA_LG9_006680 | LG9 |  |  |  |  |
| DLA_LG9_006690 | LG9 |  |  |  |  |
| DLA_LG9_006700 | LG9 |  |  |  |  |
| DLA_LG9_006710 | LG9 |  |  |  |  |
| DLA_LG9_006715 | LG9 |  |  |  |  |
| DLA_LG9_006715_2 | LG9 |  |  |  |  |
| DLA_LG9_006720 | LG9 |  |  |  |  |
| DLA_LG9_006730 | LG9 |  |  |  |  |
| DLA_LG9_006740 | LG9 |  |  |  |  |
| DLA_LG9_006750 | LG9 |  |  |  |  |
| DLA_LG9_006770 | LG9 |  |  |  |  |
| DLA_LG9_006780 | LG9 |  |  |  |  |
| DLA_LG9_006790 | LG9 |  |  |  |  |
| DLA_LG9_006800 | LG9 |  |  |  |  |
| DLA_LG9_006810 | LG9 |  |  |  |  |
| DLA_LG9_006820 | LG9 |  |  |  |  |
| DLA_LG9_006830 | LG9 |  |  |  |  |
| DLA_LG9_006840 | LG9 |  |  |  |  |
| DLA_LG9_006850 | LG9 |  |  |  |  |
| DLA_LG9_006860 | LG9 |  |  |  |  |
| DLA_LG9_006865 | LG9 |  |  |  |  |
| DLA_LG9_006870 | LG9 |  |  |  |  |
| DLA_LG9_006880 | LG9 |  |  |  |  |
| DLA_LG9_006890 | LG9 |  |  |  |  |
| DLA_LG9_006900 | LG9 |  |  |  |  |
| DLA_LG9_006910 | LG9 |  |  |  |  |
| DLA_LG9_006920 | LG9 |  |  |  |  |
| DLA_LG9_006930 | LG9 |  |  |  |  |
| DLA_LGx_004240 | LGx |  |  |  |  |
| DLA_LGx_000260 | LGx |  |  |  |  |
| DLA_LGx_001280 | LGx |  |  |  |  |
| DLA_LGx_001580 | LGx |  |  |  |  |
| DLA_LGx_002320 | LGx |  |  |  |  |
| DLA_LGx_002600 | LGx |  |  |  |  |
| DLA_LGx_003510 | LGx |  |  |  |  |
| DLA_LGx_003700 | LGx |  |  |  |  |
| DLA_LGx_000030 | LGx |  |  |  |  |
| DLA_LGx_000220 | LGx |  |  |  |  |
| DLA_LGx_000230 | LGx |  |  |  |  |
| DLA_LGx_000350 | LGx |  |  |  |  |
| DLA_LGx_000370 | LGx |  |  |  |  |
| DLA_LGx_000380 | LGx |  |  |  |  |
| DLA_LGx_000420 | LGx |  |  |  |  |
| DLA_LGx_000470 | LGx |  |  |  |  |
| DLA_LGx_000500 | LGx |  |  |  |  |
| DLA_LGx_000650 | LGx |  |  |  |  |
| DLA_LGx_000750 | LGx |  |  |  |  |
| DLA_LGx_000860 | LGx |  |  |  |  |
| DLA_LGx_000880 | LGx |  |  |  |  |
| DLA_LGx_001030 | LGx |  |  |  |  |
| DLA_LGx_001090 | LGx |  |  |  |  |
| DLA_LGx_001095 | LGx |  |  |  |  |
| DLA_LGx_001120 | LGx |  |  |  |  |
| DLA_LGx_001350 | LGx |  |  |  |  |
| DLA_LGx_001360 | LGx |  |  |  |  |
| DLA_LGx_001500 | LGx |  |  |  |  |
| DLA_LGx_001570 | LGx |  |  |  |  |
| DLA_LGx_001640 | LGx |  |  |  |  |
| DLA_LGx_001670 | LGx |  |  |  |  |
| DLA_LGx_001720 | LGx |  |  |  |  |
| DLA_LGx_001750 | LGx |  |  |  |  |
| DLA_LGx_001760 | LGx |  |  |  |  |
| DLA_LGx_001830 | LGx |  |  |  |  |
| DLA_LGx_001900 | LGx |  |  |  |  |
| DLA_LGx_001990 | LGx |  |  |  |  |
| DLA_LGx_002130 | LGx |  |  |  |  |
| DLA_LGx_002280 | LGx |  |  |  |  |
| DLA_LGx_002370 | LGx |  |  |  |  |
| DLA_LGx_002400 | LGx |  |  |  |  |
| DLA_LGx_002440 | LGx |  |  |  |  |
| DLA_LGx_002510 | LGx |  |  |  |  |
| DLA_LGx_002580 | LGx |  |  |  |  |
| DLA_LGx_002610 | LGx |  |  |  |  |
| DLA_LGx_002620 | LGx |  |  |  |  |
| DLA_LGx_002690 | LGx |  |  |  |  |
| DLA_LGx_002720 | LGx |  |  |  |  |
| DLA_LGx_002760 | LGx |  |  |  |  |
| DLA_LGx_002890 | LGx |  |  |  |  |
| DLA_LGx_003050 | LGx |  |  |  |  |
| DLA_LGx_003080 | LGx |  |  |  |  |
| DLA_LGx_003230 | LGx |  |  |  |  |
| DLA_LGx_003420 | LGx |  |  |  |  |
| DLA_LGx_003470 | LGx |  |  |  |  |
| DLA_LGx_003600 | LGx |  |  |  |  |
| DLA_LGx_003610 | LGx |  |  |  |  |
| DLA_LGx_003670 | LGx |  |  |  |  |
| DLA_LGx_003870 | LGx |  |  |  |  |
| DLA_LGx_004070 | LGx |  |  |  |  |
| DLA_LGx_004160 | LGx |  |  |  |  |
| DLA_LGx_004290 | LGx |  |  |  |  |
| DLA_LGx_004390 | LGx |  |  |  |  |
| DLA_LGx_004450 | LGx |  |  |  |  |
| DLA_LGx_000010 | LGx |  |  |  |  |
| DLA_LGx_000020 | LGx |  |  |  |  |
| DLA_LGx_000035 | LGx |  |  |  |  |
| DLA_LGx_000040 | LGx |  |  |  |  |
| DLA_LGx_000050 | LGx |  |  |  |  |
| DLA_LGx_000070 | LGx |  |  |  |  |
| DLA_LGx_000080 | LGx |  |  |  |  |
| DLA_LGx_000090 | LGx |  |  |  |  |
| DLA_LGx_000100 | LGx |  |  |  |  |
| DLA_LGx_000105 | LGx |  |  |  |  |
| DLA_LGx_000110 | LGx |  |  |  |  |
| DLA_LGx_000120 | LGx |  |  |  |  |
| DLA_LGx_000130 | LGx |  |  |  |  |
| DLA_LGx_000140 | LGx |  |  |  |  |
| DLA_LGx_000150 | LGx |  |  |  |  |
| DLA_LGx_000160 | LGx |  |  |  |  |
| DLA_LGx_000170 | LGx |  |  |  |  |
| DLA_LGx_000180 | LGx |  |  |  |  |
| DLA_LGx_000190 | LGx |  |  |  |  |
| DLA_LGx_000200 | LGx |  |  |  |  |
| DLA_LGx_000210 | LGx |  |  |  |  |
| DLA_LGx_000240 | LGx |  |  |  |  |
| DLA_LGx_000250 | LGx |  |  |  |  |
| DLA_LGx_000280 | LGx |  |  |  |  |
| DLA_LGx_000285 | LGx |  |  |  |  |
| DLA_LGx_000290 | LGx |  |  |  |  |
| DLA_LGx_000300 | LGx |  |  |  |  |
| DLA_LGx_000310 | LGx |  |  |  |  |
| DLA_LGx_000315 | LGx |  |  |  |  |
| DLA_LGx_000320 | LGx |  |  |  |  |
| DLA_LGx_000330 | LGx |  |  |  |  |
| DLA_LGx_000340 | LGx |  |  |  |  |
| DLA_LGx_000360 | LGx |  |  |  |  |
| DLA_LGx_000390 | LGx |  |  |  |  |
| DLA_LGx_000400 | LGx |  |  |  |  |
| DLA_LGx_000415 | LGx |  |  |  |  |
| DLA_LGx_000430 | LGx |  |  |  |  |
| DLA_LGx_000440 | LGx |  |  |  |  |
| DLA_LGx_000450 | LGx |  |  |  |  |
| DLA_LGx_000460 | LGx |  |  |  |  |
| DLA_LGx_000475 | LGx |  |  |  |  |
| DLA_LGx_000480 | LGx |  |  |  |  |
| DLA_LGx_000490 | LGx |  |  |  |  |
| DLA_LGx_000510 | LGx |  |  |  |  |
| DLA_LGx_000515 | LGx |  |  |  |  |
| DLA_LGx_000520 | LGx |  |  |  |  |
| DLA_LGx_000530 | LGx |  |  |  |  |
| DLA_LGx_000540 | LGx |  |  |  |  |
| DLA_LGx_000550 | LGx |  |  |  |  |
| DLA_LGx_000560 | LGx |  |  |  |  |
| DLA_LGx_000570 | LGx |  |  |  |  |
| DLA_LGx_000580 | LGx |  |  |  |  |
| DLA_LGx_000590 | LGx |  |  |  |  |
| DLA_LGx_000600 | LGx |  |  |  |  |
| DLA_LGx_000610 | LGx |  |  |  |  |
| DLA_LGx_000620 | LGx |  |  |  |  |
| DLA_LGx_000625 | LGx |  |  |  |  |
| DLA_LGx_000625_2 | LGx |  |  |  |  |
| DLA_LGx_000630 | LGx |  |  |  |  |
| DLA_LGx_000640 | LGx |  |  |  |  |
| DLA_LGx_000660 | LGx |  |  |  |  |
| DLA_LGx_000670 | LGx |  |  |  |  |
| DLA_LGx_000680 | LGx |  |  |  |  |
| DLA_LGx_000690 | LGx |  |  |  |  |
| DLA_LGx_000700 | LGx |  |  |  |  |
| DLA_LGx_000710 | LGx |  |  |  |  |
| DLA_LGx_000720 | LGx |  |  |  |  |
| DLA_LGx_000760 | LGx |  |  |  |  |
| DLA_LGx_000770 | LGx |  |  |  |  |
| DLA_LGx_000780 | LGx |  |  |  |  |
| DLA_LGx_000790 | LGx |  |  |  |  |
| DLA_LGx_000800 | LGx |  |  |  |  |
| DLA_LGx_000810 | LGx |  |  |  |  |
| DLA_LGx_000815 | LGx |  |  |  |  |
| DLA_LGx_000830 | LGx |  |  |  |  |
| DLA_LGx_000840 | LGx |  |  |  |  |
| DLA_LGx_000850 | LGx |  |  |  |  |
| DLA_LGx_000870 | LGx |  |  |  |  |
| DLA_LGx_000875 | LGx |  |  |  |  |
| DLA_LGx_000890 | LGx |  |  |  |  |
| DLA_LGx_000900 | LGx |  |  |  |  |
| DLA_LGx_000910 | LGx |  |  |  |  |
| DLA_LGx_000920 | LGx |  |  |  |  |
| DLA_LGx_000930 | LGx |  |  |  |  |
| DLA_LGx_000935 | LGx |  |  |  |  |
| DLA_LGx_000940 | LGx |  |  |  |  |
| DLA_LGx_000950 | LGx |  |  |  |  |
| DLA_LGx_000960 | LGx |  |  |  |  |
| DLA_LGx_000970 | LGx |  |  |  |  |
| DLA_LGx_000980 | LGx |  |  |  |  |
| DLA_LGx_001000 | LGx |  |  |  |  |
| DLA_LGx_001010 | LGx |  |  |  |  |
| DLA_LGx_001020 | LGx |  |  |  |  |
| DLA_LGx_001040 | LGx |  |  |  |  |
| DLA_LGx_001050 | LGx |  |  |  |  |
| DLA_LGx_001060 | LGx |  |  |  |  |
| DLA_LGx_001070 | LGx |  |  |  |  |
| DLA_LGx_001080 | LGx |  |  |  |  |
| DLA_LGx_001100 | LGx |  |  |  |  |
| DLA_LGx_001110 | LGx |  |  |  |  |
| DLA_LGx_001115 | LGx |  |  |  |  |
| DLA_LGx_001130 | LGx |  |  |  |  |
| DLA_LGx_001140 | LGx |  |  |  |  |
| DLA_LGx_001150 | LGx |  |  |  |  |
| DLA_LGx_001170 | LGx |  |  |  |  |
| DLA_LGx_001180 | LGx |  |  |  |  |
| DLA_LGx_001190 | LGx |  |  |  |  |
| DLA_LGx_001200 | LGx |  |  |  |  |
| DLA_LGx_001210 | LGx |  |  |  |  |
| DLA_LGx_001220 | LGx |  |  |  |  |
| DLA_LGx_001230 | LGx |  |  |  |  |
| DLA_LGx_001240 | LGx |  |  |  |  |
| DLA_LGx_001250 | LGx |  |  |  |  |
| DLA_LGx_001260 | LGx |  |  |  |  |
| DLA_LGx_001270 | LGx |  |  |  |  |
| DLA_LGx_001285 | LGx |  |  |  |  |
| DLA_LGx_001290 | LGx |  |  |  |  |
| DLA_LGx_001300 | LGx |  |  |  |  |
| DLA_LGx_001310 | LGx |  |  |  |  |
| DLA_LGx_001320 | LGx |  |  |  |  |
| DLA_LGx_001330 | LGx |  |  |  |  |
| DLA_LGx_001340 | LGx |  |  |  |  |
| DLA_LGx_001370 | LGx |  |  |  |  |
| DLA_LGx_001380 | LGx |  |  |  |  |
| DLA_LGx_001390 | LGx |  |  |  |  |
| DLA_LGx_001400 | LGx |  |  |  |  |
| DLA_LGx_001410 | LGx |  |  |  |  |
| DLA_LGx_001420 | LGx |  |  |  |  |
| DLA_LGx_001430 | LGx |  |  |  |  |
| DLA_LGx_001440 | LGx |  |  |  |  |
| DLA_LGx_001450 | LGx |  |  |  |  |
| DLA_LGx_001460 | LGx |  |  |  |  |
| DLA_LGx_001470 | LGx |  |  |  |  |
| DLA_LGx_001475 | LGx |  |  |  |  |
| DLA_LGx_001480 | LGx |  |  |  |  |
| DLA_LGx_001490 | LGx |  |  |  |  |
| DLA_LGx_001510 | LGx |  |  |  |  |
| DLA_LGx_001520 | LGx |  |  |  |  |
| DLA_LGx_001530 | LGx |  |  |  |  |
| DLA_LGx_001540 | LGx |  |  |  |  |
| DLA_LGx_001550 | LGx |  |  |  |  |
| DLA_LGx_001560 | LGx |  |  |  |  |
| DLA_LGx_001590 | LGx |  |  |  |  |
| DLA_LGx_001600 | LGx |  |  |  |  |
| DLA_LGx_001610 | LGx |  |  |  |  |
| DLA_LGx_001620 | LGx |  |  |  |  |
| DLA_LGx_001630 | LGx |  |  |  |  |
| DLA_LGx_001650 | LGx |  |  |  |  |
| DLA_LGx_001660 | LGx |  |  |  |  |
| DLA_LGx_001680 | LGx |  |  |  |  |
| DLA_LGx_001690 | LGx |  |  |  |  |
| DLA_LGx_001700 | LGx |  |  |  |  |
| DLA_LGx_001710 | LGx |  |  |  |  |
| DLA_LGx_001730 | LGx |  |  |  |  |
| DLA_LGx_001740 | LGx |  |  |  |  |
| DLA_LGx_001770 | LGx |  |  |  |  |
| DLA_LGx_001790 | LGx |  |  |  |  |
| DLA_LGx_001800 | LGx |  |  |  |  |
| DLA_LGx_001820 | LGx |  |  |  |  |
| DLA_LGx_001840 | LGx |  |  |  |  |
| DLA_LGx_001850 | LGx |  |  |  |  |
| DLA_LGx_001860 | LGx |  |  |  |  |
| DLA_LGx_001870 | LGx |  |  |  |  |
| DLA_LGx_001880 | LGx |  |  |  |  |
| DLA_LGx_001890 | LGx |  |  |  |  |
| DLA_LGx_001910 | LGx |  |  |  |  |
| DLA_LGx_001920 | LGx |  |  |  |  |
| DLA_LGx_001930 | LGx |  |  |  |  |
| DLA_LGx_001940 | LGx |  |  |  |  |
| DLA_LGx_001950 | LGx |  |  |  |  |
| DLA_LGx_001960 | LGx |  |  |  |  |
| DLA_LGx_001965 | LGx |  |  |  |  |
| DLA_LGx_001965_2 | LGx |  |  |  |  |
| DLA_LGx_001970 | LGx |  |  |  |  |
| DLA_LGx_001980 | LGx |  |  |  |  |
| DLA_LGx_002000 | LGx |  |  |  |  |
| DLA_LGx_002005 | LGx |  |  |  |  |
| DLA_LGx_002010 | LGx |  |  |  |  |
| DLA_LGx_002015 | LGx |  |  |  |  |
| DLA_LGx_002020 | LGx |  |  |  |  |
| DLA_LGx_002050 | LGx |  |  |  |  |
| DLA_LGx_002060 | LGx |  |  |  |  |
| DLA_LGx_002080 | LGx |  |  |  |  |
| DLA_LGx_002090 | LGx |  |  |  |  |
| DLA_LGx_002100 | LGx |  |  |  |  |
| DLA_LGx_002135 | LGx |  |  |  |  |
| DLA_LGx_002140 | LGx |  |  |  |  |
| DLA_LGx_002150 | LGx |  |  |  |  |
| DLA_LGx_002160 | LGx |  |  |  |  |
| DLA_LGx_002170 | LGx |  |  |  |  |
| DLA_LGx_002180 | LGx |  |  |  |  |
| DLA_LGx_002190 | LGx |  |  |  |  |
| DLA_LGx_002200 | LGx |  |  |  |  |
| DLA_LGx_002210 | LGx |  |  |  |  |
| DLA_LGx_002220 | LGx |  |  |  |  |
| DLA_LGx_002230 | LGx |  |  |  |  |
| DLA_LGx_002240 | LGx |  |  |  |  |
| DLA_LGx_002250 | LGx |  |  |  |  |
| DLA_LGx_002260 | LGx |  |  |  |  |
| DLA_LGx_002270 | LGx |  |  |  |  |
| DLA_LGx_002290 | LGx |  |  |  |  |
| DLA_LGx_002300 | LGx |  |  |  |  |
| DLA_LGx_002310 | LGx |  |  |  |  |
| DLA_LGx_002315 | LGx |  |  |  |  |
| DLA_LGx_002330 | LGx |  |  |  |  |
| DLA_LGx_002340 | LGx |  |  |  |  |
| DLA_LGx_002350 | LGx |  |  |  |  |
| DLA_LGx_002360 | LGx |  |  |  |  |
| DLA_LGx_002380 | LGx |  |  |  |  |
| DLA_LGx_002390 | LGx |  |  |  |  |
| DLA_LGx_002420 | LGx |  |  |  |  |
| DLA_LGx_002430 | LGx |  |  |  |  |
| DLA_LGx_002450 | LGx |  |  |  |  |
| DLA_LGx_002460 | LGx |  |  |  |  |
| DLA_LGx_002470 | LGx |  |  |  |  |
| DLA_LGx_002480 | LGx |  |  |  |  |
| DLA_LGx_002490 | LGx |  |  |  |  |
| DLA_LGx_002500 | LGx |  |  |  |  |
| DLA_LGx_002520 | LGx |  |  |  |  |
| DLA_LGx_002530 | LGx |  |  |  |  |
| DLA_LGx_002540 | LGx |  |  |  |  |
| DLA_LGx_002550 | LGx |  |  |  |  |
| DLA_LGx_002560 | LGx |  |  |  |  |
| DLA_LGx_002570 | LGx |  |  |  |  |
| DLA_LGx_002590 | LGx |  |  |  |  |
| DLA_LGx_002630 | LGx |  |  |  |  |
| DLA_LGx_002640 | LGx |  |  |  |  |
| DLA_LGx_002645 | LGx |  |  |  |  |
| DLA_LGx_002645_2 | LGx |  |  |  |  |
| DLA_LGx_002670 | LGx |  |  |  |  |
| DLA_LGx_002680 | LGx |  |  |  |  |
| DLA_LGx_002700 | LGx |  |  |  |  |
| DLA_LGx_002710 | LGx |  |  |  |  |
| DLA_LGx_002740 | LGx |  |  |  |  |
| DLA_LGx_002750 | LGx |  |  |  |  |
| DLA_LGx_002755 | LGx |  |  |  |  |
| DLA_LGx_002770 | LGx |  |  |  |  |
| DLA_LGx_002780 | LGx |  |  |  |  |
| DLA_LGx_002790 | LGx |  |  |  |  |
| DLA_LGx_002800 | LGx |  |  |  |  |
| DLA_LGx_002810 | LGx |  |  |  |  |
| DLA_LGx_002820 | LGx |  |  |  |  |
| DLA_LGx_002830 | LGx |  |  |  |  |
| DLA_LGx_002840 | LGx |  |  |  |  |
| DLA_LGx_002850 | LGx |  |  |  |  |
| DLA_LGx_002860 | LGx |  |  |  |  |
| DLA_LGx_002870 | LGx |  |  |  |  |
| DLA_LGx_002880 | LGx |  |  |  |  |
| DLA_LGx_002905 | LGx |  |  |  |  |
| DLA_LGx_002910 | LGx |  |  |  |  |
| DLA_LGx_002920 | LGx |  |  |  |  |
| DLA_LGx_002930 | LGx |  |  |  |  |
| DLA_LGx_002940 | LGx |  |  |  |  |
| DLA_LGx_002950 | LGx |  |  |  |  |
| DLA_LGx_002955 | LGx |  |  |  |  |
| DLA_LGx_002960 | LGx |  |  |  |  |
| DLA_LGx_002970 | LGx |  |  |  |  |
| DLA_LGx_002980 | LGx |  |  |  |  |
| DLA_LGx_002990 | LGx |  |  |  |  |
| DLA_LGx_002995 | LGx |  |  |  |  |
| DLA_LGx_003000 | LGx |  |  |  |  |
| DLA_LGx_003010 | LGx |  |  |  |  |
| DLA_LGx_003020 | LGx |  |  |  |  |
| DLA_LGx_003030 | LGx |  |  |  |  |
| DLA_LGx_003040 | LGx |  |  |  |  |
| DLA_LGx_003060 | LGx |  |  |  |  |
| DLA_LGx_003070 | LGx |  |  |  |  |
| DLA_LGx_003100 | LGx |  |  |  |  |
| DLA_LGx_003110 | LGx |  |  |  |  |
| DLA_LGx_003120 | LGx |  |  |  |  |
| DLA_LGx_003130 | LGx |  |  |  |  |
| DLA_LGx_003140 | LGx |  |  |  |  |
| DLA_LGx_003170 | LGx |  |  |  |  |
| DLA_LGx_003180 | LGx |  |  |  |  |
| DLA_LGx_003190 | LGx |  |  |  |  |
| DLA_LGx_003200 | LGx |  |  |  |  |
| DLA_LGx_003210 | LGx |  |  |  |  |
| DLA_LGx_003250 | LGx |  |  |  |  |
| DLA_LGx_003260 | LGx |  |  |  |  |
| DLA_LGx_003270 | LGx |  |  |  |  |
| DLA_LGx_003280 | LGx |  |  |  |  |
| DLA_LGx_003290 | LGx |  |  |  |  |
| DLA_LGx_003295 | LGx |  |  |  |  |
| DLA_LGx_003300 | LGx |  |  |  |  |
| DLA_LGx_003310 | LGx |  |  |  |  |
| DLA_LGx_003320 | LGx |  |  |  |  |
| DLA_LGx_003330 | LGx |  |  |  |  |
| DLA_LGx_003340 | LGx |  |  |  |  |
| DLA_LGx_003350 | LGx |  |  |  |  |
| DLA_LGx_003360 | LGx |  |  |  |  |
| DLA_LGx_003370 | LGx |  |  |  |  |
| DLA_LGx_003380 | LGx |  |  |  |  |
| DLA_LGx_003385 | LGx |  |  |  |  |
| DLA_LGx_003390 | LGx |  |  |  |  |
| DLA_LGx_003400 | LGx |  |  |  |  |
| DLA_LGx_003410 | LGx |  |  |  |  |
| DLA_LGx_003430 | LGx |  |  |  |  |
| DLA_LGx_003440 | LGx |  |  |  |  |
| DLA_LGx_003450 | LGx |  |  |  |  |
| DLA_LGx_003460 | LGx |  |  |  |  |
| DLA_LGx_003480 | LGx |  |  |  |  |
| DLA_LGx_003490 | LGx |  |  |  |  |
| DLA_LGx_003500 | LGx |  |  |  |  |
| DLA_LGx_003520 | LGx |  |  |  |  |
| DLA_LGx_003530 | LGx |  |  |  |  |
| DLA_LGx_003550 | LGx |  |  |  |  |
| DLA_LGx_003560 | LGx |  |  |  |  |
| DLA_LGx_003570 | LGx |  |  |  |  |
| DLA_LGx_003580 | LGx |  |  |  |  |
| DLA_LGx_003585 | LGx |  |  |  |  |
| DLA_LGx_003590 | LGx |  |  |  |  |
| DLA_LGx_003620 | LGx |  |  |  |  |
| DLA_LGx_003630 | LGx |  |  |  |  |
| DLA_LGx_003640 | LGx |  |  |  |  |
| DLA_LGx_003650 | LGx |  |  |  |  |
| DLA_LGx_003655 | LGx |  |  |  |  |
| DLA_LGx_003660 | LGx |  |  |  |  |
| DLA_LGx_003680 | LGx |  |  |  |  |
| DLA_LGx_003690 | LGx |  |  |  |  |
| DLA_LGx_003720 | LGx |  |  |  |  |
| DLA_LGx_003740 | LGx |  |  |  |  |
| DLA_LGx_003750 | LGx |  |  |  |  |
| DLA_LGx_003760 | LGx |  |  |  |  |
| DLA_LGx_003770 | LGx |  |  |  |  |
| DLA_LGx_003780 | LGx |  |  |  |  |
| DLA_LGx_003800 | LGx |  |  |  |  |
| DLA_LGx_003810 | LGx |  |  |  |  |
| DLA_LGx_003820 | LGx |  |  |  |  |
| DLA_LGx_003830 | LGx |  |  |  |  |
| DLA_LGx_003840 | LGx |  |  |  |  |
| DLA_LGx_003860 | LGx |  |  |  |  |
| DLA_LGx_003880 | LGx |  |  |  |  |
| DLA_LGx_003900 | LGx |  |  |  |  |
| DLA_LGx_003910 | LGx |  |  |  |  |
| DLA_LGx_003920 | LGx |  |  |  |  |
| DLA_LGx_003930 | LGx |  |  |  |  |
| DLA_LGx_003940 | LGx |  |  |  |  |
| DLA_LGx_003950 | LGx |  |  |  |  |
| DLA_LGx_003960 | LGx |  |  |  |  |
| DLA_LGx_003970 | LGx |  |  |  |  |
| DLA_LGx_003980 | LGx |  |  |  |  |
| DLA_LGx_003990 | LGx |  |  |  |  |
| DLA_LGx_004000 | LGx |  |  |  |  |
| DLA_LGx_004010 | LGx |  |  |  |  |
| DLA_LGx_004020 | LGx |  |  |  |  |
| DLA_LGx_004030 | LGx |  |  |  |  |
| DLA_LGx_004040 | LGx |  |  |  |  |
| DLA_LGx_004045 | LGx |  |  |  |  |
| DLA_LGx_004050 | LGx |  |  |  |  |
| DLA_LGx_004060 | LGx |  |  |  |  |
| DLA_LGx_004080 | LGx |  |  |  |  |
| DLA_LGx_004090 | LGx |  |  |  |  |
| DLA_LGx_004100 | LGx |  |  |  |  |
| DLA_LGx_004110 | LGx |  |  |  |  |
| DLA_LGx_004120 | LGx |  |  |  |  |
| DLA_LGx_004130 | LGx |  |  |  |  |
| DLA_LGx_004140 | LGx |  |  |  |  |
| DLA_LGx_004150 | LGx |  |  |  |  |
| DLA_LGx_004170 | LGx |  |  |  |  |
| DLA_LGx_004180 | LGx |  |  |  |  |
| DLA_LGx_004190 | LGx |  |  |  |  |
| DLA_LGx_004200 | LGx |  |  |  |  |
| DLA_LGx_004210 | LGx |  |  |  |  |
| DLA_LGx_004220 | LGx |  |  |  |  |
| DLA_LGx_004230 | LGx |  |  |  |  |
| DLA_LGx_004250 | LGx |  |  |  |  |
| DLA_LGx_004260 | LGx |  |  |  |  |
| DLA_LGx_004270 | LGx |  |  |  |  |
| DLA_LGx_004280 | LGx |  |  |  |  |
| DLA_LGx_004295 | LGx |  |  |  |  |
| DLA_LGx_004300 | LGx |  |  |  |  |
| DLA_LGx_004310 | LGx |  |  |  |  |
| DLA_LGx_004320 | LGx |  |  |  |  |
| DLA_LGx_004325 | LGx |  |  |  |  |
| DLA_LGx_004330 | LGx |  |  |  |  |
| DLA_LGx_004335 | LGx |  |  |  |  |
| DLA_LGx_004340 | LGx |  |  |  |  |
| DLA_LGx_004350 | LGx |  |  |  |  |
| DLA_LGx_004355 | LGx |  |  |  |  |
| DLA_LGx_004360 | LGx |  |  |  |  |
| DLA_LGx_004370 | LGx |  |  |  |  |
| DLA_LGx_004380 | LGx |  |  |  |  |
| DLA_LGx_004400 | LGx |  |  |  |  |
| DLA_LGx_004410 | LGx |  |  |  |  |
| DLA_LGx_004420 | LGx |  |  |  |  |
| DLA_LGx_004430 | LGx |  |  |  |  |
| DLA_LGx_004440 | LGx |  |  |  |  |
| DLA_LGx_004460 | LGx |  |  |  |  |
| DLA_LGx_004470 | LGx |  |  |  |  |
| DLA_LGx_004480 | LGx |  |  |  |  |
| DLA_LGx_004490 | LGx |  |  |  |  |
| DLA_LGx_004500 | LGx |  |  |  |  |
| DLA_LGx_004510 | LGx |  |  |  |  |
| DLA_LGx_004520 | LGx |  |  |  |  |
| DLA_LGx_004530 | LGx |  |  |  |  |
| DLA_LGx_004540 | LGx |  |  |  |  |
| DLA_LG10_000660 | LG10 |  |  |  |  |
| DLA_LG10_001010 | LG10 |  |  |  |  |
| DLA_LG10_002070 | LG10 |  |  |  |  |
| DLA_LG10_004060 | LG10 |  |  |  |  |
| DLA_LG10_005440 | LG10 |  |  |  |  |
| DLA_LG10_007470 | LG10 |  |  |  |  |
| DLA_LG10_007760 | LG10 |  |  |  |  |
| DLA_LG10_000130 | LG10 |  |  |  |  |
| DLA_LG10_000300 | LG10 |  |  |  |  |
| DLA_LG10_000310 | LG10 |  |  |  |  |
| DLA_LG10_000360 | LG10 |  |  |  |  |
| DLA_LG10_000460 | LG10 |  |  |  |  |
| DLA_LG10_000510 | LG10 |  |  |  |  |
| DLA_LG10_000580 | LG10 |  |  |  |  |
| DLA_LG10_000690 | LG10 |  |  |  |  |
| DLA_LG10_000755 | LG10 |  |  |  |  |
| DLA_LG10_000850 | LG10 |  |  |  |  |
| DLA_LG10_000860 | LG10 |  |  |  |  |
| DLA_LG10_000970 | LG10 |  |  |  |  |
| DLA_LG10_001070 | LG10 |  |  |  |  |
| DLA_LG10_001140 | LG10 |  |  |  |  |
| DLA_LG10_001200 | LG10 |  |  |  |  |
| DLA_LG10_001555 | LG10 |  |  |  |  |
| DLA_LG10_001950 | LG10 |  |  |  |  |
| DLA_LG10_002040 | LG10 |  |  |  |  |
| DLA_LG10_002080 | LG10 |  |  |  |  |
| DLA_LG10_002160 | LG10 |  |  |  |  |
| DLA_LG10_002190 | LG10 |  |  |  |  |
| DLA_LG10_002380 | LG10 |  |  |  |  |
| DLA_LG10_002460 | LG10 |  |  |  |  |
| DLA_LG10_002760 | LG10 |  |  |  |  |
| DLA_LG10_002830 | LG10 |  |  |  |  |
| DLA_LG10_002925_2 | LG10 |  |  |  |  |
| DLA_LG10_003200 | LG10 |  |  |  |  |
| DLA_LG10_003310 | LG10 |  |  |  |  |
| DLA_LG10_003550 | LG10 |  |  |  |  |
| DLA_LG10_003720 | LG10 |  |  |  |  |
| DLA_LG10_004070 | LG10 |  |  |  |  |
| DLA_LG10_004090 | LG10 |  |  |  |  |
| DLA_LG10_004140 | LG10 |  |  |  |  |
| DLA_LG10_004240 | LG10 |  |  |  |  |
| DLA_LG10_004340 | LG10 |  |  |  |  |
| DLA_LG10_004880 | LG10 |  |  |  |  |
| DLA_LG10_004980 | LG10 |  |  |  |  |
| DLA_LG10_005020 | LG10 |  |  |  |  |
| DLA_LG10_005070 | LG10 |  |  |  |  |
| DLA_LG10_005280 | LG10 |  |  |  |  |
| DLA_LG10_005330 | LG10 |  |  |  |  |
| DLA_LG10_005540 | LG10 |  |  |  |  |
| DLA_LG10_005575 | LG10 |  |  |  |  |
| DLA_LG10_005770 | LG10 |  |  |  |  |
| DLA_LG10_005820 | LG10 |  |  |  |  |
| DLA_LG10_006010 | LG10 |  |  |  |  |
| DLA_LG10_006095 | LG10 |  |  |  |  |
| DLA_LG10_006210 | LG10 |  |  |  |  |
| DLA_LG10_006260 | LG10 |  |  |  |  |
| DLA_LG10_006740 | LG10 |  |  |  |  |
| DLA_LG10_006840 | LG10 |  |  |  |  |
| DLA_LG10_006880 | LG10 |  |  |  |  |
| DLA_LG10_006930 | LG10 |  |  |  |  |
| DLA_LG10_007290 | LG10 |  |  |  |  |
| DLA_LG10_007340 | LG10 |  |  |  |  |
| DLA_LG10_007420 | LG10 |  |  |  |  |
| DLA_LG10_007560 | LG10 |  |  |  |  |
| DLA_LG10_007880 | LG10 |  |  |  |  |
| DLA_LG10_007940 | LG10 |  |  |  |  |
| DLA_LG10_008130 | LG10 |  |  |  |  |
| DLA_LG10_008390 | LG10 |  |  |  |  |
| DLA_LG10_008580 | LG10 |  |  |  |  |
| DLA_LG10_008670 | LG10 |  |  |  |  |
| DLA_LG10_008710 | LG10 |  |  |  |  |
| DLA_LG10_008720 | LG10 |  |  |  |  |
| DLA_LG10_008940 | LG10 |  |  |  |  |
| DLA_LG10_008980 | LG10 |  |  |  |  |
| DLA_LG10_009090 | LG10 |  |  |  |  |
| DLA_LG10_000005 | LG10 |  |  |  |  |
| DLA_LG10_000010 | LG10 |  |  |  |  |
| DLA_LG10_000020 | LG10 |  |  |  |  |
| DLA_LG10_000030 | LG10 |  |  |  |  |
| DLA_LG10_000050 | LG10 |  |  |  |  |
| DLA_LG10_000060 | LG10 |  |  |  |  |
| DLA_LG10_000070 | LG10 |  |  |  |  |
| DLA_LG10_000080 | LG10 |  |  |  |  |
| DLA_LG10_000090 | LG10 |  |  |  |  |
| DLA_LG10_000100 | LG10 |  |  |  |  |
| DLA_LG10_000110 | LG10 |  |  |  |  |
| DLA_LG10_000120 | LG10 |  |  |  |  |
| DLA_LG10_000140 | LG10 |  |  |  |  |
| DLA_LG10_000150 | LG10 |  |  |  |  |
| DLA_LG10_000160 | LG10 |  |  |  |  |
| DLA_LG10_000170 | LG10 |  |  |  |  |
| DLA_LG10_000190 | LG10 |  |  |  |  |
| DLA_LG10_000200 | LG10 |  |  |  |  |
| DLA_LG10_000210 | LG10 |  |  |  |  |
| DLA_LG10_000220 | LG10 |  |  |  |  |
| DLA_LG10_000230 | LG10 |  |  |  |  |
| DLA_LG10_000240 | LG10 |  |  |  |  |
| DLA_LG10_000250 | LG10 |  |  |  |  |
| DLA_LG10_000255 | LG10 |  |  |  |  |
| DLA_LG10_000260 | LG10 |  |  |  |  |
| DLA_LG10_000270 | LG10 |  |  |  |  |
| DLA_LG10_000280 | LG10 |  |  |  |  |
| DLA_LG10_000290 | LG10 |  |  |  |  |
| DLA_LG10_000320 | LG10 |  |  |  |  |
| DLA_LG10_000330 | LG10 |  |  |  |  |
| DLA_LG10_000340 | LG10 |  |  |  |  |
| DLA_LG10_000350 | LG10 |  |  |  |  |
| DLA_LG10_000370 | LG10 |  |  |  |  |
| DLA_LG10_000380 | LG10 |  |  |  |  |
| DLA_LG10_000390 | LG10 |  |  |  |  |
| DLA_LG10_000400 | LG10 |  |  |  |  |
| DLA_LG10_000410 | LG10 |  |  |  |  |
| DLA_LG10_000420 | LG10 |  |  |  |  |
| DLA_LG10_000430 | LG10 |  |  |  |  |
| DLA_LG10_000440 | LG10 |  |  |  |  |
| DLA_LG10_000470 | LG10 |  |  |  |  |
| DLA_LG10_000480 | LG10 |  |  |  |  |
| DLA_LG10_000490 | LG10 |  |  |  |  |
| DLA_LG10_000500 | LG10 |  |  |  |  |
| DLA_LG10_000520 | LG10 |  |  |  |  |
| DLA_LG10_000530 | LG10 |  |  |  |  |
| DLA_LG10_000550 | LG10 |  |  |  |  |
| DLA_LG10_000560 | LG10 |  |  |  |  |
| DLA_LG10_000570 | LG10 |  |  |  |  |
| DLA_LG10_000590 | LG10 |  |  |  |  |
| DLA_LG10_000600 | LG10 |  |  |  |  |
| DLA_LG10_000610 | LG10 |  |  |  |  |
| DLA_LG10_000620 | LG10 |  |  |  |  |
| DLA_LG10_000630 | LG10 |  |  |  |  |
| DLA_LG10_000640 | LG10 |  |  |  |  |
| DLA_LG10_000650 | LG10 |  |  |  |  |
| DLA_LG10_000670 | LG10 |  |  |  |  |
| DLA_LG10_000680 | LG10 |  |  |  |  |
| DLA_LG10_000700 | LG10 |  |  |  |  |
| DLA_LG10_000710 | LG10 |  |  |  |  |
| DLA_LG10_000720 | LG10 |  |  |  |  |
| DLA_LG10_000730 | LG10 |  |  |  |  |
| DLA_LG10_000740 | LG10 |  |  |  |  |
| DLA_LG10_000750 | LG10 |  |  |  |  |
| DLA_LG10_000760 | LG10 |  |  |  |  |
| DLA_LG10_000770 | LG10 |  |  |  |  |
| DLA_LG10_000780 | LG10 |  |  |  |  |
| DLA_LG10_000790 | LG10 |  |  |  |  |
| DLA_LG10_000795 | LG10 |  |  |  |  |
| DLA_LG10_000800 | LG10 |  |  |  |  |
| DLA_LG10_000810 | LG10 |  |  |  |  |
| DLA_LG10_000840 | LG10 |  |  |  |  |
| DLA_LG10_000870 | LG10 |  |  |  |  |
| DLA_LG10_000880 | LG10 |  |  |  |  |
| DLA_LG10_000890 | LG10 |  |  |  |  |
| DLA_LG10_000900 | LG10 |  |  |  |  |
| DLA_LG10_000910 | LG10 |  |  |  |  |
| DLA_LG10_000920 | LG10 |  |  |  |  |
| DLA_LG10_000930 | LG10 |  |  |  |  |
| DLA_LG10_000940 | LG10 |  |  |  |  |
| DLA_LG10_000950 | LG10 |  |  |  |  |
| DLA_LG10_000960 | LG10 |  |  |  |  |
| DLA_LG10_000980 | LG10 |  |  |  |  |
| DLA_LG10_000990 | LG10 |  |  |  |  |
| DLA_LG10_001000 | LG10 |  |  |  |  |
| DLA_LG10_001015 | LG10 |  |  |  |  |
| DLA_LG10_001020 | LG10 |  |  |  |  |
| DLA_LG10_001030 | LG10 |  |  |  |  |
| DLA_LG10_001040 | LG10 |  |  |  |  |
| DLA_LG10_001050 | LG10 |  |  |  |  |
| DLA_LG10_001060 | LG10 |  |  |  |  |
| DLA_LG10_001075 | LG10 |  |  |  |  |
| DLA_LG10_001080 | LG10 |  |  |  |  |
| DLA_LG10_001090 | LG10 |  |  |  |  |
| DLA_LG10_001100 | LG10 |  |  |  |  |
| DLA_LG10_001110 | LG10 |  |  |  |  |
| DLA_LG10_001130 | LG10 |  |  |  |  |
| DLA_LG10_001150 | LG10 |  |  |  |  |
| DLA_LG10_001160 | LG10 |  |  |  |  |
| DLA_LG10_001170 | LG10 |  |  |  |  |
| DLA_LG10_001180 | LG10 |  |  |  |  |
| DLA_LG10_001190 | LG10 |  |  |  |  |
| DLA_LG10_001210 | LG10 |  |  |  |  |
| DLA_LG10_001215 | LG10 |  |  |  |  |
| DLA_LG10_001220 | LG10 |  |  |  |  |
| DLA_LG10_001225 | LG10 |  |  |  |  |
| DLA_LG10_001230 | LG10 |  |  |  |  |
| DLA_LG10_001240 | LG10 |  |  |  |  |
| DLA_LG10_001250 | LG10 |  |  |  |  |
| DLA_LG10_001260 | LG10 |  |  |  |  |
| DLA_LG10_001270 | LG10 |  |  |  |  |
| DLA_LG10_001280 | LG10 |  |  |  |  |
| DLA_LG10_001300 | LG10 |  |  |  |  |
| DLA_LG10_001310 | LG10 |  |  |  |  |
| DLA_LG10_001320 | LG10 |  |  |  |  |
| DLA_LG10_001330 | LG10 |  |  |  |  |
| DLA_LG10_001340 | LG10 |  |  |  |  |
| DLA_LG10_001360 | LG10 |  |  |  |  |
| DLA_LG10_001390 | LG10 |  |  |  |  |
| DLA_LG10_001400 | LG10 |  |  |  |  |
| DLA_LG10_001410 | LG10 |  |  |  |  |
| DLA_LG10_001420 | LG10 |  |  |  |  |
| DLA_LG10_001430 | LG10 |  |  |  |  |
| DLA_LG10_001440 | LG10 |  |  |  |  |
| DLA_LG10_001450 | LG10 |  |  |  |  |
| DLA_LG10_001460 | LG10 |  |  |  |  |
| DLA_LG10_001470 | LG10 |  |  |  |  |
| DLA_LG10_001480 | LG10 |  |  |  |  |
| DLA_LG10_001490 | LG10 |  |  |  |  |
| DLA_LG10_001500 | LG10 |  |  |  |  |
| DLA_LG10_001520 | LG10 |  |  |  |  |
| DLA_LG10_001530 | LG10 |  |  |  |  |
| DLA_LG10_001540 | LG10 |  |  |  |  |
| DLA_LG10_001550 | LG10 |  |  |  |  |
| DLA_LG10_001555_2 | LG10 |  |  |  |  |
| DLA_LG10_001555_3 | LG10 |  |  |  |  |
| DLA_LG10_001570 | LG10 |  |  |  |  |
| DLA_LG10_001590 | LG10 |  |  |  |  |
| DLA_LG10_001600 | LG10 |  |  |  |  |
| DLA_LG10_001610 | LG10 |  |  |  |  |
| DLA_LG10_001620 | LG10 |  |  |  |  |
| DLA_LG10_001640 | LG10 |  |  |  |  |
| DLA_LG10_001650 | LG10 |  |  |  |  |
| DLA_LG10_001660 | LG10 |  |  |  |  |
| DLA_LG10_001670 | LG10 |  |  |  |  |
| DLA_LG10_001680 | LG10 |  |  |  |  |
| DLA_LG10_001690 | LG10 |  |  |  |  |
| DLA_LG10_001700 | LG10 |  |  |  |  |
| DLA_LG10_001710 | LG10 |  |  |  |  |
| DLA_LG10_001720 | LG10 |  |  |  |  |
| DLA_LG10_001730 | LG10 |  |  |  |  |
| DLA_LG10_001740 | LG10 |  |  |  |  |
| DLA_LG10_001750 | LG10 |  |  |  |  |
| DLA_LG10_001760 | LG10 |  |  |  |  |
| DLA_LG10_001770 | LG10 |  |  |  |  |
| DLA_LG10_001780 | LG10 |  |  |  |  |
| DLA_LG10_001800 | LG10 |  |  |  |  |
| DLA_LG10_001810 | LG10 |  |  |  |  |
| DLA_LG10_001820 | LG10 |  |  |  |  |
| DLA_LG10_001830 | LG10 |  |  |  |  |
| DLA_LG10_001840 | LG10 |  |  |  |  |
| DLA_LG10_001850 | LG10 |  |  |  |  |
| DLA_LG10_001860 | LG10 |  |  |  |  |
| DLA_LG10_001870 | LG10 |  |  |  |  |
| DLA_LG10_001880 | LG10 |  |  |  |  |
| DLA_LG10_001890 | LG10 |  |  |  |  |
| DLA_LG10_001895 | LG10 |  |  |  |  |
| DLA_LG10_001900 | LG10 |  |  |  |  |
| DLA_LG10_001910 | LG10 |  |  |  |  |
| DLA_LG10_001920 | LG10 |  |  |  |  |
| DLA_LG10_001930 | LG10 |  |  |  |  |
| DLA_LG10_001940 | LG10 |  |  |  |  |
| DLA_LG10_001960 | LG10 |  |  |  |  |
| DLA_LG10_001970 | LG10 |  |  |  |  |
| DLA_LG10_001980 | LG10 |  |  |  |  |
| DLA_LG10_001990 | LG10 |  |  |  |  |
| DLA_LG10_002000 | LG10 |  |  |  |  |
| DLA_LG10_002010 | LG10 |  |  |  |  |
| DLA_LG10_002020 | LG10 |  |  |  |  |
| DLA_LG10_002030 | LG10 |  |  |  |  |
| DLA_LG10_002050 | LG10 |  |  |  |  |
| DLA_LG10_002060 | LG10 |  |  |  |  |
| DLA_LG10_002100 | LG10 |  |  |  |  |
| DLA_LG10_002110 | LG10 |  |  |  |  |
| DLA_LG10_002120 | LG10 |  |  |  |  |
| DLA_LG10_002140 | LG10 |  |  |  |  |
| DLA_LG10_002150 | LG10 |  |  |  |  |
| DLA_LG10_002170 | LG10 |  |  |  |  |
| DLA_LG10_002180 | LG10 |  |  |  |  |
| DLA_LG10_002210 | LG10 |  |  |  |  |
| DLA_LG10_002220 | LG10 |  |  |  |  |
| DLA_LG10_002230 | LG10 |  |  |  |  |
| DLA_LG10_002240 | LG10 |  |  |  |  |
| DLA_LG10_002260 | LG10 |  |  |  |  |
| DLA_LG10_002270 | LG10 |  |  |  |  |
| DLA_LG10_002280 | LG10 |  |  |  |  |
| DLA_LG10_002290 | LG10 |  |  |  |  |
| DLA_LG10_002300 | LG10 |  |  |  |  |
| DLA_LG10_002310 | LG10 |  |  |  |  |
| DLA_LG10_002320 | LG10 |  |  |  |  |
| DLA_LG10_002330 | LG10 |  |  |  |  |
| DLA_LG10_002340 | LG10 |  |  |  |  |
| DLA_LG10_002350 | LG10 |  |  |  |  |
| DLA_LG10_002360 | LG10 |  |  |  |  |
| DLA_LG10_002370 | LG10 |  |  |  |  |
| DLA_LG10_002400 | LG10 |  |  |  |  |
| DLA_LG10_002410 | LG10 |  |  |  |  |
| DLA_LG10_002420 | LG10 |  |  |  |  |
| DLA_LG10_002430 | LG10 |  |  |  |  |
| DLA_LG10_002435 | LG10 |  |  |  |  |
| DLA_LG10_002440 | LG10 |  |  |  |  |
| DLA_LG10_002450 | LG10 |  |  |  |  |
| DLA_LG10_002470 | LG10 |  |  |  |  |
| DLA_LG10_002480 | LG10 |  |  |  |  |
| DLA_LG10_002490 | LG10 |  |  |  |  |
| DLA_LG10_002500 | LG10 |  |  |  |  |
| DLA_LG10_002510 | LG10 |  |  |  |  |
| DLA_LG10_002520 | LG10 |  |  |  |  |
| DLA_LG10_002530 | LG10 |  |  |  |  |
| DLA_LG10_002540 | LG10 |  |  |  |  |
| DLA_LG10_002550 | LG10 |  |  |  |  |
| DLA_LG10_002560 | LG10 |  |  |  |  |
| DLA_LG10_002570 | LG10 |  |  |  |  |
| DLA_LG10_002580 | LG10 |  |  |  |  |
| DLA_LG10_002590 | LG10 |  |  |  |  |
| DLA_LG10_002600 | LG10 |  |  |  |  |
| DLA_LG10_002610 | LG10 |  |  |  |  |
| DLA_LG10_002620 | LG10 |  |  |  |  |
| DLA_LG10_002630 | LG10 |  |  |  |  |
| DLA_LG10_002640 | LG10 |  |  |  |  |
| DLA_LG10_002650 | LG10 |  |  |  |  |
| DLA_LG10_002670 | LG10 |  |  |  |  |
| DLA_LG10_002680 | LG10 |  |  |  |  |
| DLA_LG10_002700 | LG10 |  |  |  |  |
| DLA_LG10_002710 | LG10 |  |  |  |  |
| DLA_LG10_002720 | LG10 |  |  |  |  |
| DLA_LG10_002730 | LG10 |  |  |  |  |
| DLA_LG10_002750 | LG10 |  |  |  |  |
| DLA_LG10_002770 | LG10 |  |  |  |  |
| DLA_LG10_002780 | LG10 |  |  |  |  |
| DLA_LG10_002790 | LG10 |  |  |  |  |
| DLA_LG10_002800 | LG10 |  |  |  |  |
| DLA_LG10_002810 | LG10 |  |  |  |  |
| DLA_LG10_002820 | LG10 |  |  |  |  |
| DLA_LG10_002840 | LG10 |  |  |  |  |
| DLA_LG10_002850 | LG10 |  |  |  |  |
| DLA_LG10_002870 | LG10 |  |  |  |  |
| DLA_LG10_002890 | LG10 |  |  |  |  |
| DLA_LG10_002900 | LG10 |  |  |  |  |
| DLA_LG10_002905 | LG10 |  |  |  |  |
| DLA_LG10_002910 | LG10 |  |  |  |  |
| DLA_LG10_002920 | LG10 |  |  |  |  |
| DLA_LG10_002925 | LG10 |  |  |  |  |
| DLA_LG10_002940 | LG10 |  |  |  |  |
| DLA_LG10_002950 | LG10 |  |  |  |  |
| DLA_LG10_002960 | LG10 |  |  |  |  |
| DLA_LG10_002970 | LG10 |  |  |  |  |
| DLA_LG10_002975 | LG10 |  |  |  |  |
| DLA_LG10_002980 | LG10 |  |  |  |  |
| DLA_LG10_002990 | LG10 |  |  |  |  |
| DLA_LG10_003000 | LG10 |  |  |  |  |
| DLA_LG10_003010 | LG10 |  |  |  |  |
| DLA_LG10_003020 | LG10 |  |  |  |  |
| DLA_LG10_003030 | LG10 |  |  |  |  |
| DLA_LG10_003040 | LG10 |  |  |  |  |
| DLA_LG10_003050 | LG10 |  |  |  |  |
| DLA_LG10_003060 | LG10 |  |  |  |  |
| DLA_LG10_003080 | LG10 |  |  |  |  |
| DLA_LG10_003090 | LG10 |  |  |  |  |
| DLA_LG10_003100 | LG10 |  |  |  |  |
| DLA_LG10_003110 | LG10 |  |  |  |  |
| DLA_LG10_003120 | LG10 |  |  |  |  |
| DLA_LG10_003130 | LG10 |  |  |  |  |
| DLA_LG10_003140 | LG10 |  |  |  |  |
| DLA_LG10_003150 | LG10 |  |  |  |  |
| DLA_LG10_003160 | LG10 |  |  |  |  |
| DLA_LG10_003180 | LG10 |  |  |  |  |
| DLA_LG10_003210 | LG10 |  |  |  |  |
| DLA_LG10_003230 | LG10 |  |  |  |  |
| DLA_LG10_003240 | LG10 |  |  |  |  |
| DLA_LG10_003250 | LG10 |  |  |  |  |
| DLA_LG10_003260 | LG10 |  |  |  |  |
| DLA_LG10_003270 | LG10 |  |  |  |  |
| DLA_LG10_003290 | LG10 |  |  |  |  |
| DLA_LG10_003300 | LG10 |  |  |  |  |
| DLA_LG10_003320 | LG10 |  |  |  |  |
| DLA_LG10_003330 | LG10 |  |  |  |  |
| DLA_LG10_003340 | LG10 |  |  |  |  |
| DLA_LG10_003350 | LG10 |  |  |  |  |
| DLA_LG10_003370 | LG10 |  |  |  |  |
| DLA_LG10_003380 | LG10 |  |  |  |  |
| DLA_LG10_003390 | LG10 |  |  |  |  |
| DLA_LG10_003400 | LG10 |  |  |  |  |
| DLA_LG10_003410 | LG10 |  |  |  |  |
| DLA_LG10_003420 | LG10 |  |  |  |  |
| DLA_LG10_003430 | LG10 |  |  |  |  |
| DLA_LG10_003435 | LG10 |  |  |  |  |
| DLA_LG10_003440 | LG10 |  |  |  |  |
| DLA_LG10_003450 | LG10 |  |  |  |  |
| DLA_LG10_003460 | LG10 |  |  |  |  |
| DLA_LG10_003470 | LG10 |  |  |  |  |
| DLA_LG10_003480 | LG10 |  |  |  |  |
| DLA_LG10_003490 | LG10 |  |  |  |  |
| DLA_LG10_003510 | LG10 |  |  |  |  |
| DLA_LG10_003520 | LG10 |  |  |  |  |
| DLA_LG10_003530 | LG10 |  |  |  |  |
| DLA_LG10_003540 | LG10 |  |  |  |  |
| DLA_LG10_003545 | LG10 |  |  |  |  |
| DLA_LG10_003560 | LG10 |  |  |  |  |
| DLA_LG10_003570 | LG10 |  |  |  |  |
| DLA_LG10_003580 | LG10 |  |  |  |  |
| DLA_LG10_003600 | LG10 |  |  |  |  |
| DLA_LG10_003610 | LG10 |  |  |  |  |
| DLA_LG10_003620 | LG10 |  |  |  |  |
| DLA_LG10_003630 | LG10 |  |  |  |  |
| DLA_LG10_003640 | LG10 |  |  |  |  |
| DLA_LG10_003650 | LG10 |  |  |  |  |
| DLA_LG10_003670 | LG10 |  |  |  |  |
| DLA_LG10_003680 | LG10 |  |  |  |  |
| DLA_LG10_003690 | LG10 |  |  |  |  |
| DLA_LG10_003700 | LG10 |  |  |  |  |
| DLA_LG10_003705 | LG10 |  |  |  |  |
| DLA_LG10_003730 | LG10 |  |  |  |  |
| DLA_LG10_003740 | LG10 |  |  |  |  |
| DLA_LG10_003745 | LG10 |  |  |  |  |
| DLA_LG10_003750 | LG10 |  |  |  |  |
| DLA_LG10_003760 | LG10 |  |  |  |  |
| DLA_LG10_003780 | LG10 |  |  |  |  |
| DLA_LG10_003790 | LG10 |  |  |  |  |
| DLA_LG10_003800 | LG10 |  |  |  |  |
| DLA_LG10_003810 | LG10 |  |  |  |  |
| DLA_LG10_003830 | LG10 |  |  |  |  |
| DLA_LG10_003840 | LG10 |  |  |  |  |
| DLA_LG10_003850 | LG10 |  |  |  |  |
| DLA_LG10_003860 | LG10 |  |  |  |  |
| DLA_LG10_003870 | LG10 |  |  |  |  |
| DLA_LG10_003875 | LG10 |  |  |  |  |
| DLA_LG10_003880 | LG10 |  |  |  |  |
| DLA_LG10_003900 | LG10 |  |  |  |  |
| DLA_LG10_003910 | LG10 |  |  |  |  |
| DLA_LG10_003920 | LG10 |  |  |  |  |
| DLA_LG10_003930 | LG10 |  |  |  |  |
| DLA_LG10_003940 | LG10 |  |  |  |  |
| DLA_LG10_003950 | LG10 |  |  |  |  |
| DLA_LG10_003960 | LG10 |  |  |  |  |
| DLA_LG10_003980 | LG10 |  |  |  |  |
| DLA_LG10_003990 | LG10 |  |  |  |  |
| DLA_LG10_004000 | LG10 |  |  |  |  |
| DLA_LG10_004010 | LG10 |  |  |  |  |
| DLA_LG10_004020 | LG10 |  |  |  |  |
| DLA_LG10_004030 | LG10 |  |  |  |  |
| DLA_LG10_004040 | LG10 |  |  |  |  |
| DLA_LG10_004080 | LG10 |  |  |  |  |
| DLA_LG10_004100 | LG10 |  |  |  |  |
| DLA_LG10_004110 | LG10 |  |  |  |  |
[truncated: 376,660 more chars]
