# Supplementary material for: Genome‐wide analysis of European sea bass provides insights into the evolution and functions of single‐exon genes
Source: Ecol Evol. 2021 Apr 2;11(11):6546–57. doi: 10.1002/ece3.7507 (PMC8207432; doi:10.1002/ece3.7507)
Supplement: Supplementary file 3 — Appendix S3 [file ECE3-11-6546-s001.doc]

### Promoter predictions for SEG/MEG parent sequences with score cutoff 0.80. Transcription start is shown in uppercase for the forward and in bold for the reverse sequence. The gene names of SEG1-15 and MEG1-15 are indicated in the table below

***Block 1***

SEG1: ttgtttataaaataactgtttaaaactcccctctgttttcTtaatttgca
MEG1: ttgtttataaaataactgtttaaaactcccctctgttttcTtaatttgca

***Block 2***

SEG1: aaaataactgtttaaaactcccctctgttttcttaatttgCaatatattt

MEG1: aaaataactgtttaaaactcccctctgttttcttaatttgCaatatattt

***Block 1***

SEG2: gagtgggctttttaagaaggggttaaattacagctactttAaaggactgt
MEG2: gagtgggctttttaagaaggggttaaattacagctactttAaaggactgt
***Block 2***

SEG2: aagcagtctaaatatatatctggttttacagttaaaattcCtgtatttgg

MEG2: aagcagtctaaatatatatctggttttacagttaaaattcCtgtatttgg

***Block1***

SEG3: gcagcaggatgttataaatgttgggaaacaaagtggtaacActtgcaggt
MEG3: gcagcaggatgttataaatgttgggaaacaaagtggtaacActtgcaggt
***Block2***

SEG3: gtttcatttttataaaactcaaacaatttttgacaacagtGcctccttgt
MEG3: gtttcatttttataaaactcaaacaatttttgacaacagtGcctccttgt
***Block 3***

SEG3: atgcgcattgtaaatccctggcacacccgcaccctttgacAcacatagct
MEG3: atgcgcattgtaaatccctggcacacccgcaccctttgacAcacatagct
***Block 4***

SEG3: caaacgagcgctatagaaagggggatgctgcagccccgtcCccctcatat
MEG3: caaacgagcgctatagaaagggggatgctgcagccccgtcCccctcatat

***Block 1***

SEG4: TTGATCCCTTGAAAAAGAGCCGGCAACATATTAAAGAGC**T**GTAATTCCTA
MEG4: TTGGTCCCTTGAAAAAGAGGCGGCTACATATAAAAGAAC**T**ATAATTCCTA
***Block 2***

SEG4: GCCGGCAACATATTAAAGAGCTGTAATTCCTAAACCCTT**C**CTCCAATCTG
MEG4: GGCGGCTACATATAAAAGAACTATAATTCCTAAACCCTT**C**CTCCAATGTG

***Block 1***

SEG5: acagccagccaataaggaggcgtgtcacactcagtcagccAatcaggagc
MEG5: acagccagccaataaggaggcgtgtcacactcagtcagccAatcaggagc
***Block 2***

SEG5: ctaactgttatataacagaagtgtaatgaatcgtaataatTagtgttgat

MEG5: ctaactgttatataacagaagtgtaatgaatcgtaataatTagtgttgat

***Block 1***

SEG6: tcctcctcacctataaagctcttcatggtcaggccccttcAtatcttaaa
MEG6: tcctcctcacctataaagctcttcatggtcaggccccttcAtatcttaaa
***Block 2***

SEG6: tcctatagtctctaaaagtagaacaggagccagagccttcAgttaccaag
MEG6: tcctatagtctctaaaagtagaacaggagccagagccttcAgttaccaag

***Block 1***

SEG7: tctccccaatcaaatagggtccgctggtgacgcccaggctAactggaaaa
MEG7: tctccccaatcaaatagggtccgctggtgacgcccaggctAactggaaaa

***Block 2 (Reverse sequence)***

SEG7: AACTCTTATGTAAATATAATGTGCATATTTACCCATAACT**A**CTGTTATTT
MEG7: AACTCTTATGTAAATATAATGTGCATATTTACCCATAACT**A**CTGTTATTT
***Block 3 (Reverse sequence)***

SEG7: CTTGCAGACTATGTAAAGGGCCACAATTCAAATCATCATC**C**CTTAAAGCA

MEG7: CTTGCAGACTATGTAAAGGGCCACAATTCAAATCATCATC**C**CTTAAAGCA

***Block 1***

SEG8: tgttgtataaaataaaaatctctggtacttacaatcaacgAtgagtttgg
MEG8: tgttgtataaaataaaaatctctggtacttacagtcaacgAtgagtttgg
***Block 2***

SEG8: agtggccgtacaaaaacctcctgcactgtgaacaagcatcTatccactga
MEG8: agtggccgtacaaaaacctcctgcactgtgaacaagcatcTatccactga

***Block 3 (Reverse sequence)***

SEG8: ACTCACAGGACTATAAACCTTCTCAGTTCACTGGAGGATC**A**GCTGACGAT
MEG8: ACTCACAGGACTATAAACCTTCTCAGTTCACTGGAGGATC**A**GCTGACGAT

***Block 1***

SEG9: CCATAGATTTGTAAAAACATCCACGGTCCCCAGACAACCC**C**GTCTCCTAA
MEG9: CCATAGATTTGTAAAAACATCCACGGTCCCCAGACAACCC**C**GTCTCCTAA

***Block 1***

SEG10: tctacatatatatatatagagagagagagtaaaagtacttAtggtgaaga
MEG10: tctacatatatatatatagagagagagagtaaaagtacttAtggtgaaga
***Block 2***

SEG10: gcctcaagcattaaaagcatccagattggagatttgggggAtttccatgc

MEG10: gcctcaagcattaaaagcatccagattggagatttgggggAtttccatgc

***Block 1***

SEG11: acagagcgtctaattcctcccgtgtcttcaccctgctgtcAgtgatctct
MEG11: acagagcgtctaattcctcccgtgtcttcaccctgctgtcAgtgatctct

***Block 2 (Reverse sequence)***

SEG11: GATCTTCTCTTATAAAGGTAGAGCAAATAATCATGAAATC**T**CAGAGGGGT

MEG11: GATCTTCTCTTATAAAGGTAGAGCAAATAATCATGAAATC**T**CAGAGGGGT
 ***Block 3 ( reverse sequence)***

SEG11: CTTTTACATCTAAAAAAGGGGAGCATATTCCACATGTTTT**A**GCCTTTTAC

MEG11: CTTTTACATCTAAAAAAGGGGAGCATATTCCACATGTTTT**A**GCCTTTTAC

***Block 1***

SEG12: tttgtttttctataagaagcctccctgttctccactcattAcctgtatta
MEG12: tttgtttttctataagaagcctccctgttctccactcattAcctgtatta
***Block 2***

SEG12: tgttacctgtataaaagacacctgtccacacacagactccAacctctcca

MEG12: tgttacctgtataaaagacacctgtccacacacagactccAacctctcca

***Block1***

SEG13: cacaatttctgataaaacaccggccgtatgttggctgcgcAaaaaaaaaa
MEG13: cacaatttctgataaaacaccggccgtatgttggctgcgcAaaaaaaaaa
***Block 2***

SEG13: ggtttttgcagtttaaagggccttagtggttcccaacgtgAgggccggga
MEG13: ggtttttgcagtttaaagggccttagtggttcccaacgtgAgggccggga

***Block 3***

SEG13: gcatgccttttaaaaaatcccccagattaagcaatcttggAggagtaaat
MEG13: gcatgccttttaaaaaatcccccagattaagcaatcttggAggagtaaat
***Block 4***

SEG13: cagattgacttttaaatccgctgcaatattgaggagtgatAtaaataaag

MEG13: cagattgacttttaaatccgctgcaatattgaggagtgatAtaaataaag

***Block 1***

SEG 14: ttgaatgatatatatatattcatatttgcatgagccatttGcctgcattt
MEG 14: ttgaatgatatatatatattcatatttgcatgagccatttGcctgcattt
***Block 2***

SEG14: gtatgcaggataacaaaagggcagaccatcgaactgcggcCctgtaaaag
MEG14: gtatgcaggataacaaaagggcagaccatcgaactgcggcCctgtaaaag
***Block 3***

SEG14: tgcggccctgtaaaagagacgagataaactatatttgcctAacaacggtt
MEG14: tgcggccctgtaaaagagacgagataaactatatttgcctAacaacggtt
***Block 4***

SEG14: tgaacaaaattataaacgcaacacttttgtttttgcccccAttcatcatg
MEG14: tgaacaaaattataaacgcaacacttttgtttttgcccccAttcatcatg

***Block 1***

SEG15: ttagtttttctataagaagccttcctgttctccactcattAcctgtatta
MEG15: tttgtttttctataagaagcctccctgttctccactcattAcctgtatta

| **Gene abbreviation** | **Gene name** |
| --- | --- |
| SEG1 | **dicLab1_genemodels_DLAgn_00163390** |
| MEG1 | **dicLab1_genemodels_DLAgn_00163390** |
| SEG2 | dicLab1_genemodels_DLAgn_00174220 |
| MEG2 | dicLab1_genemodels_DLAgn_00174220 |
| SEG3 | **dicLab1_genemodels_DLAgn_00175050** |
| MEG3 | **dicLab1_genemodels_DLAgn_00175040** |
| SEG4 | dicLab1_genemodels_DLAgn_00195400 |
| MEG4 | dicLab1_genemodels_DLAgn_00099540 |
| SEG5 | dicLab1_genemodels_DLAgn_00195400 |
| MEG5 | dicLab1_genemodels_DLAgn_00099540 |
| SEG6 | dicLab1_genemodels_DLAgn_00009720 |
| MEG6 | dicLab1_genemodels_DLAgn_00243340 |
| SEG7 | dicLab1_genemodels_DLAgn_00017170 |
| MEG7 | dicLab1_genemodels_DLAgn_00017160 |
| SEG8 | dicLab1_genemodels_DLAgn_00024440 |
| MEG8 | dicLab1_genemodels_DLAgn_00024430 |
| SEG9 | dicLab1_genemodels_DLAgn_00049170 |
| MEG9 | dicLab1_genemodels_DLAgn_00237040 |
| **SEG10** | **dicLab1_genemodels_DLAgn_00056330** |
| **MEG10** | **dicLab1_genemodels_DLAgn_00056330** |
| SEG11 | dicLab1_genemodels_DLAgn_00066030 |
| MEG11 | dicLab1_genemodels_DLAgn_00066020 |
| SEG12 | dicLab1_genemodels_DLAgn_00079640 |
| MEG12 | dicLab1_genemodels_DLAgn_00255410 |
| **SEG13** | **dicLab1_genemodels_DLAgn_00083790** |
| **MEG13** | **dicLab1_genemodels_DLAgn_00083790** |
| SEG14 | dicLab1_genemodels_DLAgn_00129060 |
| MEG14 | dicLab1_genemodels_DLAgn_00129050 |
| SEG15 | dicLab1_genemodels_DLAgn_00205030 |
| MEG15 | dicLab1_genemodels_DLAgn_00255410 |
